# Transfer of Viral Communities between Human Individuals during Fecal Microbiota Transplantation

**DOI:** 10.1128/mBio.00322-16

**Published:** 2016-03-29

**Authors:** Christel Chehoud, Anatoly Dryga, Young Hwang, Dorottya Nagy-Szakal, Emily B. Hollister, Ruth Ann Luna, James Versalovic, Richard Kellermayer, Frederic D. Bushman

**Affiliations:** a Department of Microbiology, University of Pennsylvania School of Medicine, Philadelphia, Pennsylvania, USA; b Department of Chemistry, Saint Petersburg State University, Saint Petersburg, Russia; c Section of Pediatric Gastroenterology, Department of Pediatrics, Baylor College of Medicine, Texas Children’s Hospital, USDA/ARS Children’s Nutrition Research Center, Houston, Texas, USA; d Texas Children’s Microbiome Center, Department of Pathology, Texas Children’s Hospital, Houston, Texas, USA; e Department of Pathology and Immunology, Baylor College of Medicine, Houston, Texas, USA

## Abstract

Fecal microbiota transplantation (FMT) is a highly effective treatment for refractory *Clostridium difficile* infections. However, concerns persist about unwanted cotransfer of pathogenic microbes such as viruses. Here we studed FMT from a single healthy human donor to three pediatric ulcerative colitis patients, each of whom received a course of 22 to 30 FMT treatments. Viral particles were purified from donor and recipient stool samples and sequenced; the reads were then assembled into contigs corresponding to viral genomes or partial genomes. Transfer of selected viruses was confirmed by quantitative PCR. Viral contigs present in the donor could be readily detected in recipients, with up to 32 different donor viral contigs appearing in a recipient sample. Reassuringly, none of these were viruses are known to replicate on human cells. Instead, viral contigs either scored as bacteriophage or could not be attributed taxonomically, suggestive of unstudied phage. The two most frequently transferred gene types were associated with temperate-phage replication. In addition, members of *Siphoviridae*, the group of typically temperate phages that includes phage lambda, were found to be transferred with significantly greater efficiency than other groups. On the basis of these findings, we propose that the temperate-phage replication style may promote efficient phage transfer between human individuals. In summary, we documented transfer of multiple viral lineages between human individuals through FMT, but in this case series, none were from viral groups known to infect human cells.

## IMPORTANCE

Transfer of whole communities of viruses between humans has rarely been studied but is of likely medical importance. Here we studied fecal microbiota transplantation (FMT), a highly successful treatment for relapsing *Clostridium difficile* infection and, potentially, other gastrointestinal (GI) diseases. We investigated the transfer of viral communities during FMT and documented transfer of multiple viral lineages between humans. None of these were viruses that replicated on animal cells or that are known to be pathogenic. We found that temperate bacteriophage, which form stable associations with their hosts, were significantly more likely to be transferred during FMT. This supports a model in which the viral temperate replication style may have evolved in part to support efficient viral transmission between environments.

## INTRODUCTION

Fecal microbiota transplantation (FMT) has been used to treat relapsing *Clostridium difficile* infection with great success and shows promise for treatment of other indications (1, 2). However, many pathogenic human viruses are known to be transmitted via the fecal-oral route, and viral transfer accompanying FMT has not been studied previously. Here we investigated viral transfer during FMT using model-independent metagenomic sequencing.

Introduction of single viruses into humans has been closely studied, but the transfer of populations of viruses is much less well understood. Multiple viruses are commonly found as coinfections in humans, though it is usually unknown whether acquisition of these viruses was sequential or simultaneous. Careful tracking of transmitted founder viruses during human immunodeficiency virus (HIV) and hepatitis C virus (HCV) transmission has revealed examples of probable simultaneous transfer of multiple lineages (3, 4). HIV and HCV commonly co-occur and may be acquired simultaneously during intravenous drug use (5). In the case of satellite viruses (e.g., hepatitis B virus and its satellite virus hepatitis D virus), the two are inferred to commonly infect together because the satellite depends on its helper for replication (6). Multiple viruses that replicate in human cells may be transferred together during organ transplantation (7). For bacterial viruses, simultaneous acquisition of multiple lineages has rarely been investigated.

The human fecal virome is immense in the numbers of viruses present—comparable to the number of bacteria, which is 10^11^ per g of feces—and the number of different types. Gut bacteriophages are so numerous and diverse that sequence databases contain only a small fraction of the global population (8–14). Thus, simply sequencing total DNA from stool and aligning to database genomes generally misses most of the bacteriophage sequences present, necessitating specialized methods.

In this study, we investigated the transfer of viral communities between humans through FMT and characterized features associated with particularly efficient transmission. We took advantage of a case series where feces samples from a single donor were used to treat three children with ulcerative colitis (UC) (15). These subjects received a particularly intensive procedure, which consisted of 22 to 30 FMT treatments per subject delivered by colonoscopy or enema during a 6 to 12 week period. We performed metagenomic analysis of viral particle fractions purified from fecal samples from the donor, the donor product, and recipients before, during, and after transplantation. To track viral transfer, we assembled contigs representing viral genomes and monitored their abundances through the transplantation process. All recipients were detectably colonized by multiple donor viruses, documenting transfer of whole viral populations.

## RESULTS

### Subjects studied.

**FIG 1:**
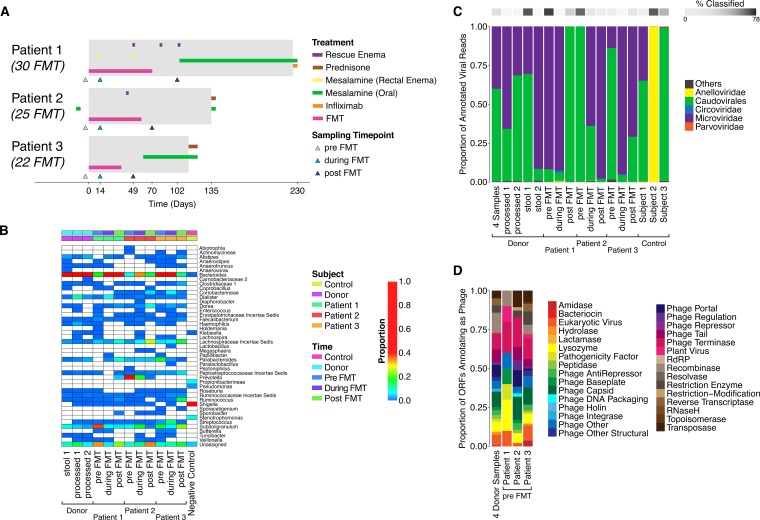
Fecal microbiota transplantation (FMT) to treat ulcerative colitis. (A) Diagram of the treatment regimens. Patient 1 received 30 rounds of FMT, patient 2 received 25, and patient 3 received 22. All patients were in remission (gray-shaded boxes) while receiving the FMT course and remained in remission for more than 11 weeks following their last FMT. However, all three eventually experienced a relapse requiring immunotherapy. Administration of mesalamine, given orally or through a rectal enema, was allowed during the trial, depending on the clinical disease activity. Three samples (indicated as triangles) were taken from each patient: one before the beginning of the FMT course (pre), the second after the 13th FMT (during), and the third 2 to 3 weeks after finishing the FMT course (post). (B) Bacterial lineages in the samples studied. Bacterial taxonomic representation and abundance were characterized by sequencing of 16S rRNA gene tags. Details are described in reference 15. (C) Viral families detected in the samples studied, assessed by alignment of reads to a curated database of viral sequences (18). (D) Gene types inferred from analysis of the VLP contigs. VLP contigs were analyzed for open reading frames (ORFs), and then ORFs were annotated using the CDD. The output was interpreted using custom annotation relating Pfam domains to viral gene functions. Gene types identified are summarized at the right in the figure. The percentages of classified phage ORFs from the four donor samples, patient 1, patient 2, and patient 3 were 8.9%, 7.1%, 3.8%, and 9.5%, respectively.

Three pediatric UC patients receiving immunotherapy regimens were switched to receiving a course of 22 to 30 FMT treatments via colonoscopy or enema during a 6 to 12 week period (Fig. 1A) (15). Donor stool specimens were from a single, healthy, 37-year-old male. FMT in all three patients was associated with a symptom-free period of at least 4 weeks, supporting the temporary withdrawal of immunotherapy (no treatment other than mesalamine). Treatment was associated with endoscopic and histologic remission for at least 2 weeks after the last FMT. All three subjects eventually relapsed and resumed therapy with immunomodulators.

### Analysis of transfer of bacterial lineages.

Bacterial microbiota composition was investigated by purifying DNA from stool and sequencing a segment of the 16S rRNA gene as previously described (15). The donor samples contained high levels of *Bacteroides* and lower levels of *Firmicutes* such as *Dialister* and *Subdoligranulum*. Prior to FMT, the three recipients harbored communities dominated by *Subdoligranulum*, *Prevotella*, and *Bacteroides*, respectively. During FMT, *Bacteroides* dominated in all three recipients (Fig. 1B). After completion of the FMT treatment, *Bacteroides* persisted, but *Firmicutes* lineages also increased in abundance in recipients 2 and 3. Global community analysis showed that for all three recipients, the composition of microbial communities during FMT became detectably (though modestly) closer to that of the donor. After FMT, all three recipients’ microbial communities increased in diversity (see Fig. S1 in the supplemental material), suggestive of acquisition of healthier microbiota.

### Composition of the fecal virome in donors and recipients.

To study viral transfer, virus-like particles (VLPs) were purified from 18 samples. We compared four donor samples of two types: two were crude stool samples, and the other two were processed to generate the liquid product used for FMT. Recipient samples were taken prior to FMT, during the FMT intervention (after the 13th FMT treatment), and after the FMT was completed. The five control samples were (i) a stool sample from a healthy 25-year-old male, (ii) two stool samples from pediatric Crohn’s disease patients on immunotherapy but not receiving FMT, and (iii) two buffer-only negative-control samples. The first three control samples were from well-studied subjects and served as positive controls. The two pediatric Crohn’s disease patients were selected because a previous metagenomic study had shown them to harbor anelloviruses (Torque teno virus) and parvoviruses (Human bocavirus) (16). The two buffer controls were included to document contamination from reagents and environmental admixture.

VLPs were purified from the 18 samples, and then DNA was isolated from the particles. VLP DNA was amplified using multiple-displacement amplification (MDA) and the product sequenced using Illumina technology. Quantitative PCR (qPCR) for 16S was used to assay contaminating bacterial DNA, documenting only low-level carryover (see Table S1 in the supplemental material). Here we investigated only DNA viruses. RNA viruses are less abundant in stool than DNA viruses, and results of previous studies performed by us and others suggest that many of the RNA viruses present in stool are transient plant RNA viruses derived from food (17; unpublished data).

At least one million reads were acquired from each fecal sample (see Table S2 in the supplemental material), allowing detailed investigation of the viral populations. Low-quality reads were filtered out, as were reads mapping to the human genome (hg18) and phage phiX174 (the latter is added to sequencing mixtures for technical reasons). Reads were aligned to a curated viral database (18) using a permissive alignment threshold (BLAST E value 10^−3^). Low-abundance negative-control samples (<50,000 reads) were filtered out, and plots of the proportions of the most abundant taxa are shown (Fig. 1C). The majority of reads did not align to reference database sequences (an average of 17.9% of reads returned significant matches).

Of the reads that did align, the most common assignments represented *Microviridae*, which are nonenveloped, single-stranded DNA phages from the family that includes phiX174, and caudovirales, the members of which comprise a broad family, including all of the tailed phages. Of the two controls with known animal cell viruses, anelloviruses were recovered efficiently and bocavirus less efficiently. The MDA step is known to amplify single-stranded circular DNA particularly efficiently, likely explaining the abundance of anelloviruses and *Microviridae*, both of which have small single-stranded circular DNA genomes.

To examine the VLP genomes in our samples more fully, reads were assembled into contigs using deBruijn graph assembly (19) and overlap consensus methods (20). A total of 3,634 contigs were built from the four donor samples (see Table S3 in the supplemental material). Of these, 261 were greater than 3,000 bp in length (mean length, 8,161 bp; maximum length, 65,335 bp). For the recipients, totals of 486, 1,177, and 1,936 contigs were built from each pre-FMT sample; among these, 38, 138, and 127 contigs were greater than 3,000 bp in length (see Table S4). These contigs were assessed for length, circularity, and number of open reading frames (ORFs). Nine of the 261 contigs were circular, suggesting that we had sequenced the entire viral genome (see Fig. S2). With one exception, these clustered at specific lengths suggestive of anelloviruses (~3,000 bp), *Microviridae* (~5,000 to 6,000 bp), and *Siphoviridae* (typically >30,000 bp). One circular contig (8,662 bp) was annotated as *Podoviridae* (typically >40,000 bp), suggesting that it may have assembled as a circle due to the presence of a repeated sequence. Linear contigs are either complete linear genomes or genome fragments.

### Proteins encoded in the gut virome.

To catalog the genes present, open reading frames (ORFs) were predicted, conceptually translated, and aligned to several protein databases (19, 21, 22), including one composed of conserved protein domains (from the Conserved Domains Database [CDD]) known to be associated with viruses (12, 14). A total of 3,321 ORFs were predicted. Protein types found in the donor and in each recipient pre-FMT are summarized in Fig. 1D. Only an average of 7.3% of proteins were annotated, which emphasizes the size of the pool of uncharacterized bacteriophage genes. Prominent CDD types were associated with phage capsids, tails, portals, terminases, and other structural components. Additional annotations, including bacteriocin, beta-lactamase, reverse transcriptase, and restriction modification, were associated with effector proteins.

Contigs of length >3,000 bp had on average 6 ORFs, allowing us to use gene family membership to annotate the probable viral family of origin. We found that each contig had on average two ORFs matching a viral genome (BLAST E value 10^−5^). The majority of classified VLP genomes belonged to tailed phages (caudovirales), which include *Siphoviridae*, *Myoviridae*, and *Podoviridae*. *Microviridae* were also prominent. In several cases, single ORFs annotated as belonging to animal cell virus lineages (*Poxviridae*, *Adenoviridae*, and *Herpesviridae*), but multiple other ORFs on the same contig annotated as caudovirales, so in these cases the caudovirales attribution was retained, or multiple other ORFs were unclassified, weakening the animal cell virus assignment. The two Crohn’s disease samples preselected for containing animal cell viruses returned contigs representing anellovirus and bocavirus as expected.

### Transfer of viral lineages with FMT.

**FIG 2:**
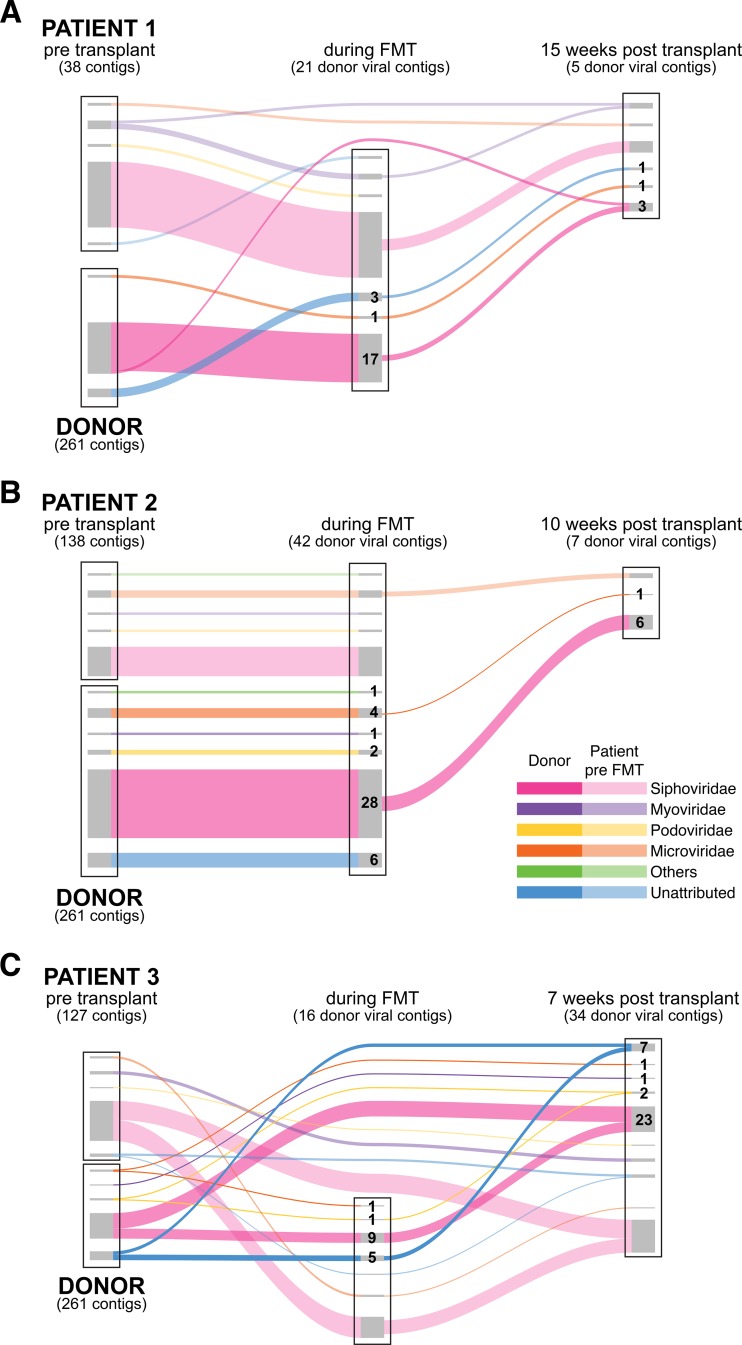
Transfer of phage between human individuals. Transmission is shown using Sankey diagrams. Data from patients 1 to 3 are shown from top to bottom. For each subject, contigs of >3,000 bp are shown as the box at the left, with recipient contigs on top and donor contigs below. In the middle, contigs detected at the FMT time point are shown; to the right are contigs detected after cessation of FMT (post). Curves with darker colors show the persistence of donor contigs and curves with lighter colors the persistence of recipient contigs. The color of each strand represents a single viral family. Numbers indicate the number of donor contigs transferred from each viral family.

We then used the contigs of >3,000 bp to investigate the transfer of phages during FMT (Fig. 2; see also Fig. S3 in the supplemental material). To score detection of a donor contig in a recipient, we required >50% coverage of the contig and alignment of at least 5 paired reads, where both reads in each pair showed high-quality alignment to the target contig. We note that these criteria are conservative, so the proposed numbers of contigs transferred represent minimal estimates. Comparing to recipient samples at the first time point after initiation of FMT, while FMT was ongoing, we detected 21, 42, and 16 donor contigs, respectively. At the second time point, 5 to 13 weeks later, and 2 to 4 weeks after the completion of the FMT treatment, we detected 5, 7, and 34 donor viral contigs, respectively. Fig. S4 shows examples of sequence alignments of reads from recipients at time points following transfer onto contigs built from donor reads, illustrating high coverage.

**FIG 3:**
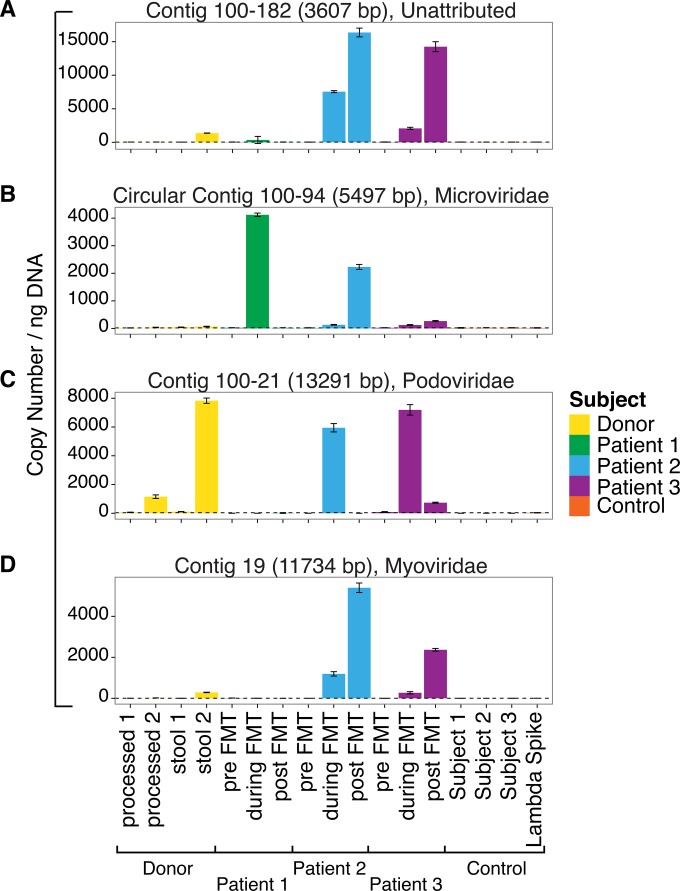
Analysis of repeatedly transferred VLP contigs by qPCR. VLP contigs built from donor samples that are indicated by sequence data to be present in patients after FMT treatment, but not in recipient pre-FMT samples, were quantified by qPCR. VLP DNA from stool was tested without the use of Genomifi amplification (to reduce distortions of abundance data). Abundance was quantified from 4 samples from the donor (two whole stool and two purified product prepared for FMT), from samples from the 3 recipient patients (at three time points each), and from samples from 3 control individuals. As a negative control, lambda DNA was also tested (far right). The dashed line along the baseline indicates the inferred background level in the lambda DNA negative control. Contig length, circularity, and viral lineage as attributed by ORF annotation are indicated on each panel. (A) Contig 100-182 (unattributed). (B) Circular contig 100-94 (*Microviridae*). (C) Contig 100-21 (*Podoviridae*). (D) Contig 19 (*Myoviridae*).

To confirm the transfer from the donor to recipients, we used quantitative PCR (qPCR) to analyze VLP DNA samples (Fig. 3). As the template, we analyzed fecal VLP DNA specimens that were not subjected to MDA amplification, which is known to distort relative abundances. To confirm that some lineages transferred efficiently, we analyzed four VLP contigs from the donor that were detected in at least two different recipients. qPCR assays were designed for each of the four contigs (see Table S5 in the supplemental material), and positive-control DNAs were synthesized for use as copy number standards. We compared results from the four donor samples to those from the nine recipient samples (three patients, three time points each). We also tested the same positive controls used in the viral sequencing analysis, specifically, stool VLP DNA from a healthy 25-year-old male and from two pediatric Crohn’s disease patients. As a negative control, we used a commercial phage lambda DNA sample.

All four qPCR assays detected the expected contigs in the donor samples and in at least two recipients. Levels were low in all controls. For contig 100-94 (annotating as *Microviridae*) and contig 100-182 (unattributed), donor contigs were detected after FMT in all three recipients (Fig. 3A and B). For contigs 100-21 (*Podoviridae*) and 19 (*Myoviridae*), transfer was robust to subjects 2 and 3 (Fig. 3C and D). The maximum copy numbers detected were in the range of 8 × 10^3^ to 15 × 10^3^ copies per nanogram of VLP DNA.

### Viral features associated with efficient viral transfer.

**FIG 4:**
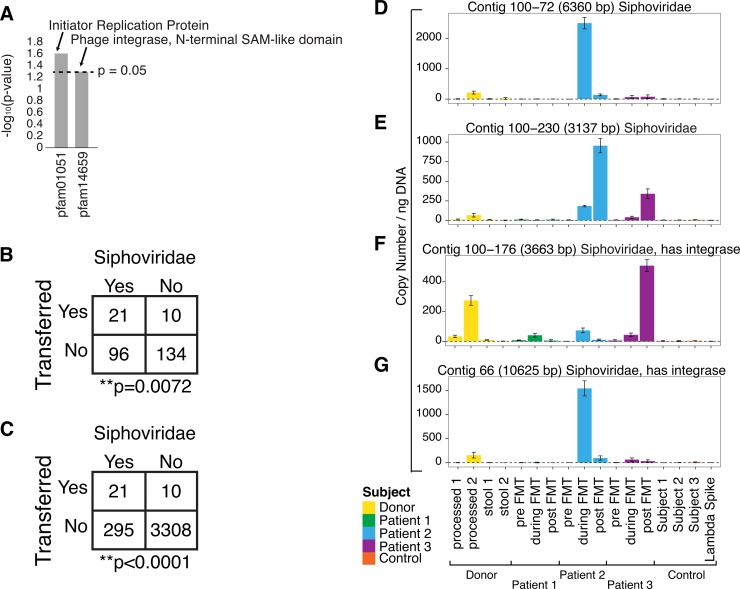
Preferential transfer of *Siphoviridae* between human individuals. (A) Top differentially transferred genes based on CDD annotation in the Pfam database. A total of 302 CDD hits were found in the contigs. The top two domains (*P* value, <0.05; odds ratio, >4) based on CDD annotation which were most frequently transferred are shown. (B) Comparison of the frequencies of transfer to least 2 of the 3 patients for *Siphoviridae* contigs versus all others contigs of >3,000 bp. Transfer is defined here as achieving >50% coverage and >5 paired reads, where the two reads in each pair detect the same contig. If transfer is defined as detection of a donor contig during FMT, after FMT, or both in at least one patient, the favoring of *Siphoviridae* achieves a *P* value of <0.0001. (C) Comparison of the frequencies of transfer to least 2 of the 3 patients for *Siphoviridae* contigs versus all other contigs built. (D to G) Contigs annotating as *Siphoviridae* found in the donors and suggested by sequence data to be transferred to multiple recipients were validated using qPCR. The samples tested and the annotation details were the same as those described for Fig. 3. (D) Contig 100-72. (E) Contig 100-230. (F) Contig 100-176. (G) Contig 66.

We next investigated features of viral contigs associated with efficient transmission during stool transplantation. We first asked whether any inferred phage-encoded proteins could be identified that correlated with efficient transmission. Figure 4A shows the top two conserved domain families that correlated with frequency of transmission (*P* values, <0.05; odds ratio, >4.2 times more likely to be transferred). These transferred domains were found to be associated with the temperate replication style, in which phages form quiescent long-term associations with their bacterial hosts. The highest-scoring family (pfam01051) is annotated as an “initiator replication protein.” The best-studied member of this group is RepA protein of the temperate-phage P1. RepA initiates DNA replication on the P1 genome, and related proteins also mediate replication of herpesviruses. The second-highest-scoring family (pfam14659) is a domain from the lambda integrase family of integration proteins. Lambda integrase is well known to mediate integration during prophage formation. Thus, the top two genes associated with preferential transfer were indicative of the temperate replication style.

We next asked whether any viral families were preferentially transferred by comparing frequencies in the donor versus recipient VLP populations. We focused the analysis on our most accurate detections, specifically, the donor viral pool consisting of contigs of >3,000 bp. Comparison showed that *Siphoviridae* were significantly more likely to be transferred (*P* = 0.0072 by the Fisher exact test; Fig. 4B). We repeated these tests by looking at *Siphoviridae* compared to all contigs and again found *Siphoviridae* to be significantly more likely to be transferred (*P* < 0.0001 by the Fisher exact test; Fig. 4C). *Siphoviridae* include phage lambda, 434, and P22 and are known be rich in temperate phage that have the capacity to integrate into the host cell genome (23).

To confirm transfer, we selected four contigs annotating as *Siphoviridae* and called as transferred and designed qPCR assays to verify transfer. VLP DNA samples (not subjected to MDA) were tested, and contigs were detected in the donor and recipient as expected from the sequencing data (Fig. 4D to G). In all four cases, the transferred phage were detected in at least two of the three recipients, indicating transfer to multiple individuals.

## DISCUSSION

Here we report transfer of gut viral communities associated with FMT. No viruses infecting human cells were detected in the donor; thus, reassuringly, no transfer could be documented in recipients. Numerous recipient VLP sequence reads showed high-quality alignments to donor VLP contigs, suggesting transfer with FMT. Many were annotated as containing genes known to be phage encoded, supporting the inference that the VLP contigs corresponded to phage. From 5 to 34 transferred contigs could be detected in recipient samples. Simultaneous transfer of multiple viruses has rarely been documented in humans but was readily detected here, potentially associated with the large number of FMT treatments (22 to 30 per subject). On the basis of these observations, we propose that transfer of bacteriophage populations is a general characteristic of FMT.

An alternative interpretation for our data could be that viruses were not transferred from donor to recipients but instead were present in recipients at levels that were initially below the level of detection. According to this view, viruses preexisting in the recipient would have grown out after FMT, resulting in the spurious appearance of transfer. We cannot completely rule out this possibility, but several aspects of phage biology in the human gut support our interpretation. Bacteriophage populations in the intestines of healthy humans are known to show extreme differences among individuals (8–14). However, within an individual, populations show notable longitudinal stability, at least in healthy subjects (8, 10, 11). As a result, most VLP genomes are likely recognizable by their individual of origin. Thus, while it is possible that some new examples of detection in recipients represent the outgrowth of preexisting viruses, or even *de novo* colonization, we expect that most recipient sequences resembling donor VLPs were genuinely derived from the donor stool product.

Our most unexpected finding was that lysogeny is associated with efficient transfer by FMT. This was indicated first by the preferential transfer of the *Siphoviridae*, the group that includes phage lambda and is known to be rich in temperate phage. Analysis of genes associated with transfer also made this point (Fig. 4A). The most strongly associated gene encoded the RepA protein, important for replication of the temperate-phage P1, and the second most strongly associated gene encoded a domain of the lambda integrase protein, important for prophage formation.

On the basis of our findings, we propose that the temperate-phage replication style may have evolved in part to optimize phage transmission between environments. If bacteriophage must pass through hostile environments to reach locations favorable for replication, transportation as a prophage may promote dispersal. Transmission of gut viruses between human hosts potentially involves persistence outside the gut, likely representing a transmission barrier. Viruses transferred efficiently by the fecal-oral route are rarely enveloped, suggesting that tough physical properties may be needed to survive fecal-oral transmission. In feces, an enveloping membrane may be damaged by the detergent effects of bile salts or by drying after shedding in feces. Respiratory viruses, in contrast, are commonly spread on respiratory droplets and are often enveloped. Integration of prophage DNA into the bacterial genome may provide a protective vehicle supporting phage transmission despite exposure to feces or other hostile environmental pressures. Thus, we propose that the temperate-phage replication style may in part have evolved to maximize survival during transmission.

In summary, we presented a metagenomic survey of DNA viruses transferred during FMT. Numerous temperate phage were found to be transferred, but no viruses were detected that corresponded to pathogenic viruses that infect human cells. The materials and methods employed for preparing and introducing feces into patients differ and may influence the likelihood of viral transmission, so additional characterization of viral transfer can help guide development of safest practices.

## MATERIALS AND METHODS

### Human subjects.

Human subjects and methods used for FMT have been previously described (15). Fresh stool specimens from the healthy adult donor were divided into ~50-g aliquots. Sterile normal saline solution was added prior to homogenization in a strainer bag with 500-µm pores (Seward Laboratory Systems Inc., Port Saint Lucie, FL) using a Smasher Laboratory Blender/Homogenizer (AES Chemunex Inc., Cranbury, NJ). Both fresh stool samples and the homogenized stool preparations used for the transplantation were analyzed here. Experiments were carried out under a protocol approved by the Institutional Review Board of Baylor College of Medicine (H-30591).

### DNA purification and sequencing.

Detailed methods are presented in Text S1 in the supplemental material and summarized here. Phage particles were purified from stool samples by suspension in SM buffer and filtration through a 0.22-µm-pore-size filter (EMD; Millipore). The filtrate was concentrated using a 100-kDa-molecular-mass Centricon Plus 70 cutoff filter (Millipore), resuspended in 40 ml SM buffer (50 mM Tris-HCl pH 7.5, 100 mM NaCl, 8 mM MgSO_4_, 0.002% gelatin), and concentrated. The concentrate was treated with DNase I and RNase (Roche) at 37°C for 30 min to eliminate nonencapsulated nucleic acids, and then the enzymes were deactivated at 65°C for 15 min. Total phage DNA was extracted from particles using a QIAamp DNA stool kit (Qiagen). Extracted phage DNA was amplified using an Illustra GenomiPhi V2 DNA amplification kit (GE Healthcare). Libraries were made using an Illumina Nextera XT Samples Prep kit and quantified using a SYBR Fast Illumina library quantification kit (Kapa Biosystems).

Purified VLP DNAs were sequenced on three MiSeq runs (250 bases per read, paired-end sequencing) according to the instructions of the manufacturer (Illumina). Two negative-control samples yielded 1,242 and 36,156 reads, respectively. Contigs were built from the four donor samples and were annotated with open reading frames (ORFs), integrase genes, and viral family names based on similarity to reference viral genomes. ORFs were also aligned to entries in a database of conserved domain integrase genes (to assess potential temperate replication style) and also the Virulence Factor Database, Aclame database of mobile genetic elements, NCBI’s reference viral database, NCBI’s reference bacterial database, and NCBI’s nucleotide database to assess additional functional features (summarized in Tables S3 and S4 in the supplemental material). Reads from all of the samples were mapped to contigs built from the four donor samples using Bowtie2 (24). Coverage metrics were calculated, including the rate of concordance between paired reads, the average depth of coverage, and the percentage of each contig that was covered by reads.

### qPCR validation.

The qPCR analysis for quantitating contigs was performed using FastSYBR green master mix (Applied Biosystems). Primers are described in Table S5 in the supplemental material. ZeroBlunt TOPO vector (Thermo, Fisher Scientific), containing the corresponding gBLOCK synthetic copy number control sequence, was linearized and used as a standard control.

Quantification of 16S rRNA copy numbers was performed using the real-time TaqMan method with TaqMan Environmental master mix 2.0 and primers as indicated in Table S5 in the supplemental material ZeroBlunt TOPO vector containing a nearly full-length clone of *Escherichia coli* 16S was linearized and used as a standard control.

### Statistical analysis.

The Fisher exact test and odds ratios were used to assess significance of conserved domains or viral family transfer.

### Assessing contamination of phage DNA preparations with bacterial and human DNA.

Purities of the phage DNA preparations were assessed using qPCR to quantify bacterial 16S rRNA gene copies (see Table S1 in the supplemental material). Values ranged from 7 to 5,021 16S rRNA gene copies/ng of purified phage DNA (average, 257 copies/ng). The contribution of bacterial DNA per nanogram of purified phage DNA could be estimated by assuming that the average bacterial genome has 2 to 5 rRNA gene copies and is 2.5 to 5.5 Mb (25) in total (molecular weight [MW], 1.6 × 10^9^ to 3.3 × 10^9^), implying that, on average, 1.0 nanograms of phage DNA was contaminated with 1.1 pg of bacterial DNA.

Human DNA contamination was assayed by aligning sequence reads from the VLP DNA samples to the human genome. Most samples contained <1% human DNA; the highest value recorded was 4% (see Table S2 in the supplemental material).

We thus concluded that bacterial DNA and human DNA were minor contributors to the VLP genomic DNA samples studied.

## SUPPLEMENTAL MATERIAL

Figure S1  Bacterial community analysis. (A) Shannon diversity. (B) Weighted Unifrac ordination. (C) Bacterial weighted Unifrac distance from donor centroid. Download Figure S1, PDF file, 0.2 MB

Figure S2  Example of circular contigs. Data represent numbers of reads mapping to each circular donor contig from each of the samples. The size of the circle represents the percentage of coverage of the contig (i.e., a small circle means less of the contig was covered by reads, while a large circle means that the entire contig was covered by reads from that sample). Contig names include lengths and putative family names. Family names were abbreviated as follows: Sipho, *Siphoviridae*; Micro, *Microviridae*; Podo, *Podoviridae*; NA, unattributed. Download Figure S2, PDF file, 0.2 MB

Figure S3  Putative viral families of transferred contigs. For each patient, the number and family name of the contigs that were built from the donor sample and the patient pre-FMT samples are listed. Contigs that transferred from the donor to a patient’s second time point (during FMT), to a patient’s third time point (post-FMT), or to both the second and third time points are listed. Similarly, contigs that transferred from a patient’s first time point (pre-FMT) to subsequent time points are listed. Download Figure S3, PDF file, 0.2 MB

Figure S4  Example of alignment of reads to a contig. The reads from each sample were aligned to circular donor contig 100-94. The reads from each sample that mapped to this contig are shown across the 5,497-bp contig length. The *y* scale (number of reads) is indicated in the brackets on top of each coverage map. Donor stool 1, patient 1 pre-FMT, patient 1 post-FMT, patient 2 pre-FMT, and patient 3 pre-FMT samples had no reads aligning to this contig. The inset on the top left corner is the same plot as that shown in Fig. S2 and shows the summarized coverage results. Download Figure S4, PDF file, 0.4 MB

Table S1  16S copy number. 16S qPCR was used to detect the quantity of contaminating bacterial DNA in each sample. Table S1, PDF file, 0.01 MB

Table S2  Human reads in the samples analyzed. Data represent the number of reads (~250 bp) sequenced in each sample before and after human filtering. Table S2, PDF file, 0.02 MB

Table S3  Donor contigs. Data represent information on contigs of >500 bp built from the four donor samples. Table S3, PDF file, 1.2 MB

Table S4  Patient pre-FMT contigs. Data represent information on contigs of >3,000 bp separately built from the patient 1 pre-FMT sample, patient 2 pre-FMT sample, and patient 3 pre-FMT sample. Table S4, PDF file, 0.2 MB

Table S5  Primers used for qPCR analysis. Data represent the oligonucleotides used for 16S and VLP contig verification qPCR. Table S5, PDF file, 0.02 MB

Text S1  Supplemental methods. Download Text S1, DOCX file, 0.03 MB
