# Supplementary figures and images for: Transfer of Viral Communities between Human Individuals during Fecal Microbiota Transplantation
Source: mBio. 2016 Mar 29;7(2):e00322-16. doi: 10.1128/mBio.00322-16 (PMC4817255; doi:10.1128/mBio.00322-16)

Figure S1

A

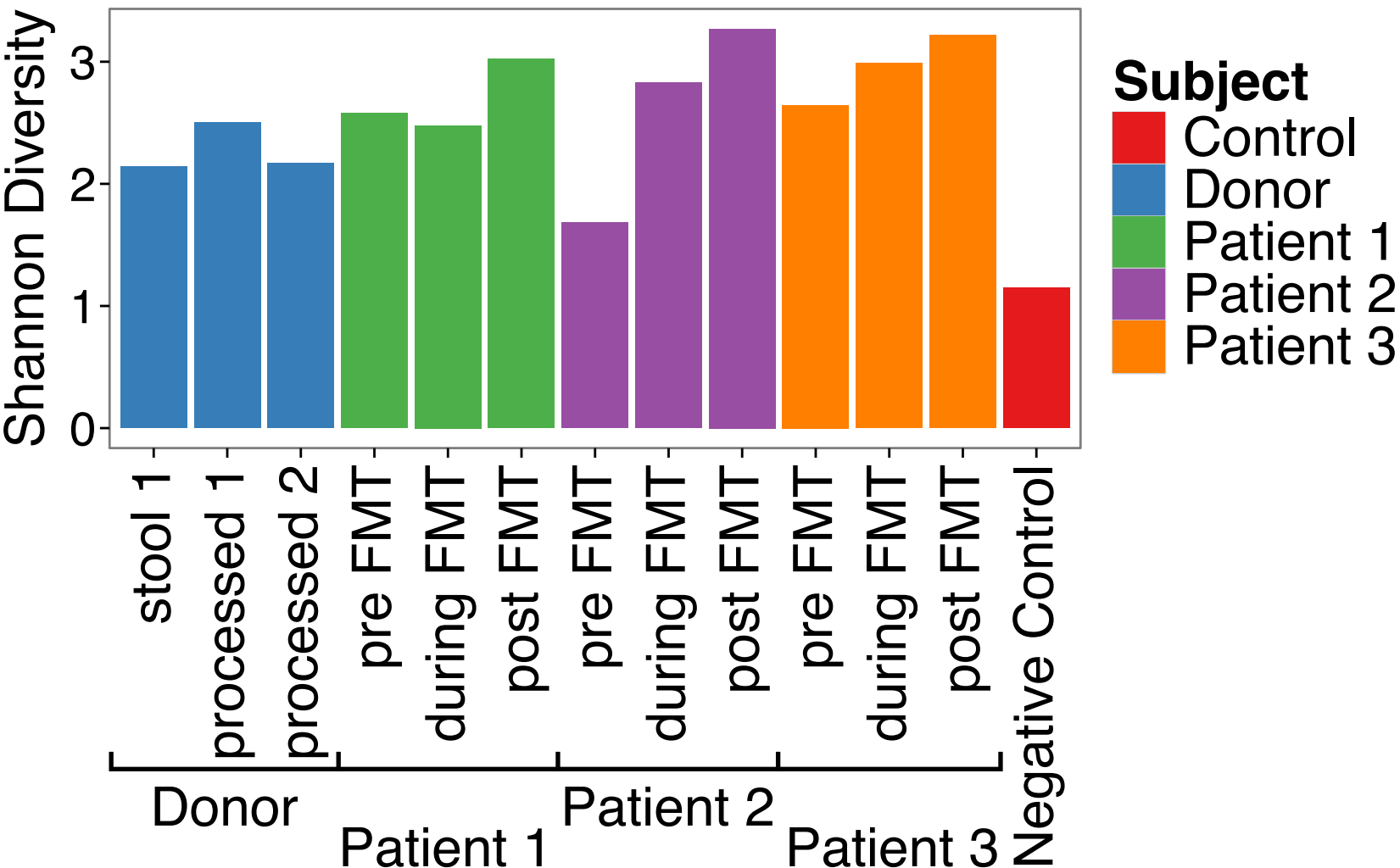

B

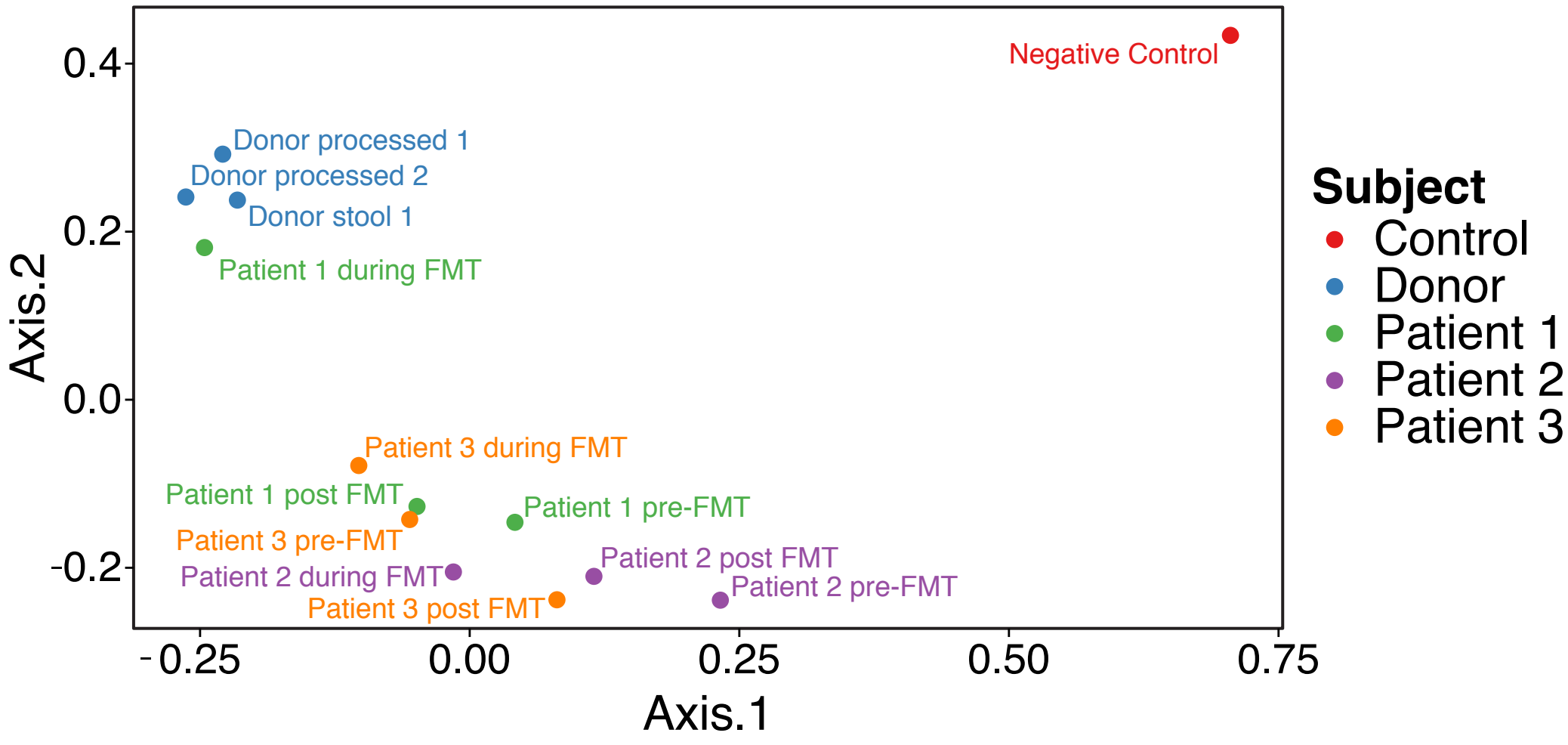

C

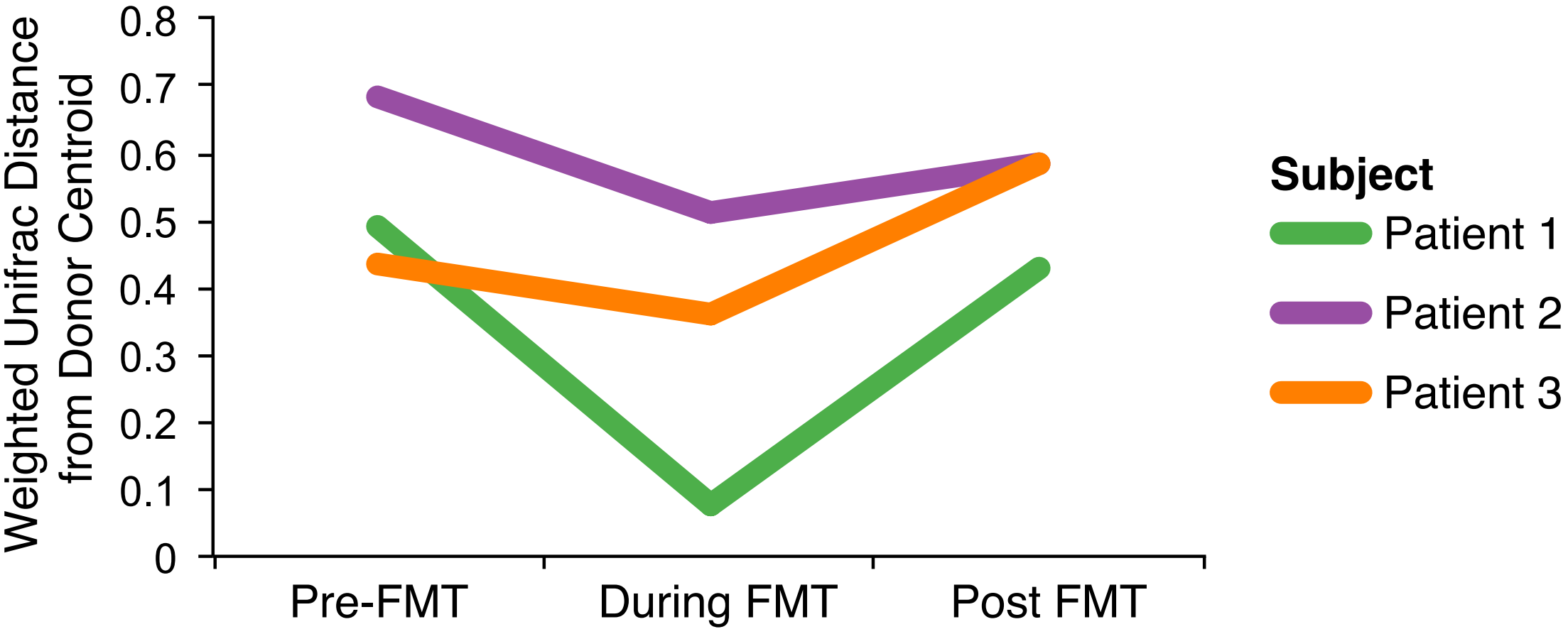

Figure S1: Bacterial Community Analysis

Supplement: Figure S1 — Bacterial community analysis. (A) Shannon diversity. (B) Weighted Unifrac ordination. (C) Bacterial weighted Unifrac distance from donor centroid. Download [file mbo002162747sf1.pdf]

# Figure S2

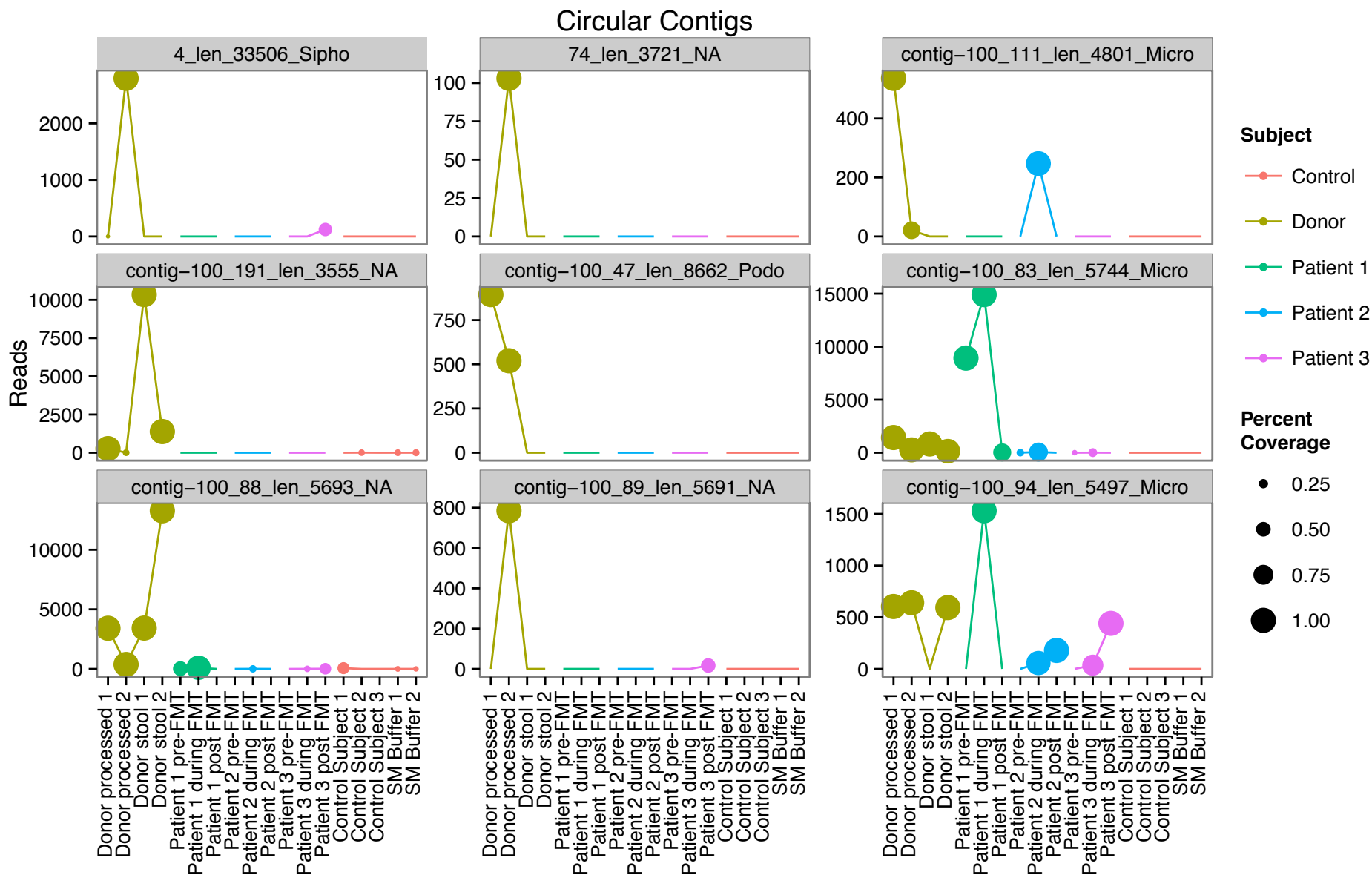

Supplement: Figure S2 — Example of circular contigs. Data represent numbers of reads mapping to each circular donor contig from each of the samples. The size of the circle represents the percentage of coverage of the contig (i.e., a small circle means less of the contig was covered by reads, while a large circle means that the entire contig was covered by reads from that sample). Contig names include lengths and putative family names. Family names were abbreviated as follows: Sipho, Siphoviridae; Micro, Microviridae; Podo, Podoviridae; NA, unattributed. Download [file mbo002162747sf2.pdf]

Figure S3  
Patient 1

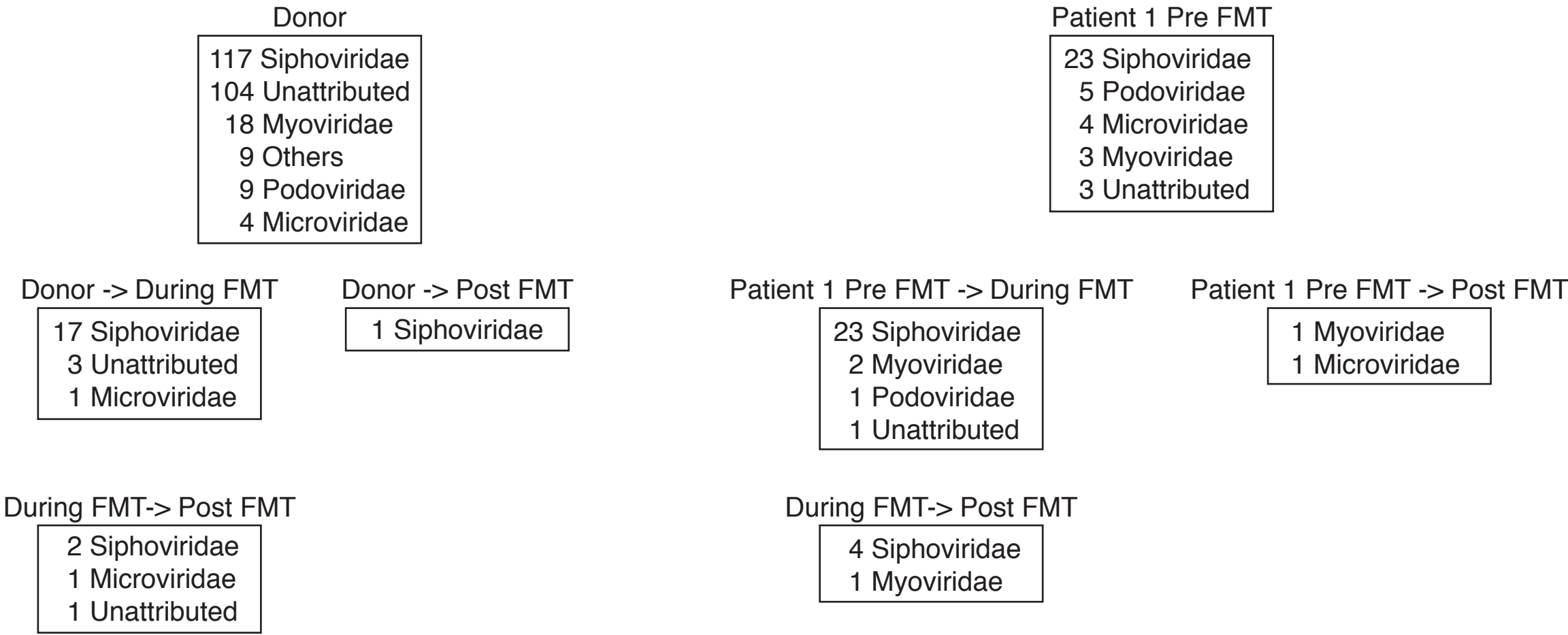

Patient 2

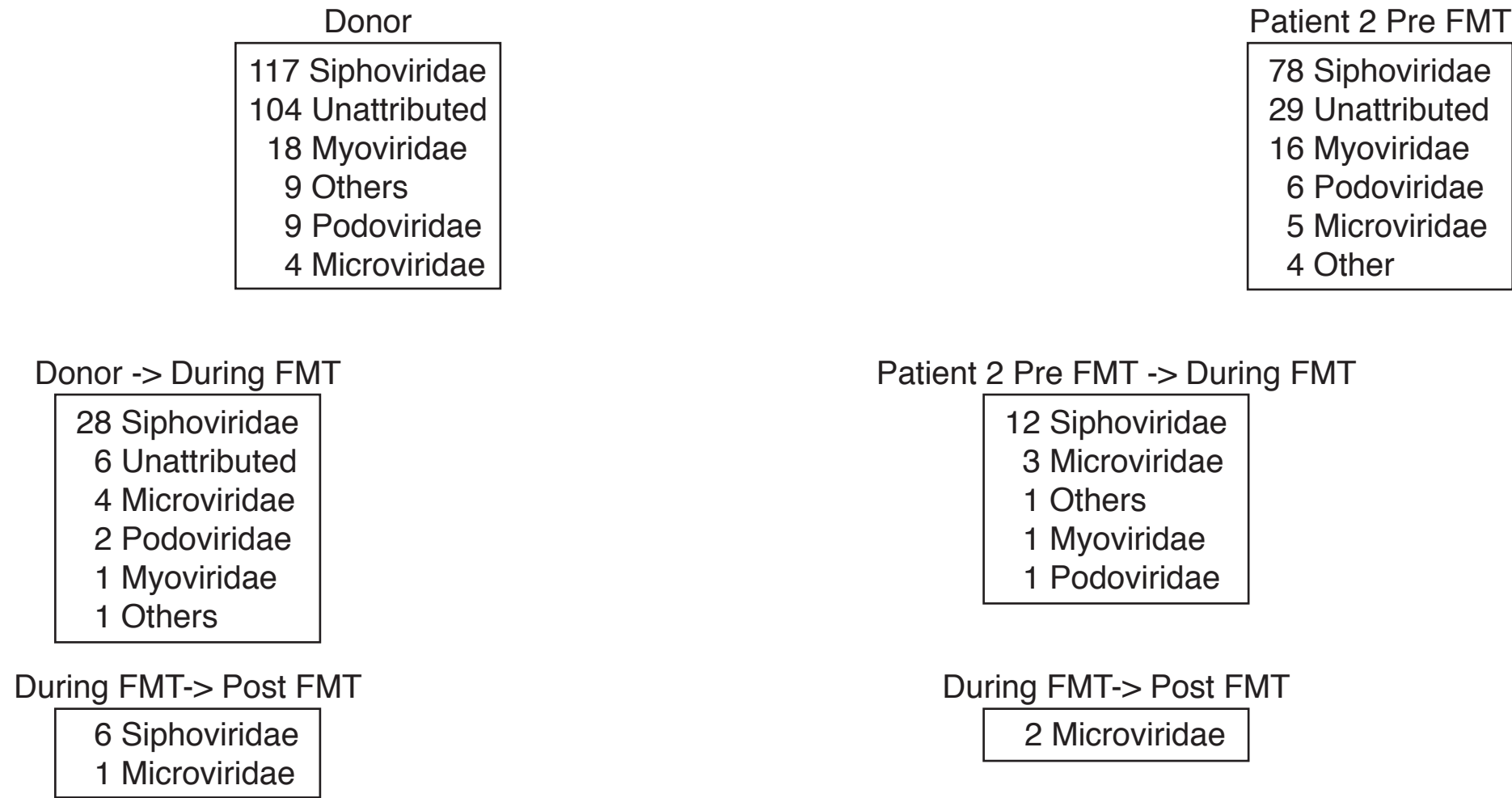

Patient 3

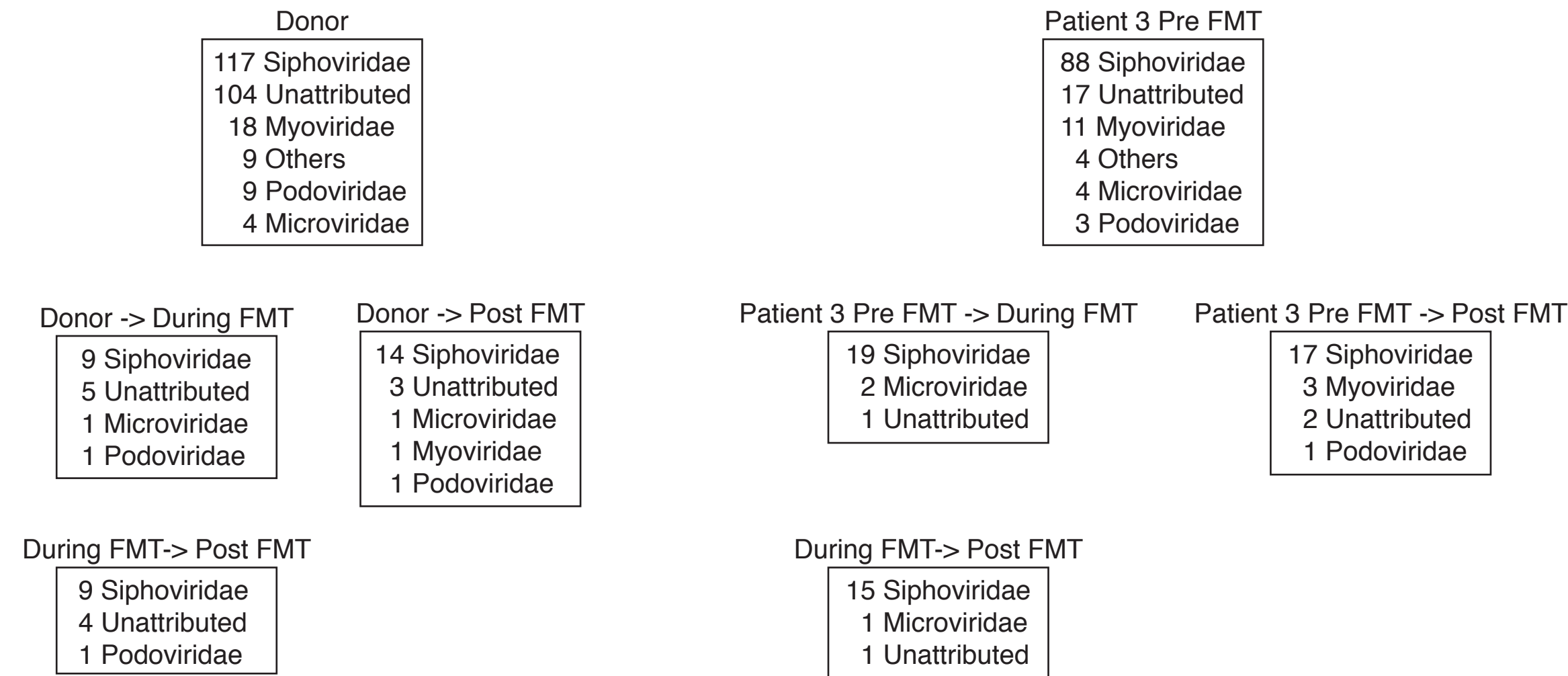

Figure S3: Viral Families of Transferred Contigs

Supplement: Figure S3 — Putative viral families of transferred contigs. For each patient, the number and family name of the contigs that were built from the donor sample and the patient pre-FMT samples are listed. Contigs that transferred from the donor to a patient’s second time point (during FMT), to a patient’s third time point (post-FMT), or to both the second and third time points are listed. Similarly, contigs that transferred from a patient’s first time point (pre-FMT) to subsequent time points are listed. Download [file mbo002162747sf3.pdf]

Figure S4

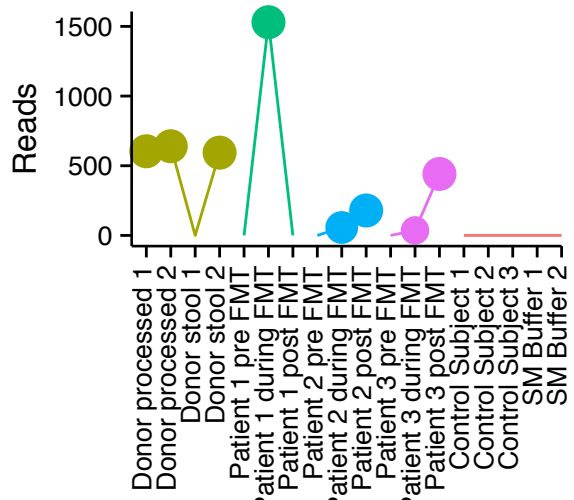

Circular Contig 100-94

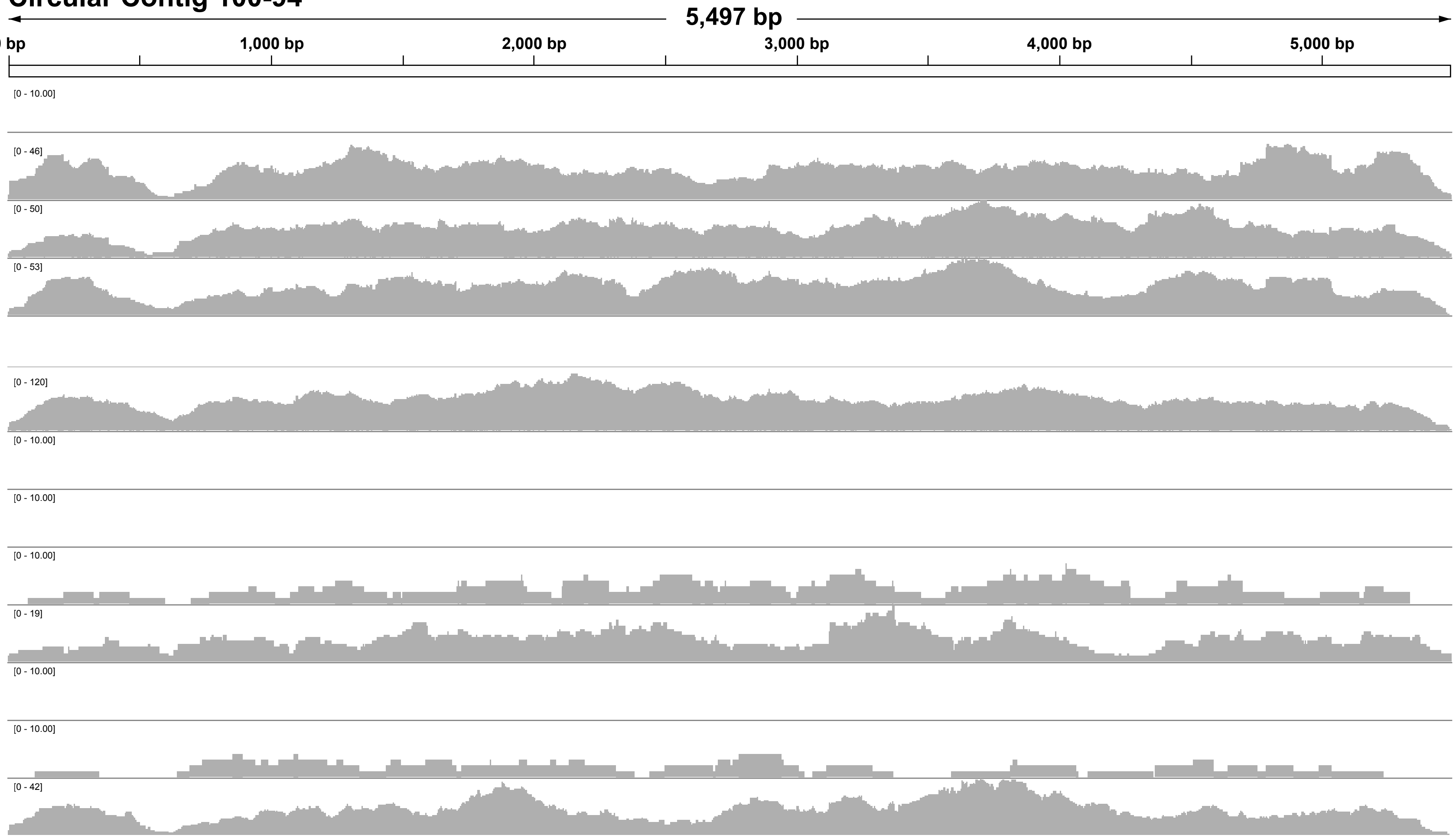

Supplement: Figure S4 — Example of alignment of reads to a contig. The reads from each sample were aligned to circular donor contig 100-94. The reads from each sample that mapped to this contig are shown across the 5,497-bp contig length. The y scale (number of reads) is indicated in the brackets on top of each coverage map. Donor stool 1, patient 1 pre-FMT, patient 1 post-FMT, patient 2 pre-FMT, and patient 3 pre-FMT samples had no reads aligning to this contig. The inset on the top left corner is the same plot as that shown in Fig. S2 and shows the summarized coverage results. Download [file mbo002162747sf4.pdf]
