# Supplementary material for: Transfer of Viral Communities between Human Individuals during Fecal Microbiota Transplantation
Source: mBio. 2016 Mar 29;7(2):e00322-16. doi: 10.1128/mBio.00322-16 (PMC4817255; doi:10.1128/mBio.00322-16)
Supplement: Table S1 — 16S copy number. 16S qPCR was used to detect the quantity of contaminating bacterial DNA in each sample. [file mbo002162747st1.pdf]

Supplementary Table 1. 16S Copy number in the Samples Analyzed.

Sample	16S copy / ng genomified DNA
Donor stool 1	854
Donor stool 2	666
Donor processed 1	199
Donor processed 2	2345
Patient 1 Pre FMT	115
Patient 1 During FMT	68
Patient 1 Post FMT	2425
Patient 2 Pre FMT	2030
Patient 2 During FMT	5021
Patient 2 Post FMT	91
Patient 3 Pre FMT	1138
Patient 3 During FMT	22
Patient 3 Post FMT	4149
No DNA Negative Control	7
Genomified Lambda DNA Control	158
