# Supplementary material for: Transfer of Viral Communities between Human Individuals during Fecal Microbiota Transplantation
Source: mBio. 2016 Mar 29;7(2):e00322-16. doi: 10.1128/mBio.00322-16 (PMC4817255; doi:10.1128/mBio.00322-16)
Supplement: Table S2 — Human reads in the samples analyzed. Data represent the number of reads (~250 bp) sequenced in each sample before and after human filtering. [file mbo002162747st2.pdf]

Supplementary Table 2. Human Reads in the Samples Analyzed.

Sample	Total	Total Paired	Filtered (Non-Human)	Filtered (Non-Human) Paired	Fraction Non-Human
Donor stool 1	1302182	651091	1293112	646556	0.99
Donor stool 2	1186822	593411	1186636	593318	1
Donor processed 1	2033390	1016695	2032440	1016220	1
Donor processed 2	1503192	751596	1502982	751492	1
Patient 1 Pre FMT	2621796	1310898	2621596	1310798	1
Patient 1 During FMT	1471624	735812	1471514	735757	1
Patient 1 Post FMT	1251230	625615	1250750	625375	1
Patient 2 Pre FMT	6542970	3271485	6542024	3271018	1
Patient 2 During FMT	1353574	676787	1353388	676694	1
Patient 2 Post FMT	1530278	765139	1530172	765086	1
Patient 3 Pre FMT	7493706	3746853	7493300	3746650	1
Patient 3 During FMT	1290234	645117	1290134	645068	1
Patient 3 Post FMT	1481912	740956	1481704	740852	1
Control Subject 1	952528	476264	952390	476195	1
Control Subject 2	1315944	657972	1315794	657897	1
Control Subject 3	1987936	993968	1987616	993809	1
Control SM Buffer 1	1282	641	1242	621	0.97
Control SM Buffer 2	37774	18887	36156	18078	0.96
