# Supplementary material for: Transfer of Viral Communities between Human Individuals during Fecal Microbiota Transplantation
Source: mBio. 2016 Mar 29;7(2):e00322-16. doi: 10.1128/mBio.00322-16 (PMC4817255; doi:10.1128/mBio.00322-16)
Supplement: Table S3 — Donor contigs. Data represent information on contigs of >500 bp built from the four donor samples. [file mbo002162747st3.pdf]

Contig Name	Length (bp)	Circular	Number of ORFs	Number of Viral ORFs	Putative Family	Number of Integrase gene matches	Number of Virulence Factor database matches	Number of ACLAME database matches	Top match to NCBI reference viral database	Top match to NCBI nucleotide (nt) database	Top match to NCBI reference bacterial database	Possible host
1	65335	N	28	10	Sipho	2	0	10	NA	Uncultured bacterium clone HA0AAA20ZG01FM1 genomic sequence	gi|150002608|ref|NC_009614.1| Bacteroides vulgatus ATCC 8482 chromosome, complete genome	gi|150002608|ref|NC_009614.1| Bacteroides vulgatus ATCC 8482 chromosome, complete genome
2	40829	N	33	13	Sipho	4	0	14	NA	NA	NA	NA
3	34787	N	23	7	Sipho	4	0	7	NA	NA	NA	NA
4	33506	Y	16	9	Sipho	6	0	10	NA	NA	NA	NA
5	28674	N	16	8	Sipho	1	0	3	NA	Faecalibacterium prausnitzii SL3/3 draft genome	gi|479170689|ref|NC_021020.1| Faecalibacterium prausnitzii SL3/3 draft genome	gi|479170689|ref|NC_021020.1| Faecalibacterium prausnitzii SL3/3 draft genome
6	59556	N	41	12	Sipho	1	1	10	NA	Uncultured bacterium clone HA0AAA2ZB09FM1 genomic sequence	gi|479162165|ref|NC_021017.1| Bacteroides xylanisolvens XB1A draft genome	gi|479162165|ref|NC_021017.1| Bacteroides xylanisolvens XB1A draft genome
7	27213	N	19	11	Sipho	0	2	13	NA	NA	NA	NA
8	27211	N	17	12	Sipho	6	0	12	NA	Clostridiales sp. SSC/2 draft genome	gi|479158859|ref|NC_021016.1| Butyrate-producing bacterium SSC/2, complete genome	gi|479158859|ref|NC_021016.1| Butyrate-producing bacterium SSC/2, complete genome

9	22171	N	11	5	Sipho	0	0	3	NA	Clostridiales sp. SSC/2 draft genome	gi|479158859|ref|NC_021016.1| Butyrate-producing bacterium SSC/2, complete genome	gi|479158859|ref|NC_021016.1| Butyrate-producing bacterium SSC/2, complete genome
10	28482	N	16	10	Sipho	5	0	11	NA	NA	NA	NA
11	21164	N	14	4	Sipho	1	0	5	NA	NA	NA	NA
12	12423	N	11	1	Sipho	0	0	1	NA	NA	NA	NA
13	23900	N	18	1	Sipho	0	0	1	NA	NA	NA	NA
14	17421	N	22	7	Sipho	0	1	8	NA	Unidentified phage clone 2011_scaffold152 genomic sequence	gi|479140210|ref|NC_021010.1| Eubacterium rectale DSM 17629 draft genome	gi|479140210|ref|NC_021010.1| Eubacterium rectale DSM 17629 draft genome
15	17591	N	9	4	Sipho	1	0	5	NA	NA	NA	NA
16	15324	N	11	3	Sipho	2	1	4	NA	Ruminococcus torques L2-14 draft genome	gi|479155735|ref|NC_021015.1| Ruminococcus torques L2-14 draft genome	gi|479155735|ref|NC_021015.1| Ruminococcus torques L2-14 draft genome
17	12601	N	7	2	Podo	0	0	2	NA	NA	NA	NA
18	18368	N	17	9	Sipho	2	1	8	NA	Thermacetogenium phaeum DSM 12270, complete genome	gi|409131816|ref|NC_018870.1| Thermacetogenium phaeum DSM 12270 chromosome, complete genome	gi|409131816|ref|NC_018870.1| Thermacetogenium phaeum DSM 12270 chromosome, complete genome
19	11734	N	8	2	Myo	0	0	2	NA	NA	NA	NA
20	12518	N	14	3	Myo	0	0	6	NA	Unidentified phage clone 2204_scaffold812 genomic sequence	NA	Unidentified phage clone 2204_scaffold 812 genomic sequence
21	16151	N	12	2	Podo	0	0	2	NA	Clostridium difficile chimeric ribozyme CdIST1a-SE918 DNA, tlpB gene for transposase-like protein B, strain SE918	gi|126697566|ref|NC_009089.1| Peptoclostridium difficile 630, complete genome	gi|126697566|ref|NC_009089.1| Peptoclostridium difficile 630, complete genome

22	14516	N	7	4	Sipho	1	0	4	NA	NA	NA	NA
23	18610	N	9	8	Sipho	2	0	8	NA	Streptococcus thermophilus bacteriophage 7201, complete genome	gi|387783149|ref|NC_017595.1| Streptococcus salivarius JIM8777, complete genome	gi|387783149|ref|NC_017595.1| Streptococcus salivarius JIM8777, complete genome
24	17498	N	15	6	Sipho	1	0	6	NA	NA	NA	NA
25	24166	N	13	6	Sipho	1	0	7	NA	NA	NA	NA
26	10363	N	4	3	Sipho	2	0	3	NA	NA	NA	NA
27	7738	N	7	1	Herpes	0	0	0	NA	Bacteroides uniformis strain BUN24 plasmid pBUN24, complete sequence	gi|52421214|ref|NC_006297.1| Bacteroides fragilis YCH46 plasmid pBFY46, complete sequence	gi|52421214|ref|NC_006297.1| Bacteroides fragilis YCH46 plasmid pBFY46, complete sequence
28	9124	N	4	3	Sipho	0	1	4	NA	Haemophilus influenzae 10810 genome	gi|378696079|ref|NC_016809.1| Haemophilus influenzae 10810, complete genome	gi|378696079|ref|NC_016809.1| Haemophilus influenzae 10810, complete genome
29	8702	N	2	1	Myo	0	0	1	NA	NA	NA	NA
30	9151	N	3	2	Sipho	0	0	1	NA	NA	NA	NA
31	25086	N	16	7	Sipho	0	1	8	NA	NA	NA	NA
32	14944	N	14	3	Sipho	0	0	3	NA	Eubacterium rectale ATCC 33656, complete genome	gi|238922432|ref|NC_012781.1| Eubacterium rectale ATCC 33656, complete genome	gi|238922432|ref|NC_012781.1| Eubacterium rectale ATCC 33656, complete genome
33	8752	N	7	4	Sipho	0	0	4	NA	Enterobacteria phage HK629, complete genome	gi|238899406|ref|NC_012759.1| Escherichia coli BW2952 chromosome, complete genome	gi|238899406|ref|NC_012759.1| Escherichia coli BW2952 chromosome, complete genome

34	8601	N	5	5	Sipho	2	1	5	NA	Streptococcus phage 5093, complete genome	gi|221230948|ref|NC_011900.1| Streptococcus pneumoniae ATCC 700669, complete genome	gi|221230948|ref|NC_011900.1| Streptococcus pneumoniae ATCC 700669, complete genome
35	16859	N	11	1	Sipho	0	0	1	NA	Unidentified phage clone 1013_scaffold1563 genomic sequence	NA	Unidentified phage clone 1013_scaffold1563 genomic sequence
36	6788	N	8	2	Sipho	2	0	3	NA	NA	NA	NA
37	22168	N	16	9	Sipho	3	0	11	NA	Staphylococcus prophage phiPV83 proviral DNA, complete sequence	NA	Staphylococcus prophage phiPV83 proviral DNA, complete sequence
38	9907	N	3	3	Myo	2	1	2	NA	Unidentified phage clone 1013_scaffold1563 genomic sequence	NA	Unidentified phage clone 1013_scaffold1563 genomic sequence
39	5646	N	8	2	Sipho	2	0	2	NA	NA	NA	NA
40	8936	N	3	3	Sipho	1	0	3	NA	NA	NA	NA
41	5537	N	3	3	Myo	0	0	3	NA	Enterobacteria phage lambda, complete genome	gi|253771435|ref|NC_012947.1| Escherichia coli 'BL21-Gold(DE3)pLysS AG' chromosome, complete genome	gi|253771435|ref|NC_012947.1| Escherichia coli 'BL21-Gold(DE3)pLysS AG' chromosome, complete genome
42	8830	N	5	5	Sipho	0	0	5	NA	Enterobacteria phage lambda, complete genome	gi|238899406|ref|NC_012759.1| Escherichia coli BW2952 chromosome, complete genome	gi|238899406|ref|NC_012759.1| Escherichia coli BW2952 chromosome, complete genome
43	9925	N	11	4	Sipho	3	0	3	NA	NA	NA	NA

44	9039	N	5	4	Sipho	2	0	4	NA	Lactobacillus casei LOCK919, complete genome	gi|523512490|ref|NC_021721.1| Lactobacillus casei LOCK919, complete genome	gi|523512490|ref|NC_021721.1| Lactobacillus casei LOCK919, complete genome
45	8198	N	8	2	Sipho	1	0	2	NA	Clostridium saccharolyticum WM1, complete genome	gi|302384444|ref|NC_014376.1| Clostridium saccharolyticum WM1 chromosome, complete genome	gi|302384444|ref|NC_014376.1| Clostridium saccharolyticum WM1 chromosome, complete genome
46	14617	N	11	9	Sipho	0	0	9	NA	Ruminococcus torques L2-14 draft genome	gi|479155735|ref|NC_021015.1| Ruminococcus torques L2-14 draft genome	gi|479155735|ref|NC_021015.1| Ruminococcus torques L2-14 draft genome
47	7518	N	5	3	Sipho	1	0	3	NA	NA	NA	NA
48	5304	N	5	4	Myo	0	0	4	NA	NA	NA	NA
49	11243	N	8	3	Sipho	0	0	3	NA	NA	NA	NA
50	6624	N	8	0	NA	0	1	0	NA	Uncultured bacterium clone 20WL_B240P7D10 putative beta-glucuronidase (BG) gene, partial cds	gi|479170689|ref|NC_021020.1| Faecalibacterium prausnitzii SL3/3 draft genome	gi|479170689|ref|NC_021020.1| Faecalibacterium prausnitzii SL3/3 draft genome
51	7485	N	5	5	Sipho	0	0	4	NA	Clostridiales sp. SM4/1 draft genome	gi|479181986|ref|NC_021024.1| Butyrate-producing bacterium SM4/1, complete genome	gi|479181986|ref|NC_021024.1| Butyrate-producing bacterium SM4/1, complete genome
52	5801	N	4	1	NA	0	0	1	NA	NA	NA	NA
53	5362	N	6	0	NA	0	0	0	NA	Uncultured organism clone VC1AJ77TF genomic sequence	NA	Uncultured organism clone VC1AJ77TF genomic sequence

54	4951	N	2	0	NA	0	0	1	NA	Haemophilus parainfluenzae T3T1 complete genome	gi|345428590|ref|NC_015964.1| Haemophilus parainfluenzae T3T1, complete genome	gi|345428590|ref|NC_015964.1| Haemophilus parainfluenzae T3T1, complete genome
55	5803	N	4	4	Sipho	2	0	4	NA	Streptococcus infantarius subsp. infantarius CJ18, complete genome	gi|379704392|ref|NC_016826.1| Streptococcus infantarius subsp. infantarius CJ18 chromosome, complete genome	gi|379704392|ref|NC_016826.1| Streptococcus infantarius subsp. infantarius CJ18 chromosome, complete genome
56	5242	N	5	1	Myo	0	1	2	NA	Propionibacterium acnes KPA171202, complete genome	gi|50841496|ref|NC_006085.1| Propionibacterium acnes KPA171202 chromosome, complete genome	gi|50841496|ref|NC_006085.1| Propionibacterium acnes KPA171202 chromosome, complete genome
57	6714	N	5	4	Sipho	1	0	4	NA	NA	NA	NA
58	7528	N	5	2	Sipho	1	0	2	NA	Clostridiales sp. SS3/4 draft genome	gi|479192860|ref|NC_021035.1| Butyrate-producing bacterium SS3/4, complete genome	gi|479192860|ref|NC_021035.1| Butyrate-producing bacterium SS3/4, complete genome
59	8815	N	5	2	Sipho	1	0	3	NA	NA	NA	NA
60	6837	N	3	2	Sipho	0	0	2	NA	NA	NA	NA
61	10259	N	6	4	Sipho	0	0	1	NA	Clostridiales sp. SM4/1 draft genome	gi|479181986|ref|NC_021024.1| Butyrate-producing bacterium SM4/1, complete genome	gi|479181986|ref|NC_021024.1| Butyrate-producing bacterium SM4/1, complete genome
62	6565	N	5	4	Myo	0	0	1	NA	NA	NA	NA

63	5138	N	3	0	NA	0	0	0	NA	Uncultured organism clone 1041059765643 genomic sequence	NA	Uncultured organism clone 1041059765643 genomic sequence
64	7736	N	9	2	Sipho	1	0	4	NA	NA	NA	NA
65	4571	N	3	1	Sipho	0	0	3	NA	NA	NA	NA
66	10625	N	6	1	Sipho	1	0	1	NA	NA	NA	NA
67	6169	N	4	3	Sipho	0	0	4	NA	Enterobacteria phage HK629, complete genome	gi|253771435|ref|NC_012947.1| Escherichia coli 'BL21-Gold(DE3)pLysS AG' chromosome, complete genome	gi|253771435|ref|NC_012947.1| Escherichia coli 'BL21-Gold(DE3)pLysS AG' chromosome, complete genome
68	8993	N	6	1	Sipho	1	0	1	NA	NA	NA	NA
69	5461	N	3	0	NA	0	0	1	NA	Haemophilus parainfluenzae T3T1 complete genome	gi|345428590|ref|NC_015964.1| Haemophilus parainfluenzae T3T1, complete genome	gi|345428590|ref|NC_015964.1| Haemophilus parainfluenzae T3T1, complete genome
70	5354	N	3	2	Sipho	1	0	3	NA	NA	NA	NA
71	5127	N	6	0	NA	1	0	0	NA	NA	NA	NA
72	5264	N	5	0	NA	0	0	0	NA	Faecalibacterium prausnitzii SL3/3 draft genome	gi|479170689|ref|NC_021020.1| Faecalibacterium prausnitzii SL3/3 draft genome	gi|479170689|ref|NC_021020.1| Faecalibacterium prausnitzii SL3/3 draft genome
73	10072	N	5	0	NA	0	3	1	NA	NA	gi|563711419|ref|NC_023004.1| Candidatus Saccharibacteria bacterium RAAC3_TM7_1, complete genome	gi|563711419|ref|NC_023004.1| Candidatus Saccharibacteria bacterium RAAC3_TM7_1, complete genome

74	3721	Y	5	0	NA	0	0	0	NA	Bifidobacterium longum subsp. longum F8 draft genome	gi|479134917|ref|NC_021008.1| Bifidobacterium longum subsp. longum F8 draft genome	gi|479134917|ref|NC_021008.1| Bifidobacterium longum subsp. longum F8 draft genome
75	11103	N	9	2	Pox	0	0	1	NA	Escherichia coli str. K-12 substr. MG1655, complete genome	gi|386703215|ref|NC_017663.1| Escherichia coli P12b chromosome, complete genome	gi|386703215|ref|NC_017663.1| Escherichia coli P12b chromosome, complete genome
76	4745	N	5	1	Myo	0	1	1	NA	NA	NA	NA
77	4286	N	2	0	NA	0	0	0	NA	NA	NA	NA
78	5254	N	4	1	Sipho	0	1	1	NA	NA	NA	NA
79	4221	N	2	1	Sipho	0	0	1	NA	NA	NA	NA
80	5857	N	6	2	Sipho	0	0	2	NA	NA	NA	NA
81	4578	N	2	0	NA	0	0	1	NA	NA	NA	NA
82	3334	N	1	0	NA	0	0	0	NA	Roseburia intestinalis M50/1 draft genome	gi|479201824|ref|NC_021040.1| Roseburia intestinalis M50/1 draft genome	gi|479201824|ref|NC_021040.1| Roseburia intestinalis M50/1 draft genome
83	10414	N	10	4	Sipho	0	0	4	NA	NA	NA	NA
84	4678	N	3	2	Sipho	0	1	2	NA	Eubacterium limosum KIST612, complete genome	gi|365823354|ref|NC_014624.2| Eubacterium limosum KIST612 chromosome, complete genome	gi|365823354|ref|NC_014624.2| Eubacterium limosum KIST612 chromosome, complete genome
85	4531	N	4	0	NA	0	0	1	NA	NA	NA	NA
86	6823	N	6	1	Sipho	0	0	1	NA	NA	NA	NA
87	4621	N	2	0	NA	0	0	0	NA	Haemophilus parainfluenzae T3T1 complete genome	gi|345428590|ref|NC_015964.1| Haemophilus parainfluenzae T3T1, complete genome	gi|345428590|ref|NC_015964.1| Haemophilus parainfluenzae T3T1, complete genome
88	4133	N	4	2	Micro	0	0	2	NA	NA	NA	NA

89	3349	N	2	2	Myo	0	1	2	NA	Clostridiales sp. SSC/2 draft genome	gi|479158859|ref|NC_021016.1| Butyrate-producing bacterium SSC/2, complete genome	gi|479158859|ref|NC_021016.1| Butyrate-producing bacterium SSC/2, complete genome
90	4737	N	2	0	NA	0	0	0	NA	NA	NA	NA
91	3934	N	3	1	Sipho	1	0	1	NA	Unidentified phage clone 2019_scaffold132 genomic sequence	NA	Unidentified phage clone 2019_scaffold132 genomic sequence
92	5300	N	5	2	Sipho	1	0	2	NA	NA	NA	NA
93	3692	N	4	2	Myo	1	0	2	NA	NA	NA	NA
94	5827	N	3	0	NA	0	1	1	NA	Bacteroides thetaiotaomicron VPI-5482, complete genome	gi|29345410|ref|NC_004663.1| Bacteroides thetaiotaomicron VPI-5482 chromosome, complete genome	gi|29345410|ref|NC_004663.1| Bacteroides thetaiotaomicron VPI-5482 chromosome, complete genome
95	7468	N	6	1	NA	0	1	0	NA	Bacteroides salanitronis DSM 18170, complete genome	gi|325297172|ref|NC_015164.1| Bacteroides salanitronis DSM 18170 chromosome, complete genome	gi|325297172|ref|NC_015164.1| Bacteroides salanitronis DSM 18170 chromosome, complete genome
96	4894	N	4	0	NA	0	0	1	NA	Rhodobacter sphaeroides ATCC 17025 plasmid pRSPA01, complete sequence	gi|146279170|ref|NC_009429.1| Rhodobacter sphaeroides ATCC 17025 plasmid pRSPA01, complete sequence	gi|146279170|ref|NC_009429.1| Rhodobacter sphaeroides ATCC 17025 plasmid pRSPA01, complete sequence
97	3928	N	2	1	Sipho	0	1	1	NA	Uncultured organism clone VC1C565TR genomic sequence	NA	Uncultured organism clone VC1C565TR genomic sequence
98	4071	N	4	0	NA	0	0	0	NA	NA	NA	NA

99	10681	N	5	3	Sipho	1	0	3	NA	NA	NA	NA
100	5376	N	2	1	Sipho	1	0	1	NA	NA	NA	NA
101	2957	N	2	0	NA	0	0	0	NA	NA	NA	NA
102	7469	N	5	2	Herpes	1	3	1	NA	Streptococcus thermophilus ND03, complete genome	gi|386085705|ref|NC_017563.1| Streptococcus thermophilus ND03 chromosome, complete genome	gi|386085705|ref|NC_017563.1| Streptococcus thermophilus ND03 chromosome, complete genome
103	5235	N	2	2	NA	0	0	1	NA	Clostridiales sp. SS3/4 draft genome	gi|479192860|ref|NC_021035.1| Butyrate-producing bacterium SS3/4, complete genome	gi|479192860|ref|NC_021035.1| Butyrate-producing bacterium SS3/4, complete genome
104	4620	N	4	0	NA	0	0	1	NA	Faecalibacterium prausnitzii SL3/3 draft genome	gi|479170689|ref|NC_021020.1| Faecalibacterium prausnitzii SL3/3 draft genome	gi|479170689|ref|NC_021020.1| Faecalibacterium prausnitzii SL3/3 draft genome
105	3852	N	4	0	NA	0	0	1	NA	Haemophilus parainfluenzae T3T1 complete genome	gi|345428590|ref|NC_015964.1| Haemophilus parainfluenzae T3T1, complete genome	gi|345428590|ref|NC_015964.1| Haemophilus parainfluenzae T3T1, complete genome
106	3866	N	1	0	NA	0	0	1	NA	NA	NA	NA
107	2652	N	2	1	Sipho	0	0	1	NA	NA	NA	NA
108	3561	N	3	2	Sipho	0	0	2	NA	NA	NA	NA
109	6796	N	6	0	NA	0	0	0	NA	Unidentified phage clone 1013_scaffold1563 genomic sequence	NA	Unidentified phage clone 1013_scaffold1563 genomic sequence

110	5596	N	3	0	NA	0	1	0	NA	Haemophilus parainfluenzae T3T1 complete genome	gi|345428590|ref|NC_015964.1| Haemophilus parainfluenzae T3T1, complete genome	gi|345428590|ref|NC_015964.1| Haemophilus parainfluenzae T3T1, complete genome
111	5205	N	3	3	Sipho	1	0	3	NA	NA	NA	NA
112	2611	N	2	1	Gemini	0	0	0	NA	NA	NA	NA
113	3261	N	2	0	NA	0	1	1	NA	Uncultured bacterium clone LM0ABA39ZD09RM1 genomic sequence	NA	Uncultured bacterium clone LM0ABA39ZD09RM1 genomic sequence
114	2341	N	3	1	Myo	0	0	2	NA	Faecalibacterium prausnitzii L2/6 draft genome	gi|479208076|ref|NC_021042.1| Faecalibacterium prausnitzii L2-6, complete genome	gi|479208076|ref|NC_021042.1| Faecalibacterium prausnitzii L2-6, complete genome
115	5844	N	6	2	Sipho	0	0	2	NA	NA	NA	NA
116	2439	N	2	1	Sipho	0	0	1	NA	Streptococcus phage 040922	gi|307126151|ref|NC_014498.1| Streptococcus pneumoniae 670-6B chromosome, complete genome	gi|307126151|ref|NC_014498.1| Streptococcus pneumoniae 670-6B chromosome, complete genome
117	2375	N	2	0	NA	0	0	0	NA	NA	NA	NA
118	2856	N	3	1	Pox	0	2	1	NA	Uncultured organism clone 7 genomic sequence	gi|260685375|ref|NC_013316.1| Clostridium difficile R20291 chromosome, complete genome	gi|260685375|ref|NC_013316.1| Clostridium difficile R20291 chromosome, complete genome
119	3770	N	2	1	Sipho	0	0	1	NA	NA	NA	NA
120	2352	N	1	1	Sipho	1	0	1	NA	NA	NA	NA
121	4191	N	4	0	NA	0	0	0	NA	NA	NA	NA
122	3211	N	2	0	NA	0	0	0	NA	NA	NA	NA

123	2293	N	1	0	NA	0	0	0	NA	Streptococcus phage V22	NA	Streptococcus phage V22
124	2218	N	1	0	NA	0	0	0	NA	NA	NA	NA
125	1978	N	1	0	NA	0	0	0	NA	TPA_exp: Clostridium difficile strain R20291 transposon Tn6103, complete sequence	gi|260685375|ref|NC_013316.1| Clostridium difficile R20291 chromosome, complete genome	gi|260685375|ref|NC_013316.1| Clostridium difficile R20291 chromosome, complete genome
126	2158	N	1	0	NA	0	0	0	NA	NA	NA	NA
127	2050	N	3	0	NA	0	0	0	NA	NA	NA	NA
128	3720	N	2	2	Sipho	0	0	2	NA	NA	NA	NA
129	2265	N	2	0	NA	0	0	0	NA	Coccomyxa sp. SAG 20.40 23S ribosomal RNA gene, partial sequence; and putative LAGLIDADG homing endonuclease gene, complete cds; chloroplast	NA	Coccomyxa sp. SAG 20.40 23S ribosomal RNA gene, partial sequence; and putative LAGLIDADG homing endonuclease gene, complete cds; chloroplast
130	2359	N	1	1	Sipho	0	0	1	NA	NA	NA	NA
131	4689	N	3	2	Sipho	1	0	2	NA	Streptococcus mitis phage SM1,complete genome	gi|405759923|ref|NC_018594.1| Streptococcus pneumoniae SPNA45, complete genome	gi|405759923|ref|NC_018594.1| Streptococcus pneumoniae SPNA45, complete genome
132	3781	N	4	2	Myo	0	0	2	NA	Haemophilus parainfluenzae T3T1 complete genome	gi|345428590|ref|NC_015964.1| Haemophilus parainfluenzae T3T1, complete genome	gi|345428590|ref|NC_015964.1| Haemophilus parainfluenzae T3T1, complete genome

133	2192	N	2	0	NA	0	0	0	NA	Veillonella parvula DSM 2008, complete genome	gi|269797069|ref|NC_013520.1| Veillonella parvula DSM 2008 chromosome, complete genome	gi|269797069|ref|NC_013520.1| Veillonella parvula DSM 2008 chromosome, complete genome
134	2882	N	2	1	Sipho	0	0	1	NA	NA	NA	NA
135	2830	N	1	0	NA	0	0	0	NA	NA	NA	NA
136	2613	N	1	1	Sipho	0	0	1	NA	NA	NA	NA
137	3743	N	3	0	NA	0	0	0	NA	NA	NA	NA
138	2680	N	4	1	Sipho	0	0	1	NA	NA	NA	NA
139	2212	N	2	0	NA	0	0	1	NA	NA	NA	NA
140	2518	N	2	0	NA	0	0	0	NA	Propionibacterium acnes ATCC 11828, complete genome	gi|386069650|ref|NC_017550.1| Propionibacterium acnes ATCC 11828 chromosome, complete genome	gi|386069650|ref|NC_017550.1| Propionibacterium acnes ATCC 11828 chromosome, complete genome
141	2238	N	1	0	NA	0	0	0	NA	Faecalibacterium prausnitzii L2/6 draft genome	gi|479208076|ref|NC_021042.1| Faecalibacterium prausnitzii L2-6, complete genome	gi|479208076|ref|NC_021042.1| Faecalibacterium prausnitzii L2-6, complete genome
142	2528	N	3	0	NA	0	0	0	NA	NA	NA	NA
143	2350	N	3	0	NA	0	0	0	NA	NA	NA	NA
144	3188	N	4	1	NA	0	0	0	NA	Faecalibacterium prausnitzii SL3/3 draft genome	gi|479170689|ref|NC_021020.1| Faecalibacterium prausnitzii SL3/3 draft genome	gi|479170689|ref|NC_021020.1| Faecalibacterium prausnitzii SL3/3 draft genome
145	2662	N	2	0	NA	0	0	0	NA	NA	NA	NA
146	3139	N	2	0	NA	0	0	0	NA	NA	NA	NA

147	4861	N	2	1	NA	0	0	1	NA	Haemophilus parainfluenzae T3T1 complete genome	gi|345428590|ref|NC_015964.1| Haemophilus parainfluenzae T3T1, complete genome	gi|345428590|ref|NC_015964.1| Haemophilus parainfluenzae T3T1, complete genome
148	3338	N	3	0	NA	0	0	0	NA	NA	NA	NA
149	2736	N	0	0	NA	0	0	0	NA	NA	NA	NA
150	2646	N	1	0	NA	0	0	0	NA	NA	NA	NA
151	2559	N	0	0	NA	0	0	0	NA	NA	NA	NA
152	3697	N	1	0	NA	0	0	0	NA	NA	NA	NA
153	2505	N	1	0	NA	0	0	0	NA	Bacteroides fragilis YCH46 DNA, complete genome	gi|53711291|ref|NC_006347.1| Bacteroides fragilis YCH46 DNA, complete genome	gi|53711291|ref|NC_006347.1| Bacteroides fragilis YCH46 DNA, complete genome
154	6560	N	5	0	NA	0	0	0	NA	NA	NA	NA
155	3209	N	1	0	NA	0	0	0	NA	NA	NA	NA
156	1911	N	2	0	NA	0	0	0	NA	Bacteroides fragilis YCH46 DNA, complete genome	gi|53711291|ref|NC_006347.1| Bacteroides fragilis YCH46 DNA, complete genome	gi|53711291|ref|NC_006347.1| Bacteroides fragilis YCH46 DNA, complete genome
157	1784	N	1	0	NA	0	0	0	NA	Bacteroides xylanisolvens XB1A draft genome	gi|479162165|ref|NC_021017.1| Bacteroides xylanisolvens XB1A draft genome	gi|479162165|ref|NC_021017.1| Bacteroides xylanisolvens XB1A draft genome
158	3910	N	2	0	NA	0	0	0	NA	Uncultured organism clone 1041059765645 genomic sequence	gi|319899888|ref|NC_014933.1| Bacteroides helcogenes P 36-108 chromosome, complete genome	gi|319899888|ref|NC_014933.1| Bacteroides helcogenes P 36-108 chromosome, complete genome
159	2810	N	1	0	NA	0	0	0	NA	NA	NA	NA
160	2645	N	2	0	NA	0	0	0	NA	NA	NA	NA

161	3434	N	2	0	NA	0	0	0	NA	Uncultured organism clone 1041059766731 genomic sequence	gi|319899888|ref|NC_014933.1| Bacteroides helcogenes P 36-108 chromosome, complete genome	gi|319899888|ref|NC_014933.1| Bacteroides helcogenes P 36-108 chromosome, complete genome
162	2349	N	2	0	NA	0	0	1	NA	NA	NA	NA
163	2431	N	2	1	Sipho	1	0	1	NA	NA	NA	NA
164	4171	N	4	3	Sipho	2	0	3	NA	NA	NA	NA
165	2851	N	2	0	NA	0	0	0	NA	Bacteroides salanitronis DSM 18170, complete genome	gi|325297172|ref|NC_015164.1| Bacteroides salanitronis DSM 18170 chromosome, complete genome	gi|325297172|ref|NC_015164.1| Bacteroides salanitronis DSM 18170 chromosome, complete genome
166	2251	N	2	0	NA	0	0	0	NA	Eubacterium rectale DSM 17629 draft genome	gi|479140210|ref|NC_021010.1| Eubacterium rectale DSM 17629 draft genome	gi|479140210|ref|NC_021010.1| Eubacterium rectale DSM 17629 draft genome
167	3653	N	2	1	Sipho	0	0	1	NA	Streptococcus pyogenes SSI-1 DNA, complete genome	gi|28894912|ref|NC_004606.1| Streptococcus pyogenes SSI-1 chromosome, complete genome	gi|28894912|ref|NC_004606.1| Streptococcus pyogenes SSI-1 chromosome, complete genome
168	3115	N	2	0	NA	0	0	0	NA	Eubacterium rectale M104/1 draft genome	gi|479213596|ref|NC_021044.1| Eubacterium rectale M104/1 draft genome	gi|479213596|ref|NC_021044.1| Eubacterium rectale M104/1 draft genome

169	3047	N	3	0	NA	0	0	0	NA	Escherichia coli str. K-12 substr. MG1655, complete genome	gi|388476123|ref|NC_007779.1| Escherichia coli str. K-12 substr. W3110, complete genome	gi|388476123|ref|NC_007779.1| Escherichia coli str. K-12 substr. W3110, complete genome
170	2024	N	1	1	Sipho	1	0	1	NA	NA	NA	NA
171	2580	N	1	0	NA	0	0	0	NA	NA	NA	NA
172	2269	N	4	0	NA	0	0	0	NA	Uncultured organism clone VC1BZ08TR genomic sequence	gi|479170689|ref|NC_021020.1| Faecalibacterium prausnitzii SL3/3 draft genome	gi|479170689|ref|NC_021020.1| Faecalibacterium prausnitzii SL3/3 draft genome
173	2313	N	1	0	NA	0	1	0	NA	Haemophilus parainfluenzae T3T1 complete genome	gi|345428590|ref|NC_015964.1| Haemophilus parainfluenzae T3T1, complete genome	gi|345428590|ref|NC_015964.1| Haemophilus parainfluenzae T3T1, complete genome
174	3666	N	3	1	Phycodna	0	1	0	NA	Roseburia hominis A2-183, complete genome	gi|347530298|ref|NC_015977.1| Roseburia hominis A2-183 chromosome, complete genome	gi|347530298|ref|NC_015977.1| Roseburia hominis A2-183 chromosome, complete genome
175	1577	N	1	0	NA	0	0	0	NA	NA	NA	NA
176	3211	N	2	0	NA	0	0	1	NA	Haemophilus influenzae KR494, complete genome	gi|543951066|ref|NC_022356.1| Haemophilus influenzae KR494, complete genome	gi|543951066|ref|NC_022356.1| Haemophilus influenzae KR494, complete genome
177	2377	N	4	2	Sipho	1	0	2	NA	NA	NA	NA

178	5237	N	2	2	Sipho	1	0	2	NA	Enterobacteria phage lambda, complete genome	gi|253771435|ref|NC_012947.1| Escherichia coli 'BL21-Gold(DE3)pLysS AG' chromosome, complete genome	gi|253771435|ref|NC_012947.1| Escherichia coli 'BL21-Gold(DE3)pLysS AG' chromosome, complete genome
179	2805	N	2	0	NA	0	0	0	NA	NA	NA	NA
180	2920	N	2	2	Sipho	1	0	2	NA	NA	NA	NA
181	1868	N	2	2	Myo	0	1	2	NA	NA	NA	NA
182	1840	N	1	0	NA	0	0	0	NA	Eubacterium rectale M104/1 draft genome	gi|479213596|ref|NC_021044.1| Eubacterium rectale M104/1 draft genome	gi|479213596|ref|NC_021044.1| Eubacterium rectale M104/1 draft genome
183	3275	N	2	0	NA	0	0	0	NA	Ruminococcus sp. SR1/5 draft genome	gi|479152295|ref|NC_021014.1| Ruminococcus sp. SR1/5 draft genome	gi|479152295|ref|NC_021014.1| Ruminococcus sp. SR1/5 draft genome
184	1832	N	0	0	NA	0	0	0	NA	NA	NA	NA
185	2390	N	1	1	Sipho	0	0	1	NA	NA	NA	NA
186	2353	N	2	0	NA	0	0	1	NA	Roseburia intestinalis M50/1 draft genome	gi|479201824|ref|NC_021040.1| Roseburia intestinalis M50/1 draft genome	gi|479201824|ref|NC_021040.1| Roseburia intestinalis M50/1 draft genome
187	2428	N	1	0	NA	0	0	0	NA	Unidentified phage clone 1013_scaffold1563 genomic sequence	NA	Unidentified phage clone 1013_scaffold1563 genomic sequence
188	1831	N	0	0	NA	0	0	0	NA	Parabacteroides distasonis ATCC 8503, complete genome	gi|150006674|ref|NC_009615.1| Parabacteroides distasonis ATCC 8503 chromosome, complete genome	gi|150006674|ref|NC_009615.1| Parabacteroides distasonis ATCC 8503 chromosome, complete genome
189	2269	N	2	1	Plasma	0	0	1	NA	NA	NA	NA
190	3300	N	5	0	NA	0	0	0	NA	NA	NA	NA

191	1969	N	0	0	NA	0	0	0	NA	Bacillus cereus E33L, complete genome	gi|52140164|ref|NC_006274.1| Bacillus cereus E33L chromosome, complete genome	gi|52140164|ref|NC_006274.1| Bacillus cereus E33L chromosome, complete genome
192	2806	N	1	0	NA	0	0	0	NA	NA	NA	NA
193	3106	N	2	1	NA	0	0	0	NA	NA	NA	NA
194	1949	N	1	0	NA	0	0	0	NA	Haemophilus parainfluenzae T3T1 complete genome	gi|345428590|ref|NC_015964.1| Haemophilus parainfluenzae T3T1, complete genome	gi|345428590|ref|NC_015964.1| Haemophilus parainfluenzae T3T1, complete genome
195	1966	N	1	0	NA	0	0	0	NA	NA	NA	NA
196	2946	N	2	0	NA	0	0	0	NA	Haemophilus parainfluenzae T3T1 complete genome	gi|345428590|ref|NC_015964.1| Haemophilus parainfluenzae T3T1, complete genome	gi|345428590|ref|NC_015964.1| Haemophilus parainfluenzae T3T1, complete genome
197	1498	N	1	0	NA	0	0	0	NA	Bacteroides fragilis plasmid pBFUK1 DNA, complete genome, strain: GAI92082	NA	Bacteroides fragilis plasmid pBFUK1 DNA, complete genome, strain: GAI92082
198	1463	N	1	0	NA	0	0	0	NA	NA	NA	NA
199	1918	N	0	0	NA	0	0	0	NA	NA	NA	NA
200	2512	N	3	1	Micro	0	0	1	NA	NA	NA	NA
201	2372	N	2	0	NA	0	0	1	NA	Unidentified phage clone 1013_scaffold1563 genomic sequence	NA	Unidentified phage clone 1013_scaffold1563 genomic sequence
202	1469	N	1	0	NA	0	0	0	NA	NA	NA	NA
203	2196	N	1	0	NA	0	0	0	NA	NA	NA	NA

204	2300	N	3	0	NA	0	0	0	NA	Bacteroides fragilis strain IB143 plasmid pBI143, complete sequence	gi|150006674|ref|NC_009615.1| Parabacteroides distasonis ATCC 8503 chromosome, complete genome	gi|150006674|ref|NC_009615.1| Parabacteroides distasonis ATCC 8503 chromosome, complete genome
205	1469	N	0	0	NA	0	0	0	NA	NA	NA	NA
206	1499	N	1	0	NA	0	0	0	NA	NA	NA	NA
207	1454	N	1	0	NA	0	0	0	NA	Uncultured organism clone 1041059767276 genomic sequence	NA	Uncultured organism clone 1041059767276 genomic sequence
208	1917	N	2	0	NA	0	0	1	NA	NA	NA	NA
209	2438	N	1	0	NA	0	0	0	NA	NA	NA	NA
210	1329	N	1	0	NA	0	0	0	NA	NA	NA	NA
211	1422	N	1	1	Sipho	0	0	1	NA	NA	NA	NA
212	1193	N	1	0	NA	0	0	0	NA	Unidentified phage clone 2019_scaffold132 genomic sequence	NA	Unidentified phage clone 2019_scaffold132 genomic sequence
213	1316	N	1	0	NA	0	0	0	NA	NA	NA	NA
214	1609	N	1	0	NA	0	0	0	NA	NA	NA	NA
215	2585	N	3	2	Podo	1	0	2	NA	NA	NA	NA
216	1734	N	1	1	Sipho	0	0	1	NA	NA	NA	NA
217	1751	N	1	0	NA	0	0	0	NA	Haemophilus parainfluenzae T3T1 complete genome	gi|345428590|ref|NC_015964.1| Haemophilus parainfluenzae T3T1, complete genome	gi|345428590|ref|NC_015964.1| Haemophilus parainfluenzae T3T1, complete genome
218	1458	N	1	0	NA	0	0	0	NA	Uncultured organism clone VC1AM05TF genomic sequence	NA	Uncultured organism clone VC1AM05TF genomic sequence

219	1911	N	2	0	NA	0	0	0	NA	Uncultured bacterium xylooligosaccharide degrading DNA fragment, clone MetaPbio 5	gi|325297172|ref|NC_015164.1| Bacteroides salanitronis DSM 18170 chromosome, complete genome	gi|325297172|ref|NC_015164.1| Bacteroides salanitronis DSM 18170 chromosome, complete genome
220	2049	N	0	0	NA	0	0	0	NA	NA	NA	NA
221	1526	N	1	0	NA	0	0	0	NA	Haemophilus parainfluenzae T3T1 complete genome	gi|345428590|ref|NC_015964.1| Haemophilus parainfluenzae T3T1, complete genome	gi|345428590|ref|NC_015964.1| Haemophilus parainfluenzae T3T1, complete genome
222	2227	N	0	0	NA	0	0	0	NA	NA	NA	NA
223	1203	N	1	0	NA	0	0	0	NA	Escherichia coli str. K-12 substr. MG1655, complete genome	gi|386612163|ref|NC_017641.1| Escherichia coli UMNK88 chromosome, complete genome	gi|386612163|ref|NC_017641.1| Escherichia coli UMNK88 chromosome, complete genome
224	1297	N	0	0	NA	0	0	0	NA	NA	NA	NA
225	1957	N	1	1	Sipho	0	0	1	NA	Streptococcus phage 858, complete genome	gi|387783149|ref|NC_017595.1| Streptococcus salivarius JIM8777, complete genome	gi|387783149|ref|NC_017595.1| Streptococcus salivarius JIM8777, complete genome
226	2371	N	1	0	NA	0	0	0	NA	NA	NA	NA
227	1229	N	2	0	NA	0	0	0	NA	NA	NA	NA
228	1799	N	0	0	NA	0	0	0	NA	Haemophilus parainfluenzae T3T1 complete genome	gi|345428590|ref|NC_015964.1| Haemophilus parainfluenzae T3T1, complete genome	gi|345428590|ref|NC_015964.1| Haemophilus parainfluenzae T3T1, complete genome
229	1295	N	2	0	NA	0	0	0	NA	NA	NA	NA

230	2451	N	0	0	NA	0	0	0	NA	Bacteroides vulgatus ATCC 8482, complete genome	gi|150002608|ref|NC_009614.1| Bacteroides vulgatus ATCC 8482 chromosome, complete genome	gi|150002608|ref|NC_009614.1| Bacteroides vulgatus ATCC 8482 chromosome, complete genome
231	2253	N	1	1	Sipho	0	0	1	NA	NA	NA	NA
232	2439	N	0	0	NA	0	0	0	NA	NA	NA	NA
233	1997	N	2	1	Sipho	0	0	1	NA	NA	NA	NA
234	1292	N	1	0	NA	0	0	0	NA	NA	NA	NA
235	1329	N	0	0	NA	0	0	0	NA	NA	NA	NA
236	1656	N	1	0	NA	0	0	0	NA	NA	NA	NA
237	1185	N	2	0	NA	0	0	0	NA	NA	NA	NA
238	1433	N	2	0	NA	0	0	0	NA	Unidentified phage clone 2011_scaffold3 genomic sequence	NA	Unidentified phage clone 2011_scaffold3 genomic sequence
239	1097	N	2	0	NA	0	0	0	NA	NA	NA	NA
240	1328	N	0	0	NA	0	0	0	NA	NA	NA	NA
241	1493	N	2	0	NA	0	0	0	NA	Faecalibacterium prausnitzii L2/6 draft genome	gi|479208076|ref|NC_021042.1| Faecalibacterium prausnitzii L2-6, complete genome	gi|479208076|ref|NC_021042.1| Faecalibacterium prausnitzii L2-6, complete genome
242	1278	N	1	0	NA	0	0	0	NA	NA	NA	NA
243	1833	N	2	1	Sipho	1	0	1	NA	Unidentified phage clone 2011_scaffold3 genomic sequence	NA	Unidentified phage clone 2011_scaffold3 genomic sequence
244	1402	N	1	0	NA	0	0	0	NA	Eubacterium rectale DSM 17629 draft genome	gi|479140210|ref|NC_021010.1| Eubacterium rectale DSM 17629 draft genome	gi|479140210|ref|NC_021010.1| Eubacterium rectale DSM 17629 draft genome

245	1190	N	0	0	NA	0	0	0	NA	Uncultured organism clone 1041059764542 genomic sequence	gi|479162165|ref|NC_021017.1| Bacteroides xylanisolvens XB1A draft genome	gi|479162165|ref|NC_021017.1| Bacteroides xylanisolvens XB1A draft genome
246	1373	N	1	0	NA	0	0	0	NA	Klebsiella pneumoniae JM45, complete genome	gi|530627845|ref|NC_022082.1| Klebsiella pneumoniae JM45, complete genome	gi|530627845|ref|NC_022082.1| Klebsiella pneumoniae JM45, complete genome
247	1132	N	1	0	NA	0	0	0	NA	NA	NA	NA
248	1178	N	0	0	NA	0	0	0	NA	NA	NA	NA
249	1691	N	1	0	NA	0	0	0	NA	Lactobacillus casei W56 complete genome	gi|409995665|ref|NC_018641.1| Lactobacillus casei W56, complete genome	gi|409995665|ref|NC_018641.1| Lactobacillus casei W56, complete genome
250	1546	N	0	0	NA	0	0	0	NA	Haemophilus influenzae F3047 complete genome	gi|319774951|ref|NC_014922.1| Haemophilus influenzae F3047 chromosome, complete genome	gi|319774951|ref|NC_014922.1| Haemophilus influenzae F3047 chromosome, complete genome
251	1098	N	0	0	NA	0	0	0	NA	NA	NA	NA
252	1041	N	1	0	NA	0	0	0	NA	NA	NA	NA
253	1356	N	0	0	NA	0	0	0	NA	Bacteroides vulgatus ATCC 8482, complete genome	gi|150002608|ref|NC_009614.1| Bacteroides vulgatus ATCC 8482 chromosome, complete genome	gi|150002608|ref|NC_009614.1| Bacteroides vulgatus ATCC 8482 chromosome, complete genome

254	1223	N	0	0	NA	0	0	0	NA	Bacteroides thetaiotaomicron VPI-5482, complete genome	gi|29345410|ref|NC_004663.1| Bacteroides thetaiotaomicron VPI-5482 chromosome, complete genome	gi|29345410|ref|NC_004663.1| Bacteroides thetaiotaomicron VPI-5482 chromosome, complete genome
255	1521	N	1	0	NA	0	0	0	NA	NA	NA	NA
256	1639	N	2	0	NA	0	1	0	NA	NA	NA	NA
257	1282	N	1	0	NA	0	0	0	NA	NA	NA	NA
258	1570	N	2	0	NA	0	0	0	NA	Haemophilus parainfluenzae T3T1 complete genome	gi|345428590|ref|NC_015964.1| Haemophilus parainfluenzae T3T1, complete genome	gi|345428590|ref|NC_015964.1| Haemophilus parainfluenzae T3T1, complete genome
259	1211	N	0	0	NA	0	0	0	NA	Uncultured bacterium 52B7 8-amino-7-oxononanoate synthase gene, partial cds; and hypothetical protein, efflux ABC transporter permease protein, putative ABC transporter ATP-binding protein, efflux ABC transporter permease proteins, hypothetical protein, efflux ABC transporter permease protein, hypothetical protein, putative outer membrane efflux protein, hypothetical proteins, putative two-component system response regulators, two-component system sensor histidine kinase, hypothetical proteins, putative dinitrogenase iron-molybdenum cofactor, hypothetical protein, putative integrase, hypothetical proteins, putative lipoprotein, and	NA	Uncultured bacterium 52B7 8-amino-7-oxononanoate synthase gene, partial cds; and hypothetical protein, efflux ABC transporter permease protein, putative ABC transporter ATP-binding protein, efflux ABC transporter permease proteins, hypothetical protein, efflux ABC transporter permease protein, hypothetical protein, putative outer membrane

260	1679	N	1	0	NA	0	0	0	NA	NA	NA	NA
261	1588	N	1	0	NA	0	0	0	NA	Ruminococcus obeum A2-162 draft genome	gi|479176048|ref|NC_021022.1| Ruminococcus obeum A2-162 draft genome	gi|479176048|ref|NC_021022.1| Ruminococcus obeum A2-162 draft genome
262	1484	N	1	0	NA	0	0	0	NA	NA	NA	NA
263	2443	N	4	1	Sipho	0	0	2	NA	NA	NA	NA
264	1833	N	0	0	NA	0	0	0	NA	NA	NA	NA
265	973	N	0	0	NA	0	0	0	NA	NA	NA	NA
266	1394	N	0	0	NA	0	0	0	NA	NA	NA	NA
267	1167	N	2	0	NA	0	0	0	NA	NA	NA	NA
268	1065	N	1	0	NA	0	0	0	NA	NA	NA	NA
269	2150	N	1	0	NA	0	0	0	NA	NA	NA	NA
270	1390	N	2	0	NA	0	0	0	NA	NA	NA	NA
271	1075	N	2	0	NA	0	0	0	NA	Bacteroides vulgatus ATCC 8482, complete genome	gi|150002608|ref|NC_009614.1| Bacteroides vulgatus ATCC 8482 chromosome, complete genome	gi|150002608|ref|NC_009614.1| Bacteroides vulgatus ATCC 8482 chromosome, complete genome
272	1248	N	0	0	NA	0	0	0	NA	Bacteroides fragilis YCH46 DNA, complete genome	gi|53711291|ref|NC_006347.1| Bacteroides fragilis YCH46 DNA, complete genome	gi|53711291|ref|NC_006347.1| Bacteroides fragilis YCH46 DNA, complete genome
273	1557	N	1	0	NA	0	0	0	NA	Uncultured organism clone 1041059767135 genomic sequence	gi|319899888|ref|NC_014933.1| Bacteroides helcogenes P 36-108 chromosome, complete genome	gi|319899888|ref|NC_014933.1| Bacteroides helcogenes P 36-108 chromosome, complete genome

274	1141	N	1	0	NA	0	0	0	NA	Uncultured bacterium fructooligosaccharides degrading DNA fragment, clone MetaPbio 9a	NA	Uncultured bacterium fructooligosaccharides degrading DNA fragment, clone MetaPbio 9a
275	1593	N	1	0	NA	0	0	0	NA	Odoribacter splanchnicus DSM 20712, complete genome	gi|325278757|ref|NC_015160.1| Odoribacter splanchnicus DSM 20712 chromosome, complete genome	gi|325278757|ref|NC_015160.1| Odoribacter splanchnicus DSM 20712 chromosome, complete genome
276	1363	N	0	0	NA	0	0	0	NA	NA	NA	NA
277	1151	N	0	0	NA	0	0	0	NA	NA	NA	NA
278	1102	N	0	0	NA	0	0	0	NA	Bacteroides fragilis mobilization protein MbpA (mbpA), MbpB (mbpB) and MbpC (mbpC) genes, complete cds	NA	Bacteroides fragilis mobilization protein MbpA (mbpA), MbpB (mbpB) and MbpC (mbpC) genes, complete cds
279	964	N	0	0	NA	0	0	0	NA	NA	NA	NA
280	1209	N	1	0	NA	0	0	0	NA	NA	NA	NA
281	1249	N	1	0	NA	0	0	0	NA	NA	NA	NA
282	1254	N	0	0	NA	0	0	0	NA	Haemophilus parainfluenzae T3T1 complete genome	gi|345428590|ref|NC_015964.1| Haemophilus parainfluenzae T3T1, complete genome	gi|345428590|ref|NC_015964.1| Haemophilus parainfluenzae T3T1, complete genome
283	1206	N	1	1	Podo	0	1	1	NA	NA	NA	NA
284	1429	N	1	0	NA	0	0	0	NA	NA	NA	NA
285	1725	N	1	0	NA	0	0	0	NA	NA	NA	NA

286	857	N	0	0	NA	0	0	0	NA	Haemophilus influenzae 10810 genome	gi|378696079|ref|NC_016809.1| Haemophilus influenzae 10810, complete genome	gi|378696079|ref|NC_016809.1| Haemophilus influenzae 10810, complete genome
287	1759	N	1	0	NA	0	0	0	NA	NA	NA	NA
288	1773	N	1	0	NA	0	0	0	NA	Haemophilus parainfluenzae T3T1 complete genome	gi|345428590|ref|NC_015964.1| Haemophilus parainfluenzae T3T1, complete genome	gi|345428590|ref|NC_015964.1| Haemophilus parainfluenzae T3T1, complete genome
289	974	N	0	0	NA	0	0	0	NA	NA	NA	NA
290	986	N	0	0	NA	0	0	0	NA	Faecalibacterium prausnitzii L2/6 draft genome	gi|479208076|ref|NC_021042.1| Faecalibacterium prausnitzii L2-6, complete genome	gi|479208076|ref|NC_021042.1| Faecalibacterium prausnitzii L2-6, complete genome
291	2505	N	2	2	Sipho	1	0	2	NA	NA	NA	NA
292	1055	N	1	0	NA	0	0	0	NA	Bacteroides vulgatus ATCC 8482, complete genome	gi|150002608|ref|NC_009614.1| Bacteroides vulgatus ATCC 8482 chromosome, complete genome	gi|150002608|ref|NC_009614.1| Bacteroides vulgatus ATCC 8482 chromosome, complete genome
293	1515	N	2	1	Sipho	0	0	1	NA	NA	NA	NA
294	876	N	0	0	NA	0	0	0	NA	NA	NA	NA
295	1033	N	1	0	NA	0	0	0	NA	NA	NA	NA
296	1169	N	1	0	NA	0	0	0	NA	NA	NA	NA
297	774	N	0	0	NA	0	0	0	NA	NA	NA	NA
298	899	N	1	0	NA	0	0	0	NA	NA	NA	NA
299	938	N	0	0	NA	0	0	0	NA	Haemophilus parainfluenzae T3T1 complete genome	gi|345428590|ref|NC_015964.1| Haemophilus parainfluenzae T3T1, complete genome	gi|345428590|ref|NC_015964.1| Haemophilus parainfluenzae T3T1, complete genome

300	888	N	0	0	NA	0	0	0	NA	NA	NA	NA
301	1400	N	1	0	NA	0	0	0	NA	NA	NA	NA
302	862	N	1	0	NA	0	0	0	NA	NA	NA	NA
303	1330	N	1	0	NA	0	0	0	NA	NA	NA	NA
304	938	N	0	0	NA	0	0	0	NA	Bacteroides fragilis strain IB143 plasmid pBI143, complete sequence	NA	Bacteroides fragilis strain IB143 plasmid pBI143, complete sequence
305	989	N	1	0	NA	0	0	0	NA	NA	NA	NA
306	1152	N	1	0	NA	0	0	0	NA	NA	NA	NA
307	854	N	1	0	NA	0	0	0	NA	NA	NA	NA
308	807	N	0	0	NA	0	0	0	NA	NA	NA	NA
309	745	N	0	0	NA	0	0	0	NA	Veillonella parvula DSM 2008, complete genome	gi|269797069|ref|NC_013520.1| Veillonella parvula DSM 2008 chromosome, complete genome	gi|269797069|ref|NC_013520.1| Veillonella parvula DSM 2008 chromosome, complete genome
310	913	N	0	0	NA	0	0	0	NA	Haemophilus parainfluenzae T3T1 complete genome	gi|345428590|ref|NC_015964.1| Haemophilus parainfluenzae T3T1, complete genome	gi|345428590|ref|NC_015964.1| Haemophilus parainfluenzae T3T1, complete genome
311	898	N	1	0	NA	0	0	0	NA	NA	NA	NA
312	846	N	0	0	NA	0	0	0	NA	NA	NA	NA
313	1024	N	0	0	NA	0	0	0	NA	NA	NA	NA
314	861	N	0	0	NA	0	0	0	NA	Streptococcus pyogenes M1 GAS, complete genome	gi|15674250|ref|NC_002737.1| Streptococcus pyogenes SF370 chromosome, complete genome	gi|15674250|ref|NC_002737.1| Streptococcus pyogenes SF370 chromosome, complete genome
315	1075	N	1	0	NA	0	0	0	NA	NA	NA	NA
316	862	N	0	0	NA	0	0	0	NA	NA	NA	NA

317	929	N	0	0	NA	0	0	0	NA	Haemophilus parainfluenzae T3T1 complete genome	gi|345428590|ref|NC_015964.1| Haemophilus parainfluenzae T3T1, complete genome	gi|345428590|ref|NC_015964.1| Haemophilus parainfluenzae T3T1, complete genome
318	860	N	2	0	NA	0	0	0	NA	Faecalibacterium prausnitzii L2/6 draft genome	gi|479208076|ref|NC_021042.1| Faecalibacterium prausnitzii L2-6, complete genome	gi|479208076|ref|NC_021042.1| Faecalibacterium prausnitzii L2-6, complete genome
319	738	N	0	0	NA	0	0	0	NA	NA	NA	NA
320	679	N	1	0	NA	0	0	0	NA	NA	NA	NA
321	871	N	1	0	NA	0	0	0	NA	NA	NA	NA
322	672	N	1	0	NA	0	0	0	NA	NA	NA	NA
323	886	N	0	0	NA	0	0	0	NA	Bacteroides fragilis plasmid pBFUK1 DNA, complete genome, strain: GAI92082	NA	Bacteroides fragilis plasmid pBFUK1 DNA, complete genome, strain: GAI92082
324	910	N	1	0	NA	0	0	0	NA	Bacteroides vulgatus ATCC 8482, complete genome	gi|150002608|ref|NC_009614.1| Bacteroides vulgatus ATCC 8482 chromosome, complete genome	gi|150002608|ref|NC_009614.1| Bacteroides vulgatus ATCC 8482 chromosome, complete genome
325	1007	N	0	0	NA	0	0	0	NA	NA	NA	NA
326	1160	N	1	0	NA	0	0	0	NA	NA	NA	NA
327	1643	N	1	0	NA	0	0	0	NA	NA	NA	NA
328	918	N	0	0	NA	0	0	0	NA	NA	NA	NA
329	668	N	0	0	NA	0	0	0	NA	NA	NA	NA
330	662	N	0	0	NA	0	0	0	NA	NA	NA	NA
331	730	N	3	0	NA	0	0	0	NA	NA	NA	NA

332	1019	N	0	0	NA	0	0	0	NA	Haemophilus parainfluenzae T3T1 complete genome	gi|345428590|ref|NC_015964.1| Haemophilus parainfluenzae T3T1, complete genome	gi|345428590|ref|NC_015964.1| Haemophilus parainfluenzae T3T1, complete genome
333	795	N	0	0	NA	0	0	0	NA	NA	NA	NA
334	940	N	0	0	NA	0	0	0	NA	Uncultured bacterium clone HA0AAA18ZE11FM1 genomic sequence	NA	Uncultured bacterium clone HA0AAA18ZE11FM1 genomic sequence
335	822	N	1	0	NA	0	0	0	NA	NA	NA	NA
336	872	N	0	0	NA	0	0	0	NA	NA	NA	NA
337	998	N	2	0	NA	0	0	0	NA	NA	NA	NA
338	1467	N	0	0	NA	0	0	0	NA	NA	NA	NA
339	830	N	0	0	NA	0	0	0	NA	NA	NA	NA
340	735	N	0	0	NA	0	0	0	NA	NA	NA	NA
341	778	N	0	0	NA	0	0	0	NA	Uncultured bacterium clone LM0ABA27ZC06FM1 genomic sequence	gi|150002608|ref|NC_009614.1| Bacteroides vulgatus ATCC 8482 chromosome, complete genome	gi|150002608|ref|NC_009614.1| Bacteroides vulgatus ATCC 8482 chromosome, complete genome
342	587	N	0	0	NA	0	0	0	NA	TPA_exp: Clostridium difficile strain R20291 transposon Tn6103, complete sequence	gi|260685375|ref|NC_013316.1| Clostridium difficile R20291 chromosome, complete genome	gi|260685375|ref|NC_013316.1| Clostridium difficile R20291 chromosome, complete genome
343	641	N	0	0	NA	0	0	0	NA	NA	NA	NA
344	660	N	0	0	NA	0	0	0	NA	Bacteroides fragilis plasmid pBFUK1 DNA, complete genome, strain: GAI92082	NA	Bacteroides fragilis plasmid pBFUK1 DNA, complete genome, strain: GAI92082

345	775	N	0	0	NA	0	0	0	NA	NA	NA	NA
346	562	N	0	0	NA	0	0	0	NA	NA	NA	NA
347	854	N	1	0	NA	0	0	0	NA	Uncultured bacterium clone LM0ACA18ZE06RM1 genomic sequence	NA	Uncultured bacterium clone LM0ACA18ZE06RM1 genomic sequence
348	655	N	0	0	NA	0	0	0	NA	NA	NA	NA
349	934	N	1	0	NA	0	0	0	NA	NA	NA	NA
350	731	N	0	0	NA	0	0	0	NA	NA	NA	NA
351	619	N	0	0	NA	0	0	0	NA	NA	NA	NA
352	638	N	1	0	NA	0	0	0	NA	NA	NA	NA
353	667	N	0	0	NA	0	0	0	NA	Haemophilus parainfluenzae T3T1 complete genome	gi|345428590|ref|NC_015964.1| Haemophilus parainfluenzae T3T1, complete genome	gi|345428590|ref|NC_015964.1| Haemophilus parainfluenzae T3T1, complete genome
354	706	N	0	0	NA	0	0	0	NA	NA	NA	NA
355	642	N	0	0	NA	0	0	0	NA	NA	NA	NA
356	585	N	0	0	NA	0	0	0	NA	Shuttle vector pBA complete sequence	NA	Shuttle vector pBA complete sequence
357	505	N	0	0	NA	0	0	0	NA	NA	NA	NA
358	623	N	2	0	NA	0	0	0	NA	NA	NA	NA
359	531	N	0	0	NA	0	0	0	NA	NA	NA	NA
360	660	N	1	0	NA	0	0	0	NA	Faecalibacterium prausnitzii L2/6 draft genome	gi|479208076|ref|NC_021042.1| Faecalibacterium prausnitzii L2-6, complete genome	gi|479208076|ref|NC_021042.1| Faecalibacterium prausnitzii L2-6, complete genome
361	581	N	0	0	NA	0	0	0	NA	Bacteroides salanitronis DSM 18170, complete genome	gi|325297172|ref|NC_015164.1| Bacteroides salanitronis DSM 18170 chromosome, complete genome	gi|325297172|ref|NC_015164.1| Bacteroides salanitronis DSM 18170 chromosome, complete genome
362	868	N	1	0	NA	0	0	0	NA	NA	NA	NA

363	501	N	0	0	NA	0	0	0	NA	NA	NA	NA
365	628	N	0	0	NA	0	0	0	NA	NA	NA	NA
366	502	N	0	0	NA	0	0	0	NA	NA	NA	NA
367	713	N	0	0	NA	0	0	0	NA	Odoribacter splanchnicus DSM 20712, complete genome	gi|325278757|ref|NC_015160.1| Odoribacter splanchnicus DSM 20712 chromosome, complete genome	gi|325278757|ref|NC_015160.1| Odoribacter splanchnicus DSM 20712 chromosome, complete genome
368	568	N	0	0	NA	0	0	0	NA	NA	NA	NA
369	592	N	0	0	NA	0	0	0	NA	NA	NA	NA
372	582	N	1	0	NA	0	0	0	NA	NA	NA	NA
374	650	N	0	0	NA	0	0	0	NA	Clostridium difficile M68, complete genome	gi|479133135|ref|NC_017175.1| Clostridium difficile M68, complete genome	gi|479133135|ref|NC_017175.1| Clostridium difficile M68, complete genome
377	580	N	0	0	NA	0	0	0	NA	NA	NA	NA
contig-100_1	40656	N	30	10	Sipho	3	2	13	NA	Roseburia intestinalis XB6B4 draft genome	gi|479146200|ref|NC_021012.1| Roseburia intestinalis XB6B4 draft genome	gi|479146200|ref|NC_021012.1| Roseburia intestinalis XB6B4 draft genome
contig-100_1001	1256	N	0	0	NA	0	0	0	NA	NA	NA	NA
contig-100_1002	1255	N	0	0	NA	0	0	0	NA	NA	NA	NA
contig-100_1003	1255	N	2	0	NA	0	0	0	NA	NA	NA	NA
contig-100_1005	1254	N	1	0	NA	0	0	0	NA	Clostridiales sp. SM4/1 draft genome	gi|479181986|ref|NC_021024.1| Butyrate-producing bacterium SM4/1, complete genome	gi|479181986|ref|NC_021024.1| Butyrate-producing bacterium SM4/1, complete genome
contig-100_1006	1253	N	0	0	NA	0	0	0	NA	NA	NA	NA
contig-100_1007	1250	N	0	0	NA	0	0	0	NA	NA	NA	NA
contig-100_1008	1249	N	1	0	NA	0	0	0	NA	NA	NA	NA
contig-100_1009	1248	N	1	0	NA	0	0	0	NA	Uncultured organism clone VC1AO65TR genomic sequence	NA	Uncultured organism clone VC1AO65TR genomic sequence

contig-100_1010	1247	N	1	0	NA	0	0	0	NA	NA	NA	NA
contig-100_1011	1245	N	1	0	NA	0	0	0	NA	NA	NA	NA
contig-100_1012	1245	N	0	0	NA	0	0	0	NA	Haemophilus influenzae F3031 complete genome	gi|319896422|ref|NC_014920.1| Haemophilus influenzae F3031 chromosome, complete genome	gi|319896422|ref|NC_014920.1| Haemophilus influenzae F3031 chromosome, complete genome
contig-100_1014	1242	N	1	0	NA	0	0	0	NA	NA	NA	NA
contig-100_1015	1242	N	0	0	NA	0	0	0	NA	NA	NA	NA
contig-100_1016	1241	N	2	0	NA	0	0	0	NA	NA	NA	NA
contig-100_1017	1241	N	1	0	NA	0	0	0	NA	NA	NA	NA
contig-100_1018	1241	N	0	0	NA	0	0	0	NA	NA	NA	NA
contig-100_1019	1240	N	2	0	NA	0	0	0	NA	NA	NA	NA
contig-100_1020	1237	N	1	0	NA	0	0	0	NA	Haemophilus parainfluenzae T3T1 complete genome	gi|345428590|ref|NC_015964.1| Haemophilus parainfluenzae T3T1, complete genome	gi|345428590|ref|NC_015964.1| Haemophilus parainfluenzae T3T1, complete genome
contig-100_1021	1237	N	1	0	NA	0	0	0	NA	Roseburia intestinalis M50/1 draft genome	gi|479201824|ref|NC_021040.1| Roseburia intestinalis M50/1 draft genome	gi|479201824|ref|NC_021040.1| Roseburia intestinalis M50/1 draft genome
contig-100_1023	1236	N	1	0	NA	0	0	0	NA	Uncultured bacterium clone LM0ABA42ZG12RM1 genomic sequence	NA	Uncultured bacterium clone LM0ABA42ZG12RM1 genomic sequence
contig-100_1024	1235	N	2	0	NA	0	0	0	NA	Bacteroides fragilis strain IB143 plasmid pBI143, complete sequence	gi|150006674|ref|NC_009615.1| Parabacteroides distasonis ATCC 8503 chromosome, complete genome	gi|150006674|ref|NC_009615.1| Parabacteroides distasonis ATCC 8503 chromosome, complete genome
contig-100_1025	1233	N	2	1	Sipho	0	0	1	NA	NA	NA	NA
contig-100_1027	1233	N	2	0	NA	0	0	0	NA	NA	NA	NA

contig-100_1028	1232	N	0	0	NA	0	0	0	NA	NA	NA	NA
contig-100_1029	1232	N	0	0	NA	0	0	0	NA	NA	NA	NA
contig-100_1030	1231	N	0	0	NA	0	0	0	NA	NA	NA	NA
contig-100_1031	1230	N	0	0	NA	0	0	0	NA	Uncultured bacterium clone LM0ABA27ZE01RM1 genomic sequence	gi|150002608|ref|NC_009614.1| Bacteroides vulgatus ATCC 8482 chromosome, complete genome	gi|150002608|ref|NC_009614.1| Bacteroides vulgatus ATCC 8482 chromosome, complete genome
contig-100_1034	1230	N	1	0	NA	0	0	0	NA	NA	NA	NA
contig-100_1035	1230	N	1	0	NA	0	0	0	NA	NA	NA	NA
contig-100_1036	1229	N	1	0	NA	0	0	0	NA	NA	NA	NA
contig-100_1037	1228	N	0	0	NA	0	0	0	NA	NA	NA	NA
contig-100_1038	1227	N	0	0	NA	0	0	0	NA	NA	NA	NA
contig-100_1039	1226	N	0	0	NA	0	0	0	NA	NA	NA	NA
contig-100_1040	1226	N	2	0	NA	0	0	0	NA	NA	NA	NA
contig-100_1041	1225	N	1	0	NA	0	0	0	NA	NA	NA	NA
contig-100_1042	1224	N	1	0	NA	0	0	0	NA	NA	NA	NA
contig-100_1043	1223	N	1	0	NA	0	0	0	NA	NA	NA	NA
contig-100_1044	1223	N	0	0	NA	0	0	0	NA	NA	NA	NA
contig-100_1045	1222	N	2	0	NA	0	0	0	NA	NA	NA	NA
contig-100_1046	1221	N	2	0	NA	0	0	0	NA	Clostridium difficile 630 complete genome	gi|126697566|ref|NC_009089.1| Peptoclostridium difficile 630, complete genome	gi|126697566|ref|NC_009089.1| Peptoclostridium difficile 630, complete genome
contig-100_1048	1221	N	0	0	NA	0	0	0	NA	NA	NA	NA
contig-100_1049	1221	N	1	0	NA	0	0	0	NA	NA	NA	NA
contig-100_105	5159	N	4	0	NA	0	0	0	NA	NA	NA	NA
contig-100_1050	1220	N	1	0	NA	0	0	0	NA	NA	NA	NA
contig-100_1051	1220	N	1	0	NA	0	0	0	NA	NA	NA	NA
contig-100_1052	1220	N	0	0	NA	0	0	0	NA	Haemophilus parainfluenzae T3T1 complete genome	gi|345428590|ref|NC_015964.1| Haemophilus parainfluenzae T3T1, complete genome	gi|345428590|ref|NC_015964.1| Haemophilus parainfluenzae T3T1, complete genome

contig-100_1053	1219	N	1	0	NA	0	0	0	NA	Unidentified phage clone 2019_scaffold132 genomic sequence	NA	Unidentified phage clone 2019_scaffold 132 genomic sequence
contig-100_1054	1219	N	0	0	NA	0	0	0	NA	NA	NA	NA
contig-100_1056	1218	N	1	0	NA	0	0	0	NA	NA	NA	NA
contig-100_1057	1218	N	0	0	NA	0	0	0	NA	NA	NA	NA
contig-100_1058	1217	N	1	0	NA	0	0	0	NA	Streptococcus dysgalactiae subsp. equisimilis GGS_124 DNA, complete genome	gi|251781468|ref|NC_012891.1| Streptococcus dysgalactiae subsp. equisimilis GGS_124 chromosome 1, complete sequence	gi|251781468|ref|NC_012891.1| Streptococcus dysgalactiae subsp. equisimilis GGS_124 chromosome 1, complete sequence
contig-100_1059	1214	N	1	0	NA	0	0	0	NA	NA	NA	NA
contig-100_1060	1214	N	1	0	NA	0	0	0	NA	Eubacterium rectale ATCC 33656, complete genome	gi|238922432|ref|NC_012781.1| Eubacterium rectale ATCC 33656, complete genome	gi|238922432|ref|NC_012781.1| Eubacterium rectale ATCC 33656, complete genome
contig-100_1062	1213	N	0	0	NA	0	0	0	NA	Alistipes shahii WAL 8301 draft genome	gi|479185170|ref|NC_021030.1| Alistipes shahii WAL 8301 draft genome	gi|479185170|ref|NC_021030.1| Alistipes shahii WAL 8301 draft genome
contig-100_1063	1212	N	2	0	NA	0	0	0	NA	NA	NA	NA
contig-100_1064	1212	N	1	0	NA	0	0	0	NA	NA	NA	NA
contig-100_1065	1210	N	0	0	NA	0	0	0	NA	Lactobacillus helveticus CNRZ32, complete genome	gi|525706521|ref|NC_021744.1| Lactobacillus helveticus CNRZ32, complete genome	gi|525706521|ref|NC_021744.1| Lactobacillus helveticus CNRZ32, complete genome
contig-100_1066	1210	N	0	0	NA	0	0	0	NA	NA	NA	NA
contig-100_1068	1209	N	0	0	NA	0	0	0	NA	NA	NA	NA

contig-100_107	4918	N	2	0	NA	0	0	1	NA	Bacteroides thetaiotaomicron VPI-5482, complete genome	gi|29345410|ref|NC_004663.1| Bacteroides thetaiotaomicron VPI-5482 chromosome, complete genome	gi|29345410|ref|NC_004663.1| Bacteroides thetaiotaomicron VPI-5482 chromosome, complete genome
contig-100_1071	1208	N	1	0	NA	0	0	0	NA	NA	NA	NA
contig-100_1074	1207	N	1	0	NA	0	0	0	NA	Haemophilus parainfluenzae T3T1 complete genome	gi|345428590|ref|NC_015964.1| Haemophilus parainfluenzae T3T1, complete genome	gi|345428590|ref|NC_015964.1| Haemophilus parainfluenzae T3T1, complete genome
contig-100_1075	1205	N	0	0	NA	0	0	0	NA	NA	NA	NA
contig-100_1076	1205	N	1	0	NA	0	0	0	NA	NA	NA	NA
contig-100_1078	1203	N	1	0	NA	0	0	0	NA	Uncultured organism clone 1041059765766 genomic sequence	gi|375143366|ref|NC_016609.1| Niastella koreensis GR20-10 chromosome, complete genome	gi|375143366|ref|NC_016609.1| Niastella koreensis GR20-10 chromosome, complete genome
contig-100_1079	1203	N	1	0	NA	0	0	0	NA	Faecalibacterium prausnitzii L2/6 draft genome	gi|479208076|ref|NC_021042.1| Faecalibacterium prausnitzii L2-6, complete genome	gi|479208076|ref|NC_021042.1| Faecalibacterium prausnitzii L2-6, complete genome
contig-100_1080	1203	N	1	0	NA	0	0	0	NA	NA	NA	NA
contig-100_1082	1202	N	1	0	NA	0	0	0	NA	NA	NA	NA
contig-100_1083	1201	N	0	0	NA	0	0	0	NA	NA	NA	NA
contig-100_1084	1200	N	1	0	NA	0	0	0	NA	NA	NA	NA
contig-100_1085	1199	N	0	0	NA	0	0	0	NA	Bacteroides vulgatus ATCC 8482, complete genome	gi|150002608|ref|NC_009614.1| Bacteroides vulgatus ATCC 8482 chromosome, complete genome	gi|150002608|ref|NC_009614.1| Bacteroides vulgatus ATCC 8482 chromosome, complete genome
contig-100_1088	1198	N	1	0	NA	0	0	0	NA	NA	NA	NA

contig-100_1089	1198	N	0	0	NA	0	0	0	NA	Haemophilus parainfluenzae T3T1 complete genome	gi|345428590|ref|NC_015964.1| Haemophilus parainfluenzae T3T1, complete genome	gi|345428590|ref|NC_015964.1| Haemophilus parainfluenzae T3T1, complete genome
contig-100_109	4857	N	4	1	Sipho	0	0	1	NA	NA	NA	NA
contig-100_1090	1197	N	1	0	NA	0	0	0	NA	Pseudomonas fluorescens SBW25 complete genome	gi|568136993|ref|NC_023064.1| Pseudomonas sp. TKP, complete genome	gi|568136993|ref|NC_023064.1| Pseudomonas sp. TKP, complete genome
contig-100_1091	1197	N	0	0	NA	0	0	0	NA	NA	NA	NA
contig-100_1092	1196	N	0	0	NA	0	0	0	NA	NA	NA	NA
contig-100_1096	1195	N	2	0	NA	0	0	0	NA	NA	NA	NA
contig-100_1097	1194	N	1	0	NA	0	0	0	NA	NA	NA	NA
contig-100_1098	1193	N	0	0	NA	0	0	0	NA	Eubacterium eligens ATCC 27750 plasmid, complete sequence	gi|238921767|ref|NC_012780.1| Eubacterium eligens ATCC 27750 plasmid unnamed, complete sequence	gi|238921767|ref|NC_012780.1| Eubacterium eligens ATCC 27750 plasmid unnamed, complete sequence
contig-100_1099	1193	N	2	0	NA	0	0	0	NA	Faecalibacterium prausnitzii SL3/3 draft genome	gi|479170689|ref|NC_021020.1| Faecalibacterium prausnitzii SL3/3 draft genome	gi|479170689|ref|NC_021020.1| Faecalibacterium prausnitzii SL3/3 draft genome
contig-100_1102	1191	N	0	0	NA	0	0	0	NA	Faecalibacterium prausnitzii SL3/3 draft genome	gi|479170689|ref|NC_021020.1| Faecalibacterium prausnitzii SL3/3 draft genome	gi|479170689|ref|NC_021020.1| Faecalibacterium prausnitzii SL3/3 draft genome
contig-100_1103	1190	N	0	0	NA	0	0	0	NA	NA	NA	NA
contig-100_1104	1190	N	0	0	NA	0	0	0	NA	NA	NA	NA

contig-100_1105	1190	N	2	0	NA	0	0	0	NA	Faecalibacterium prausnitzii SL3/3 draft genome	gi|479170689|ref|NC_021020.1| Faecalibacterium prausnitzii SL3/3 draft genome	gi|479170689|ref|NC_021020.1| Faecalibacterium prausnitzii SL3/3 draft genome
contig-100_1106	1190	N	0	0	NA	0	0	0	NA	NA	NA	NA
contig-100_1108	1188	N	0	0	NA	0	0	0	NA	NA	NA	NA
contig-100_1109	1187	N	2	0	NA	0	0	0	NA	Uncultured bacterium clone LM0ABA29ZE06RM1 genomic sequence	gi|319899888|ref|NC_014933.1| Bacteroides helcogenes P 36-108 chromosome, complete genome	gi|319899888|ref|NC_014933.1| Bacteroides helcogenes P 36-108 chromosome, complete genome
contig-100_111	4801	Y	5	2	Micro	0	0	2	NA	NA	NA	NA
contig-100_1111	1187	N	1	0	NA	0	0	0	NA	NA	NA	NA
contig-100_1112	1186	N	0	0	NA	0	0	0	NA	NA	NA	NA
contig-100_1113	1186	N	1	0	NA	0	0	0	NA	Pseudomonas protegens CHA0, complete genome	gi|501677485|ref|NC_021237.1| Pseudomonas protegens CHA0, complete genome	gi|501677485|ref|NC_021237.1| Pseudomonas protegens CHA0, complete genome
contig-100_1115	1185	N	1	0	NA	0	0	0	NA	Haemophilus parainfluenzae T3T1 complete genome	gi|345428590|ref|NC_015964.1| Haemophilus parainfluenzae T3T1, complete genome	gi|345428590|ref|NC_015964.1| Haemophilus parainfluenzae T3T1, complete genome
contig-100_1118	1183	N	0	0	NA	0	0	0	NA	Bacteroides vulgatus ATCC 8482, complete genome	gi|150002608|ref|NC_009614.1| Bacteroides vulgatus ATCC 8482 chromosome, complete genome	gi|150002608|ref|NC_009614.1| Bacteroides vulgatus ATCC 8482 chromosome, complete genome
contig-100_1119	1183	N	0	0	NA	0	0	0	NA	NA	NA	NA

contig-100_1123	1180	N	1	0	NA	0	0	0	NA	Haemophilus parainfluenzae T3T1 complete genome	gi|345428590|ref|NC_015964.1| Haemophilus parainfluenzae T3T1, complete genome	gi|345428590|ref|NC_015964.1| Haemophilus parainfluenzae T3T1, complete genome
contig-100_1125	1179	N	1	0	NA	0	0	0	NA	NA	NA	NA
contig-100_1126	1178	N	1	0	NA	0	0	0	NA	NA	NA	NA
contig-100_1128	1176	N	0	0	NA	0	0	0	NA	Uncultured bacterium clone HA0AAA13ZD11RM1 genomic sequence	NA	Uncultured bacterium clone HA0AAA13ZD11RM1 genomic sequence
contig-100_1129	1176	N	1	0	NA	0	0	0	NA	Bacteroides vulgatus ATCC 8482, complete genome	gi|150002608|ref|NC_009614.1| Bacteroides vulgatus ATCC 8482 chromosome, complete genome	gi|150002608|ref|NC_009614.1| Bacteroides vulgatus ATCC 8482 chromosome, complete genome
contig-100_113	4753	N	2	1	Sipho	0	1	1	NA	Enterobacteria phage HK629, complete genome	gi|253771435|ref|NC_012947.1| Escherichia coli 'BL21-Gold(DE3)pLysS AG' chromosome, complete genome	gi|253771435|ref|NC_012947.1| Escherichia coli 'BL21-Gold(DE3)pLysS AG' chromosome, complete genome
contig-100_1130	1176	N	2	0	NA	0	0	0	NA	Bacteroides vulgatus ATCC 8482, complete genome	gi|150002608|ref|NC_009614.1| Bacteroides vulgatus ATCC 8482 chromosome, complete genome	gi|150002608|ref|NC_009614.1| Bacteroides vulgatus ATCC 8482 chromosome, complete genome

contig-100_1131	1176	N	0	0	NA	0	0	0	NA	Bacteroides fragilis YCH46 DNA, complete genome	gi|53711291|ref|NC_006347.1| Bacteroides fragilis YCH46 DNA, complete genome	gi|53711291|ref|NC_006347.1| Bacteroides fragilis YCH46 DNA, complete genome
contig-100_1132	1175	N	2	0	NA	0	0	0	NA	NA	NA	NA
contig-100_1133	1173	N	0	0	NA	0	0	0	NA	NA	NA	NA
contig-100_1134	1173	N	2	0	NA	0	0	0	NA	NA	NA	NA
contig-100_1135	1173	N	1	0	NA	0	0	0	NA	NA	NA	NA
contig-100_1137	1172	N	0	0	NA	0	0	0	NA	NA	NA	NA
contig-100_1138	1171	N	0	0	NA	0	0	0	NA	NA	NA	NA
contig-100_1139	1171	N	1	0	NA	0	0	0	NA	NA	NA	NA
contig-100_1140	1171	N	1	0	NA	0	0	0	NA	NA	NA	NA
contig-100_1141	1171	N	0	0	NA	0	0	0	NA	NA	NA	NA
contig-100_1142	1171	N	1	0	NA	0	0	0	NA	NA	NA	NA
contig-100_1143	1168	N	0	0	NA	0	0	0	NA	NA	NA	NA
contig-100_1144	1168	N	1	0	NA	0	0	0	NA	Clostridium clostridioforme strain CIP 110263 integrative conjugative element ICECCm201 genomic sequence	gi|92112136|ref|NC_007963.1| Chromohalobacter salexigens DSM 3043 chromosome, complete genome	gi|92112136|ref|NC_007963.1| Chromohalobacter salexigens DSM 3043 chromosome, complete genome
contig-100_1145	1168	N	0	0	NA	0	0	0	NA	Escherichia coli str. K-12 substr. MG1655, complete genome	gi|388476123|ref|NC_007779.1| Escherichia coli str. K-12 substr. W3110, complete genome	gi|388476123|ref|NC_007779.1| Escherichia coli str. K-12 substr. W3110, complete genome
contig-100_1146	1168	N	0	0	NA	0	0	0	NA	NA	NA	NA
contig-100_1147	1167	N	1	0	NA	0	0	0	NA	Uncultured organism clone 1041059766209 genomic sequence	NA	Uncultured organism clone 1041059766209 genomic sequence

contig-100_1149	1167	N	1	0	NA	0	0	0	NA	Uncultured organism clone 1041059764712 genomic sequence	NA	Uncultured organism clone 1041059764712 genomic sequence
contig-100_1150	1167	N	0	0	NA	0	0	0	NA	NA	NA	NA
contig-100_1151	1166	N	1	0	NA	0	0	0	NA	NA	NA	NA
contig-100_1152	1163	N	0	0	NA	0	0	0	NA	Uncultured bacterium clone SJTU_G_06_83 16S ribosomal RNA gene, partial sequence	gi|479210985|ref|NC_021043.1| Eubacterium siraeum V10Sc8a draft genome	gi|479210985|ref|NC_021043.1| Eubacterium siraeum V10Sc8a draft genome
contig-100_1153	1163	N	1	0	NA	0	0	0	NA	Haemophilus parainfluenzae T3T1 complete genome	gi|345428590|ref|NC_015964.1| Haemophilus parainfluenzae T3T1, complete genome	gi|345428590|ref|NC_015964.1| Haemophilus parainfluenzae T3T1, complete genome
contig-100_1154	1163	N	0	0	NA	0	0	0	NA	NA	NA	NA
contig-100_1156	1161	N	2	0	NA	0	0	0	NA	NA	NA	NA
contig-100_1158	1161	N	1	0	NA	0	0	0	NA	Haemophilus parainfluenzae T3T1 complete genome	gi|345428590|ref|NC_015964.1| Haemophilus parainfluenzae T3T1, complete genome	gi|345428590|ref|NC_015964.1| Haemophilus parainfluenzae T3T1, complete genome
contig-100_1159	1160	N	0	0	NA	0	0	0	NA	Haemophilus parainfluenzae T3T1 complete genome	gi|345428590|ref|NC_015964.1| Haemophilus parainfluenzae T3T1, complete genome	gi|345428590|ref|NC_015964.1| Haemophilus parainfluenzae T3T1, complete genome
contig-100_1160	1160	N	1	0	NA	0	0	0	NA	NA	NA	NA
contig-100_1161	1160	N	0	0	NA	0	0	0	NA	NA	NA	NA
contig-100_1162	1160	N	1	0	NA	0	0	0	NA	NA	NA	NA

contig-100_1163	1158	N	1	0	NA	0	0	0	NA	Bacteroides salanitronis DSM 18170, complete genome	gi|325297172|ref|NC_015164.1| Bacteroides salanitronis DSM 18170 chromosome, complete genome	gi|325297172|ref|NC_015164.1| Bacteroides salanitronis DSM 18170 chromosome, complete genome
contig-100_1165	1157	N	1	0	NA	0	0	0	NA	NA	NA	NA
contig-100_1166	1156	N	0	0	NA	0	0	0	NA	NA	NA	NA
contig-100_1167	1156	N	2	0	NA	0	0	0	NA	Bacteroides salanitronis DSM 18170, complete genome	gi|325297172|ref|NC_015164.1| Bacteroides salanitronis DSM 18170 chromosome, complete genome	gi|325297172|ref|NC_015164.1| Bacteroides salanitronis DSM 18170 chromosome, complete genome
contig-100_1168	1156	N	2	0	NA	0	0	0	NA	NA	NA	NA
contig-100_1169	1155	N	0	0	NA	0	0	0	NA	Haemophilus influenzae F3047 complete genome	gi|319774951|ref|NC_014922.1| Haemophilus influenzae F3047 chromosome, complete genome	gi|319774951|ref|NC_014922.1| Haemophilus influenzae F3047 chromosome, complete genome
contig-100_117	4665	N	5	1	Sipho	0	0	1	NA	NA	NA	NA
contig-100_1170	1154	N	1	0	NA	0	0	0	NA	NA	NA	NA
contig-100_1171	1154	N	0	0	NA	0	0	0	NA	NA	NA	NA
contig-100_1172	1153	N	1	0	NA	0	0	0	NA	Bacteroides vulgatus ATCC 8482, complete genome	gi|150002608|ref|NC_009614.1| Bacteroides vulgatus ATCC 8482 chromosome, complete genome	gi|150002608|ref|NC_009614.1| Bacteroides vulgatus ATCC 8482 chromosome, complete genome
contig-100_1173	1153	N	0	0	NA	0	0	0	NA	NA	NA	NA
contig-100_1174	1153	N	1	0	NA	0	0	0	NA	NA	NA	NA

contig-100_1175	1152	N	2	0	NA	0	0	0	NA	TPA_exp: Clostridium difficile strain QCD-66C26 transposon Tn6110, complete sequence	gi|260685375|ref|NC_013316.1| Clostridium difficile R20291 chromosome, complete genome	gi|260685375|ref|NC_013316.1| Clostridium difficile R20291 chromosome, complete genome
contig-100_1176	1152	N	2	0	NA	0	0	0	NA	NA	NA	NA
contig-100_1177	1152	N	1	0	NA	0	0	0	NA	NA	NA	NA
contig-100_1178	1152	N	1	0	NA	0	0	0	NA	Neisseria meningitidis WUE 2594 complete genome	gi|385337120|ref|NC_017512.1| Neisseria meningitidis WUE 2594, complete genome	gi|385337120|ref|NC_017512.1| Neisseria meningitidis WUE 2594, complete genome
contig-100_1179	1151	N	1	0	NA	0	0	0	NA	NA	NA	NA
contig-100_118	4658	N	1	0	NA	0	0	0	NA	NA	NA	NA
contig-100_1180	1151	N	0	0	NA	0	0	0	NA	NA	NA	NA
contig-100_1181	1148	N	1	0	NA	0	0	0	NA	Bacteroides vulgatus ATCC 8482, complete genome	gi|150002608|ref|NC_009614.1| Bacteroides vulgatus ATCC 8482 chromosome, complete genome	gi|150002608|ref|NC_009614.1| Bacteroides vulgatus ATCC 8482 chromosome, complete genome
contig-100_1182	1148	N	0	0	NA	0	0	0	NA	TPA_exp: Clostridium difficile strain QCD-66C26 transposon Tn6110, complete sequence	gi|260685375|ref|NC_013316.1| Clostridium difficile R20291 chromosome, complete genome	gi|260685375|ref|NC_013316.1| Clostridium difficile R20291 chromosome, complete genome
contig-100_1183	1148	N	2	0	NA	0	0	0	NA	NA	NA	NA
contig-100_1184	1148	N	0	0	NA	0	0	0	NA	Faecalibacterium prausnitzii SL3/3 draft genome	gi|479170689|ref|NC_021020.1| Faecalibacterium prausnitzii SL3/3 draft genome	gi|479170689|ref|NC_021020.1| Faecalibacterium prausnitzii SL3/3 draft genome
contig-100_1186	1146	N	1	0	NA	0	0	0	NA	NA	NA	NA
contig-100_1187	1144	N	1	0	NA	0	0	0	NA	NA	NA	NA

contig-100_1189	1144	N	1	0	NA	0	0	0	NA	Propionibacterium acnes HL096PA1, complete genome	gi|295129529|ref|NC_014039.1| Propionibacterium acnes SK137 chromosome, complete genome	gi|295129529|ref|NC_014039.1| Propionibacterium acnes SK137 chromosome, complete genome
contig-100_119	4655	N	6	0	NA	0	2	2	NA	Faecalibacterium prausnitzii L2/6 draft genome	gi|479208076|ref|NC_021042.1| Faecalibacterium prausnitzii L2-6, complete genome	gi|479208076|ref|NC_021042.1| Faecalibacterium prausnitzii L2-6, complete genome
contig-100_1190	1144	N	1	0	NA	0	0	0	NA	NA	NA	NA
contig-100_1191	1142	N	2	0	NA	0	0	0	NA	Uncultured bacterium xylooligosaccharide degrading DNA fragment, clone MetaPbio 3	NA	Uncultured bacterium xylooligosaccharide degrading DNA fragment, clone MetaPbio 3
contig-100_1192	1142	N	0	0	NA	0	0	0	NA	NA	NA	NA
contig-100_1193	1141	N	2	0	NA	0	0	0	NA	NA	NA	NA
contig-100_1194	1141	N	1	0	NA	0	0	0	NA	NA	NA	NA
contig-100_1196	1139	N	1	0	NA	0	0	0	NA	NA	NA	NA
contig-100_1197	1139	N	0	0	NA	0	0	0	NA	NA	NA	NA
contig-100_1198	1138	N	0	0	NA	0	0	0	NA	Uncultured bacterium clone LM0ABA27ZB05FM1 genomic sequence	NA	Uncultured bacterium clone LM0ABA27ZB05FM1 genomic sequence
contig-100_1200	1137	N	0	0	NA	0	0	0	NA	NA	NA	NA
contig-100_1201	1137	N	2	0	NA	0	0	0	NA	Bacteroides vulgatus ATCC 8482, complete genome	gi|150002608|ref|NC_009614.1| Bacteroides vulgatus ATCC 8482 chromosome, complete genome	gi|150002608|ref|NC_009614.1| Bacteroides vulgatus ATCC 8482 chromosome, complete genome
contig-100_1202	1136	N	1	0	NA	0	0	0	NA	NA	NA	NA

contig-100_1203	1136	N	1	0	NA	0	0	0	NA	NA	NA	NA
contig-100_1204	1135	N	2	0	NA	0	0	0	NA	Lactobacillus bacteriophage phage AQ113, complete genome	gi|525706521|ref|NC_021744.1| Lactobacillus helveticus CNRZ32, complete genome	gi|525706521|ref|NC_021744.1| Lactobacillus helveticus CNRZ32, complete genome
contig-100_1206	1135	N	1	0	NA	0	0	0	NA	Streptococcus phage YMC-2011, complete genome	gi|399498678|ref|NC_018285.1| Streptococcus phage YMC-2011, complete genome	gi|399498678|ref|NC_018285.1| Streptococcus phage YMC-2011, complete genome
contig-100_1207	1134	N	1	0	NA	0	0	0	NA	Haemophilus parainfluenzae T3T1 complete genome	gi|345428590|ref|NC_015964.1| Haemophilus parainfluenzae T3T1, complete genome	gi|345428590|ref|NC_015964.1| Haemophilus parainfluenzae T3T1, complete genome
contig-100_1208	1133	N	0	0	NA	0	0	0	NA	Bacteroides thetaiotaomicron VPI-5482, complete genome	gi|29345410|ref|NC_004663.1| Bacteroides thetaiotaomicron VPI-5482 chromosome, complete genome	gi|29345410|ref|NC_004663.1| Bacteroides thetaiotaomicron VPI-5482 chromosome, complete genome
contig-100_1209	1132	N	0	0	NA	0	0	0	NA	NA	NA	NA
contig-100_121	4571	N	5	0	NA	0	0	1	NA	Bacteroides vulgatus ATCC 8482, complete genome	gi|150002608|ref|NC_009614.1| Bacteroides vulgatus ATCC 8482 chromosome, complete genome	gi|150002608|ref|NC_009614.1| Bacteroides vulgatus ATCC 8482 chromosome, complete genome

contig-100_1211	1131	N	0	0	NA	0	0	0	NA	Haemophilus parainfluenzae T3T1 complete genome	gi|345428590|ref|NC_015964.1| Haemophilus parainfluenzae T3T1, complete genome	gi|345428590|ref|NC_015964.1| Haemophilus parainfluenzae T3T1, complete genome
contig-100_1213	1130	N	0	0	NA	0	0	0	NA	NA	NA	NA
contig-100_1215	1130	N	1	0	NA	0	0	0	NA	NA	NA	NA
contig-100_1218	1129	N	0	0	NA	0	0	0	NA	NA	NA	NA
contig-100_1219	1128	N	1	0	NA	0	0	0	NA	NA	NA	NA
contig-100_122	4558	N	4	1	Sipho	1	0	1	NA	NA	NA	NA
contig-100_1220	1128	N	0	0	NA	0	0	0	NA	NA	NA	NA
contig-100_1222	1126	N	0	0	NA	0	0	0	NA	NA	NA	NA
contig-100_1223	1126	N	1	0	NA	0	0	0	NA	NA	NA	NA
contig-100_1224	1125	N	0	0	NA	0	0	0	NA	NA	NA	NA
contig-100_1226	1124	N	1	0	NA	0	0	0	NA	NA	NA	NA
contig-100_1227	1124	N	1	0	NA	0	0	0	NA	NA	NA	NA
contig-100_1228	1123	N	2	0	NA	0	0	0	NA	Faecalibacterium prausnitzii L2/6 draft genome	gi|479208076|ref|NC_021042.1| Faecalibacterium prausnitzii L2-6, complete genome	gi|479208076|ref|NC_021042.1| Faecalibacterium prausnitzii L2-6, complete genome
contig-100_1229	1123	N	2	0	NA	0	0	0	NA	Faecalibacterium prausnitzii L2/6 draft genome	gi|479208076|ref|NC_021042.1| Faecalibacterium prausnitzii L2-6, complete genome	gi|479208076|ref|NC_021042.1| Faecalibacterium prausnitzii L2-6, complete genome
contig-100_1230	1123	N	0	0	NA	0	0	0	NA	NA	NA	NA
contig-100_1232	1122	N	1	0	NA	0	0	0	NA	Escherichia coli str. K-12 substr. MG1655, complete genome	gi|74310614|ref|NC_007384.1| Shigella sonnei Ss046 chromosome, complete genome	gi|74310614|ref|NC_007384.1| Shigella sonnei Ss046 chromosome, complete genome

contig-100_1233	1122	N	0	0	NA	0	0	0	NA	Alicycliphilus denitrificans BC, complete genome	gi|319760738|ref|NC_014910.1| Alicycliphilus denitrificans BC chromosome, complete genome	gi|319760738|ref|NC_014910.1| Alicycliphilus denitrificans BC chromosome, complete genome
contig-100_1234	1121	N	1	0	NA	0	0	0	NA	Uncultured organism clone 1041059767488 genomic sequence	NA	Uncultured organism clone 1041059767488 genomic sequence
contig-100_1235	1121	N	1	0	NA	0	0	0	NA	NA	NA	NA
contig-100_1236	1121	N	0	0	NA	0	0	0	NA	NA	gi|479336697|ref|NC_021047.1| Clostridium cf. saccharolyticum K10, complete genome	gi|479336697|ref|NC_021047.1| Clostridium cf. saccharolyticum K10, complete genome
contig-100_1238	1120	N	1	0	NA	0	0	0	NA	NA	NA	NA
contig-100_1239	1120	N	0	0	NA	0	0	0	NA	NA	NA	NA
contig-100_124	4533	N	5	0	NA	0	0	0	NA	Faecalibacterium prausnitzii SL3/3 draft genome	gi|479170689|ref|NC_021020.1| Faecalibacterium prausnitzii SL3/3 draft genome	gi|479170689|ref|NC_021020.1| Faecalibacterium prausnitzii SL3/3 draft genome
contig-100_1240	1120	N	0	0	NA	0	0	0	NA	Bacteroides vulgatus ATCC 8482, complete genome	gi|150002608|ref|NC_009614.1| Bacteroides vulgatus ATCC 8482 chromosome, complete genome	gi|150002608|ref|NC_009614.1| Bacteroides vulgatus ATCC 8482 chromosome, complete genome
contig-100_1241	1119	N	1	0	NA	0	0	0	NA	NA	NA	NA

contig-100_1242	1118	N	0	0	NA	0	0	0	NA	Bacteroides helcogenes P 36-108, complete genome	gi|319899888|ref|NC_014933.1| Bacteroides helcogenes P 36-108 chromosome, complete genome	gi|319899888|ref|NC_014933.1| Bacteroides helcogenes P 36-108 chromosome, complete genome
contig-100_1243	1117	N	1	0	NA	0	0	0	NA	Bacteroides xylanisolvens XB1A draft genome	gi|479162165|ref|NC_021017.1| Bacteroides xylanisolvens XB1A draft genome	gi|479162165|ref|NC_021017.1| Bacteroides xylanisolvens XB1A draft genome
contig-100_1244	1117	N	0	0	NA	0	0	0	NA	NA	NA	NA
contig-100_1245	1117	N	0	0	NA	0	0	0	NA	NA	NA	NA
contig-100_1247	1116	N	1	0	NA	0	0	0	NA	Haemophilus parainfluenzae T3T1 complete genome	gi|345428590|ref|NC_015964.1| Haemophilus parainfluenzae T3T1, complete genome	gi|345428590|ref|NC_015964.1| Haemophilus parainfluenzae T3T1, complete genome
contig-100_1248	1116	N	1	0	NA	0	0	0	NA	NA	NA	NA
contig-100_1249	1114	N	2	0	NA	0	0	0	NA	Faecalibacterium prausnitzii L2/6 draft genome	gi|479208076|ref|NC_021042.1| Faecalibacterium prausnitzii L2-6, complete genome	gi|479208076|ref|NC_021042.1| Faecalibacterium prausnitzii L2-6, complete genome
contig-100_125	4512	N	3	0	NA	0	0	0	NA	Uncultured organism clone 1041059765029 genomic sequence	gi|150002608|ref|NC_009614.1| Bacteroides vulgatus ATCC 8482 chromosome, complete genome	gi|150002608|ref|NC_009614.1| Bacteroides vulgatus ATCC 8482 chromosome, complete genome
contig-100_1250	1114	N	2	0	NA	0	0	0	NA	NA	NA	NA
contig-100_1251	1113	N	0	0	NA	0	0	0	NA	NA	NA	NA
contig-100_1252	1113	N	0	0	NA	0	0	0	NA	NA	NA	NA

contig-100_1253	1112	N	1	0	NA	0	0	0	NA	Bacteroides salanitronis DSM 18170, complete genome	gi|325297172|ref|NC_015164.1| Bacteroides salanitronis DSM 18170 chromosome, complete genome	gi|325297172|ref|NC_015164.1| Bacteroides salanitronis DSM 18170 chromosome, complete genome
contig-100_1254	1112	N	0	0	NA	0	0	0	NA	NA	NA	NA
contig-100_1256	1112	N	0	0	NA	0	0	0	NA	NA	NA	NA
contig-100_1257	1111	N	0	0	NA	0	0	0	NA	NA	NA	NA
contig-100_1258	1111	N	2	0	NA	0	0	0	NA	Bacteroides xylanisolvens XB1A draft genome	gi|479162165|ref|NC_021017.1| Bacteroides xylanisolvens XB1A draft genome	gi|479162165|ref|NC_021017.1| Bacteroides xylanisolvens XB1A draft genome
contig-100_1259	1110	N	1	0	NA	0	0	0	NA	Haemophilus parainfluenzae T3T1 complete genome	gi|345428590|ref|NC_015964.1| Haemophilus parainfluenzae T3T1, complete genome	gi|345428590|ref|NC_015964.1| Haemophilus parainfluenzae T3T1, complete genome
contig-100_1260	1110	N	1	0	NA	0	0	0	NA	NA	NA	NA
contig-100_1261	1110	N	0	0	NA	0	0	0	NA	Streptococcus pyogenes M1 GAS, complete genome	gi|94987631|ref|NC_008021.1| Streptococcus pyogenes MGAS9429 chromosome, complete genome	gi|94987631|ref|NC_008021.1| Streptococcus pyogenes MGAS9429 chromosome, complete genome
contig-100_1262	1109	N	1	0	NA	0	0	0	NA	Uncultured organism clone VC1BS40TF genomic sequence	NA	Uncultured organism clone VC1BS40TF genomic sequence
contig-100_1265	1107	N	1	0	NA	0	0	0	NA	NA	NA	NA

contig-100_1266	1107	N	0	0	NA	0	0	0	NA	Bacteroides salanitronis DSM 18170, complete genome	gi|325297172|ref|NC_015164.1| Bacteroides salanitronis DSM 18170 chromosome, complete genome	gi|325297172|ref|NC_015164.1| Bacteroides salanitronis DSM 18170 chromosome, complete genome
contig-100_1267	1107	N	0	0	NA	0	0	0	NA	NA	NA	NA
contig-100_1268	1105	N	1	0	NA	0	0	0	NA	NA	NA	NA
contig-100_1269	1104	N	1	0	NA	0	0	0	NA	Haemophilus parainfluenzae T3T1 complete genome	gi|345428590|ref|NC_015964.1| Haemophilus parainfluenzae T3T1, complete genome	gi|345428590|ref|NC_015964.1| Haemophilus parainfluenzae T3T1, complete genome
contig-100_127	4435	N	0	0	NA	0	0	0	NA	NA	NA	NA
contig-100_1270	1104	N	1	0	NA	0	0	0	NA	NA	NA	NA
contig-100_1271	1104	N	0	0	NA	0	0	0	NA	NA	NA	NA
contig-100_1272	1103	N	0	0	NA	0	0	0	NA	Bacteroides vulgatus ATCC 8482, complete genome	gi|150002608|ref|NC_009614.1| Bacteroides vulgatus ATCC 8482 chromosome, complete genome	gi|150002608|ref|NC_009614.1| Bacteroides vulgatus ATCC 8482 chromosome, complete genome
contig-100_1274	1102	N	1	0	NA	0	0	0	NA	NA	NA	NA
contig-100_1275	1101	N	1	0	NA	0	0	0	NA	NA	NA	NA
contig-100_1276	1101	N	0	0	NA	0	0	0	NA	Clostridiales sp. SM4/1 draft genome	gi|479181986|ref|NC_021024.1| Butyrate-producing bacterium SM4/1, complete genome	gi|479181986|ref|NC_021024.1| Butyrate-producing bacterium SM4/1, complete genome
contig-100_1277	1101	N	1	0	NA	0	0	0	NA	Uncultured bacterium clone LM0ABA39ZD09FM1 genomic sequence	NA	Uncultured bacterium clone LM0ABA39ZD09FM1 genomic sequence
contig-100_1278	1100	N	0	0	NA	0	0	0	NA	NA	NA	NA
contig-100_1279	1100	N	1	0	NA	0	0	0	NA	NA	NA	NA

contig-100_1280	1100	N	1	0	NA	0	0	0	NA	Uncultured organism clone VC1BY52TF genomic sequence	gi|479176048|ref|NC_021022.1| Ruminococcus obeum A2-162 draft genome	gi|479176048|ref|NC_021022.1| Ruminococcus obeum A2-162 draft genome
contig-100_1281	1098	N	1	0	NA	0	0	0	NA	NA	NA	NA
contig-100_1282	1098	N	0	0	NA	0	0	0	NA	NA	NA	NA
contig-100_1283	1098	N	2	0	NA	0	0	0	NA	NA	NA	NA
contig-100_1284	1098	N	1	0	NA	0	0	0	NA	NA	NA	NA
contig-100_1285	1098	N	1	0	NA	0	0	0	NA	Propionibacterium acnes 6609, complete genome	gi|50841496|ref|NC_006085.1| Propionibacterium acnes KPA171202 chromosome, complete genome	gi|50841496|ref|NC_006085.1| Propionibacterium acnes KPA171202 chromosome, complete genome
contig-100_1286	1097	N	1	0	NA	0	0	0	NA	NA	NA	NA
contig-100_1287	1097	N	1	0	NA	0	0	0	NA	NA	NA	NA
contig-100_1288	1097	N	0	0	NA	0	0	0	NA	NA	NA	NA
contig-100_1289	1096	N	0	0	NA	0	0	0	NA	Bacteroides fragilis conjugative transposon CTn341, complete sequence	gi|375253814|ref|NC_016610.1| Tannerella forsythia ATCC 43037 chromosome, complete genome	gi|375253814|ref|NC_016610.1| Tannerella forsythia ATCC 43037 chromosome, complete genome
contig-100_1291	1096	N	1	0	NA	0	0	0	NA	NA	NA	NA
contig-100_1293	1095	N	1	0	NA	0	0	0	NA	NA	NA	NA
contig-100_1295	1092	N	1	0	NA	0	0	0	NA	NA	NA	NA
contig-100_1296	1092	N	1	0	NA	0	0	0	NA	NA	NA	NA
contig-100_1297	1091	N	1	0	NA	0	0	0	NA	NA	NA	NA
contig-100_1298	1091	N	0	0	NA	0	0	0	NA	Bacteroides salanitronis DSM 18170, complete genome	gi|325297172|ref|NC_015164.1| Bacteroides salanitronis DSM 18170 chromosome, complete genome	gi|325297172|ref|NC_015164.1| Bacteroides salanitronis DSM 18170 chromosome, complete genome
contig-100_1299	1091	N	1	0	NA	0	0	0	NA	NA	NA	NA
contig-100_130	4387	N	5	1	NA	0	0	1	NA	NA	NA	NA

contig-100_1300	1091	N	1	0	NA	0	0	0	NA	Eubacterium siraeum 70/3 draft genome	gi|479143419|ref|NC_021011.1| Eubacterium siraeum 70/3 draft genome	gi|479143419|ref|NC_021011.1| Eubacterium siraeum 70/3 draft genome
contig-100_1301	1089	N	0	0	NA	0	0	0	NA	NA	NA	NA
contig-100_1302	1089	N	0	0	NA	0	0	0	NA	Bacteroides salanitronis DSM 18170, complete genome	gi|325297172|ref|NC_015164.1| Bacteroides salanitronis DSM 18170 chromosome, complete genome	gi|325297172|ref|NC_015164.1| Bacteroides salanitronis DSM 18170 chromosome, complete genome
contig-100_1303	1087	N	0	0	NA	0	0	0	NA	NA	gi|563711419|ref|NC_023004.1| Candidatus Saccharibacteria bacterium RAAC3_TM7_1, complete genome	gi|563711419|ref|NC_023004.1| Candidatus Saccharibacteria bacterium RAAC3_TM7_1, complete genome
contig-100_1304	1086	N	2	0	NA	0	0	0	NA	Uncultured organism clone VC1AO86TF genomic sequence	NA	Uncultured organism clone VC1AO86TF genomic sequence
contig-100_1305	1086	N	0	0	NA	0	0	0	NA	NA	NA	NA
contig-100_1306	1086	N	1	0	NA	0	0	0	NA	NA	NA	NA
contig-100_1307	1086	N	1	0	NA	0	0	0	NA	Bacteroides vulgatus ATCC 8482, complete genome	gi|150002608|ref|NC_009614.1| Bacteroides vulgatus ATCC 8482 chromosome, complete genome	gi|150002608|ref|NC_009614.1| Bacteroides vulgatus ATCC 8482 chromosome, complete genome
contig-100_1308	1085	N	1	0	NA	0	0	0	NA	Faecalibacterium prausnitzii L2/6 draft genome	gi|479208076|ref|NC_021042.1| Faecalibacterium prausnitzii L2-6, complete genome	gi|479208076|ref|NC_021042.1| Faecalibacterium prausnitzii L2-6, complete genome

contig-100_1309	1085	N	2	0	NA	0	0	0	NA	Coprococcus catus GD/7 draft genome	gi|479136967|ref|NC_021009.1| Coprococcus catus GD/7 draft genome	gi|479136967|ref|NC_021009.1| Coprococcus catus GD/7 draft genome
contig-100_131	4364	N	4	1	Sipho	0	0	1	NA	NA	NA	NA
contig-100_1310	1085	N	1	0	NA	0	0	0	NA	NA	NA	NA
contig-100_1311	1085	N	0	0	NA	0	0	0	NA	NA	NA	NA
contig-100_1312	1085	N	1	0	NA	0	0	0	NA	NA	NA	NA
contig-100_1314	1084	N	1	0	NA	0	0	0	NA	NA	NA	NA
contig-100_1315	1081	N	0	0	NA	0	0	0	NA	NA	NA	NA
contig-100_1316	1081	N	0	0	NA	0	0	0	NA	NA	NA	NA
contig-100_1317	1080	N	1	0	NA	0	0	0	NA	NA	NA	NA
contig-100_1318	1078	N	2	0	NA	0	0	1	NA	Clostridiales sp. SSC/2 draft genome	gi|479158859|ref|NC_021016.1| Butyrate-producing bacterium SSC/2, complete genome	gi|479158859|ref|NC_021016.1| Butyrate-producing bacterium SSC/2, complete genome
contig-100_1319	1078	N	0	0	NA	0	0	0	NA	Lactobacillus helveticus CNRZ32, complete genome	gi|525706521|ref|NC_021744.1| Lactobacillus helveticus CNRZ32, complete genome	gi|525706521|ref|NC_021744.1| Lactobacillus helveticus CNRZ32, complete genome
contig-100_1321	1077	N	1	0	NA	0	0	0	NA	NA	NA	NA
contig-100_1322	1076	N	0	0	NA	0	0	0	NA	Bacteroides salanitronis DSM 18170, complete genome	gi|325297172|ref|NC_015164.1| Bacteroides salanitronis DSM 18170 chromosome, complete genome	gi|325297172|ref|NC_015164.1| Bacteroides salanitronis DSM 18170 chromosome, complete genome
contig-100_1323	1076	N	1	0	NA	0	0	0	NA	NA	NA	NA
contig-100_1324	1076	N	2	0	NA	0	0	0	NA	NA	NA	NA
contig-100_1325	1076	N	1	0	NA	0	0	0	NA	NA	NA	NA
contig-100_1326	1075	N	1	0	NA	0	0	0	NA	NA	NA	NA
contig-100_1327	1075	N	0	0	NA	0	0	0	NA	NA	NA	NA
contig-100_1329	1075	N	0	0	NA	0	0	0	NA	NA	NA	NA
contig-100_133	4305	N	4	0	NA	0	0	2	NA	NA	NA	NA
contig-100_1330	1075	N	1	0	NA	0	0	0	NA	NA	NA	NA

contig-100_1331	1074	N	1	0	NA	0	0	0	NA	Bacteroides vulgatus ATCC 8482, complete genome	gi|150002608|ref|NC_009614.1| Bacteroides vulgatus ATCC 8482 chromosome, complete genome	gi|150002608|ref|NC_009614.1| Bacteroides vulgatus ATCC 8482 chromosome, complete genome
contig-100_1332	1073	N	1	0	NA	0	0	0	NA	Haemophilus parainfluenzae T3T1 complete genome	gi|345428590|ref|NC_015964.1| Haemophilus parainfluenzae T3T1, complete genome	gi|345428590|ref|NC_015964.1| Haemophilus parainfluenzae T3T1, complete genome
contig-100_1333	1071	N	0	0	NA	0	0	0	NA	NA	NA	NA
contig-100_1335	1071	N	2	0	NA	0	0	0	NA	NA	NA	NA
contig-100_1336	1069	N	1	0	NA	0	0	0	NA	Odoribacter splanchnicus DSM 20712, complete genome	gi|325278757|ref|NC_015160.1| Odoribacter splanchnicus DSM 20712 chromosome, complete genome	gi|325278757|ref|NC_015160.1| Odoribacter splanchnicus DSM 20712 chromosome, complete genome
contig-100_1337	1069	N	0	0	NA	0	0	0	NA	NA	NA	NA
contig-100_1338	1068	N	1	0	NA	0	0	0	NA	NA	NA	NA
contig-100_1339	1068	N	0	0	NA	0	0	0	NA	NA	NA	NA
contig-100_134	4283	N	4	0	NA	0	1	1	NA	Klebsiella pneumoniae subsp. pneumoniae MGH 78578, complete sequence	gi|550443072|ref|NC_022566.1| Klebsiella pneumoniae CG43, complete genome	gi|550443072|ref|NC_022566.1| Klebsiella pneumoniae CG43, complete genome
contig-100_1340	1067	N	1	0	NA	0	0	0	NA	NA	NA	NA
contig-100_1341	1066	N	0	0	NA	0	0	0	NA	NA	NA	NA
contig-100_1343	1066	N	1	0	NA	0	0	0	NA	NA	NA	NA
contig-100_1344	1065	N	2	0	NA	0	0	0	NA	NA	NA	NA
contig-100_1345	1065	N	1	0	NA	0	0	0	NA	NA	NA	NA
contig-100_1346	1064	N	0	0	NA	0	0	0	NA	NA	NA	NA
contig-100_1347	1064	N	1	0	NA	0	0	0	NA	NA	NA	NA

contig-100_1348	1063	N	1	0	NA	0	0	0	NA	Eubacterium siraeum V10Sc8a draft genome	gi|479210985|ref|NC_021043.1| Eubacterium siraeum V10Sc8a draft genome	gi|479210985|ref|NC_021043.1| Eubacterium siraeum V10Sc8a draft genome
contig-100_1349	1063	N	0	0	NA	0	0	0	NA	NA	NA	NA
contig-100_1350	1063	N	1	0	NA	0	0	0	NA	NA	NA	NA
contig-100_1351	1062	N	1	0	NA	0	0	0	NA	NA	NA	NA
contig-100_1352	1062	N	1	0	NA	0	0	0	NA	Coprococcus catus GD/7 draft genome	gi|479136967|ref|NC_021009.1| Coprococcus catus GD/7 draft genome	gi|479136967|ref|NC_021009.1| Coprococcus catus GD/7 draft genome
contig-100_1353	1062	N	1	0	NA	0	0	0	NA	NA	NA	NA
contig-100_1354	1062	N	1	0	NA	0	0	0	NA	NA	NA	NA
contig-100_1355	1062	N	0	0	NA	0	0	0	NA	NA	NA	NA
contig-100_1356	1061	N	1	0	NA	0	0	0	NA	Bacteroides thetaiotaomicron VPI-5482, complete genome	gi|29345410|ref|NC_004663.1| Bacteroides thetaiotaomicron VPI-5482 chromosome, complete genome	gi|29345410|ref|NC_004663.1| Bacteroides thetaiotaomicron VPI-5482 chromosome, complete genome
contig-100_1357	1061	N	1	0	NA	0	0	0	NA	NA	NA	NA
contig-100_1358	1060	N	0	0	NA	0	0	0	NA	Clostridiales sp. SM4/1 draft genome	gi|479181986|ref|NC_021024.1| Butyrate-producing bacterium SM4/1, complete genome	gi|479181986|ref|NC_021024.1| Butyrate-producing bacterium SM4/1, complete genome
contig-100_1360	1060	N	1	0	NA	0	0	0	NA	Propionibacterium acnes ATCC 11828, complete genome	gi|386069650|ref|NC_017550.1| Propionibacterium acnes ATCC 11828 chromosome, complete genome	gi|386069650|ref|NC_017550.1| Propionibacterium acnes ATCC 11828 chromosome, complete genome
contig-100_1361	1059	N	0	0	NA	0	0	0	NA	NA	NA	NA
contig-100_1362	1059	N	0	0	NA	0	0	0	NA	NA	NA	NA
contig-100_1363	1059	N	1	0	NA	0	0	0	NA	NA	NA	NA

contig-100_1364	1059	N	1	0	NA	0	0	0	NA	NA	NA	NA
contig-100_1365	1059	N	0	0	NA	0	0	0	NA	NA	NA	NA
contig-100_1366	1058	N	0	0	NA	0	0	0	NA	NA	NA	NA
contig-100_1368	1058	N	0	0	NA	0	0	0	NA	Corynebacterium efficiens YS-314 DNA, complete genome	gi|25026556|ref|NC_004369.1| Corynebacterium efficiens YS-314 chromosome, complete genome	gi|25026556|ref|NC_004369.1| Corynebacterium efficiens YS-314 chromosome, complete genome
contig-100_1369	1057	N	3	0	NA	0	0	0	NA	NA	NA	NA
contig-100_137	4255	N	4	0	NA	0	0	0	NA	NA	NA	NA
contig-100_1370	1057	N	0	0	NA	0	0	0	NA	NA	NA	NA
contig-100_1371	1056	N	1	0	NA	0	0	0	NA	Bacteroides vulgatus ATCC 8482, complete genome	gi|150002608|ref|NC_009614.1| Bacteroides vulgatus ATCC 8482 chromosome, complete genome	gi|150002608|ref|NC_009614.1| Bacteroides vulgatus ATCC 8482 chromosome, complete genome
contig-100_1372	1056	N	1	0	NA	0	0	0	NA	NA	NA	NA
contig-100_1374	1055	N	0	0	NA	0	0	0	NA	NA	NA	NA
contig-100_1375	1055	N	0	0	NA	0	0	0	NA	NA	NA	NA
contig-100_1376	1055	N	0	0	NA	0	0	0	NA	Clostridiales sp. SSC/2 draft genome	gi|479158859|ref|NC_021016.1| Butyrate-producing bacterium SSC/2, complete genome	gi|479158859|ref|NC_021016.1| Butyrate-producing bacterium SSC/2, complete genome
contig-100_1377	1054	N	2	0	NA	0	0	0	NA	NA	NA	NA
contig-100_138	4255	N	3	2	Sipho	1	0	2	NA	NA	NA	NA
contig-100_1380	1053	N	0	0	NA	0	0	0	NA	Aggregatibacter phage S1249, complete sequence	gi|273809542|ref|NC_013597.1| Aggregatibacter phage S1249, complete sequence	gi|273809542|ref|NC_013597.1| Aggregatibacter phage S1249, complete sequence

contig-100_1381	1051	N	0	0	NA	0	0	0	NA	Uncultured organism clone 1041059767705 genomic sequence	gi|319899888|ref|NC_014933.1| Bacteroides helcogenes P 36-108 chromosome, complete genome	gi|319899888|ref|NC_014933.1| Bacteroides helcogenes P 36-108 chromosome, complete genome
contig-100_1382	1051	N	1	0	NA	0	0	0	NA	NA	gi|549482487|ref|NC_022526.1| Bacteroides sp. CF50, complete genome	gi|549482487|ref|NC_022526.1| Bacteroides sp. CF50, complete genome
contig-100_1383	1050	N	1	0	NA	0	0	0	NA	Faecalibacterium prausnitzii SL3/3 draft genome	gi|479170689|ref|NC_021020.1| Faecalibacterium prausnitzii SL3/3 draft genome	gi|479170689|ref|NC_021020.1| Faecalibacterium prausnitzii SL3/3 draft genome
contig-100_1384	1050	N	0	0	NA	0	0	0	NA	NA	NA	NA
contig-100_1385	1050	N	1	0	NA	0	0	0	NA	Bacteroides vulgatus ATCC 8482, complete genome	gi|150002608|ref|NC_009614.1| Bacteroides vulgatus ATCC 8482 chromosome, complete genome	gi|150002608|ref|NC_009614.1| Bacteroides vulgatus ATCC 8482 chromosome, complete genome
contig-100_1386	1050	N	0	0	NA	0	0	0	NA	NA	NA	NA
contig-100_1388	1049	N	1	0	NA	0	0	0	NA	NA	NA	NA
contig-100_139	4198	N	2	1	Sipho	0	0	1	NA	NA	NA	NA
contig-100_1390	1049	N	0	0	NA	0	0	0	NA	NA	NA	NA
contig-100_1391	1049	N	1	0	NA	0	0	0	NA	Bacteroides vulgatus ATCC 8482, complete genome	gi|150002608|ref|NC_009614.1| Bacteroides vulgatus ATCC 8482 chromosome, complete genome	gi|150002608|ref|NC_009614.1| Bacteroides vulgatus ATCC 8482 chromosome, complete genome
contig-100_1392	1049	N	0	0	NA	0	0	0	NA	NA	NA	NA

contig-100_1393	1048	N	0	0	NA	0	0	0	NA	Bacteroides fragilis NCTC 9343, complete genome	gi|60679597|ref|NC_003228.3| Bacteroides fragilis NCTC 9343, complete genome	gi|60679597|ref|NC_003228.3| Bacteroides fragilis NCTC 9343, complete genome
contig-100_1394	1048	N	0	0	NA	0	0	0	NA	NA	NA	NA
contig-100_1396	1047	N	0	0	NA	0	0	0	NA	NA	NA	NA
contig-100_1398	1046	N	2	0	NA	0	0	0	NA	NA	NA	NA
contig-100_1399	1046	N	0	0	NA	0	0	0	NA	NA	NA	NA
contig-100_14	19314	N	18	8	Sipho	1	1	9	NA	NA	NA	NA
contig-100_1401	1045	N	1	0	NA	0	0	0	NA	NA	NA	NA
contig-100_1402	1045	N	1	0	NA	0	0	0	NA	NA	NA	NA
contig-100_1403	1044	N	0	0	NA	0	0	0	NA	Propionibacterium acnes ATCC 11828, complete genome	gi|386069650|ref|NC_017550.1| Propionibacterium acnes ATCC 11828 chromosome, complete genome	gi|386069650|ref|NC_017550.1| Propionibacterium acnes ATCC 11828 chromosome, complete genome
contig-100_1404	1044	N	1	0	NA	0	0	0	NA	NA	NA	NA
contig-100_1405	1044	N	0	0	NA	0	0	0	NA	NA	NA	NA
contig-100_1406	1044	N	0	0	NA	0	0	0	NA	NA	NA	NA
contig-100_1407	1043	N	2	0	NA	0	0	0	NA	Faecalibacterium prausnitzii SL3/3 draft genome	gi|479170689|ref|NC_021020.1| Faecalibacterium prausnitzii SL3/3 draft genome	gi|479170689|ref|NC_021020.1| Faecalibacterium prausnitzii SL3/3 draft genome
contig-100_1408	1043	N	1	0	NA	0	0	0	NA	NA	NA	NA
contig-100_1409	1043	N	0	0	NA	0	0	0	NA	NA	NA	NA
contig-100_141	4178	N	3	2	Sipho	1	0	2	NA	Streptococcus phage 040922	gi|556587607|ref|NC_022665.1| Streptococcus suis T15, complete genome	gi|556587607|ref|NC_022665.1| Streptococcus suis T15, complete genome
contig-100_1410	1041	N	1	0	NA	0	0	0	NA	NA	NA	NA

contig-100_1411	1041	N	0	0	NA	0	0	0	NA	Uncultured organism clone 1041059765405 genomic sequence	gi|53711291|ref|NC_006347.1| Bacteroides fragilis YCH46 DNA, complete genome	gi|53711291|ref|NC_006347.1| Bacteroides fragilis YCH46 DNA, complete genome
contig-100_1412	1041	N	1	0	NA	0	0	0	NA	NA	NA	NA
contig-100_1413	1041	N	1	0	NA	0	0	0	NA	NA	NA	NA
contig-100_1414	1040	N	0	0	NA	0	0	0	NA	Bacteroides vulgatus ATCC 8482, complete genome	gi|150002608|ref|NC_009614.1| Bacteroides vulgatus ATCC 8482 chromosome, complete genome	gi|150002608|ref|NC_009614.1| Bacteroides vulgatus ATCC 8482 chromosome, complete genome
contig-100_1415	1040	N	1	0	NA	0	0	0	NA	Propionibacterium acnes 6609, complete genome	gi|50841496|ref|NC_006085.1| Propionibacterium acnes KPA171202 chromosome, complete genome	gi|50841496|ref|NC_006085.1| Propionibacterium acnes KPA171202 chromosome, complete genome
contig-100_1416	1039	N	1	0	NA	0	0	0	NA	NA	NA	NA
contig-100_1417	1039	N	1	0	NA	0	0	0	NA	Parabacteroides distasonis ATCC 8503, complete genome	gi|150006674|ref|NC_009615.1| Parabacteroides distasonis ATCC 8503 chromosome, complete genome	gi|150006674|ref|NC_009615.1| Parabacteroides distasonis ATCC 8503 chromosome, complete genome
contig-100_1418	1039	N	0	0	NA	0	0	0	NA	NA	NA	NA
contig-100_1419	1038	N	0	0	NA	0	0	0	NA	NA	NA	NA
contig-100_1420	1037	N	0	0	NA	0	0	0	NA	Bacteroides fragilis YCH46 DNA, complete genome	gi|53711291|ref|NC_006347.1| Bacteroides fragilis YCH46 DNA, complete genome	gi|53711291|ref|NC_006347.1| Bacteroides fragilis YCH46 DNA, complete genome
contig-100_1422	1037	N	0	0	NA	0	0	0	NA	NA	NA	NA

contig-100_1423	1036	N	1	0	NA	0	0	0	NA	Pseudomonas fluorescens Pf29Arp contig091, whole genome shotgun sequence	gi|251787652|ref|NC_012912.1| Dickeya zeae Ech1591 chromosome, complete genome	gi|251787652|ref|NC_012912.1| Dickeya zeae Ech1591 chromosome, complete genome
contig-100_1424	1036	N	1	0	NA	0	0	0	NA	Bacteroides vulgatus ATCC 8482, complete genome	gi|150002608|ref|NC_009614.1| Bacteroides vulgatus ATCC 8482 chromosome, complete genome	gi|150002608|ref|NC_009614.1| Bacteroides vulgatus ATCC 8482 chromosome, complete genome
contig-100_1426	1036	N	1	0	NA	0	0	0	NA	NA	NA	NA
contig-100_1427	1036	N	0	0	NA	0	0	0	NA	Clostridiales sp. SSC/2 draft genome	gi|479158859|ref|NC_021016.1| Butyrate-producing bacterium SSC/2, complete genome	gi|479158859|ref|NC_021016.1| Butyrate-producing bacterium SSC/2, complete genome
contig-100_1428	1036	N	0	0	NA	0	0	0	NA	NA	NA	NA
contig-100_1429	1035	N	0	0	NA	0	0	0	NA	NA	NA	NA
contig-100_1430	1035	N	0	0	NA	0	0	0	NA	NA	NA	NA
contig-100_1433	1034	N	1	0	NA	0	0	0	NA	NA	NA	NA
contig-100_1434	1033	N	0	0	NA	0	0	0	NA	Uncultured bacterium clone zdt-45e5, complete sequence	gi|563711419|ref|NC_023004.1| Candidatus Saccharibacteria bacterium RAAC3_TM7_1, complete genome	gi|563711419|ref|NC_023004.1| Candidatus Saccharibacteria bacterium RAAC3_TM7_1, complete genome
contig-100_1435	1033	N	1	0	NA	0	0	0	NA	Bacteroides vulgatus ATCC 8482, complete genome	gi|150002608|ref|NC_009614.1| Bacteroides vulgatus ATCC 8482 chromosome, complete genome	gi|150002608|ref|NC_009614.1| Bacteroides vulgatus ATCC 8482 chromosome, complete genome
contig-100_1436	1032	N	0	0	NA	0	0	0	NA	NA	NA	NA
contig-100_1437	1031	N	2	0	NA	0	0	0	NA	NA	NA	NA
contig-100_1438	1031	N	0	0	NA	0	0	0	NA	NA	NA	NA

contig-100_1439	1031	N	1	0	NA	0	0	0	NA	Bifidobacterium breve ACS-071-V-Sch8b, complete genome	gi|479158859|ref|NC_021016.1| Butyrate-producing bacterium SSC/2, complete genome	gi|479158859|ref|NC_021016.1| Butyrate-producing bacterium SSC/2, complete genome
contig-100_144	4133	N	4	3	Sipho	1	0	3	NA	Clostridium phytofermentans ISDg, complete genome	gi|160878162|ref|NC_010001.1| Clostridium phytofermentans ISDg chromosome, complete genome	gi|160878162|ref|NC_010001.1| Clostridium phytofermentans ISDg chromosome, complete genome
contig-100_1440	1031	N	0	0	NA	0	0	0	NA	NA	NA	NA
contig-100_1441	1031	N	0	0	NA	0	0	0	NA	Faecalibacterium prausnitzii L2/6 draft genome	gi|479208076|ref|NC_021042.1| Faecalibacterium prausnitzii L2-6, complete genome	gi|479208076|ref|NC_021042.1| Faecalibacterium prausnitzii L2-6, complete genome
contig-100_1442	1030	N	1	0	NA	0	0	0	NA	NA	NA	NA
contig-100_1443	1029	N	1	0	NA	0	0	0	NA	NA	NA	NA
contig-100_1446	1028	N	1	0	NA	0	0	0	NA	NA	NA	NA
contig-100_1447	1028	N	0	0	NA	0	0	0	NA	NA	NA	NA
contig-100_1448	1026	N	1	0	NA	0	0	0	NA	NA	NA	NA
contig-100_1449	1026	N	0	0	NA	0	0	0	NA	Bacteroides salanitronis DSM 18170, complete genome	gi|325297172|ref|NC_015164.1| Bacteroides salanitronis DSM 18170 chromosome, complete genome	gi|325297172|ref|NC_015164.1| Bacteroides salanitronis DSM 18170 chromosome, complete genome
contig-100_145	4124	N	2	0	NA	0	0	0	NA	Bacteroides vulgatus ATCC 8482, complete genome	gi|150002608|ref|NC_009614.1| Bacteroides vulgatus ATCC 8482 chromosome, complete genome	gi|150002608|ref|NC_009614.1| Bacteroides vulgatus ATCC 8482 chromosome, complete genome
contig-100_1451	1026	N	0	0	NA	0	0	0	NA	NA	NA	NA

contig-100_1453	1024	N	0	0	NA	0	0	0	NA	Clostridium saccharolyticum-like K10 draft genome	gi|479336697|ref|NC_021047.1| Clostridium cf. saccharolyticum K10, complete genome	gi|479336697|ref|NC_021047.1| Clostridium cf. saccharolyticum K10, complete genome
contig-100_1454	1024	N	1	0	NA	0	0	0	NA	Faecalibacterium prausnitzii L2/6 draft genome	gi|479208076|ref|NC_021042.1| Faecalibacterium prausnitzii L2-6, complete genome	gi|479208076|ref|NC_021042.1| Faecalibacterium prausnitzii L2-6, complete genome
contig-100_1455	1024	N	2	0	NA	0	0	0	NA	NA	NA	NA
contig-100_1456	1024	N	1	0	NA	0	0	0	NA	Roseburia hominis A2-183, complete genome	gi|347530298|ref|NC_015977.1| Roseburia hominis A2-183 chromosome, complete genome	gi|347530298|ref|NC_015977.1| Roseburia hominis A2-183 chromosome, complete genome
contig-100_1457	1023	N	0	0	NA	0	0	0	NA	NA	NA	NA
contig-100_1458	1022	N	0	0	NA	0	0	0	NA	Eubacterium siraeum V10Sc8a draft genome	gi|479210985|ref|NC_021043.1| Eubacterium siraeum V10Sc8a draft genome	gi|479210985|ref|NC_021043.1| Eubacterium siraeum V10Sc8a draft genome
contig-100_1459	1022	N	0	0	NA	0	0	0	NA	Bacteroides vulgatus ATCC 8482, complete genome	gi|150002608|ref|NC_009614.1| Bacteroides vulgatus ATCC 8482 chromosome, complete genome	gi|150002608|ref|NC_009614.1| Bacteroides vulgatus ATCC 8482 chromosome, complete genome

contig-100_1460	1022	N	1	0	NA	0	0	0	NA	Clostridium lentocellum DSM 5427, complete genome	gi|326789139|ref|NC_015275.1| Clostridium lentocellum DSM 5427 chromosome, complete genome	gi|326789139|ref|NC_015275.1| Clostridium lentocellum DSM 5427 chromosome, complete genome
contig-100_1461	1022	N	1	0	NA	0	0	0	NA	NA	NA	NA
contig-100_1462	1022	N	0	0	NA	0	0	0	NA	NA	NA	NA
contig-100_1463	1022	N	0	0	NA	0	0	0	NA	Faecalibacterium prausnitzii SL3/3 draft genome	gi|479170689|ref|NC_021020.1| Faecalibacterium prausnitzii SL3/3 draft genome	gi|479170689|ref|NC_021020.1| Faecalibacterium prausnitzii SL3/3 draft genome
contig-100_1464	1021	N	2	0	NA	0	0	0	NA	Uncultured organism clone 1041059764970 genomic sequence	NA	Uncultured organism clone 1041059764970 genomic sequence
contig-100_1465	1021	N	2	0	NA	0	0	0	NA	Odoribacter splanchnicus DSM 20712, complete genome	gi|325278757|ref|NC_015160.1| Odoribacter splanchnicus DSM 20712 chromosome, complete genome	gi|325278757|ref|NC_015160.1| Odoribacter splanchnicus DSM 20712 chromosome, complete genome
contig-100_1466	1021	N	1	0	NA	0	0	0	NA	NA	NA	NA
contig-100_1467	1021	N	2	0	NA	0	0	0	NA	Faecalibacterium prausnitzii SL3/3 draft genome	gi|479170689|ref|NC_021020.1| Faecalibacterium prausnitzii SL3/3 draft genome	gi|479170689|ref|NC_021020.1| Faecalibacterium prausnitzii SL3/3 draft genome
contig-100_1468	1021	N	0	0	NA	0	0	0	NA	Uncultured organism clone 1041059765741 genomic sequence	NA	Uncultured organism clone 1041059765741 genomic sequence
contig-100_1469	1020	N	0	0	NA	0	0	0	NA	NA	NA	NA
contig-100_1471	1019	N	1	0	NA	0	0	0	NA	NA	NA	NA

contig-100_1472	1019	N	2	0	NA	0	0	0	NA	Ruminococcus torques L2-14 draft genome	gi|479155735|ref|NC_021015.1| Ruminococcus torques L2-14 draft genome	gi|479155735|ref|NC_021015.1| Ruminococcus torques L2-14 draft genome
contig-100_1473	1018	N	1	0	NA	0	0	0	NA	NA	NA	NA
contig-100_1474	1018	N	1	0	NA	0	0	0	NA	NA	NA	NA
contig-100_1475	1018	N	0	0	NA	0	0	0	NA	NA	NA	NA
contig-100_1476	1018	N	1	0	NA	0	0	0	NA	Faecalibacterium prausnitzii L2/6 draft genome	gi|479208076|ref|NC_021042.1| Faecalibacterium prausnitzii L2-6, complete genome	gi|479208076|ref|NC_021042.1| Faecalibacterium prausnitzii L2-6, complete genome
contig-100_1477	1018	N	1	0	NA	0	0	0	NA	NA	NA	NA
contig-100_1478	1017	N	0	0	NA	0	0	0	NA	Uncultured bacterium clone LM0ACA22ZG04FM1 genomic sequence	NA	Uncultured bacterium clone LM0ACA22ZG04FM1 genomic sequence
contig-100_1479	1017	N	0	0	NA	0	0	0	NA	NA	NA	NA
contig-100_1480	1017	N	1	0	NA	0	0	0	NA	NA	NA	NA
contig-100_1481	1016	N	1	0	NA	0	0	0	NA	NA	NA	NA
contig-100_1482	1014	N	0	0	NA	0	0	0	NA	NA	NA	NA
contig-100_1483	1014	N	0	0	NA	0	0	0	NA	NA	NA	NA
contig-100_1484	1014	N	0	0	NA	0	0	0	NA	NA	NA	NA
contig-100_1485	1014	N	1	0	NA	0	0	0	NA	NA	NA	NA
contig-100_1486	1014	N	0	0	NA	0	0	0	NA	NA	NA	NA
contig-100_1487	1014	N	1	0	NA	0	0	0	NA	NA	NA	NA
contig-100_1488	1013	N	2	0	NA	0	0	0	NA	NA	NA	NA
contig-100_1489	1013	N	0	0	NA	0	0	0	NA	NA	NA	NA
contig-100_149	4096	N	2	1	Sipho	0	0	1	NA	NA	NA	NA
contig-100_1490	1013	N	0	0	NA	0	0	0	NA	Uncultured bacterium clone LM0ACA2ZH05FM1 genomic sequence	NA	Uncultured bacterium clone LM0ACA2ZH05FM1 genomic sequence
contig-100_1491	1012	N	0	0	NA	0	0	0	NA	NA	NA	NA
contig-100_1492	1012	N	0	0	NA	0	0	0	NA	NA	NA	NA

contig-100_1493	1012	N	0	0	NA	0	0	0	NA	NA	NA	NA
contig-100_1494	1012	N	0	0	NA	0	0	0	NA	Bacteroides xylanisolvens XB1A draft genome	gi|479162165|ref|NC_021017.1| Bacteroides xylanisolvens XB1A draft genome	gi|479162165|ref|NC_021017.1| Bacteroides xylanisolvens XB1A draft genome
contig-100_1495	1010	N	1	0	NA	0	0	0	NA	Faecalibacterium prausnitzii L2/6 draft genome	gi|479208076|ref|NC_021042.1| Faecalibacterium prausnitzii L2-6, complete genome	gi|479208076|ref|NC_021042.1| Faecalibacterium prausnitzii L2-6, complete genome
contig-100_1496	1010	N	0	0	NA	0	0	0	NA	NA	NA	NA
contig-100_1497	1009	N	2	0	NA	0	0	0	NA	Clostridiales sp. SSC/2 draft genome	gi|479158859|ref|NC_021016.1| Butyrate-producing bacterium SSC/2, complete genome	gi|479158859|ref|NC_021016.1| Butyrate-producing bacterium SSC/2, complete genome
contig-100_1498	1009	N	1	0	NA	0	0	0	NA	Bacteroides vulgatus ATCC 8482, complete genome	gi|150002608|ref|NC_009614.1| Bacteroides vulgatus ATCC 8482 chromosome, complete genome	gi|150002608|ref|NC_009614.1| Bacteroides vulgatus ATCC 8482 chromosome, complete genome
contig-100_1499	1008	N	1	0	NA	0	0	0	NA	Eubacterium eligens ATCC 27750 plasmid, complete sequence	gi|238921767|ref|NC_012780.1| Eubacterium eligens ATCC 27750 plasmid unnamed, complete sequence	gi|238921767|ref|NC_012780.1| Eubacterium eligens ATCC 27750 plasmid unnamed, complete sequence
contig-100_15	18365	N	9	5	Sipho	2	0	5	NA	NA	NA	NA

contig-100_150	4094	N	2	0	NA	0	0	0	NA	Burkholderia pseudomallei 1026b chromosome 1, complete sequence	gi|556574580|ref|NC_022659.1| Burkholderia pseudomallei NCTC 13179 chromosome 1, complete sequence	gi|556574580|ref|NC_022659.1| Burkholderia pseudomallei NCTC 13179 chromosome 1, complete sequence
contig-100_1500	1008	N	3	0	NA	0	0	0	NA	Unidentified phage clone 1013_scaffold47 genomic sequence	NA	Unidentified phage clone 1013_scaffold47 genomic sequence
contig-100_1501	1006	N	0	0	NA	0	0	0	NA	Bacteroides vulgatus ATCC 8482, complete genome	gi|150002608|ref|NC_009614.1| Bacteroides vulgatus ATCC 8482 chromosome, complete genome	gi|150002608|ref|NC_009614.1| Bacteroides vulgatus ATCC 8482 chromosome, complete genome
contig-100_1502	1006	N	2	0	NA	0	0	0	NA	Uncultured organism clone VC1AF49TR genomic sequence	NA	Uncultured organism clone VC1AF49TR genomic sequence
contig-100_1503	1006	N	0	0	NA	0	0	0	NA	NA	NA	NA
contig-100_1504	1006	N	2	0	NA	0	0	0	NA	NA	NA	NA
contig-100_1505	1006	N	1	0	NA	0	0	0	NA	NA	NA	NA
contig-100_1506	1006	N	3	0	NA	0	0	0	NA	NA	NA	NA
contig-100_1507	1005	N	0	0	NA	0	0	0	NA	NA	NA	NA
contig-100_1508	1003	N	0	0	NA	0	0	0	NA	NA	NA	NA
contig-100_1509	1003	N	0	0	NA	0	0	0	NA	Klebsiella pneumoniae KCTC 2242, complete genome	gi|550443072|ref|NC_022566.1| Klebsiella pneumoniae CG43, complete genome	gi|550443072|ref|NC_022566.1| Klebsiella pneumoniae CG43, complete genome
contig-100_1510	1003	N	1	0	NA	0	0	0	NA	NA	NA	NA

contig-100_1511	1003	N	1	0	NA	0	0	0	NA	Bacteroides vulgatus ATCC 8482, complete genome	gi|150002608|ref|NC_009614.1| Bacteroides vulgatus ATCC 8482 chromosome, complete genome	gi|150002608|ref|NC_009614.1| Bacteroides vulgatus ATCC 8482 chromosome, complete genome
contig-100_1515	1001	N	1	0	NA	0	0	0	NA	Faecalibacterium prausnitzii L2/6 draft genome	gi|479208076|ref|NC_021042.1| Faecalibacterium prausnitzii L2-6, complete genome	gi|479208076|ref|NC_021042.1| Faecalibacterium prausnitzii L2-6, complete genome
contig-100_1516	1000	N	1	0	NA	0	0	0	NA	NA	NA	NA
contig-100_1517	1000	N	3	0	NA	0	0	0	NA	NA	NA	NA
contig-100_1518	1000	N	1	0	NA	0	0	0	NA	Methylobacillus flagellatus KT, complete genome	gi|91774356|ref|NC_007947.1| Methylobacillus flagellatus KT, complete genome	gi|91774356|ref|NC_007947.1| Methylobacillus flagellatus KT, complete genome
contig-100_1519	1000	N	2	0	NA	0	0	0	NA	NA	NA	NA
contig-100_152	4078	N	5	1	Sipho	0	0	1	NA	Faecalibacterium prausnitzii SL3/3 draft genome	gi|479170689|ref|NC_021020.1| Faecalibacterium prausnitzii SL3/3 draft genome	gi|479170689|ref|NC_021020.1| Faecalibacterium prausnitzii SL3/3 draft genome
contig-100_1520	1000	N	0	0	NA	0	0	0	NA	NA	NA	NA
contig-100_1521	1000	N	1	0	NA	0	0	0	NA	NA	NA	NA
contig-100_1523	999	N	0	0	NA	0	0	0	NA	NA	NA	NA
contig-100_1524	998	N	1	0	NA	0	0	0	NA	Bacteroides salanitronis DSM 18170, complete genome	gi|325297172|ref|NC_015164.1| Bacteroides salanitronis DSM 18170 chromosome, complete genome	gi|325297172|ref|NC_015164.1| Bacteroides salanitronis DSM 18170 chromosome, complete genome
contig-100_1525	998	N	0	0	NA	0	0	0	NA	NA	NA	NA
contig-100_1527	997	N	1	0	NA	0	0	0	NA	NA	NA	NA

contig-100_1528	997	N	0	0	NA	0	0	0	NA	Bacteroides xylanisolvens XB1A draft genome	gi|479162165|ref|NC_021017.1| Bacteroides xylanisolvens XB1A draft genome	gi|479162165|ref|NC_021017.1| Bacteroides xylanisolvens XB1A draft genome
contig-100_1529	997	N	0	0	NA	0	0	0	NA	NA	NA	NA
contig-100_1530	996	N	2	0	NA	0	0	0	NA	NA	NA	NA
contig-100_1532	996	N	0	0	NA	0	0	0	NA	Faecalibacterium prausnitzii L2/6 draft genome	gi|479208076|ref|NC_021042.1| Faecalibacterium prausnitzii L2-6, complete genome	gi|479208076|ref|NC_021042.1| Faecalibacterium prausnitzii L2-6, complete genome
contig-100_1533	995	N	1	0	NA	0	0	0	NA	Faecalibacterium prausnitzii L2/6 draft genome	gi|479208076|ref|NC_021042.1| Faecalibacterium prausnitzii L2-6, complete genome	gi|479208076|ref|NC_021042.1| Faecalibacterium prausnitzii L2-6, complete genome
contig-100_1534	994	N	2	0	NA	0	0	0	NA	NA	NA	NA
contig-100_1535	993	N	2	0	NA	0	0	0	NA	NA	NA	NA
contig-100_1536	993	N	2	0	NA	0	0	0	NA	NA	NA	NA
contig-100_1537	992	N	0	0	NA	0	0	0	NA	Escherichia coli str. K-12 substr. MG1655, complete genome	gi|386612163|ref|NC_017641.1| Escherichia coli UMNK88 chromosome, complete genome	gi|386612163|ref|NC_017641.1| Escherichia coli UMNK88 chromosome, complete genome
contig-100_1538	992	N	0	0	NA	0	0	0	NA	NA	NA	NA
contig-100_1540	992	N	0	0	NA	0	0	0	NA	NA	NA	NA
contig-100_1541	992	N	0	0	NA	0	0	0	NA	NA	NA	NA
contig-100_1543	991	N	1	0	NA	0	0	0	NA	Faecalibacterium prausnitzii L2/6 draft genome	gi|479208076|ref|NC_021042.1| Faecalibacterium prausnitzii L2-6, complete genome	gi|479208076|ref|NC_021042.1| Faecalibacterium prausnitzii L2-6, complete genome
contig-100_1544	991	N	0	0	NA	0	0	0	NA	NA	NA	NA

contig-100_1545	991	N	0	0	NA	0	0	0	NA	Faecalibacterium prausnitzii SL3/3 draft genome	gi|479170689|ref|NC_021020.1| Faecalibacterium prausnitzii SL3/3 draft genome	gi|479170689|ref|NC_021020.1| Faecalibacterium prausnitzii SL3/3 draft genome
contig-100_1546	990	N	2	0	NA	0	0	0	NA	Bacteroides vulgatus ATCC 8482, complete genome	gi|150002608|ref|NC_009614.1| Bacteroides vulgatus ATCC 8482 chromosome, complete genome	gi|150002608|ref|NC_009614.1| Bacteroides vulgatus ATCC 8482 chromosome, complete genome
contig-100_1547	990	N	0	0	NA	0	0	0	NA	NA	NA	NA
contig-100_1548	990	N	1	0	NA	0	0	0	NA	NA	NA	NA
contig-100_1550	987	N	2	0	NA	0	0	0	NA	NA	NA	NA
contig-100_1551	986	N	1	0	NA	0	0	0	NA	Odoribacter splanchnicus DSM 20712, complete genome	gi|325278757|ref|NC_015160.1| Odoribacter splanchnicus DSM 20712 chromosome, complete genome	gi|325278757|ref|NC_015160.1| Odoribacter splanchnicus DSM 20712 chromosome, complete genome
contig-100_1553	986	N	0	0	NA	0	0	0	NA	Uncultured organism clone VC1A048TR genomic sequence	gi|479170689|ref|NC_021020.1| Faecalibacterium prausnitzii SL3/3 draft genome	gi|479170689|ref|NC_021020.1| Faecalibacterium prausnitzii SL3/3 draft genome
contig-100_1554	986	N	2	0	NA	0	0	0	NA	NA	NA	NA
contig-100_1555	985	N	0	0	NA	0	0	0	NA	NA	NA	NA
contig-100_1557	984	N	0	0	NA	0	0	0	NA	Uncultured organism clone VC1CC69TR genomic sequence	NA	Uncultured organism clone VC1CC69TR genomic sequence

contig-100_1558	983	N	0	0	NA	0	0	0	NA	Uncultured bacterium xylooligosaccharide degrading DNA fragment, clone MetaPbio 5	NA	Uncultured bacterium xylooligosaccharide degrading DNA fragment, clone MetaPbio 5
contig-100_1559	983	N	1	0	NA	0	0	0	NA	Uncultured organism clone VC1D646TR genomic sequence	NA	Uncultured organism clone VC1D646TR genomic sequence
contig-100_156	4028	N	4	1	Herpes	0	0	2	NA	Unidentified phage clone 1013_scaffold47 genomic sequence	NA	Unidentified phage clone 1013_scaffold47 genomic sequence
contig-100_1561	983	N	0	0	NA	0	0	0	NA	NA	NA	NA
contig-100_1562	982	N	0	0	NA	0	0	0	NA	NA	NA	NA
contig-100_1563	982	N	1	0	NA	0	0	0	NA	Bacteroides fragilis YCH46 DNA, complete genome	gi|53711291|ref|NC_006347.1| Bacteroides fragilis YCH46 DNA, complete genome	gi|53711291|ref|NC_006347.1| Bacteroides fragilis YCH46 DNA, complete genome
contig-100_1564	982	N	2	0	NA	0	0	0	NA	Coprococcus sp. ART55/1 draft genome	gi|479166807|ref|NC_021018.1| Coprococcus sp. ART55/1 draft genome	gi|479166807|ref|NC_021018.1| Coprococcus sp. ART55/1 draft genome
contig-100_1565	981	N	1	0	NA	0	0	0	NA	Faecalibacterium prausnitzii L2/6 draft genome	gi|479208076|ref|NC_021042.1| Faecalibacterium prausnitzii L2-6, complete genome	gi|479208076|ref|NC_021042.1| Faecalibacterium prausnitzii L2-6, complete genome

contig-100_1566	979	N	0	0	NA	0	0	0	NA	Veillonella parvula DSM 2008, complete genome	gi|269797069|ref|NC_013520.1| Veillonella parvula DSM 2008 chromosome, complete genome	gi|269797069|ref|NC_013520.1| Veillonella parvula DSM 2008 chromosome, complete genome
contig-100_1568	978	N	1	0	NA	0	0	0	NA	NA	NA	NA
contig-100_1569	978	N	0	0	NA	0	0	0	NA	NA	NA	NA
contig-100_157	4011	N	2	0	NA	0	0	0	NA	NA	NA	NA
contig-100_1570	978	N	0	0	NA	0	0	0	NA	NA	NA	NA
contig-100_1571	977	N	0	0	NA	0	0	0	NA	NA	NA	NA
contig-100_1572	976	N	2	0	NA	0	0	0	NA	Escherichia coli str. K-12 substr. MG1655, complete genome	gi|386703215|ref|NC_017663.1| Escherichia coli P12b chromosome, complete genome	gi|386703215|ref|NC_017663.1| Escherichia coli P12b chromosome, complete genome
contig-100_1573	976	N	0	0	NA	0	0	0	NA	NA	NA	NA
contig-100_1574	975	N	1	0	NA	0	0	0	NA	NA	NA	NA
contig-100_1576	975	N	1	0	NA	0	0	0	NA	Lactobacillus helveticus CNRZ32, complete genome	gi|525706521|ref|NC_021744.1| Lactobacillus helveticus CNRZ32, complete genome	gi|525706521|ref|NC_021744.1| Lactobacillus helveticus CNRZ32, complete genome
contig-100_1577	975	N	0	0	NA	0	0	0	NA	NA	NA	NA
contig-100_1578	975	N	1	0	NA	0	0	0	NA	NA	NA	NA
contig-100_1579	975	N	1	0	NA	0	0	0	NA	NA	NA	NA
contig-100_1580	974	N	0	0	NA	0	0	0	NA	NA	gi|550916528|ref|NC_022571.1| Clostridium saccharobutylicum DSM 13864, complete genome	gi|550916528|ref|NC_022571.1| Clostridium saccharobutylicum DSM 13864, complete genome

contig-100_1581	974	N	1	0	NA	0	0	0	NA	Faecalibacterium prausnitzii L2/6 draft genome	gi|479208076|ref|NC_021042.1| Faecalibacterium prausnitzii L2-6, complete genome	gi|479208076|ref|NC_021042.1| Faecalibacterium prausnitzii L2-6, complete genome
contig-100_1582	974	N	0	0	NA	0	0	0	NA	NA	NA	NA
contig-100_1583	973	N	0	0	NA	0	0	0	NA	Veillonella parvula DSM 2008, complete genome	gi|269797069|ref|NC_013520.1| Veillonella parvula DSM 2008 chromosome, complete genome	gi|269797069|ref|NC_013520.1| Veillonella parvula DSM 2008 chromosome, complete genome
contig-100_1584	973	N	0	0	NA	0	0	0	NA	NA	NA	NA
contig-100_1585	973	N	2	0	NA	0	0	0	NA	Bacteroides salanitronis DSM 18170, complete genome	gi|325297172|ref|NC_015164.1| Bacteroides salanitronis DSM 18170 chromosome, complete genome	gi|325297172|ref|NC_015164.1| Bacteroides salanitronis DSM 18170 chromosome, complete genome
contig-100_1586	973	N	1	0	NA	0	0	0	NA	NA	NA	NA
contig-100_1587	973	N	0	0	NA	0	0	0	NA	NA	NA	NA
contig-100_1589	972	N	0	0	NA	0	0	0	NA	NA	NA	NA
contig-100_159	3958	N	3	1	Podo	0	0	1	NA	NA	NA	NA
contig-100_1590	972	N	0	0	NA	0	0	0	NA	NA	NA	NA
contig-100_1591	972	N	1	0	NA	0	0	0	NA	NA	NA	NA
contig-100_1592	971	N	1	0	NA	0	0	0	NA	Clostridiales sp. SM4/1 draft genome	gi|479181986|ref|NC_021024.1| Butyrate-producing bacterium SM4/1, complete genome	gi|479181986|ref|NC_021024.1| Butyrate-producing bacterium SM4/1, complete genome
contig-100_1593	971	N	1	0	NA	0	0	0	NA	Streptococcus phage 858, complete genome	NA	Streptococcus phage 858, complete genome
contig-100_1594	970	N	1	0	NA	0	0	0	NA	NA	NA	NA
contig-100_1595	970	N	0	0	NA	0	0	0	NA	NA	NA	NA
contig-100_1596	970	N	0	0	NA	0	0	0	NA	NA	NA	NA

contig-100_1597	969	N	0	0	NA	0	0	0	NA	Haemophilus influenzae 10810 genome	gi|378696079|ref|NC_016809.1| Haemophilus influenzae 10810, complete genome	gi|378696079|ref|NC_016809.1| Haemophilus influenzae 10810, complete genome
contig-100_1598	969	N	0	0	NA	0	0	0	NA	NA	NA	NA
contig-100_16	18354	N	14	1	NA	1	0	0	NA	Uncultured bacterium clone HA0AAA15ZD01FM1 genomic sequence	gi|150002608|ref|NC_009614.1| Bacteroides vulgatus ATCC 8482 chromosome, complete genome	gi|150002608|ref|NC_009614.1| Bacteroides vulgatus ATCC 8482 chromosome, complete genome
contig-100_1600	969	N	0	0	NA	0	0	0	NA	NA	NA	NA
contig-100_1601	969	N	0	0	NA	0	0	0	NA	NA	NA	NA
contig-100_1602	968	N	1	0	NA	0	0	0	NA	NA	NA	NA
contig-100_1604	968	N	2	0	NA	0	0	0	NA	NA	NA	NA
contig-100_1605	968	N	2	0	NA	0	0	0	NA	NA	NA	NA
contig-100_1606	968	N	0	0	NA	0	0	0	NA	Odoribacter splanchnicus DSM 20712, complete genome	gi|325278757|ref|NC_015160.1| Odoribacter splanchnicus DSM 20712 chromosome, complete genome	gi|325278757|ref|NC_015160.1| Odoribacter splanchnicus DSM 20712 chromosome, complete genome
contig-100_1608	966	N	1	0	NA	0	0	0	NA	NA	NA	NA
contig-100_1609	966	N	2	0	NA	0	0	0	NA	Parabacteroides distasonis ATCC 8503, complete genome	gi|150006674|ref|NC_009615.1| Parabacteroides distasonis ATCC 8503 chromosome, complete genome	gi|150006674|ref|NC_009615.1| Parabacteroides distasonis ATCC 8503 chromosome, complete genome
contig-100_1611	966	N	0	0	NA	0	0	0	NA	NA	NA	NA
contig-100_1612	965	N	0	0	NA	0	0	0	NA	NA	NA	NA

contig-100_1613	965	N	1	0	NA	0	0	0	NA	Bacteroides vulgatus ATCC 8482, complete genome	gi|150002608|ref|NC_009614.1| Bacteroides vulgatus ATCC 8482 chromosome, complete genome	gi|150002608|ref|NC_009614.1| Bacteroides vulgatus ATCC 8482 chromosome, complete genome
contig-100_1614	965	N	1	0	NA	0	0	0	NA	NA	NA	NA
contig-100_1615	964	N	0	0	NA	0	0	0	NA	Uncultured bacterium clone LM0ABA36ZB05FM1 genomic sequence	NA	Uncultured bacterium clone LM0ABA36ZB05FM1 genomic sequence
contig-100_1616	964	N	0	0	NA	0	0	0	NA	NA	NA	NA
contig-100_1617	964	N	0	0	NA	0	0	0	NA	NA	NA	NA
contig-100_1618	964	N	0	0	NA	0	0	0	NA	NA	NA	NA
contig-100_1619	964	N	1	0	NA	0	0	0	NA	NA	NA	NA
contig-100_1620	963	N	2	0	NA	0	0	0	NA	NA	NA	NA
contig-100_1621	963	N	1	0	NA	0	0	0	NA	NA	NA	NA
contig-100_1622	962	N	0	0	NA	0	0	0	NA	NA	NA	NA
contig-100_1624	961	N	0	0	NA	0	0	0	NA	NA	NA	NA
contig-100_1625	961	N	0	0	NA	0	0	0	NA	Bacteroides vulgatus ATCC 8482, complete genome	gi|150002608|ref|NC_009614.1| Bacteroides vulgatus ATCC 8482 chromosome, complete genome	gi|150002608|ref|NC_009614.1| Bacteroides vulgatus ATCC 8482 chromosome, complete genome
contig-100_1626	960	N	0	0	NA	0	0	0	NA	NA	NA	NA
contig-100_1627	960	N	0	0	NA	0	0	0	NA	NA	NA	NA
contig-100_1628	959	N	0	0	NA	0	0	0	NA	Bacteroides vulgatus ATCC 8482, complete genome	gi|150002608|ref|NC_009614.1| Bacteroides vulgatus ATCC 8482 chromosome, complete genome	gi|150002608|ref|NC_009614.1| Bacteroides vulgatus ATCC 8482 chromosome, complete genome
contig-100_1629	958	N	0	0	NA	0	0	0	NA	NA	NA	NA

contig-100_163	3851	N	1	0	NA	0	0	0	NA	Clostridiales sp. SSC/2 draft genome	gi|479158859|ref|NC_021016.1| Butyrate-producing bacterium SSC/2, complete genome	gi|479158859|ref|NC_021016.1| Butyrate-producing bacterium SSC/2, complete genome
contig-100_1630	958	N	0	0	NA	0	0	0	NA	NA	NA	NA
contig-100_1631	958	N	1	0	NA	0	0	0	NA	NA	NA	NA
contig-100_1632	958	N	0	0	NA	0	0	0	NA	NA	NA	NA
contig-100_1633	958	N	1	0	NA	0	0	0	NA	NA	NA	NA
contig-100_1634	958	N	0	0	NA	0	0	0	NA	NA	NA	NA
contig-100_1635	957	N	1	0	NA	0	0	0	NA	NA	NA	NA
contig-100_1636	956	N	1	0	NA	0	0	0	NA	Clostridium difficile 630 complete genome	gi|126697566|ref|NC_009089.1| Peptoclostridium difficile 630, complete genome	gi|126697566|ref|NC_009089.1| Peptoclostridium difficile 630, complete genome
contig-100_1637	956	N	0	0	NA	0	0	0	NA	NA	NA	NA
contig-100_1640	955	N	0	0	NA	0	0	0	NA	NA	NA	NA
contig-100_1641	954	N	1	0	NA	0	0	0	NA	Haemophilus parainfluenzae T3T1 complete genome	gi|345428590|ref|NC_015964.1| Haemophilus parainfluenzae T3T1, complete genome	gi|345428590|ref|NC_015964.1| Haemophilus parainfluenzae T3T1, complete genome
contig-100_1642	954	N	0	0	NA	0	0	0	NA	NA	NA	NA
contig-100_1643	953	N	1	0	NA	0	0	0	NA	Ruminococcus obeum A2-162 draft genome	gi|479176048|ref|NC_021022.1| Ruminococcus obeum A2-162 draft genome	gi|479176048|ref|NC_021022.1| Ruminococcus obeum A2-162 draft genome
contig-100_1644	953	N	0	0	NA	0	0	0	NA	NA	NA	NA
contig-100_1645	953	N	1	0	NA	0	0	0	NA	NA	NA	NA
contig-100_1646	953	N	0	0	NA	0	0	0	NA	NA	NA	NA
contig-100_1647	952	N	1	0	NA	0	0	0	NA	Uncultured organism clone VC1CI43TR genomic sequence	NA	Uncultured organism clone VC1CI43TR genomic sequence

contig-100_1648	951	N	0	0	NA	0	0	0	NA	Faecalibacterium prausnitzii SL3/3 draft genome	gi|479170689|ref|NC_021020.1| Faecalibacterium prausnitzii SL3/3 draft genome	gi|479170689|ref|NC_021020.1| Faecalibacterium prausnitzii SL3/3 draft genome
contig-100_1649	951	N	0	0	NA	0	0	0	NA	NA	NA	NA
contig-100_1650	951	N	0	0	NA	0	0	0	NA	NA	NA	NA
contig-100_1651	950	N	1	0	NA	0	0	0	NA	Bacteroides salanitronis DSM 18170, complete genome	gi|325297172|ref|NC_015164.1| Bacteroides salanitronis DSM 18170 chromosome, complete genome	gi|325297172|ref|NC_015164.1| Bacteroides salanitronis DSM 18170 chromosome, complete genome
contig-100_1652	950	N	0	0	NA	0	0	0	NA	Clostridiales sp. SSC/2 draft genome	gi|479158859|ref|NC_021016.1| Butyrate-producing bacterium SSC/2, complete genome	gi|479158859|ref|NC_021016.1| Butyrate-producing bacterium SSC/2, complete genome
contig-100_1653	950	N	0	0	NA	0	0	0	NA	Uncultured organism clone VC1CN40TF genomic sequence	NA	Uncultured organism clone VC1CN40TF genomic sequence
contig-100_1655	949	N	1	0	NA	0	0	0	NA	NA	NA	NA
contig-100_1657	949	N	1	0	NA	0	0	0	NA	NA	NA	NA
contig-100_1658	948	N	1	0	NA	0	0	0	NA	Faecalibacterium prausnitzii L2/6 draft genome	gi|479208076|ref|NC_021042.1| Faecalibacterium prausnitzii L2-6, complete genome	gi|479208076|ref|NC_021042.1| Faecalibacterium prausnitzii L2-6, complete genome
contig-100_1661	948	N	0	0	NA	0	0	0	NA	NA	gi|556589855|ref|NC_022737.1| Staphylococcus pasteuri SP1, complete genome	gi|556589855|ref|NC_022737.1| Staphylococcus pasteuri SP1, complete genome
contig-100_1663	946	N	0	0	NA	0	0	0	NA	NA	NA	NA

contig-100_1664	946	N	2	0	NA	0	0	0	NA	NA	NA	NA
contig-100_1666	945	N	1	0	NA	0	0	0	NA	NA	NA	NA
contig-100_1667	945	N	0	0	NA	0	0	0	NA	NA	NA	NA
contig-100_1668	945	N	1	0	NA	0	0	0	NA	Faecalibacterium prausnitzii SL3/3 draft genome	gi|479170689|ref|NC_021020.1| Faecalibacterium prausnitzii SL3/3 draft genome	gi|479170689|ref|NC_021020.1| Faecalibacterium prausnitzii SL3/3 draft genome
contig-100_167	3833	N	2	2	Podo	0	1	2	NA	Odoribacter splanchnicus DSM 20712, complete genome	gi|325278757|ref|NC_015160.1| Odoribacter splanchnicus DSM 20712 chromosome, complete genome	gi|325278757|ref|NC_015160.1| Odoribacter splanchnicus DSM 20712 chromosome, complete genome
contig-100_1670	945	N	1	0	NA	0	0	0	NA	Bacteroides thetaiotaomicron VPI-5482, complete genome	gi|29345410|ref|NC_004663.1| Bacteroides thetaiotaomicron VPI-5482 chromosome, complete genome	gi|29345410|ref|NC_004663.1| Bacteroides thetaiotaomicron VPI-5482 chromosome, complete genome
contig-100_1671	944	N	1	0	NA	0	0	0	NA	Eubacterium eligens ATCC 27750, complete genome	gi|238915976|ref|NC_012778.1| Eubacterium eligens ATCC 27750 chromosome, complete genome	gi|238915976|ref|NC_012778.1| Eubacterium eligens ATCC 27750 chromosome, complete genome
contig-100_1672	944	N	0	0	NA	0	0	0	NA	Ruminococcus obeum A2-162 draft genome	gi|479176048|ref|NC_021022.1| Ruminococcus obeum A2-162 draft genome	gi|479176048|ref|NC_021022.1| Ruminococcus obeum A2-162 draft genome
contig-100_1673	944	N	1	0	NA	0	0	0	NA	NA	NA	NA
contig-100_1674	943	N	0	0	NA	0	0	0	NA	NA	NA	NA
contig-100_1675	943	N	0	0	NA	0	0	0	NA	NA	NA	NA
contig-100_1677	943	N	0	0	NA	0	0	0	NA	NA	NA	NA
contig-100_1678	943	N	0	0	NA	0	0	0	NA	NA	NA	NA

contig-100_1679	942	N	2	0	NA	0	0	0	NA	Unidentified phage clone 2019_scaffold132 genomic sequence	NA	Unidentified phage clone 2019_scaffold 132 genomic sequence
contig-100_1680	942	N	1	0	NA	0	0	0	NA	Alkalilimnicola ehrlichii MLHE-1, complete genome	gi|114319166|ref|NC_008340.1| Alkalilimnicola ehrlichii MLHE-1 chromosome, complete genome	gi|114319166|ref|NC_008340.1| Alkalilimnicola ehrlichii MLHE-1 chromosome, complete genome
contig-100_1681	942	N	0	0	NA	0	0	0	NA	NA	NA	NA
contig-100_1684	941	N	0	0	NA	0	0	0	NA	Uncultured bacterium clone HA0AAA18ZD03RM1 genomic sequence	NA	Uncultured bacterium clone HA0AAA18ZD03RM1 genomic sequence
contig-100_1686	941	N	0	0	NA	0	0	0	NA	Faecalibacterium prausnitzii SL3/3 draft genome	gi|479170689|ref|NC_021020.1| Faecalibacterium prausnitzii SL3/3 draft genome	gi|479170689|ref|NC_021020.1| Faecalibacterium prausnitzii SL3/3 draft genome
contig-100_1687	940	N	0	0	NA	0	0	0	NA	NA	NA	NA
contig-100_1688	940	N	0	0	NA	0	0	0	NA	NA	NA	NA
contig-100_1689	940	N	1	0	NA	0	0	0	NA	NA	NA	NA
contig-100_169	3817	N	3	1	Sipho	0	0	1	NA	NA	NA	NA
contig-100_1690	940	N	1	0	NA	0	0	0	NA	Uncultured organism clone VC1AM22TF genomic sequence	NA	Uncultured organism clone VC1AM22TF genomic sequence
contig-100_1692	939	N	1	0	NA	0	0	0	NA	NA	NA	NA
contig-100_1693	939	N	0	0	NA	0	0	0	NA	NA	NA	NA
contig-100_1694	939	N	0	0	NA	0	0	0	NA	Bacteroides thetaiotaomicron VPI-5482, complete genome	gi|29345410|ref|NC_004663.1| Bacteroides thetaiotaomicron VPI-5482 chromosome, complete genome	gi|29345410|ref|NC_004663.1| Bacteroides thetaiotaomicron VPI-5482 chromosome, complete genome

contig-100_1696	938	N	0	0	NA	0	0	0	NA	NA	NA	NA
contig-100_1697	938	N	1	0	NA	0	0	0	NA	Faecalibacterium prausnitzii SL3/3 draft genome	gi|479170689|ref|NC_021020.1| Faecalibacterium prausnitzii SL3/3 draft genome	gi|479170689|ref|NC_021020.1| Faecalibacterium prausnitzii SL3/3 draft genome
contig-100_1698	938	N	1	0	NA	0	0	0	NA	Bacteroides thetaiotaomicron VPI-5482, complete genome	gi|29345410|ref|NC_004663.1| Bacteroides thetaiotaomicron VPI-5482 chromosome, complete genome	gi|29345410|ref|NC_004663.1| Bacteroides thetaiotaomicron VPI-5482 chromosome, complete genome
contig-100_1699	938	N	1	0	NA	0	0	0	NA	Bacteroides vulgatus ATCC 8482, complete genome	gi|150002608|ref|NC_009614.1| Bacteroides vulgatus ATCC 8482 chromosome, complete genome	gi|150002608|ref|NC_009614.1| Bacteroides vulgatus ATCC 8482 chromosome, complete genome
contig-100_170	3808	N	1	0	NA	0	0	0	NA	NA	NA	NA
contig-100_1700	938	N	1	0	NA	0	0	0	NA	NA	NA	NA
contig-100_1701	937	N	1	0	NA	0	0	0	NA	NA	NA	NA
contig-100_1702	937	N	0	0	NA	0	0	0	NA	Haemophilus parainfluenzae T3T1 complete genome	gi|345428590|ref|NC_015964.1| Haemophilus parainfluenzae T3T1, complete genome	gi|345428590|ref|NC_015964.1| Haemophilus parainfluenzae T3T1, complete genome
contig-100_1703	937	N	0	0	NA	0	0	0	NA	NA	NA	NA
contig-100_1704	937	N	2	0	NA	0	0	0	NA	NA	NA	NA
contig-100_1705	937	N	2	0	NA	0	0	0	NA	NA	NA	NA
contig-100_1706	937	N	0	0	NA	0	0	0	NA	Escherichia coli str. K-12 substr. MG1655, complete genome	gi|386612163|ref|NC_017641.1| Escherichia coli UMNK88 chromosome, complete genome	gi|386612163|ref|NC_017641.1| Escherichia coli UMNK88 chromosome, complete genome

contig-100_1707	936	N	0	0	NA	0	0	0	NA	Bacteroides vulgatus ATCC 8482, complete genome	gi|150002608|ref|NC_009614.1| Bacteroides vulgatus ATCC 8482 chromosome, complete genome	gi|150002608|ref|NC_009614.1| Bacteroides vulgatus ATCC 8482 chromosome, complete genome
contig-100_1708	935	N	0	0	NA	0	0	0	NA	Haemophilus parainfluenzae T3T1 complete genome	gi|345428590|ref|NC_015964.1| Haemophilus parainfluenzae T3T1, complete genome	gi|345428590|ref|NC_015964.1| Haemophilus parainfluenzae T3T1, complete genome
contig-100_1709	935	N	1	0	NA	0	0	0	NA	NA	NA	NA
contig-100_171	3770	N	5	2	Sipho	2	0	2	NA	Coprococcus catus GD/7 draft genome	gi|479136967|ref|NC_021009.1| Coprococcus catus GD/7 draft genome	gi|479136967|ref|NC_021009.1| Coprococcus catus GD/7 draft genome
contig-100_1710	935	N	0	0	NA	0	0	0	NA	Bacteroides salanitronis DSM 18170, complete genome	gi|325297172|ref|NC_015164.1| Bacteroides salanitronis DSM 18170 chromosome, complete genome	gi|325297172|ref|NC_015164.1| Bacteroides salanitronis DSM 18170 chromosome, complete genome
contig-100_1711	934	N	1	0	NA	0	0	0	NA	Dendroctonus ponderosae Seq01002067, whole genome shotgun sequence	NA	Dendroctonus ponderosae Seq01002067, whole genome shotgun sequence
contig-100_1713	934	N	1	0	NA	0	0	0	NA	Unidentified phage clone 2204_scaffold812 genomic sequence	NA	Unidentified phage clone 2204_scaffold812 genomic sequence
contig-100_1714	933	N	0	0	NA	0	0	0	NA	NA	NA	NA
contig-100_1715	932	N	1	0	NA	0	0	0	NA	NA	NA	NA

contig-100_1716	932	N	0	0	NA	0	0	0	NA	Bacteroides vulgatus ATCC 8482, complete genome	gi|150002608|ref|NC_009614.1| Bacteroides vulgatus ATCC 8482 chromosome, complete genome	gi|150002608|ref|NC_009614.1| Bacteroides vulgatus ATCC 8482 chromosome, complete genome
contig-100_1717	931	N	0	0	NA	0	0	0	NA	NA	NA	NA
contig-100_1718	931	N	0	0	NA	0	0	0	NA	NA	NA	NA
contig-100_1719	931	N	0	0	NA	0	0	0	NA	NA	NA	NA
contig-100_172	3744	N	2	1	Sipho	0	0	1	NA	NA	NA	NA
contig-100_1720	931	N	0	0	NA	0	0	0	NA	Bacteroides thetaiotaomicron VPI-5482, complete genome	gi|29345410|ref|NC_004663.1| Bacteroides thetaiotaomicron VPI-5482 chromosome, complete genome	gi|29345410|ref|NC_004663.1| Bacteroides thetaiotaomicron VPI-5482 chromosome, complete genome
contig-100_1721	930	N	0	0	NA	0	0	0	NA	Bacteroides helcogenes P 36-108, complete genome	gi|319899888|ref|NC_014933.1| Bacteroides helcogenes P 36-108 chromosome, complete genome	gi|319899888|ref|NC_014933.1| Bacteroides helcogenes P 36-108 chromosome, complete genome
contig-100_1722	929	N	0	0	NA	0	0	0	NA	Clostridiales sp. SSC/2 draft genome	gi|479158859|ref|NC_021016.1| Butyrate-producing bacterium SSC/2, complete genome	gi|479158859|ref|NC_021016.1| Butyrate-producing bacterium SSC/2, complete genome
contig-100_1723	929	N	0	0	NA	0	0	0	NA	NA	NA	NA
contig-100_1724	929	N	0	0	NA	0	0	0	NA	NA	NA	NA
contig-100_1725	929	N	1	0	NA	0	0	0	NA	NA	NA	NA

contig-100_1726	929	N	0	0	NA	0	0	0	NA	Escherichia coli str. K-12 substr. MG1655, complete genome	gi|388476123|ref|NC_007779.1| Escherichia coli str. K-12 substr. W3110, complete genome	gi|388476123|ref|NC_007779.1| Escherichia coli str. K-12 substr. W3110, complete genome
contig-100_1728	929	N	1	0	NA	0	0	0	NA	NA	NA	NA
contig-100_1729	928	N	0	0	NA	0	0	0	NA	NA	NA	NA
contig-100_173	3733	N	2	0	NA	0	0	1	NA	NA	NA	NA
contig-100_1730	927	N	1	0	NA	0	0	0	NA	NA	NA	NA
contig-100_1731	927	N	0	0	NA	0	0	0	NA	NA	NA	NA
contig-100_1732	927	N	0	0	NA	0	0	0	NA	Bacteroides thetaiotaomicron VPI-5482, complete genome	gi|29345410|ref|NC_004663.1| Bacteroides thetaiotaomicron VPI-5482 chromosome, complete genome	gi|29345410|ref|NC_004663.1| Bacteroides thetaiotaomicron VPI-5482 chromosome, complete genome
contig-100_1733	926	N	0	0	NA	0	0	0	NA	NA	NA	NA
contig-100_1735	926	N	0	0	NA	0	0	0	NA	NA	NA	NA
contig-100_1736	926	N	0	0	NA	0	0	0	NA	Bacteroides vulgatus ATCC 8482, complete genome	gi|150002608|ref|NC_009614.1| Bacteroides vulgatus ATCC 8482 chromosome, complete genome	gi|150002608|ref|NC_009614.1| Bacteroides vulgatus ATCC 8482 chromosome, complete genome
contig-100_1737	925	N	0	0	NA	0	0	0	NA	NA	NA	NA
contig-100_1738	925	N	1	0	NA	0	0	0	NA	Veillonella parvula DSM 2008, complete genome	gi|269797069|ref|NC_013520.1| Veillonella parvula DSM 2008 chromosome, complete genome	gi|269797069|ref|NC_013520.1| Veillonella parvula DSM 2008 chromosome, complete genome

contig-100_1739	924	N	0	0	NA	0	0	0	NA	Haemophilus influenzae F3047 complete genome	gi|319774951|ref|NC_014922.1| Haemophilus influenzae F3047 chromosome, complete genome	gi|319774951|ref|NC_014922.1| Haemophilus influenzae F3047 chromosome, complete genome
contig-100_1740	924	N	0	0	NA	0	0	0	NA	NA	NA	NA
contig-100_1741	923	N	1	0	NA	0	0	0	NA	NA	NA	NA
contig-100_1742	923	N	0	0	NA	0	0	0	NA	NA	NA	NA
contig-100_1743	922	N	0	0	NA	0	0	0	NA	Parabacteroides distasonis ATCC 8503, complete genome	gi|150006674|ref|NC_009615.1| Parabacteroides distasonis ATCC 8503 chromosome, complete genome	gi|150006674|ref|NC_009615.1| Parabacteroides distasonis ATCC 8503 chromosome, complete genome
contig-100_1744	922	N	1	0	NA	0	0	0	NA	Uncultured bacterium clone LM0ACA11ZC05FM1 genomic sequence	NA	Uncultured bacterium clone LM0ACA11ZC05FM1 genomic sequence
contig-100_1745	922	N	1	0	NA	0	0	0	NA	Unidentified phage clone 2011_scaffold3 genomic sequence	NA	Unidentified phage clone 2011_scaffold3 genomic sequence
contig-100_1746	921	N	0	0	NA	0	0	0	NA	NA	NA	NA
contig-100_1747	921	N	0	0	NA	0	0	0	NA	NA	NA	NA
contig-100_1748	921	N	0	0	NA	0	0	0	NA	NA	NA	NA
contig-100_1749	921	N	0	0	NA	0	0	0	NA	NA	NA	NA
contig-100_175	3685	N	3	2	Podo	0	0	2	NA	NA	NA	NA
contig-100_1751	920	N	1	0	NA	0	0	0	NA	Bacteroides vulgatus ATCC 8482, complete genome	gi|150002608|ref|NC_009614.1| Bacteroides vulgatus ATCC 8482 chromosome, complete genome	gi|150002608|ref|NC_009614.1| Bacteroides vulgatus ATCC 8482 chromosome, complete genome
contig-100_1752	920	N	1	0	NA	0	0	0	NA	NA	NA	NA
contig-100_1753	919	N	1	0	NA	0	0	0	NA	NA	NA	NA

contig-100_1754	919	N	1	0	NA	0	0	0	NA	NA	NA	NA
contig-100_1755	919	N	1	0	NA	0	0	0	NA	NA	NA	NA
contig-100_1756	918	N	0	0	NA	0	0	0	NA	Uncultured organism clone 1041059765155 genomic sequence	NA	Uncultured organism clone 1041059765155 genomic sequence
contig-100_1757	918	N	0	0	NA	0	0	0	NA	Bacteroides vulgatus ATCC 8482, complete genome	gi|150002608|ref|NC_009614.1| Bacteroides vulgatus ATCC 8482 chromosome, complete genome	gi|150002608|ref|NC_009614.1| Bacteroides vulgatus ATCC 8482 chromosome, complete genome
contig-100_1758	918	N	1	0	NA	0	0	0	NA	NA	NA	NA
contig-100_1759	917	N	0	0	NA	0	0	0	NA	Bacteroides vulgatus ATCC 8482, complete genome	gi|150002608|ref|NC_009614.1| Bacteroides vulgatus ATCC 8482 chromosome, complete genome	gi|150002608|ref|NC_009614.1| Bacteroides vulgatus ATCC 8482 chromosome, complete genome
contig-100_176	3663	N	2	1	Sipho	1	0	1	NA	NA	NA	NA
contig-100_1760	917	N	0	0	NA	0	0	0	NA	NA	NA	NA
contig-100_1761	917	N	0	0	NA	0	0	0	NA	NA	NA	NA
contig-100_1762	917	N	0	0	NA	0	0	0	NA	Bacteroides vulgatus ATCC 8482, complete genome	gi|150002608|ref|NC_009614.1| Bacteroides vulgatus ATCC 8482 chromosome, complete genome	gi|150002608|ref|NC_009614.1| Bacteroides vulgatus ATCC 8482 chromosome, complete genome
contig-100_1763	916	N	0	0	NA	0	0	0	NA	Faecalibacterium prausnitzii L2/6 draft genome	gi|479208076|ref|NC_021042.1| Faecalibacterium prausnitzii L2-6, complete genome	gi|479208076|ref|NC_021042.1| Faecalibacterium prausnitzii L2-6, complete genome

contig-100_1765	916	N	0	0	NA	0	0	0	NA	Bacteroides vulgatus ATCC 8482, complete genome	gi|150002608|ref|NC_009614.1| Bacteroides vulgatus ATCC 8482 chromosome, complete genome	gi|150002608|ref|NC_009614.1| Bacteroides vulgatus ATCC 8482 chromosome, complete genome
contig-100_1766	916	N	0	0	NA	0	0	0	NA	NA	NA	NA
contig-100_1767	916	N	1	0	NA	0	0	0	NA	NA	NA	NA
contig-100_1768	916	N	0	0	NA	0	0	0	NA	Faecalibacterium prausnitzii L2/6 draft genome	gi|479208076|ref|NC_021042.1| Faecalibacterium prausnitzii L2-6, complete genome	gi|479208076|ref|NC_021042.1| Faecalibacterium prausnitzii L2-6, complete genome
contig-100_1769	916	N	0	0	NA	0	0	0	NA	Acidovorax sp. KKS102, complete genome	gi|407936729|ref|NC_018708.1| Acidovorax sp. KKS102 chromosome, complete genome	gi|407936729|ref|NC_018708.1| Acidovorax sp. KKS102 chromosome, complete genome
contig-100_177	3651	N	2	0	NA	0	0	0	NA	NA	NA	NA
contig-100_1770	915	N	0	0	NA	0	0	0	NA	Unidentified phage clone 2019_scaffold132 genomic sequence	NA	Unidentified phage clone 2019_scaffold132 genomic sequence
contig-100_1771	915	N	0	0	NA	0	0	0	NA	NA	NA	NA
contig-100_1773	914	N	0	0	NA	0	0	0	NA	Odoribacter splanchnicus DSM 20712, complete genome	gi|325278757|ref|NC_015160.1| Odoribacter splanchnicus DSM 20712 chromosome, complete genome	gi|325278757|ref|NC_015160.1| Odoribacter splanchnicus DSM 20712 chromosome, complete genome

contig-100_1775	913	N	1	0	NA	0	0	0	NA	Bacteroides thetaiotaomicron VPI-5482, complete genome	gi|29345410|ref|NC_004663.1| Bacteroides thetaiotaomicron VPI-5482 chromosome, complete genome	gi|29345410|ref|NC_004663.1| Bacteroides thetaiotaomicron VPI-5482 chromosome, complete genome
contig-100_1776	913	N	1	0	NA	0	0	0	NA	NA	NA	NA
contig-100_1777	913	N	0	0	NA	0	0	0	NA	NA	NA	NA
contig-100_1778	913	N	1	0	NA	0	0	0	NA	Haemophilus influenzae F3047 complete genome	gi|319774951|ref|NC_014922.1| Haemophilus influenzae F3047 chromosome, complete genome	gi|319774951|ref|NC_014922.1| Haemophilus influenzae F3047 chromosome, complete genome
contig-100_1779	912	N	2	0	NA	0	0	0	NA	NA	NA	NA
contig-100_1780	912	N	0	0	NA	0	0	0	NA	NA	NA	NA
contig-100_1781	912	N	1	0	NA	0	0	0	NA	Uncultured bacterium clone HA0AAA7ZB08FM1 genomic sequence	NA	Uncultured bacterium clone HA0AAA7ZB08FM1 genomic sequence
contig-100_1783	911	N	0	0	NA	0	0	0	NA	NA	NA	NA
contig-100_1784	910	N	1	0	NA	0	0	0	NA	Streptococcus phage TP-J34 complete genome	NA	Streptococcus phage TP-J34 complete genome
contig-100_1785	910	N	0	0	NA	0	0	0	NA	NA	NA	NA
contig-100_1786	910	N	0	0	NA	0	0	0	NA	NA	NA	NA
contig-100_1787	910	N	0	0	NA	0	0	0	NA	Bacteroides thetaiotaomicron VPI-5482, complete genome	gi|29345410|ref|NC_004663.1| Bacteroides thetaiotaomicron VPI-5482 chromosome, complete genome	gi|29345410|ref|NC_004663.1| Bacteroides thetaiotaomicron VPI-5482 chromosome, complete genome
contig-100_1788	910	N	1	0	NA	0	0	0	NA	NA	NA	NA
contig-100_179	3638	N	3	1	Pox	0	1	1	NA	NA	NA	NA

contig-100_1790	908	N	2	0	NA	0	1	0	NA	Uncultured organism clone VC1A991TR genomic sequence	NA	Uncultured organism clone VC1A991TR genomic sequence
contig-100_1791	908	N	1	0	NA	0	0	0	NA	Uncultured organism clone VC1D964TF genomic sequence	NA	Uncultured organism clone VC1D964TF genomic sequence
contig-100_1792	908	N	0	0	NA	0	0	0	NA	NA	NA	NA
contig-100_1793	907	N	0	0	NA	0	0	0	NA	Bacteroides helcogenes P 36-108, complete genome	gi|319899888|ref|NC_014933.1| Bacteroides helcogenes P 36-108 chromosome, complete genome	gi|319899888|ref|NC_014933.1| Bacteroides helcogenes P 36-108 chromosome, complete genome
contig-100_1794	907	N	1	0	NA	0	0	0	NA	Ruminococcus sp. SR1/5 draft genome	gi|479152295|ref|NC_021014.1| Ruminococcus sp. SR1/5 draft genome	gi|479152295|ref|NC_021014.1| Ruminococcus sp. SR1/5 draft genome
contig-100_1795	907	N	1	0	NA	0	0	0	NA	NA	NA	NA
contig-100_1797	906	N	1	0	NA	0	0	0	NA	Roseburia hominis A2-183, complete genome	gi|347530298|ref|NC_015977.1| Roseburia hominis A2-183 chromosome, complete genome	gi|347530298|ref|NC_015977.1| Roseburia hominis A2-183 chromosome, complete genome
contig-100_1798	906	N	1	0	NA	0	0	0	NA	Faecalibacterium prausnitzii SL3/3 draft genome	gi|479170689|ref|NC_021020.1| Faecalibacterium prausnitzii SL3/3 draft genome	gi|479170689|ref|NC_021020.1| Faecalibacterium prausnitzii SL3/3 draft genome

contig-100_1799	906	N	2	0	NA	0	0	0	NA	Pseudomonas fluorescens SBW25 complete genome	gi|229587578|ref|NC_012660.1| Pseudomonas fluorescens SBW25 chromosome, complete genome	gi|229587578|ref|NC_012660.1| Pseudomonas fluorescens SBW25 chromosome, complete genome
contig-100_18	16577	N	15	7	Sipho	0	2	8	NA	Unidentified phage clone 1013_scaffold47 genomic sequence	gi|479140210|ref|NC_021010.1| Eubacterium rectale DSM 17629 draft genome	gi|479140210|ref|NC_021010.1| Eubacterium rectale DSM 17629 draft genome
contig-100_1800	906	N	0	0	NA	0	0	0	NA	NA	NA	NA
contig-100_1801	906	N	0	0	NA	0	0	0	NA	Clostridiales sp. SSC/2 draft genome	gi|479158859|ref|NC_021016.1| Butyrate-producing bacterium SSC/2, complete genome	gi|479158859|ref|NC_021016.1| Butyrate-producing bacterium SSC/2, complete genome
contig-100_1802	905	N	1	0	NA	0	0	0	NA	NA	NA	NA
contig-100_1804	905	N	0	0	NA	0	0	0	NA	Bacteroides salanitronis DSM 18170, complete genome	gi|325297172|ref|NC_015164.1| Bacteroides salanitronis DSM 18170 chromosome, complete genome	gi|325297172|ref|NC_015164.1| Bacteroides salanitronis DSM 18170 chromosome, complete genome
contig-100_1805	905	N	0	0	NA	0	0	0	NA	Escherichia coli str. K-12 substr. MG1655, complete genome	gi|386612163|ref|NC_017641.1| Escherichia coli UMNK88 chromosome, complete genome	gi|386612163|ref|NC_017641.1| Escherichia coli UMNK88 chromosome, complete genome
contig-100_1806	905	N	0	0	NA	0	0	0	NA	NA	NA	NA
contig-100_1807	904	N	1	1	Sipho	0	0	1	NA	NA	NA	NA

contig-100_1808	904	N	0	0	NA	0	0	0	NA	Bacteroides vulgatus ATCC 8482, complete genome	gi|150002608|ref|NC_009614.1| Bacteroides vulgatus ATCC 8482 chromosome, complete genome	gi|150002608|ref|NC_009614.1| Bacteroides vulgatus ATCC 8482 chromosome, complete genome
contig-100_1809	904	N	0	0	NA	0	0	0	NA	Uncultured organism clone VC1D812TF genomic sequence	NA	Uncultured organism clone VC1D812TF genomic sequence
contig-100_181	3611	N	2	1	Myo	0	0	1	NA	NA	NA	NA
contig-100_1810	903	N	0	0	NA	0	0	0	NA	NA	NA	NA
contig-100_1811	903	N	2	0	NA	0	0	0	NA	Bacteroides vulgatus ATCC 8482, complete genome	gi|150002608|ref|NC_009614.1| Bacteroides vulgatus ATCC 8482 chromosome, complete genome	gi|150002608|ref|NC_009614.1| Bacteroides vulgatus ATCC 8482 chromosome, complete genome
contig-100_1812	903	N	3	0	NA	0	0	0	NA	NA	NA	NA
contig-100_1813	902	N	0	0	NA	0	0	0	NA	Bacteroides helcogenes P 36-108, complete genome	gi|319899888|ref|NC_014933.1| Bacteroides helcogenes P 36-108 chromosome, complete genome	gi|319899888|ref|NC_014933.1| Bacteroides helcogenes P 36-108 chromosome, complete genome
contig-100_1814	902	N	2	0	NA	0	0	0	NA	NA	NA	NA
contig-100_1815	902	N	1	0	NA	0	0	0	NA	Faecalibacterium prausnitzii SL3/3 draft genome	gi|479170689|ref|NC_021020.1| Faecalibacterium prausnitzii SL3/3 draft genome	gi|479170689|ref|NC_021020.1| Faecalibacterium prausnitzii SL3/3 draft genome
contig-100_1816	902	N	0	0	NA	0	0	0	NA	NA	NA	NA
contig-100_1817	901	N	0	0	NA	0	0	0	NA	NA	NA	NA
contig-100_1818	901	N	0	0	NA	0	0	0	NA	NA	NA	NA
contig-100_182	3607	N	1	0	NA	0	0	0	NA	NA	NA	NA
contig-100_1821	900	N	2	0	NA	0	0	0	NA	NA	NA	NA

contig-100_1822	899	N	0	0	NA	0	0	0	NA	Uncultured bacterium clone HA0AAA2ZD02RM1 genomic sequence	NA	Uncultured bacterium clone HA0AAA2ZD02RM1 genomic sequence
contig-100_1823	899	N	2	0	NA	0	0	0	NA	NA	NA	NA
contig-100_1824	899	N	2	0	NA	0	0	0	NA	NA	NA	NA
contig-100_1825	899	N	0	0	NA	0	0	0	NA	NA	NA	NA
contig-100_1827	898	N	0	0	NA	0	0	0	NA	NA	NA	NA
contig-100_1828	898	N	0	0	NA	0	0	0	NA	NA	NA	NA
contig-100_1829	898	N	0	0	NA	0	0	0	NA	NA	NA	NA
contig-100_1830	898	N	1	0	NA	0	0	0	NA	Haemophilus parainfluenzae T3T1 complete genome	gi|345428590|ref|NC_015964.1| Haemophilus parainfluenzae T3T1, complete genome	gi|345428590|ref|NC_015964.1| Haemophilus parainfluenzae T3T1, complete genome
contig-100_1831	898	N	0	0	NA	0	0	0	NA	NA	NA	NA
contig-100_1833	897	N	0	0	NA	0	0	0	NA	Geobacillus sp. JF8, complete genome	gi|563649971|ref|NC_022080.4| Geobacillus sp. JF8, complete genome	gi|563649971|ref|NC_022080.4| Geobacillus sp. JF8, complete genome
contig-100_1835	897	N	0	0	NA	0	0	0	NA	NA	NA	NA
contig-100_1836	896	N	0	0	NA	0	0	0	NA	PREDICTED: Ceratitis capitata UDP-glucose 6-dehydrogenase-like (LOC101448879), mRNA	gi|590001402|ref|NC_013446.2| Comamonas testosteroni CNB-2, complete genome	gi|590001402|ref|NC_013446.2| Comamonas testosteroni CNB-2, complete genome
contig-100_1837	896	N	0	0	NA	0	0	0	NA	Haemophilus parainfluenzae T3T1 complete genome	gi|345428590|ref|NC_015964.1| Haemophilus parainfluenzae T3T1, complete genome	gi|345428590|ref|NC_015964.1| Haemophilus parainfluenzae T3T1, complete genome
contig-100_1838	896	N	1	0	NA	0	0	0	NA	NA	NA	NA
contig-100_1840	894	N	0	0	NA	0	0	0	NA	NA	NA	NA
contig-100_1841	893	N	0	0	NA	0	0	0	NA	NA	NA	NA

contig-100_1842	893	N	1	0	NA	0	0	0	NA	Clostridiales sp. SSC/2 draft genome	gi|479158859|ref|NC_021016.1| Butyrate-producing bacterium SSC/2, complete genome	gi|479158859|ref|NC_021016.1| Butyrate-producing bacterium SSC/2, complete genome
contig-100_1843	893	N	0	0	NA	0	0	0	NA	NA	NA	NA
contig-100_1844	893	N	2	0	NA	0	0	0	NA	NA	NA	NA
contig-100_1845	893	N	0	0	NA	0	0	0	NA	Bacteroides vulgatus ATCC 8482, complete genome	gi|150002608|ref|NC_009614.1| Bacteroides vulgatus ATCC 8482 chromosome, complete genome	gi|150002608|ref|NC_009614.1| Bacteroides vulgatus ATCC 8482 chromosome, complete genome
contig-100_1846	893	N	1	0	NA	0	0	0	NA	Streptococcus phage 2167	gi|307126151|ref|NC_014498.1| Streptococcus pneumoniae 670-6B chromosome, complete genome	gi|307126151|ref|NC_014498.1| Streptococcus pneumoniae 670-6B chromosome, complete genome
contig-100_1847	892	N	1	0	NA	0	0	0	NA	NA	NA	NA
contig-100_1848	892	N	0	0	NA	0	0	0	NA	NA	NA	NA
contig-100_1849	892	N	0	0	NA	0	0	0	NA	Faecalibacterium prausnitzii SL3/3 draft genome	gi|479170689|ref|NC_021020.1| Faecalibacterium prausnitzii SL3/3 draft genome	gi|479170689|ref|NC_021020.1| Faecalibacterium prausnitzii SL3/3 draft genome
contig-100_185	3606	N	1	0	NA	0	0	0	NA	Bacteroides vulgatus ATCC 8482, complete genome	gi|150002608|ref|NC_009614.1| Bacteroides vulgatus ATCC 8482 chromosome, complete genome	gi|150002608|ref|NC_009614.1| Bacteroides vulgatus ATCC 8482 chromosome, complete genome

contig-100_1850	891	N	1	0	NA	0	0	0	NA	Bifidobacterium catenulatum plasmid pBC1, complete sequence	gi|213690928|ref|NC_011593.1| Bifidobacterium longum subsp. infantis ATCC 15697 chromosome, complete genome	gi|213690928|ref|NC_011593.1| Bifidobacterium longum subsp. infantis ATCC 15697 chromosome, complete genome
contig-100_1851	890	N	1	0	NA	0	0	0	NA	Bacteroides xylanisolvens XB1A draft genome	gi|479162165|ref|NC_021017.1| Bacteroides xylanisolvens XB1A draft genome	gi|479162165|ref|NC_021017.1| Bacteroides xylanisolvens XB1A draft genome
contig-100_1852	890	N	0	0	NA	0	0	0	NA	NA	NA	NA
contig-100_1853	890	N	1	0	NA	0	0	0	NA	NA	NA	NA
contig-100_1854	890	N	0	0	NA	0	0	0	NA	Haemophilus influenzae F3047 complete genome	gi|319774951|ref|NC_014922.1| Haemophilus influenzae F3047 chromosome, complete genome	gi|319774951|ref|NC_014922.1| Haemophilus influenzae F3047 chromosome, complete genome
contig-100_1855	890	N	1	0	NA	0	0	0	NA	NA	NA	NA
contig-100_1856	890	N	1	0	NA	0	0	0	NA	NA	NA	NA
contig-100_1857	889	N	0	0	NA	0	0	0	NA	Lactobacillus bacteriophage phage AQ113, complete genome	NA	Lactobacillus bacteriophage phage AQ113, complete genome
contig-100_1858	889	N	0	0	NA	0	0	0	NA	Alistipes shahii WAL 8301 draft genome	gi|479185170|ref|NC_021030.1| Alistipes shahii WAL 8301 draft genome	gi|479185170|ref|NC_021030.1| Alistipes shahii WAL 8301 draft genome
contig-100_186	3604	N	3	0	NA	0	0	0	NA	Uncultured bacterium clone HA0AAA3ZG01FM1 genomic sequence	gi|325297172|ref|NC_015164.1| Bacteroides salanitronis DSM 18170 chromosome, complete genome	gi|325297172|ref|NC_015164.1| Bacteroides salanitronis DSM 18170 chromosome, complete genome

contig-100_1860	888	N	0	0	NA	0	0	0	NA	NA	NA	NA
contig-100_1861	888	N	0	0	NA	0	0	0	NA	NA	NA	NA
contig-100_1862	888	N	1	0	NA	0	0	0	NA	NA	NA	NA
contig-100_1863	887	N	0	0	NA	0	0	0	NA	Kyrpidia tusciae DSM 2912, complete genome	gi|295694686|ref|NC_014098.1| Kyrpidia tusciae DSM 2912 chromosome, complete genome	gi|295694686|ref|NC_014098.1| Kyrpidia tusciae DSM 2912 chromosome, complete genome
contig-100_1865	887	N	0	0	NA	0	0	0	NA	NA	NA	NA
contig-100_1866	887	N	0	0	NA	0	0	0	NA	NA	NA	NA
contig-100_1867	887	N	0	0	NA	0	0	0	NA	NA	NA	NA
contig-100_1868	886	N	0	0	NA	0	0	0	NA	Haemophilus parainfluenzae T3T1 complete genome	gi|345428590|ref|NC_015964.1| Haemophilus parainfluenzae T3T1, complete genome	gi|345428590|ref|NC_015964.1| Haemophilus parainfluenzae T3T1, complete genome
contig-100_1869	886	N	0	0	NA	0	0	0	NA	NA	NA	NA
contig-100_187	3600	N	3	0	NA	0	0	0	NA	NA	NA	NA
contig-100_1870	885	N	2	0	NA	0	0	0	NA	NA	NA	NA
contig-100_1871	884	N	1	0	NA	0	0	0	NA	Faecalibacterium prausnitzii L2/6 draft genome	gi|479208076|ref|NC_021042.1| Faecalibacterium prausnitzii L2-6, complete genome	gi|479208076|ref|NC_021042.1| Faecalibacterium prausnitzii L2-6, complete genome
contig-100_1872	884	N	0	0	NA	0	0	0	NA	Bacteroides vulgatus ATCC 8482, complete genome	gi|150002608|ref|NC_009614.1| Bacteroides vulgatus ATCC 8482 chromosome, complete genome	gi|150002608|ref|NC_009614.1| Bacteroides vulgatus ATCC 8482 chromosome, complete genome
contig-100_1873	883	N	0	0	NA	0	0	0	NA	NA	NA	NA
contig-100_1874	883	N	0	0	NA	0	0	0	NA	NA	NA	NA
contig-100_1875	883	N	1	0	NA	0	0	0	NA	NA	NA	NA
contig-100_1876	882	N	2	0	NA	0	0	0	NA	NA	NA	NA
contig-100_1878	881	N	0	0	NA	0	0	0	NA	NA	NA	NA
contig-100_1879	881	N	0	0	NA	0	0	0	NA	NA	NA	NA

contig-100_188	3597	N	4	2	Sipho	1	2	2	NA	Haemophilus parainfluenzae T3T1 complete genome	gi|345428590|ref|NC_015964.1| Haemophilus parainfluenzae T3T1, complete genome	gi|345428590|ref|NC_015964.1| Haemophilus parainfluenzae T3T1, complete genome
contig-100_1880	881	N	0	0	NA	0	0	0	NA	NA	NA	NA
contig-100_1881	881	N	0	0	NA	0	0	0	NA	NA	NA	NA
contig-100_1882	881	N	0	0	NA	0	0	0	NA	NA	NA	NA
contig-100_1883	880	N	2	0	NA	0	0	0	NA	Faecalibacterium prausnitzii SL3/3 draft genome	gi|479170689|ref|NC_021020.1| Faecalibacterium prausnitzii SL3/3 draft genome	gi|479170689|ref|NC_021020.1| Faecalibacterium prausnitzii SL3/3 draft genome
contig-100_1884	880	N	1	0	NA	0	0	0	NA	NA	NA	NA
contig-100_1885	880	N	0	0	NA	0	0	0	NA	NA	NA	NA
contig-100_1888	879	N	0	0	NA	0	0	0	NA	NA	NA	NA
contig-100_1889	879	N	0	0	NA	0	0	0	NA	NA	NA	NA
contig-100_189	3583	N	3	1	Podo	0	1	1	NA	NA	NA	NA
contig-100_1890	879	N	2	0	NA	0	0	0	NA	NA	NA	NA
contig-100_1891	879	N	1	0	NA	0	0	0	NA	NA	NA	NA
contig-100_1892	878	N	1	0	NA	0	0	0	NA	Streptococcus phage Abc2, complete genome	NA	Streptococcus phage Abc2, complete genome
contig-100_1893	878	N	1	0	NA	0	0	0	NA	Uncultured bacterium clone LM0ABA34ZE05RM1 genomic sequence	NA	Uncultured bacterium clone LM0ABA34ZE05RM1 genomic sequence
contig-100_1894	878	N	0	0	NA	0	0	0	NA	NA	NA	NA
contig-100_1895	877	N	1	0	NA	0	0	0	NA	Uncultured organism clone VC1CG60TR genomic sequence	gi|319899888|ref|NC_014933.1| Bacteroides helcogenes P 36-108 chromosome, complete genome	gi|319899888|ref|NC_014933.1| Bacteroides helcogenes P 36-108 chromosome, complete genome
contig-100_1896	877	N	0	0	NA	0	0	0	NA	NA	NA	NA
contig-100_1898	876	N	0	0	NA	0	0	0	NA	NA	NA	NA

contig-100_190	3565	N	2	1	Sipho	0	0	1	NA	NA	NA	NA
contig-100_1900	876	N	0	0	NA	0	0	0	NA	NA	NA	NA
contig-100_1901	876	N	1	0	NA	0	0	0	NA	Faecalibacterium prausnitzii SL3/3 draft genome	gi|479170689|ref|NC_021020.1| Faecalibacterium prausnitzii SL3/3 draft genome	gi|479170689|ref|NC_021020.1| Faecalibacterium prausnitzii SL3/3 draft genome
contig-100_1902	876	N	2	0	NA	0	0	0	NA	Bacteroides salanitronis DSM 18170, complete genome	gi|325297172|ref|NC_015164.1| Bacteroides salanitronis DSM 18170 chromosome, complete genome	gi|325297172|ref|NC_015164.1| Bacteroides salanitronis DSM 18170 chromosome, complete genome
contig-100_1903	875	N	2	0	NA	0	0	0	NA	NA	NA	NA
contig-100_1904	875	N	0	0	NA	0	0	0	NA	Faecalibacterium prausnitzii L2/6 draft genome	gi|479208076|ref|NC_021042.1| Faecalibacterium prausnitzii L2-6, complete genome	gi|479208076|ref|NC_021042.1| Faecalibacterium prausnitzii L2-6, complete genome
contig-100_1906	875	N	0	0	NA	0	0	0	NA	Bacteroides vulgatus ATCC 8482, complete genome	gi|150002608|ref|NC_009614.1| Bacteroides vulgatus ATCC 8482 chromosome, complete genome	gi|150002608|ref|NC_009614.1| Bacteroides vulgatus ATCC 8482 chromosome, complete genome
contig-100_1907	874	N	0	0	NA	0	0	0	NA	NA	NA	NA
contig-100_1908	874	N	1	0	NA	0	0	0	NA	Bacteroides vulgatus ATCC 8482, complete genome	gi|150002608|ref|NC_009614.1| Bacteroides vulgatus ATCC 8482 chromosome, complete genome	gi|150002608|ref|NC_009614.1| Bacteroides vulgatus ATCC 8482 chromosome, complete genome

contig-100_1909	874	N	0	0	NA	0	0	0	NA	Porphyromonas gingivalis TDC60 DNA, complete genome	gi|334145811|ref|NC_015571.1| Porphyromonas gingivalis TDC60, complete genome	gi|334145811|ref|NC_015571.1| Porphyromonas gingivalis TDC60, complete genome
contig-100_191	3555	Y	1	0	NA	0	0	0	NA	Klebsiella pneumoniae plasmid pJHCMW1, complete sequence	gi|384541569|ref|NC_017320.1| Shigella flexneri 2002017 plasmid pSFxv_2, complete sequence	gi|384541569|ref|NC_017320.1| Shigella flexneri 2002017 plasmid pSFxv_2, complete sequence
contig-100_1911	873	N	1	0	NA	0	0	0	NA	NA	NA	NA
contig-100_1912	873	N	1	0	NA	0	0	0	NA	Bacteroides vulgatus ATCC 8482, complete genome	gi|150002608|ref|NC_009614.1| Bacteroides vulgatus ATCC 8482 chromosome, complete genome	gi|150002608|ref|NC_009614.1| Bacteroides vulgatus ATCC 8482 chromosome, complete genome
contig-100_1913	872	N	0	0	NA	0	0	0	NA	NA	NA	NA
contig-100_1914	872	N	0	0	NA	0	0	0	NA	Clostridiales sp. SM4/1 draft genome	gi|479181986|ref|NC_021024.1| Butyrate-producing bacterium SM4/1, complete genome	gi|479181986|ref|NC_021024.1| Butyrate-producing bacterium SM4/1, complete genome
contig-100_1915	872	N	0	0	NA	0	0	0	NA	NA	NA	NA
contig-100_1916	871	N	1	0	NA	0	0	0	NA	NA	NA	NA
contig-100_1918	871	N	0	0	NA	0	0	0	NA	NA	NA	NA
contig-100_1919	871	N	0	0	NA	0	0	0	NA	Faecalibacterium prausnitzii SL3/3 draft genome	gi|479170689|ref|NC_021020.1| Faecalibacterium prausnitzii SL3/3 draft genome	gi|479170689|ref|NC_021020.1| Faecalibacterium prausnitzii SL3/3 draft genome
contig-100_1920	871	N	1	0	NA	0	0	0	NA	NA	NA	NA

contig-100_1921	871	N	0	0	NA	0	0	0	NA	Bacteroides vulgatus ATCC 8482, complete genome	gi|150002608|ref|NC_009614.1| Bacteroides vulgatus ATCC 8482 chromosome, complete genome	gi|150002608|ref|NC_009614.1| Bacteroides vulgatus ATCC 8482 chromosome, complete genome
contig-100_1923	870	N	0	0	NA	0	0	0	NA	NA	NA	NA
contig-100_1924	870	N	0	0	NA	0	0	0	NA	NA	NA	NA
contig-100_1925	870	N	2	0	NA	0	0	0	NA	Acidovorax ebreus TPSY, complete genome	gi|222109225|ref|NC_011992.1| Acidovorax ebreus TPSY chromosome, complete genome	gi|222109225|ref|NC_011992.1| Acidovorax ebreus TPSY chromosome, complete genome
contig-100_1926	870	N	1	0	NA	0	0	0	NA	NA	NA	NA
contig-100_1927	870	N	1	0	NA	0	0	0	NA	NA	NA	NA
contig-100_1928	869	N	1	0	NA	0	0	0	NA	NA	NA	NA
contig-100_1929	869	N	0	0	NA	0	0	0	NA	NA	NA	NA
contig-100_193	3463	N	4	0	NA	0	0	0	NA	Uncultured organism clone 1041059764889 genomic sequence	NA	Uncultured organism clone 1041059764889 genomic sequence
contig-100_1930	869	N	0	0	NA	0	0	0	NA	Eubacterium rectale M104/1 draft genome	gi|479213596|ref|NC_021044.1| Eubacterium rectale M104/1 draft genome	gi|479213596|ref|NC_021044.1| Eubacterium rectale M104/1 draft genome
contig-100_1932	869	N	1	0	NA	0	0	0	NA	NA	NA	NA
contig-100_1933	869	N	3	0	NA	0	0	0	NA	NA	NA	NA
contig-100_1934	867	N	0	0	NA	0	0	0	NA	NA	NA	NA
contig-100_1935	867	N	0	0	NA	0	0	0	NA	NA	NA	NA
contig-100_1936	867	N	1	0	NA	0	0	0	NA	Bacteroides salanitronis DSM 18170, complete genome	gi|325297172|ref|NC_015164.1| Bacteroides salanitronis DSM 18170 chromosome, complete genome	gi|325297172|ref|NC_015164.1| Bacteroides salanitronis DSM 18170 chromosome, complete genome

contig-100_1937	867	N	1	0	NA	0	0	0	NA	Faecalibacterium prausnitzii L2/6 draft genome	gi|479208076|ref|NC_021042.1| Faecalibacterium prausnitzii L2-6, complete genome	gi|479208076|ref|NC_021042.1| Faecalibacterium prausnitzii L2-6, complete genome
contig-100_1938	867	N	0	0	NA	0	0	0	NA	Uncultured bacterium clone HA0AAA15ZD11RM1 genomic sequence	NA	Uncultured bacterium clone HA0AAA15ZD11RM1 genomic sequence
contig-100_1939	867	N	0	0	NA	0	0	0	NA	NA	NA	NA
contig-100_194	3463	N	0	0	NA	0	0	0	NA	NA	NA	NA
contig-100_1940	867	N	0	0	NA	0	0	0	NA	NA	NA	NA
contig-100_1941	867	N	0	0	NA	0	0	0	NA	NA	NA	NA
contig-100_1943	866	N	0	0	NA	0	0	0	NA	NA	NA	NA
contig-100_1944	866	N	0	0	NA	0	0	0	NA	Bacteroides vulgatus ATCC 8482, complete genome	gi|150002608|ref|NC_009614.1| Bacteroides vulgatus ATCC 8482 chromosome, complete genome	gi|150002608|ref|NC_009614.1| Bacteroides vulgatus ATCC 8482 chromosome, complete genome
contig-100_1947	866	N	1	0	NA	0	0	0	NA	Clostridiales sp. SSC/2 draft genome	gi|479158859|ref|NC_021016.1| Butyrate-producing bacterium SSC/2, complete genome	gi|479158859|ref|NC_021016.1| Butyrate-producing bacterium SSC/2, complete genome
contig-100_1948	866	N	1	0	NA	0	0	0	NA	NA	NA	NA
contig-100_1949	866	N	2	0	NA	0	0	0	NA	NA	NA	NA
contig-100_195	3453	N	2	0	NA	0	0	0	NA	NA	NA	NA
contig-100_1950	865	N	0	0	NA	0	0	0	NA	NA	NA	NA
contig-100_1952	865	N	1	0	NA	0	0	0	NA	Uncultured organism clone VC1DF08TF genomic sequence	NA	Uncultured organism clone VC1DF08TF genomic sequence
contig-100_1953	865	N	1	0	NA	0	0	0	NA	NA	NA	NA
contig-100_1954	864	N	0	0	NA	0	0	0	NA	NA	NA	NA

contig-100_1956	864	N	0	0	NA	0	0	0	NA	Bacteroides fragilis NCTC 9343, complete genome	gi|60679597|ref|NC_003228.3| Bacteroides fragilis NCTC 9343, complete genome	gi|60679597|ref|NC_003228.3| Bacteroides fragilis NCTC 9343, complete genome
contig-100_1958	864	N	1	0	NA	0	0	0	NA	NA	NA	NA
contig-100_1959	863	N	0	0	NA	0	0	0	NA	NA	NA	NA
contig-100_196	3447	N	3	1	Phycodna	0	1	1	NA	NA	NA	NA
contig-100_1960	863	N	0	0	NA	0	0	0	NA	NA	NA	NA
contig-100_1961	862	N	1	0	NA	0	0	0	NA	Bacteroides vulgatus ATCC 8482, complete genome	gi|150002608|ref|NC_009614.1| Bacteroides vulgatus ATCC 8482 chromosome, complete genome	gi|150002608|ref|NC_009614.1| Bacteroides vulgatus ATCC 8482 chromosome, complete genome
contig-100_1964	860	N	1	0	NA	0	0	0	NA	Roseburia hominis A2-183, complete genome	gi|347530298|ref|NC_015977.1| Roseburia hominis A2-183 chromosome, complete genome	gi|347530298|ref|NC_015977.1| Roseburia hominis A2-183 chromosome, complete genome
contig-100_1965	860	N	1	0	NA	0	0	0	NA	Bacteroides vulgatus ATCC 8482, complete genome	gi|150002608|ref|NC_009614.1| Bacteroides vulgatus ATCC 8482 chromosome, complete genome	gi|150002608|ref|NC_009614.1| Bacteroides vulgatus ATCC 8482 chromosome, complete genome
contig-100_1967	860	N	1	0	NA	0	0	0	NA	Faecalibacterium prausnitzii SL3/3 draft genome	gi|479170689|ref|NC_021020.1| Faecalibacterium prausnitzii SL3/3 draft genome	gi|479170689|ref|NC_021020.1| Faecalibacterium prausnitzii SL3/3 draft genome

contig-100_1968	860	N	0	0	NA	0	0	0	NA	Neisseria gonorrhoeae strain GP08-MUS-021 plasmid pEM1, complete sequence	NA	Neisseria gonorrhoeae strain GP08-MUS-021 plasmid pEM1, complete sequence
contig-100_1969	858	N	0	0	NA	0	0	0	NA	NA	NA	NA
contig-100_197	3447	N	3	3	Sipho	0	0	3	NA	NA	NA	NA
contig-100_1970	858	N	1	0	NA	0	0	0	NA	NA	NA	NA
contig-100_1971	858	N	1	0	NA	0	0	0	NA	NA	NA	NA
contig-100_1972	858	N	1	0	NA	0	0	0	NA	Bacteroides vulgatus ATCC 8482, complete genome	gi|150002608|ref|NC_009614.1| Bacteroides vulgatus ATCC 8482 chromosome, complete genome	gi|150002608|ref|NC_009614.1| Bacteroides vulgatus ATCC 8482 chromosome, complete genome
contig-100_1973	857	N	0	0	NA	0	0	0	NA	Bacteroides thetaiotaomicron VPI-5482, complete genome	gi|29345410|ref|NC_004663.1| Bacteroides thetaiotaomicron VPI-5482 chromosome, complete genome	gi|29345410|ref|NC_004663.1| Bacteroides thetaiotaomicron VPI-5482 chromosome, complete genome
contig-100_1974	857	N	1	0	NA	0	0	0	NA	NA	NA	NA
contig-100_1975	857	N	1	0	NA	0	0	0	NA	NA	NA	NA
contig-100_1976	857	N	0	0	NA	0	0	0	NA	NA	NA	NA
contig-100_1977	856	N	1	0	NA	0	0	0	NA	Dendroctonus ponderosae Seq01005302, whole genome shotgun sequence	NA	Dendroctonus ponderosae Seq01005302, whole genome shotgun sequence
contig-100_1978	856	N	1	0	NA	0	0	0	NA	Clostridiales sp. SSC/2 draft genome	gi|479158859|ref|NC_021016.1| Butyrate-producing bacterium SSC/2, complete genome	gi|479158859|ref|NC_021016.1| Butyrate-producing bacterium SSC/2, complete genome

contig-100_1979	856	N	1	0	NA	0	0	0	NA	Acidovorax sp. KKS102, complete genome	gi|407936729|ref|NC_018708.1| Acidovorax sp. KKS102 chromosome, complete genome	gi|407936729|ref|NC_018708.1| Acidovorax sp. KKS102 chromosome, complete genome
contig-100_198	3444	N	2	1	Sipho	0	1	1	NA	NA	NA	NA
contig-100_1980	855	N	0	0	NA	0	0	0	NA	Eubacterium rectale ATCC 33656, complete genome	gi|238922432|ref|NC_012781.1| Eubacterium rectale ATCC 33656, complete genome	gi|238922432|ref|NC_012781.1| Eubacterium rectale ATCC 33656, complete genome
contig-100_1981	855	N	0	0	NA	0	0	0	NA	NA	NA	NA
contig-100_1982	854	N	1	0	NA	0	0	0	NA	Uncultured organism clone Turk_fec371 genomic sequence	NA	Uncultured organism clone Turk_fec371 genomic sequence
contig-100_1983	854	N	1	0	NA	0	0	0	NA	Uncultured bacterium contig00219 genomic sequence	NA	Uncultured bacterium contig00219 genomic sequence
contig-100_1984	854	N	0	0	NA	0	0	0	NA	Uncultured organism clone 7 genomic sequence	gi|479170689|ref|NC_021020.1| Faecalibacterium prausnitzii SL3/3 draft genome	gi|479170689|ref|NC_021020.1| Faecalibacterium prausnitzii SL3/3 draft genome
contig-100_1985	854	N	0	0	NA	0	0	0	NA	Eubacterium rectale ATCC 33656, complete genome	gi|238922432|ref|NC_012781.1| Eubacterium rectale ATCC 33656, complete genome	gi|238922432|ref|NC_012781.1| Eubacterium rectale ATCC 33656, complete genome

contig-100_1988	853	N	0	0	NA	0	0	0	NA	Bacteroides vulgatus ATCC 8482, complete genome	gi|150002608|ref|NC_009614.1| Bacteroides vulgatus ATCC 8482 chromosome, complete genome	gi|150002608|ref|NC_009614.1| Bacteroides vulgatus ATCC 8482 chromosome, complete genome
contig-100_1989	853	N	3	0	NA	0	0	0	NA	Faecalibacterium prausnitzii L2/6 draft genome	gi|479208076|ref|NC_021042.1| Faecalibacterium prausnitzii L2-6, complete genome	gi|479208076|ref|NC_021042.1| Faecalibacterium prausnitzii L2-6, complete genome
contig-100_199	3401	N	1	1	Sipho	0	0	1	NA	NA	NA	NA
contig-100_1990	853	N	0	0	NA	0	0	0	NA	Uncultured organism clone 1041059766597 genomic sequence	gi|60650141|ref|NC_006873.1| Bacteroides fragilis NCTC 9343 plasmid pBF9343, complete sequence	gi|60650141|ref|NC_006873.1| Bacteroides fragilis NCTC 9343 plasmid pBF9343, complete sequence
contig-100_1991	852	N	1	0	NA	0	0	0	NA	NA	NA	NA
contig-100_1992	852	N	1	0	NA	0	0	0	NA	Faecalibacterium prausnitzii L2/6 draft genome	gi|479208076|ref|NC_021042.1| Faecalibacterium prausnitzii L2-6, complete genome	gi|479208076|ref|NC_021042.1| Faecalibacterium prausnitzii L2-6, complete genome
contig-100_1993	852	N	0	0	NA	0	0	0	NA	NA	NA	NA
contig-100_1994	851	N	0	0	NA	0	0	0	NA	NA	NA	NA
contig-100_1995	850	N	1	0	NA	0	0	0	NA	Haemophilus parainfluenzae T3T1 complete genome	gi|345428590|ref|NC_015964.1| Haemophilus parainfluenzae T3T1, complete genome	gi|345428590|ref|NC_015964.1| Haemophilus parainfluenzae T3T1, complete genome

contig-100_1996	850	N	0	0	NA	0	0	0	NA	Faecalibacterium prausnitzii SL3/3 draft genome	gi|479170689|ref|NC_021020.1| Faecalibacterium prausnitzii SL3/3 draft genome	gi|479170689|ref|NC_021020.1| Faecalibacterium prausnitzii SL3/3 draft genome
contig-100_1997	850	N	0	0	NA	0	0	0	NA	NA	NA	NA
contig-100_1998	850	N	2	1	Podo	0	0	1	NA	NA	NA	NA
contig-100_20	13492	N	12	6	Sipho	1	1	7	NA	NA	NA	NA
contig-100_200	3400	N	3	1	Sipho	1	0	1	NA	Ruminococcus torques L2-14 draft genome	gi|479155735|ref|NC_021015.1| Ruminococcus torques L2-14 draft genome	gi|479155735|ref|NC_021015.1| Ruminococcus torques L2-14 draft genome
contig-100_2000	849	N	1	0	NA	0	0	0	NA	Uncultured organism clone VC1CX78TF genomic sequence	NA	Uncultured organism clone VC1CX78TF genomic sequence
contig-100_2001	849	N	0	0	NA	0	0	0	NA	Clostridium saccharolyticum-like K10 draft genome	gi|479336697|ref|NC_021047.1| Clostridium cf. saccharolyticum K10, complete genome	gi|479336697|ref|NC_021047.1| Clostridium cf. saccharolyticum K10, complete genome
contig-100_2002	849	N	0	0	NA	0	0	0	NA	Uncultured organism clone 1041059766979 genomic sequence	gi|53711291|ref|NC_006347.1| Bacteroides fragilis YCH46 DNA, complete genome	gi|53711291|ref|NC_006347.1| Bacteroides fragilis YCH46 DNA, complete genome
contig-100_2004	849	N	0	0	NA	0	0	0	NA	Faecalibacterium prausnitzii SL3/3 draft genome	gi|479170689|ref|NC_021020.1| Faecalibacterium prausnitzii SL3/3 draft genome	gi|479170689|ref|NC_021020.1| Faecalibacterium prausnitzii SL3/3 draft genome
contig-100_2006	848	N	0	0	NA	0	0	0	NA	NA	NA	NA

contig-100_2008	848	N	0	0	NA	0	0	0	NA	Roseburia intestinalis M50/1 draft genome	gi|479201824|ref|NC_021040.1| Roseburia intestinalis M50/1 draft genome	gi|479201824|ref|NC_021040.1| Roseburia intestinalis M50/1 draft genome
contig-100_2009	847	N	0	0	NA	0	0	0	NA	Uncultured bacterium clone LM0ABA27ZA09RM1 genomic sequence	NA	Uncultured bacterium clone LM0ABA27ZA09RM1 genomic sequence
contig-100_201	3396	N	6	2	Sipho	1	1	2	NA	Unidentified phage clone 1013_scaffold47 genomic sequence	NA	Unidentified phage clone 1013_scaffold47 genomic sequence
contig-100_2010	847	N	1	0	NA	0	0	0	NA	NA	NA	NA
contig-100_2011	847	N	0	0	NA	0	0	0	NA	Clostridiales sp. SSC/2 draft genome	gi|479158859|ref|NC_021016.1| Butyrate-producing bacterium SSC/2, complete genome	gi|479158859|ref|NC_021016.1| Butyrate-producing bacterium SSC/2, complete genome
contig-100_2012	846	N	0	0	NA	0	0	0	NA	NA	NA	NA
contig-100_2013	846	N	0	0	NA	0	0	0	NA	NA	NA	NA
contig-100_2015	846	N	0	0	NA	0	0	0	NA	Eubacterium rectale M104/1 draft genome	gi|479213596|ref|NC_021044.1| Eubacterium rectale M104/1 draft genome	gi|479213596|ref|NC_021044.1| Eubacterium rectale M104/1 draft genome
contig-100_2016	845	N	0	0	NA	0	0	0	NA	NA	NA	NA
contig-100_2017	845	N	0	0	NA	0	0	0	NA	Uncultured organism clone VC1CI54TR genomic sequence	NA	Uncultured organism clone VC1CI54TR genomic sequence
contig-100_2018	844	N	1	0	NA	0	0	0	NA	Uncultured organism clone 1041059765743 genomic sequence	gi|53711291|ref|NC_006347.1| Bacteroides fragilis YCH46 DNA, complete genome	gi|53711291|ref|NC_006347.1| Bacteroides fragilis YCH46 DNA, complete genome

contig-100_2019	843	N	0	0	NA	0	0	0	NA	Bacteroides helcogenes P 36-108, complete genome	gi|319899888|ref|NC_014933.1| Bacteroides helcogenes P 36-108 chromosome, complete genome	gi|319899888|ref|NC_014933.1| Bacteroides helcogenes P 36-108 chromosome, complete genome
contig-100_2021	843	N	0	0	NA	0	0	0	NA	Eubacterium rectale ATCC 33656, complete genome	gi|238922432|ref|NC_012781.1| Eubacterium rectale ATCC 33656, complete genome	gi|238922432|ref|NC_012781.1| Eubacterium rectale ATCC 33656, complete genome
contig-100_2022	843	N	0	0	NA	0	0	0	NA	Propionibacterium acnes TypeIA2 P.acn17, complete genome	gi|365961730|ref|NC_016511.1| Propionibacterium acnes TypeIA2 P.acn31 chromosome, complete genome	gi|365961730|ref|NC_016511.1| Propionibacterium acnes TypeIA2 P.acn31 chromosome, complete genome
contig-100_2023	842	N	0	0	NA	0	0	0	NA	NA	NA	NA
contig-100_2024	842	N	1	0	NA	0	0	0	NA	Eubacterium rectale DSM 17629 draft genome	gi|479140210|ref|NC_021010.1| Eubacterium rectale DSM 17629 draft genome	gi|479140210|ref|NC_021010.1| Eubacterium rectale DSM 17629 draft genome
contig-100_2025	842	N	0	0	NA	0	0	0	NA	NA	NA	NA
contig-100_2026	842	N	0	0	NA	0	0	0	NA	NA	NA	NA
contig-100_2027	841	N	0	0	NA	0	0	0	NA	Propionibacterium acidipropionici ATCC 4875, complete genome	gi|410864597|ref|NC_019395.1| Propionibacterium acidipropionici ATCC 4875 chromosome, complete genome	gi|410864597|ref|NC_019395.1| Propionibacterium acidipropionici ATCC 4875 chromosome, complete genome
contig-100_2028	841	N	1	0	NA	0	0	0	NA	NA	NA	NA
contig-100_203	3361	N	3	0	NA	0	0	0	NA	NA	NA	NA
contig-100_2030	841	N	0	0	NA	0	0	0	NA	NA	NA	NA

contig-100_2032	841	N	1	0	NA	0	0	0	NA	Lactobacillus casei LOCK919, complete genome	gi|523512490|ref|NC_021721.1| Lactobacillus casei LOCK919, complete genome	gi|523512490|ref|NC_021721.1| Lactobacillus casei LOCK919, complete genome
contig-100_2033	840	N	1	0	NA	0	0	0	NA	Bacteroides thetaiotaomicron VPI-5482, complete genome	gi|29345410|ref|NC_004663.1| Bacteroides thetaiotaomicron VPI-5482 chromosome, complete genome	gi|29345410|ref|NC_004663.1| Bacteroides thetaiotaomicron VPI-5482 chromosome, complete genome
contig-100_2036	839	N	1	0	NA	0	0	0	NA	NA	NA	NA
contig-100_2037	839	N	0	0	NA	0	0	0	NA	NA	NA	NA
contig-100_2038	838	N	0	0	NA	0	0	0	NA	NA	NA	NA
contig-100_2039	838	N	0	0	NA	0	0	0	NA	NA	NA	NA
contig-100_2040	838	N	0	0	NA	0	0	0	NA	Bacteroides vulgatus ATCC 8482, complete genome	gi|150002608|ref|NC_009614.1| Bacteroides vulgatus ATCC 8482 chromosome, complete genome	gi|150002608|ref|NC_009614.1| Bacteroides vulgatus ATCC 8482 chromosome, complete genome
contig-100_2041	837	N	1	0	NA	0	0	0	NA	NA	NA	NA
contig-100_2044	836	N	0	0	NA	0	0	0	NA	NA	NA	NA
contig-100_2045	835	N	1	0	NA	0	0	0	NA	Prevotella dentalis DSM 3688 plasmid pPREDE01, complete sequence	gi|433653593|ref|NC_019961.1| Prevotella dentalis DSM 3688 plasmid pPREDE01, complete sequence	gi|433653593|ref|NC_019961.1| Prevotella dentalis DSM 3688 plasmid pPREDE01, complete sequence
contig-100_2046	835	N	2	0	NA	0	0	0	NA	NA	NA	NA
contig-100_2047	835	N	0	0	NA	0	0	0	NA	NA	NA	NA
contig-100_2048	835	N	1	0	NA	0	0	0	NA	Faecalibacterium prausnitzii SL3/3 draft genome	gi|479170689|ref|NC_021020.1| Faecalibacterium prausnitzii SL3/3 draft genome	gi|479170689|ref|NC_021020.1| Faecalibacterium prausnitzii SL3/3 draft genome

contig-100_2049	835	N	0	0	NA	0	0	0	NA	Uncultured bacterium clone HA0AAA9ZH06FM1 genomic sequence	NA	Uncultured bacterium clone HA0AAA9ZH06FM1 genomic sequence
contig-100_205	3359	N	3	0	NA	0	0	0	NA	Streptococcus pneumoniae SPNA45, complete genome	gi|405759923|ref|NC_018594.1| Streptococcus pneumoniae SPNA45, complete genome	gi|405759923|ref|NC_018594.1| Streptococcus pneumoniae SPNA45, complete genome
contig-100_2050	833	N	1	0	NA	0	0	0	NA	NA	NA	NA
contig-100_2052	833	N	0	0	NA	0	0	0	NA	NA	NA	NA
contig-100_2053	833	N	0	0	NA	0	0	0	NA	NA	NA	NA
contig-100_2054	833	N	0	0	NA	0	0	0	NA	Haemophilus parainfluenzae T3T1 complete genome	gi|345428590|ref|NC_015964.1| Haemophilus parainfluenzae T3T1, complete genome	gi|345428590|ref|NC_015964.1| Haemophilus parainfluenzae T3T1, complete genome
contig-100_2055	833	N	0	0	NA	0	0	0	NA	Uncultured organism clone 1041059767137 genomic sequence	gi|327312315|ref|NC_015311.1| Prevotella denticola F0289 chromosome, complete genome	gi|327312315|ref|NC_015311.1| Prevotella denticola F0289 chromosome, complete genome
contig-100_2056	832	N	1	0	NA	0	0	0	NA	NA	NA	NA
contig-100_2057	832	N	0	0	NA	0	0	0	NA	Bacteroides vulgatus ATCC 8482, complete genome	gi|150002608|ref|NC_009614.1| Bacteroides vulgatus ATCC 8482 chromosome, complete genome	gi|150002608|ref|NC_009614.1| Bacteroides vulgatus ATCC 8482 chromosome, complete genome
contig-100_2058	832	N	0	0	NA	0	0	0	NA	NA	NA	NA
contig-100_2059	832	N	1	0	NA	0	0	0	NA	NA	NA	NA

contig-100_206	3342	N	3	0	NA	0	0	0	NA	Bacteroides fragilis plasmid pBFUK1 DNA, complete genome, strain: GAI92082	NA	Bacteroides fragilis plasmid pBFUK1 DNA, complete genome, strain: GAI92082
contig-100_2060	832	N	2	0	NA	0	0	0	NA	NA	NA	NA
contig-100_2061	832	N	0	0	NA	0	0	0	NA	TPA_exp: Clostridium difficile strain QCD-66C26 transposon Tn6110, complete sequence	gi|260685375|ref|NC_013316.1| Clostridium difficile R20291 chromosome, complete genome	gi|260685375|ref|NC_013316.1| Clostridium difficile R20291 chromosome, complete genome
contig-100_2062	832	N	1	0	NA	0	0	0	NA	NA	NA	NA
contig-100_2063	831	N	0	0	NA	0	0	0	NA	NA	NA	NA
contig-100_2064	830	N	0	0	NA	0	0	0	NA	NA	NA	NA
contig-100_2065	830	N	0	0	NA	0	0	0	NA	Faecalibacterium prausnitzii L2/6 draft genome	gi|479208076|ref|NC_021042.1| Faecalibacterium prausnitzii L2-6, complete genome	gi|479208076|ref|NC_021042.1| Faecalibacterium prausnitzii L2-6, complete genome
contig-100_2066	830	N	0	0	NA	0	0	0	NA	NA	NA	NA
contig-100_2067	830	N	1	0	NA	0	0	0	NA	Faecalibacterium prausnitzii L2/6 draft genome	gi|479208076|ref|NC_021042.1| Faecalibacterium prausnitzii L2-6, complete genome	gi|479208076|ref|NC_021042.1| Faecalibacterium prausnitzii L2-6, complete genome
contig-100_2068	829	N	0	0	NA	0	0	0	NA	Haemophilus parainfluenzae T3T1 complete genome	gi|345428590|ref|NC_015964.1| Haemophilus parainfluenzae T3T1, complete genome	gi|345428590|ref|NC_015964.1| Haemophilus parainfluenzae T3T1, complete genome

contig-100_2069	829	N	0	0	NA	0	0	0	NA	Bacteroides vulgatus ATCC 8482, complete genome	gi|150002608|ref|NC_009614.1| Bacteroides vulgatus ATCC 8482 chromosome, complete genome	gi|150002608|ref|NC_009614.1| Bacteroides vulgatus ATCC 8482 chromosome, complete genome
contig-100_207	3338	N	2	0	NA	0	0	1	NA	Clostridiales sp. SSC/2 draft genome	gi|479158859|ref|NC_021016.1| Butyrate-producing bacterium SSC/2, complete genome	gi|479158859|ref|NC_021016.1| Butyrate-producing bacterium SSC/2, complete genome
contig-100_2073	828	N	0	0	NA	0	0	0	NA	Eubacterium rectale DSM 17629 draft genome	gi|479140210|ref|NC_021010.1| Eubacterium rectale DSM 17629 draft genome	gi|479140210|ref|NC_021010.1| Eubacterium rectale DSM 17629 draft genome
contig-100_2074	828	N	1	0	NA	0	0	0	NA	Eubacterium rectale DSM 17629 draft genome	gi|479140210|ref|NC_021010.1| Eubacterium rectale DSM 17629 draft genome	gi|479140210|ref|NC_021010.1| Eubacterium rectale DSM 17629 draft genome
contig-100_2075	828	N	0	0	NA	0	0	0	NA	NA	NA	NA
contig-100_2076	828	N	0	0	NA	0	0	0	NA	NA	NA	NA
contig-100_2077	828	N	0	0	NA	0	0	0	NA	Eubacterium eligens ATCC 27750 plasmid, complete sequence	gi|238921767|ref|NC_012780.1| Eubacterium eligens ATCC 27750 plasmid unnamed, complete sequence	gi|238921767|ref|NC_012780.1| Eubacterium eligens ATCC 27750 plasmid unnamed, complete sequence
contig-100_2078	827	N	1	0	NA	0	0	0	NA	NA	NA	NA

contig-100_2079	827	N	0	0	NA	0	0	0	NA	Bacteroides vulgatus ATCC 8482, complete genome	gi|150002608|ref|NC_009614.1| Bacteroides vulgatus ATCC 8482 chromosome, complete genome	gi|150002608|ref|NC_009614.1| Bacteroides vulgatus ATCC 8482 chromosome, complete genome
contig-100_2080	827	N	0	0	NA	0	0	0	NA	Roseburia hominis A2-183, complete genome	gi|347530298|ref|NC_015977.1| Roseburia hominis A2-183 chromosome, complete genome	gi|347530298|ref|NC_015977.1| Roseburia hominis A2-183 chromosome, complete genome
contig-100_2081	826	N	0	0	NA	0	0	0	NA	Campylobacter coli strain 6461 putative ExpD, ExpB, hypothetical proteins, TetO, putative periplasmic protein, hypothetical protein, and serine transporter genes, complete cds	gi|298345126|ref|NC_014246.1| Mobiluncus curtisii ATCC 43063 chromosome, complete genome	gi|298345126|ref|NC_014246.1| Mobiluncus curtisii ATCC 43063 chromosome, complete genome
contig-100_2083	826	N	0	0	NA	0	0	0	NA	NA	NA	NA
contig-100_2084	825	N	0	0	NA	0	0	0	NA	NA	NA	NA
contig-100_2085	825	N	1	0	NA	0	0	0	NA	NA	NA	NA
contig-100_2086	825	N	1	0	NA	0	0	0	NA	Uncultured organism clone 1041059766037 genomic sequence	NA	Uncultured organism clone 1041059766037 genomic sequence
contig-100_2087	825	N	0	0	NA	0	0	0	NA	NA	NA	NA
contig-100_2088	824	N	0	0	NA	0	0	0	NA	Bacteroides vulgatus ATCC 8482, complete genome	gi|150002608|ref|NC_009614.1| Bacteroides vulgatus ATCC 8482 chromosome, complete genome	gi|150002608|ref|NC_009614.1| Bacteroides vulgatus ATCC 8482 chromosome, complete genome
contig-100_2089	824	N	0	0	NA	0	0	0	NA	NA	NA	NA

contig-100_2090	824	N	2	0	NA	0	0	0	NA	Propionibacterium acnes HL096PA1, complete genome	gi|365972921|ref|NC_016516.1| Propionibacterium acnes TypeIA2 P.acn33 chromosome, complete genome	gi|365972921|ref|NC_016516.1| Propionibacterium acnes TypeIA2 P.acn33 chromosome, complete genome
contig-100_2091	824	N	0	0	NA	0	0	0	NA	Faecalibacterium prausnitzii L2/6 draft genome	gi|479208076|ref|NC_021042.1| Faecalibacterium prausnitzii L2-6, complete genome	gi|479208076|ref|NC_021042.1| Faecalibacterium prausnitzii L2-6, complete genome
contig-100_2093	823	N	0	0	NA	0	0	0	NA	NA	NA	NA
contig-100_2094	823	N	0	0	NA	0	0	0	NA	Faecalibacterium prausnitzii SL3/3 draft genome	gi|479170689|ref|NC_021020.1| Faecalibacterium prausnitzii SL3/3 draft genome	gi|479170689|ref|NC_021020.1| Faecalibacterium prausnitzii SL3/3 draft genome
contig-100_2095	823	N	0	0	NA	0	0	0	NA	NA	NA	NA
contig-100_2097	823	N	0	0	NA	0	0	0	NA	NA	NA	NA
contig-100_2098	822	N	0	0	NA	0	0	0	NA	NA	NA	NA
contig-100_21	13291	N	9	6	Podo	0	0	6	NA	NA	NA	NA
contig-100_210	3327	N	1	0	NA	0	0	0	NA	NA	NA	NA
contig-100_2100	821	N	0	0	NA	0	0	0	NA	NA	NA	NA
contig-100_2101	821	N	0	0	NA	0	0	0	NA	NA	NA	NA
contig-100_2102	821	N	2	0	NA	0	0	0	NA	NA	NA	NA
contig-100_2103	821	N	0	0	NA	0	0	0	NA	NA	NA	NA
contig-100_2104	820	N	0	0	NA	0	0	0	NA	NA	NA	NA
contig-100_2105	820	N	0	0	NA	0	0	0	NA	NA	NA	NA
contig-100_2106	819	N	2	0	NA	0	0	0	NA	NA	NA	NA
contig-100_2107	819	N	2	0	NA	0	0	0	NA	NA	NA	NA
contig-100_2108	819	N	1	0	NA	0	0	0	NA	NA	NA	NA

contig-100_2109	819	N	1	0	NA	0	0	0	NA	Bacteroides vulgatus ATCC 8482, complete genome	gi|150002608|ref|NC_009614.1| Bacteroides vulgatus ATCC 8482 chromosome, complete genome	gi|150002608|ref|NC_009614.1| Bacteroides vulgatus ATCC 8482 chromosome, complete genome
contig-100_2110	819	N	0	0	NA	0	0	0	NA	NA	NA	NA
contig-100_2112	818	N	0	0	NA	0	0	0	NA	Clostridium saccharolyticum-like K10 draft genome	gi|479336697|ref|NC_021047.1| Clostridium cf. saccharolyticum K10, complete genome	gi|479336697|ref|NC_021047.1| Clostridium cf. saccharolyticum K10, complete genome
contig-100_2113	818	N	0	0	NA	0	0	0	NA	Bacteroides vulgatus ATCC 8482, complete genome	gi|150002608|ref|NC_009614.1| Bacteroides vulgatus ATCC 8482 chromosome, complete genome	gi|150002608|ref|NC_009614.1| Bacteroides vulgatus ATCC 8482 chromosome, complete genome
contig-100_2114	818	N	0	0	NA	0	0	0	NA	Tannerella forsythia ATCC 43037, complete genome	gi|375253814|ref|NC_016610.1| Tannerella forsythia ATCC 43037 chromosome, complete genome	gi|375253814|ref|NC_016610.1| Tannerella forsythia ATCC 43037 chromosome, complete genome
contig-100_2115	818	N	0	0	NA	0	0	0	NA	Unidentified phage clone 1013_scaffold47 genomic sequence	NA	Unidentified phage clone 1013_scaffold47 genomic sequence
contig-100_2117	818	N	0	0	NA	0	0	0	NA	Propionibacterium acnes HL096PA1, complete genome	gi|295129529|ref|NC_014039.1| Propionibacterium acnes SK137 chromosome, complete genome	gi|295129529|ref|NC_014039.1| Propionibacterium acnes SK137 chromosome, complete genome

contig-100_2118	817	N	1	0	NA	0	0	0	NA	NA	NA	NA
contig-100_2119	817	N	0	0	NA	0	0	0	NA	Bacteroides vulgatus ATCC 8482, complete genome	gi|150002608|ref|NC_009614.1| Bacteroides vulgatus ATCC 8482 chromosome, complete genome	gi|150002608|ref|NC_009614.1| Bacteroides vulgatus ATCC 8482 chromosome, complete genome
contig-100_212	3323	N	2	0	NA	0	0	0	NA	Uncultured organism clone VC1D729TF genomic sequence	NA	Uncultured organism clone VC1D729TF genomic sequence
contig-100_2121	816	N	0	0	NA	0	0	0	NA	Haemophilus parainfluenzae T3T1 complete genome	gi|345428590|ref|NC_015964.1| Haemophilus parainfluenzae T3T1, complete genome	gi|345428590|ref|NC_015964.1| Haemophilus parainfluenzae T3T1, complete genome
contig-100_2122	816	N	1	0	NA	0	0	0	NA	NA	NA	NA
contig-100_2123	816	N	0	0	NA	0	0	0	NA	NA	NA	NA
contig-100_2124	816	N	0	0	NA	0	0	0	NA	Faecalibacterium prausnitzii L2/6 draft genome	gi|479208076|ref|NC_021042.1| Faecalibacterium prausnitzii L2-6, complete genome	gi|479208076|ref|NC_021042.1| Faecalibacterium prausnitzii L2-6, complete genome
contig-100_2125	816	N	0	0	NA	0	0	0	NA	Faecalibacterium prausnitzii L2/6 draft genome	gi|479208076|ref|NC_021042.1| Faecalibacterium prausnitzii L2-6, complete genome	gi|479208076|ref|NC_021042.1| Faecalibacterium prausnitzii L2-6, complete genome
contig-100_2128	815	N	1	0	NA	0	0	0	NA	Thauera sp. MZ1T, complete genome	gi|237653092|ref|NC_011662.2| Thauera sp. MZ1T chromosome, complete genome	gi|237653092|ref|NC_011662.2| Thauera sp. MZ1T chromosome, complete genome
contig-100_2129	814	N	1	0	NA	0	0	0	NA	NA	NA	NA

contig-100_213	3320	N	3	0	NA	0	1	0	NA	Uncultured organism clone 1041059766313 genomic sequence	NA	Uncultured organism clone 1041059766313 genomic sequence
contig-100_2130	814	N	1	0	NA	0	0	0	NA	NA	NA	NA
contig-100_2131	814	N	0	0	NA	0	0	0	NA	Haemophilus parainfluenzae T3T1 complete genome	gi|345428590|ref|NC_015964.1| Haemophilus parainfluenzae T3T1, complete genome	gi|345428590|ref|NC_015964.1| Haemophilus parainfluenzae T3T1, complete genome
contig-100_2133	814	N	1	0	NA	0	0	0	NA	Faecalibacterium prausnitzii L2/6 draft genome	gi|479208076|ref|NC_021042.1| Faecalibacterium prausnitzii L2-6, complete genome	gi|479208076|ref|NC_021042.1| Faecalibacterium prausnitzii L2-6, complete genome
contig-100_2134	814	N	0	0	NA	0	0	0	NA	NA	NA	NA
contig-100_2135	814	N	0	0	NA	0	0	0	NA	Bacteroides fragilis NCTC 9343 pBF9343 plasmid complete sequence	gi|60650141|ref|NC_006873.1| Bacteroides fragilis NCTC 9343 plasmid pBF9343, complete sequence	gi|60650141|ref|NC_006873.1| Bacteroides fragilis NCTC 9343 plasmid pBF9343, complete sequence
contig-100_2136	814	N	0	0	NA	0	0	0	NA	NA	NA	NA
contig-100_2137	813	N	0	0	NA	0	0	0	NA	Faecalibacterium prausnitzii SL3/3 draft genome	gi|479170689|ref|NC_021020.1| Faecalibacterium prausnitzii SL3/3 draft genome	gi|479170689|ref|NC_021020.1| Faecalibacterium prausnitzii SL3/3 draft genome
contig-100_2138	813	N	1	0	NA	0	0	0	NA	Uncultured bacterium clone LM0ABA33ZF03FM1 genomic sequence	NA	Uncultured bacterium clone LM0ABA33ZF03FM1 genomic sequence

contig-100_2139	812	N	0	0	NA	0	0	0	NA	Odoribacter splanchnicus DSM 20712, complete genome	gi|325278757|ref|NC_015160.1| Odoribacter splanchnicus DSM 20712 chromosome, complete genome	gi|325278757|ref|NC_015160.1| Odoribacter splanchnicus DSM 20712 chromosome, complete genome
contig-100_2140	812	N	0	0	NA	0	0	0	NA	NA	NA	NA
contig-100_2142	812	N	0	0	NA	0	0	0	NA	NA	NA	NA
contig-100_2143	812	N	0	0	NA	0	0	0	NA	NA	NA	NA
contig-100_2144	812	N	1	0	NA	0	0	0	NA	NA	NA	NA
contig-100_2145	811	N	0	0	NA	0	0	0	NA	NA	NA	NA
contig-100_2147	811	N	0	0	NA	0	0	0	NA	Clostridiales sp. SM4/1 draft genome	gi|479170689|ref|NC_021020.1| Faecalibacterium prausnitzii SL3/3 draft genome	gi|479170689|ref|NC_021020.1| Faecalibacterium prausnitzii SL3/3 draft genome
contig-100_2148	810	N	1	0	NA	0	0	0	NA	NA	NA	NA
contig-100_2149	810	N	0	0	NA	0	0	0	NA	NA	NA	NA
contig-100_215	3274	N	2	0	NA	0	0	0	NA	Uncultured organism clone 1041059767304 genomic sequence	NA	Uncultured organism clone 1041059767304 genomic sequence
contig-100_2150	810	N	0	0	NA	0	0	0	NA	Enterococcus hirae ATCC 9790, complete genome	gi|392987295|ref|NC_018081.1| Enterococcus hirae ATCC 9790 chromosome, complete genome	gi|392987295|ref|NC_018081.1| Enterococcus hirae ATCC 9790 chromosome, complete genome
contig-100_2151	810	N	1	0	NA	0	0	0	NA	Uncultured organism clone VC1D713TR genomic sequence	gi|479176048|ref|NC_021022.1| Ruminococcus obeum A2-162 draft genome	gi|479176048|ref|NC_021022.1| Ruminococcus obeum A2-162 draft genome

contig-100_2153	809	N	0	0	NA	0	0	0	NA	Faecalibacterium prausnitzii L2/6 draft genome	gi|479208076|ref|NC_021042.1| Faecalibacterium prausnitzii L2-6, complete genome	gi|479208076|ref|NC_021042.1| Faecalibacterium prausnitzii L2-6, complete genome
contig-100_2154	808	N	0	0	NA	0	0	0	NA	NA	NA	NA
contig-100_2155	808	N	0	0	NA	0	0	0	NA	NA	NA	NA
contig-100_2156	807	N	0	0	NA	0	0	0	NA	NA	NA	NA
contig-100_2157	807	N	0	0	NA	0	0	0	NA	NA	NA	NA
contig-100_2159	806	N	0	0	NA	0	0	0	NA	Bacteroides vulgatus ATCC 8482, complete genome	gi|150002608|ref|NC_009614.1| Bacteroides vulgatus ATCC 8482 chromosome, complete genome	gi|150002608|ref|NC_009614.1| Bacteroides vulgatus ATCC 8482 chromosome, complete genome
contig-100_216	3273	N	2	0	NA	0	0	0	NA	NA	NA	NA
contig-100_2160	806	N	0	0	NA	0	0	0	NA	Roseburia intestinalis M50/1 draft genome	gi|479201824|ref|NC_021040.1| Roseburia intestinalis M50/1 draft genome	gi|479201824|ref|NC_021040.1| Roseburia intestinalis M50/1 draft genome
contig-100_2161	805	N	0	0	NA	0	0	0	NA	Streptococcus salivarius JIM8777 complete genome	gi|387783149|ref|NC_017595.1| Streptococcus salivarius JIM8777, complete genome	gi|387783149|ref|NC_017595.1| Streptococcus salivarius JIM8777, complete genome
contig-100_2162	805	N	2	0	NA	0	0	0	NA	Bacteroides xylanisolvens XB1A draft genome	gi|479162165|ref|NC_021017.1| Bacteroides xylanisolvens XB1A draft genome	gi|479162165|ref|NC_021017.1| Bacteroides xylanisolvens XB1A draft genome
contig-100_2163	805	N	0	0	NA	0	0	0	NA	Bacteroides fragilis 638R genome	gi|375356399|ref|NC_016776.1| Bacteroides fragilis 638R, complete genome	gi|375356399|ref|NC_016776.1| Bacteroides fragilis 638R, complete genome

contig-100_2164	804	N	0	0	NA	0	0	0	NA	NA	NA	NA
contig-100_2165	804	N	0	0	NA	0	0	0	NA	NA	NA	NA
contig-100_2166	803	N	0	0	NA	0	0	0	NA	NA	NA	NA
contig-100_2168	802	N	1	0	NA	0	0	0	NA	NA	NA	NA
contig-100_2169	802	N	0	0	NA	0	0	0	NA	Uncultured organism clone VC1CB72TR genomic sequence	NA	Uncultured organism clone VC1CB72TR genomic sequence
contig-100_217	3231	N	3	1	Myo	0	0	1	NA	Unidentified phage clone 1013_scaffold1563 genomic sequence	NA	Unidentified phage clone 1013_scaffold 1563 genomic sequence
contig-100_2170	802	N	0	0	NA	0	0	0	NA	NA	NA	NA
contig-100_2171	802	N	1	0	NA	0	0	0	NA	NA	NA	NA
contig-100_2172	801	N	0	0	NA	0	0	0	NA	NA	NA	NA
contig-100_2173	801	N	0	0	NA	0	0	0	NA	NA	NA	NA
contig-100_2174	801	N	0	0	NA	0	0	0	NA	Bacteroides vulgatus ATCC 8482, complete genome	gi|150002608|ref|NC_009614.1| Bacteroides vulgatus ATCC 8482 chromosome, complete genome	gi|150002608|ref|NC_009614.1| Bacteroides vulgatus ATCC 8482 chromosome, complete genome
contig-100_2175	801	N	0	0	NA	0	0	0	NA	Bacteroides fragilis 638R genome	gi|53711291|ref|NC_006347.1| Bacteroides fragilis YCH46 DNA, complete genome	gi|53711291|ref|NC_006347.1| Bacteroides fragilis YCH46 DNA, complete genome
contig-100_2176	801	N	0	0	NA	0	0	0	NA	NA	NA	NA
contig-100_2177	801	N	0	0	NA	0	0	0	NA	NA	NA	NA
contig-100_2178	801	N	0	0	NA	0	0	0	NA	Bacteroides fragilis 638R genome	gi|375356399|ref|NC_016776.1| Bacteroides fragilis 638R, complete genome	gi|375356399|ref|NC_016776.1| Bacteroides fragilis 638R, complete genome
contig-100_2179	801	N	0	0	NA	0	0	0	NA	NA	NA	NA

contig-100_2180	800	N	0	0	NA	0	0	0	NA	Bacteroides salanitronis DSM 18170, complete genome	gi|325297172|ref|NC_015164.1| Bacteroides salanitronis DSM 18170 chromosome, complete genome	gi|325297172|ref|NC_015164.1| Bacteroides salanitronis DSM 18170 chromosome, complete genome
contig-100_2181	800	N	0	0	NA	0	0	0	NA	Odoribacter splanchnicus DSM 20712, complete genome	gi|325278757|ref|NC_015160.1| Odoribacter splanchnicus DSM 20712 chromosome, complete genome	gi|325278757|ref|NC_015160.1| Odoribacter splanchnicus DSM 20712 chromosome, complete genome
contig-100_2182	800	N	0	0	NA	0	0	0	NA	Bacteroides thetaiotaomicron VPI-5482, complete genome	gi|29345410|ref|NC_004663.1| Bacteroides thetaiotaomicron VPI-5482 chromosome, complete genome	gi|29345410|ref|NC_004663.1| Bacteroides thetaiotaomicron VPI-5482 chromosome, complete genome
contig-100_2183	800	N	0	0	NA	0	0	0	NA	NA	NA	NA
contig-100_2186	798	N	0	0	NA	0	0	0	NA	NA	NA	NA
contig-100_2188	798	N	2	0	NA	0	0	0	NA	Faecalibacterium prausnitzii SL3/3 draft genome	gi|479170689|ref|NC_021020.1| Faecalibacterium prausnitzii SL3/3 draft genome	gi|479170689|ref|NC_021020.1| Faecalibacterium prausnitzii SL3/3 draft genome
contig-100_2190	798	N	0	0	NA	0	0	0	NA	NA	NA	NA
contig-100_2191	798	N	1	0	NA	0	0	0	NA	NA	NA	NA
contig-100_2192	797	N	0	0	NA	0	0	0	NA	NA	NA	NA
contig-100_2193	797	N	0	0	NA	0	0	0	NA	NA	NA	NA
contig-100_2194	797	N	2	0	NA	0	0	0	NA	NA	NA	NA
contig-100_2195	796	N	0	0	NA	0	0	0	NA	NA	NA	NA

contig-100_2196	796	N	0	0	NA	0	0	0	NA	Bacteroides vulgatus ATCC 8482, complete genome	gi|150002608|ref|NC_009614.1| Bacteroides vulgatus ATCC 8482 chromosome, complete genome	gi|150002608|ref|NC_009614.1| Bacteroides vulgatus ATCC 8482 chromosome, complete genome
contig-100_2197	796	N	0	0	NA	0	0	0	NA	NA	NA	NA
contig-100_2198	796	N	0	0	NA	0	0	0	NA	Faecalibacterium prausnitzii L2/6 draft genome	gi|479208076|ref|NC_021042.1| Faecalibacterium prausnitzii L2-6, complete genome	gi|479208076|ref|NC_021042.1| Faecalibacterium prausnitzii L2-6, complete genome
contig-100_2199	795	N	0	0	NA	0	0	0	NA	NA	NA	NA
contig-100_22	12889	N	10	0	NA	0	0	0	NA	Uncultured organism clone 1041059764585 genomic sequence	gi|150002608|ref|NC_009614.1| Bacteroides vulgatus ATCC 8482 chromosome, complete genome	gi|150002608|ref|NC_009614.1| Bacteroides vulgatus ATCC 8482 chromosome, complete genome
contig-100_2201	794	N	1	0	NA	0	0	0	NA	Uncultured organism clone VC1C662TF genomic sequence	gi|479170689|ref|NC_021020.1| Faecalibacterium prausnitzii SL3/3 draft genome	gi|479170689|ref|NC_021020.1| Faecalibacterium prausnitzii SL3/3 draft genome
contig-100_2202	794	N	0	0	NA	0	0	0	NA	Bacteroides vulgatus ATCC 8482, complete genome	gi|150002608|ref|NC_009614.1| Bacteroides vulgatus ATCC 8482 chromosome, complete genome	gi|150002608|ref|NC_009614.1| Bacteroides vulgatus ATCC 8482 chromosome, complete genome

contig-100_2203	793	N	2	0	NA	0	0	0	NA	Bacteroides vulgatus ATCC 8482, complete genome	gi|150002608|ref|NC_009614.1| Bacteroides vulgatus ATCC 8482 chromosome, complete genome	gi|150002608|ref|NC_009614.1| Bacteroides vulgatus ATCC 8482 chromosome, complete genome
contig-100_2204	793	N	0	0	NA	0	0	0	NA	NA	NA	NA
contig-100_2205	793	N	0	0	NA	0	0	0	NA	NA	NA	NA
contig-100_2207	793	N	0	0	NA	0	0	0	NA	NA	NA	NA
contig-100_2208	793	N	0	0	NA	0	0	0	NA	Faecalibacterium prausnitzii L2/6 draft genome	gi|479208076|ref|NC_021042.1| Faecalibacterium prausnitzii L2-6, complete genome	gi|479208076|ref|NC_021042.1| Faecalibacterium prausnitzii L2-6, complete genome
contig-100_2209	792	N	1	0	NA	0	0	0	NA	NA	NA	NA
contig-100_2210	792	N	0	0	NA	0	0	0	NA	NA	NA	NA
contig-100_2213	792	N	0	0	NA	0	0	0	NA	NA	NA	NA
contig-100_2216	791	N	0	0	NA	0	0	0	NA	NA	NA	NA
contig-100_2217	791	N	0	0	NA	0	0	0	NA	NA	NA	NA
contig-100_2218	791	N	1	0	NA	0	0	0	NA	NA	NA	NA
contig-100_2219	790	N	0	0	NA	0	0	0	NA	NA	NA	NA
contig-100_222	3191	N	3	1	Myo	0	0	1	NA	Bacteroides vulgatus ATCC 8482, complete genome	gi|150002608|ref|NC_009614.1| Bacteroides vulgatus ATCC 8482 chromosome, complete genome	gi|150002608|ref|NC_009614.1| Bacteroides vulgatus ATCC 8482 chromosome, complete genome
contig-100_2220	790	N	0	0	NA	0	0	0	NA	NA	NA	NA
contig-100_2221	790	N	0	0	NA	0	0	0	NA	NA	NA	NA
contig-100_2222	790	N	0	0	NA	0	0	0	NA	NA	NA	NA
contig-100_2223	789	N	0	0	NA	0	0	0	NA	NA	NA	NA
contig-100_2224	789	N	0	0	NA	0	0	0	NA	Bacteroides vulgatus ATCC 8482, complete genome	gi|150002608|ref|NC_009614.1| Bacteroides vulgatus ATCC 8482 chromosome, complete genome	gi|150002608|ref|NC_009614.1| Bacteroides vulgatus ATCC 8482 chromosome, complete genome

contig-100_2225	789	N	0	0	NA	0	0	0	NA	Alistipes finegoldii DSM 17242, complete genome	gi|390945347|ref|NC_018011.1| Alistipes finegoldii DSM 17242 chromosome, complete genome	gi|390945347|ref|NC_018011.1| Alistipes finegoldii DSM 17242 chromosome, complete genome
contig-100_2226	789	N	0	0	NA	0	0	0	NA	NA	NA	NA
contig-100_2227	788	N	0	0	NA	0	0	0	NA	NA	NA	NA
contig-100_2228	788	N	1	0	NA	0	0	0	NA	NA	NA	NA
contig-100_2229	788	N	0	0	NA	0	0	0	NA	NA	NA	NA
contig-100_2230	788	N	1	0	NA	0	0	0	NA	Eubacterium rectale M104/1 draft genome	gi|479213596|ref|NC_021044.1| Eubacterium rectale M104/1 draft genome	gi|479213596|ref|NC_021044.1| Eubacterium rectale M104/1 draft genome
contig-100_2231	788	N	0	0	NA	0	0	0	NA	NA	NA	NA
contig-100_2232	788	N	0	0	NA	0	0	0	NA	NA	NA	NA
contig-100_2233	787	N	0	0	NA	0	0	0	NA	NA	NA	NA
contig-100_2234	787	N	0	0	NA	0	0	0	NA	Bacteroides vulgatus ATCC 8482, complete genome	gi|150002608|ref|NC_009614.1| Bacteroides vulgatus ATCC 8482 chromosome, complete genome	gi|150002608|ref|NC_009614.1| Bacteroides vulgatus ATCC 8482 chromosome, complete genome
contig-100_2235	787	N	0	0	NA	0	0	0	NA	Ruminococcus obeum A2-162 draft genome	gi|479176048|ref|NC_021022.1| Ruminococcus obeum A2-162 draft genome	gi|479176048|ref|NC_021022.1| Ruminococcus obeum A2-162 draft genome
contig-100_2236	786	N	0	0	NA	0	0	0	NA	Porphyromonas gulae kat gene for catalase HP2, complete cds	gi|56475432|ref|NC_006513.1| Aromatoleum aromaticum EbN1 chromosome, complete genome	gi|56475432|ref|NC_006513.1| Aromatoleum aromaticum EbN1 chromosome, complete genome
contig-100_2237	786	N	1	0	NA	0	0	0	NA	NA	NA	NA
contig-100_2238	786	N	1	0	NA	0	0	0	NA	NA	NA	NA

contig-100_2239	785	N	2	0	NA	0	0	0	NA	Faecalibacterium prausnitzii SL3/3 draft genome	gi|479170689|ref|NC_021020.1| Faecalibacterium prausnitzii SL3/3 draft genome	gi|479170689|ref|NC_021020.1| Faecalibacterium prausnitzii SL3/3 draft genome
contig-100_2240	784	N	0	0	NA	0	0	0	NA	NA	NA	NA
contig-100_2241	784	N	0	0	NA	0	0	0	NA	NA	NA	NA
contig-100_2242	784	N	2	0	NA	0	0	0	NA	NA	NA	NA
contig-100_2243	784	N	0	0	NA	0	0	0	NA	NA	NA	NA
contig-100_2245	784	N	0	0	NA	0	0	0	NA	Faecalibacterium prausnitzii SL3/3 draft genome	gi|479170689|ref|NC_021020.1| Faecalibacterium prausnitzii SL3/3 draft genome	gi|479170689|ref|NC_021020.1| Faecalibacterium prausnitzii SL3/3 draft genome
contig-100_2246	784	N	1	0	NA	0	0	0	NA	Bacteroides vulgatus ATCC 8482, complete genome	gi|150002608|ref|NC_009614.1| Bacteroides vulgatus ATCC 8482 chromosome, complete genome	gi|150002608|ref|NC_009614.1| Bacteroides vulgatus ATCC 8482 chromosome, complete genome
contig-100_2248	784	N	0	0	NA	0	0	0	NA	Uncultured organism clone 1041059765200 genomic sequence	NA	Uncultured organism clone 1041059765200 genomic sequence
contig-100_2249	783	N	0	0	NA	0	0	0	NA	NA	NA	NA
contig-100_2250	783	N	0	0	NA	0	0	0	NA	Haemophilus influenzae 86-028NP, complete genome	gi|162960935|ref|NC_007146.2| Haemophilus influenzae 86-028NP chromosome, complete genome	gi|162960935|ref|NC_007146.2| Haemophilus influenzae 86-028NP chromosome, complete genome
contig-100_2251	783	N	1	0	NA	0	0	0	NA	NA	NA	NA
contig-100_2253	782	N	0	0	NA	0	0	0	NA	NA	NA	NA

contig-100_2254	781	N	0	0	NA	0	0	0	NA	Uncultured organism clone 1041059766582 genomic sequence	gi|319899888|ref|NC_014933.1| Bacteroides helcogenes P 36-108 chromosome, complete genome	gi|319899888|ref|NC_014933.1| Bacteroides helcogenes P 36-108 chromosome, complete genome
contig-100_2255	781	N	1	0	NA	0	0	0	NA	NA	NA	NA
contig-100_2257	781	N	0	0	NA	0	0	0	NA	Ruminococcus obeum A2-162 draft genome	gi|479176048|ref|NC_021022.1| Ruminococcus obeum A2-162 draft genome	gi|479176048|ref|NC_021022.1| Ruminococcus obeum A2-162 draft genome
contig-100_2258	781	N	0	0	NA	0	0	0	NA	NA	NA	NA
contig-100_2259	781	N	2	0	NA	0	0	0	NA	NA	NA	NA
contig-100_2260	781	N	0	0	NA	0	0	0	NA	NA	NA	NA
contig-100_2261	781	N	0	0	NA	0	0	0	NA	NA	NA	NA
contig-100_2262	780	N	1	0	NA	0	0	0	NA	NA	NA	NA
contig-100_2263	780	N	0	0	NA	0	0	0	NA	Bacteroides helcogenes P 36-108, complete genome	gi|319899888|ref|NC_014933.1| Bacteroides helcogenes P 36-108 chromosome, complete genome	gi|319899888|ref|NC_014933.1| Bacteroides helcogenes P 36-108 chromosome, complete genome
contig-100_2264	780	N	0	0	NA	0	0	0	NA	NA	NA	NA
contig-100_2265	779	N	0	0	NA	0	0	0	NA	NA	NA	NA
contig-100_2266	779	N	0	0	NA	0	0	0	NA	Bacteroides vulgatus ATCC 8482, complete genome	gi|150002608|ref|NC_009614.1| Bacteroides vulgatus ATCC 8482 chromosome, complete genome	gi|150002608|ref|NC_009614.1| Bacteroides vulgatus ATCC 8482 chromosome, complete genome
contig-100_2267	779	N	0	0	NA	0	0	0	NA	Escherichia coli str. K-12 substr. MG1655, complete genome	gi|386703215|ref|NC_017663.1| Escherichia coli P12b chromosome, complete genome	gi|386703215|ref|NC_017663.1| Escherichia coli P12b chromosome, complete genome

contig-100_2268	778	N	0	0	NA	0	0	0	NA	NA	NA	NA
contig-100_2269	778	N	0	0	NA	0	0	0	NA	NA	NA	NA
contig-100_227	3158	N	3	1	Sipho	1	0	2	NA	Bifidobacterium longum NCC2705, complete genome	gi|58036264|ref|NC_004307.2| Bifidobacterium longum NCC2705 chromosome, complete genome	gi|58036264|ref|NC_004307.2| Bifidobacterium longum NCC2705 chromosome, complete genome
contig-100_2270	778	N	0	0	NA	0	0	0	NA	Staphylococcus epidermidis plasmid SAP108C, complete sequence	gi|433653600|ref|NC_019969.1| Prevotella dentalis DSM 3688 plasmid pPREDE02, complete sequence	gi|433653600|ref|NC_019969.1| Prevotella dentalis DSM 3688 plasmid pPREDE02, complete sequence
contig-100_2271	778	N	0	0	NA	0	0	0	NA	NA	NA	NA
contig-100_2272	778	N	0	0	NA	0	0	0	NA	NA	NA	NA
contig-100_2273	778	N	0	0	NA	0	0	0	NA	Uncultured organism clone VC1DC74TF genomic sequence	NA	Uncultured organism clone VC1DC74TF genomic sequence
contig-100_2274	778	N	0	0	NA	0	0	0	NA	Haemophilus influenzae F3031 complete genome	gi|319896422|ref|NC_014920.1| Haemophilus influenzae F3031 chromosome, complete genome	gi|319896422|ref|NC_014920.1| Haemophilus influenzae F3031 chromosome, complete genome
contig-100_2275	778	N	0	0	NA	0	0	0	NA	Lactobacillus helveticus CNRZ32, complete genome	gi|525706521|ref|NC_021744.1| Lactobacillus helveticus CNRZ32, complete genome	gi|525706521|ref|NC_021744.1| Lactobacillus helveticus CNRZ32, complete genome

contig-100_2276	777	N	0	0	NA	0	0	0	NA	Candidatus Saccharimonas aalborgensis, complete genome	gi|501435806|ref|NC_021219.1| Candidatus Saccharimonas aalborgensis, complete genome	gi|501435806|ref|NC_021219.1| Candidatus Saccharimonas aalborgensis, complete genome
contig-100_2277	777	N	0	0	NA	0	0	0	NA	NA	NA	NA
contig-100_2278	776	N	0	0	NA	0	0	0	NA	NA	NA	NA
contig-100_2279	776	N	0	0	NA	0	0	0	NA	NA	NA	NA
contig-100_228	3152	N	2	0	NA	0	0	0	NA	NA	NA	NA
contig-100_2280	776	N	0	0	NA	0	0	0	NA	NA	NA	NA
contig-100_2281	775	N	0	0	NA	0	0	0	NA	Haemophilus parainfluenzae T3T1 complete genome	gi|345428590|ref|NC_015964.1| Haemophilus parainfluenzae T3T1, complete genome	gi|345428590|ref|NC_015964.1| Haemophilus parainfluenzae T3T1, complete genome
contig-100_2282	775	N	1	0	NA	0	0	0	NA	NA	NA	NA
contig-100_2283	775	N	0	0	NA	0	0	0	NA	NA	NA	NA
contig-100_2286	774	N	0	0	NA	0	0	0	NA	NA	NA	NA
contig-100_2287	774	N	1	0	NA	0	0	0	NA	Ruminococcus obeum A2-162 draft genome	gi|479176048|ref|NC_021022.1| Ruminococcus obeum A2-162 draft genome	gi|479176048|ref|NC_021022.1| Ruminococcus obeum A2-162 draft genome
contig-100_2289	774	N	0	0	NA	0	0	0	NA	NA	NA	NA
contig-100_2290	774	N	0	0	NA	0	0	0	NA	NA	NA	NA
contig-100_2291	773	N	1	0	NA	0	0	0	NA	Haemophilus parainfluenzae T3T1 complete genome	gi|345428590|ref|NC_015964.1| Haemophilus parainfluenzae T3T1, complete genome	gi|345428590|ref|NC_015964.1| Haemophilus parainfluenzae T3T1, complete genome
contig-100_2292	773	N	0	0	NA	0	0	0	NA	NA	NA	NA

contig-100_2293	773	N	0	0	NA	0	0	0	NA	Bacteroides thetaiotaomicron VPI-5482, complete genome	gi|29345410|ref|NC_004663.1| Bacteroides thetaiotaomicron VPI-5482 chromosome, complete genome	gi|29345410|ref|NC_004663.1| Bacteroides thetaiotaomicron VPI-5482 chromosome, complete genome
contig-100_2294	773	N	0	0	NA	0	0	0	NA	NA	NA	NA
contig-100_2295	773	N	0	0	NA	0	0	0	NA	NA	NA	NA
contig-100_2296	773	N	0	0	NA	0	0	0	NA	NA	NA	NA
contig-100_2297	773	N	0	0	NA	0	0	0	NA	NA	NA	NA
contig-100_2298	772	N	1	0	NA	0	0	0	NA	Azospirillum lipoferum 4B main chromosome, complete genome	gi|374290655|ref|NC_016622.1| Azospirillum lipoferum 4B, complete genome	gi|374290655|ref|NC_016622.1| Azospirillum lipoferum 4B, complete genome
contig-100_2299	772	N	0	0	NA	0	0	0	NA	NA	NA	NA
contig-100_23	12296	N	12	1	NA	0	2	3	NA	Culex pipiens quinquefasciatus, clone Culex pipiens quinquefasciatus-3940115D9, complete sequence	gi|160895450|ref|NC_010002.1| Delftia acidovorans SPH-1 chromosome, complete genome	gi|160895450|ref|NC_010002.1| Delftia acidovorans SPH-1 chromosome, complete genome
contig-100_230	3137	N	1	1	Sipho	0	0	1	NA	NA	NA	NA
contig-100_2300	772	N	1	0	NA	0	0	0	NA	Faecalibacterium prausnitzii SL3/3 draft genome	gi|479170689|ref|NC_021020.1| Faecalibacterium prausnitzii SL3/3 draft genome	gi|479170689|ref|NC_021020.1| Faecalibacterium prausnitzii SL3/3 draft genome
contig-100_2301	771	N	0	0	NA	0	0	0	NA	Bacteroides vulgatus ATCC 8482, complete genome	gi|150002608|ref|NC_009614.1| Bacteroides vulgatus ATCC 8482 chromosome, complete genome	gi|150002608|ref|NC_009614.1| Bacteroides vulgatus ATCC 8482 chromosome, complete genome

contig-100_2302	771	N	0	0	NA	0	0	0	NA	Eubacterium rectale DSM 17629 draft genome	gi|479140210|ref|NC_021010.1| Eubacterium rectale DSM 17629 draft genome	gi|479140210|ref|NC_021010.1| Eubacterium rectale DSM 17629 draft genome
contig-100_2304	771	N	1	1	Sipho	0	0	1	NA	NA	NA	NA
contig-100_2305	771	N	1	0	NA	0	0	0	NA	NA	NA	NA
contig-100_2306	770	N	0	0	NA	0	0	0	NA	NA	NA	NA
contig-100_2307	770	N	0	0	NA	0	0	0	NA	Faecalibacterium prausnitzii L2/6 draft genome	gi|479208076|ref|NC_021042.1| Faecalibacterium prausnitzii L2-6, complete genome	gi|479208076|ref|NC_021042.1| Faecalibacterium prausnitzii L2-6, complete genome
contig-100_2308	770	N	0	0	NA	0	0	0	NA	NA	NA	NA
contig-100_2309	770	N	0	0	NA	0	0	0	NA	NA	NA	NA
contig-100_2310	770	N	0	0	NA	0	0	0	NA	NA	NA	NA
contig-100_2311	770	N	0	0	NA	0	0	0	NA	NA	NA	NA
contig-100_2312	770	N	0	0	NA	0	0	0	NA	NA	NA	NA
contig-100_2314	769	N	0	0	NA	0	0	0	NA	Bacteroides vulgatus ATCC 8482, complete genome	gi|150002608|ref|NC_009614.1| Bacteroides vulgatus ATCC 8482 chromosome, complete genome	gi|150002608|ref|NC_009614.1| Bacteroides vulgatus ATCC 8482 chromosome, complete genome
contig-100_2315	769	N	0	0	NA	0	0	0	NA	Bacteroides vulgatus ATCC 8482, complete genome	gi|150002608|ref|NC_009614.1| Bacteroides vulgatus ATCC 8482 chromosome, complete genome	gi|150002608|ref|NC_009614.1| Bacteroides vulgatus ATCC 8482 chromosome, complete genome
contig-100_2316	768	N	1	0	NA	0	0	0	NA	Clostridiales sp. SSC/2 draft genome	gi|479158859|ref|NC_021016.1| Butyrate-producing bacterium SSC/2, complete genome	gi|479158859|ref|NC_021016.1| Butyrate-producing bacterium SSC/2, complete genome

contig-100_2317	768	N	0	0	NA	0	0	0	NA	Clostridiales sp. SM4/1 draft genome	gi|479208076|ref|NC_021042.1| Faecalibacterium prausnitzii L2-6, complete genome	gi|479208076|ref|NC_021042.1| Faecalibacterium prausnitzii L2-6, complete genome
contig-100_2318	768	N	0	0	NA	0	0	0	NA	NA	NA	NA
contig-100_2319	768	N	2	1	Sipho	0	0	1	NA	NA	NA	NA
contig-100_2320	768	N	0	0	NA	0	0	0	NA	Uncultured organism clone 7 genomic sequence	gi|479176048|ref|NC_021022.1| Ruminococcus obeum A2-162 draft genome	gi|479176048|ref|NC_021022.1| Ruminococcus obeum A2-162 draft genome
contig-100_2322	768	N	0	0	NA	0	0	0	NA	Enterobacteria phage lambda, complete genome	NA	Enterobacteria phage lambda, complete genome
contig-100_2323	768	N	1	0	NA	0	0	0	NA	NA	NA	NA
contig-100_2324	767	N	0	0	NA	0	0	0	NA	Bacteroides thetaiotaomicron VPI-5482, complete genome	gi|29345410|ref|NC_004663.1| Bacteroides thetaiotaomicron VPI-5482 chromosome, complete genome	gi|29345410|ref|NC_004663.1| Bacteroides thetaiotaomicron VPI-5482 chromosome, complete genome
contig-100_2325	767	N	1	0	NA	0	0	0	NA	NA	NA	NA
contig-100_2326	767	N	1	0	NA	0	0	0	NA	NA	NA	NA
contig-100_2327	767	N	0	0	NA	0	0	0	NA	NA	NA	NA
contig-100_2328	767	N	0	0	NA	0	0	0	NA	NA	NA	NA
contig-100_2329	766	N	0	0	NA	0	0	0	NA	Bacteroides vulgatus ATCC 8482, complete genome	gi|150002608|ref|NC_009614.1| Bacteroides vulgatus ATCC 8482 chromosome, complete genome	gi|150002608|ref|NC_009614.1| Bacteroides vulgatus ATCC 8482 chromosome, complete genome
contig-100_233	3123	N	2	1	Sipho	1	0	1	NA	NA	NA	NA

contig-100_2330	766	N	0	0	NA	0	0	0	NA	Uncultured bacterium clone LM0ACA22ZE03FM1 genomic sequence	NA	Uncultured bacterium clone LM0ACA22ZE03FM1 genomic sequence
contig-100_2331	766	N	0	0	NA	0	0	0	NA	NA	NA	NA
contig-100_2333	766	N	0	0	NA	0	0	0	NA	NA	NA	NA
contig-100_2334	766	N	0	0	NA	0	0	0	NA	Haemophilus parainfluenzae T3T1 complete genome	gi|345428590|ref|NC_015964.1| Haemophilus parainfluenzae T3T1, complete genome	gi|345428590|ref|NC_015964.1| Haemophilus parainfluenzae T3T1, complete genome
contig-100_2335	765	N	1	0	NA	0	0	0	NA	Faecalibacterium prausnitzii L2/6 draft genome	gi|479208076|ref|NC_021042.1| Faecalibacterium prausnitzii L2-6, complete genome	gi|479208076|ref|NC_021042.1| Faecalibacterium prausnitzii L2-6, complete genome
contig-100_2336	765	N	0	0	NA	0	0	0	NA	NA	NA	NA
contig-100_2337	765	N	1	0	NA	0	0	0	NA	NA	NA	NA
contig-100_2338	764	N	0	0	NA	0	0	0	NA	NA	NA	NA
contig-100_2339	764	N	1	0	NA	0	0	0	NA	NA	NA	NA
contig-100_234	3118	N	4	1	Herpes	0	0	2	NA	Neisseria gonorrhoeae 5.2 kb beta-lactamase plasmid pSJ5.2, complete sequence	NA	Neisseria gonorrhoeae 5.2 kb beta-lactamase plasmid pSJ5.2, complete sequence
contig-100_2342	764	N	0	0	NA	0	0	0	NA	NA	NA	NA
contig-100_2343	764	N	0	0	NA	0	0	0	NA	NA	NA	NA
contig-100_2344	763	N	0	0	NA	0	0	0	NA	NA	NA	NA
contig-100_2345	763	N	1	0	NA	0	0	0	NA	NA	NA	NA
contig-100_2346	763	N	0	0	NA	0	0	0	NA	NA	NA	NA
contig-100_2347	763	N	0	0	NA	0	0	0	NA	NA	NA	NA

contig-100_2348	762	N	0	0	NA	0	0	0	NA	Haemophilus parainfluenzae T3T1 complete genome	gi|345428590|ref|NC_015964.1| Haemophilus parainfluenzae T3T1, complete genome	gi|345428590|ref|NC_015964.1| Haemophilus parainfluenzae T3T1, complete genome
contig-100_2349	762	N	1	0	NA	0	0	0	NA	NA	NA	NA
contig-100_2350	762	N	0	0	NA	0	0	0	NA	NA	NA	NA
contig-100_2351	762	N	0	0	NA	0	0	0	NA	Faecalibacterium prausnitzii L2/6 draft genome	gi|479208076|ref|NC_021042.1| Faecalibacterium prausnitzii L2-6, complete genome	gi|479208076|ref|NC_021042.1| Faecalibacterium prausnitzii L2-6, complete genome
contig-100_2352	762	N	0	0	NA	0	0	0	NA	NA	NA	NA
contig-100_2353	762	N	0	0	NA	0	0	0	NA	Faecalibacterium prausnitzii SL3/3 draft genome	gi|479170689|ref|NC_021020.1| Faecalibacterium prausnitzii SL3/3 draft genome	gi|479170689|ref|NC_021020.1| Faecalibacterium prausnitzii SL3/3 draft genome
contig-100_2354	762	N	0	0	NA	0	0	0	NA	NA	NA	NA
contig-100_2355	761	N	0	0	NA	0	0	0	NA	Faecalibacterium prausnitzii SL3/3 draft genome	gi|479170689|ref|NC_021020.1| Faecalibacterium prausnitzii SL3/3 draft genome	gi|479170689|ref|NC_021020.1| Faecalibacterium prausnitzii SL3/3 draft genome
contig-100_2356	760	N	0	0	NA	0	0	0	NA	NA	NA	NA
contig-100_2357	760	N	2	0	NA	0	0	0	NA	NA	NA	NA
contig-100_2358	760	N	0	0	NA	0	0	0	NA	NA	NA	NA
contig-100_2359	760	N	1	0	NA	0	0	0	NA	NA	NA	NA
contig-100_2360	759	N	1	0	NA	0	0	0	NA	NA	NA	NA
contig-100_2361	759	N	0	0	NA	0	0	0	NA	NA	NA	NA
contig-100_2362	759	N	1	0	NA	0	0	0	NA	NA	NA	NA
contig-100_2363	759	N	0	0	NA	0	0	0	NA	NA	NA	NA
contig-100_2364	759	N	0	0	NA	0	0	0	NA	NA	NA	NA
contig-100_2365	759	N	1	0	NA	0	0	0	NA	NA	NA	NA

contig-100_2366	759	N	0	0	NA	0	0	0	NA	Bacteroides uniformis strain WH207 transposon CTnBST, complete sequence	gi|328946930|ref|NC_015385.1| Treponema succinifaciens DSM 2489 chromosome, complete genome	gi|328946930|ref|NC_015385.1| Treponema succinifaciens DSM 2489 chromosome, complete genome
contig-100_2367	758	N	0	0	NA	0	0	0	NA	NA	NA	NA
contig-100_2368	758	N	0	0	NA	0	0	0	NA	Clostridium saccharolyticum-like K10 draft genome	gi|479336697|ref|NC_021047.1| Clostridium cf. saccharolyticum K10, complete genome	gi|479336697|ref|NC_021047.1| Clostridium cf. saccharolyticum K10, complete genome
contig-100_2369	758	N	0	0	NA	0	0	0	NA	Parabacteroides distasonis ATCC 8503, complete genome	gi|150006674|ref|NC_009615.1| Parabacteroides distasonis ATCC 8503 chromosome, complete genome	gi|150006674|ref|NC_009615.1| Parabacteroides distasonis ATCC 8503 chromosome, complete genome
contig-100_2371	758	N	2	0	NA	0	0	0	NA	NA	NA	NA
contig-100_2373	758	N	0	0	NA	0	0	0	NA	Clostridiales sp. SM4/1 draft genome	gi|479181986|ref|NC_021024.1| Butyrate-producing bacterium SM4/1, complete genome	gi|479181986|ref|NC_021024.1| Butyrate-producing bacterium SM4/1, complete genome
contig-100_2374	758	N	0	0	NA	0	0	0	NA	NA	NA	NA
contig-100_2375	757	N	0	0	NA	0	0	0	NA	Escherichia coli str. K-12 substr. MG1655, complete genome	gi|386612163|ref|NC_017641.1| Escherichia coli UMNK88 chromosome, complete genome	gi|386612163|ref|NC_017641.1| Escherichia coli UMNK88 chromosome, complete genome

contig-100_2376	757	N	0	0	NA	0	0	0	NA	Lactobacillus casei LOCK919, complete genome	gi|523512490|ref|NC_021721.1| Lactobacillus casei LOCK919, complete genome	gi|523512490|ref|NC_021721.1| Lactobacillus casei LOCK919, complete genome
contig-100_2377	757	N	1	0	NA	0	0	0	NA	NA	NA	NA
contig-100_2378	757	N	1	0	NA	0	0	0	NA	NA	NA	NA
contig-100_2379	757	N	0	0	NA	0	0	0	NA	NA	NA	NA
contig-100_2381	756	N	1	0	NA	0	0	0	NA	NA	NA	NA
contig-100_2382	756	N	0	0	NA	0	0	0	NA	NA	NA	NA
contig-100_2383	756	N	0	0	NA	0	0	0	NA	NA	NA	NA
contig-100_2384	755	N	0	0	NA	0	0	0	NA	NA	NA	NA
contig-100_2385	755	N	0	0	NA	0	0	0	NA	Bacteroides salanitronis DSM 18170, complete genome	gi|325297172|ref|NC_015164.1| Bacteroides salanitronis DSM 18170 chromosome, complete genome	gi|325297172|ref|NC_015164.1| Bacteroides salanitronis DSM 18170 chromosome, complete genome
contig-100_2386	754	N	1	0	NA	0	0	0	NA	Eubacterium siraeum V10Sc8a draft genome	gi|479210985|ref|NC_021043.1| Eubacterium siraeum V10Sc8a draft genome	gi|479210985|ref|NC_021043.1| Eubacterium siraeum V10Sc8a draft genome
contig-100_2387	754	N	0	0	NA	0	0	0	NA	Faecalibacterium prausnitzii L2/6 draft genome	gi|479208076|ref|NC_021042.1| Faecalibacterium prausnitzii L2-6, complete genome	gi|479208076|ref|NC_021042.1| Faecalibacterium prausnitzii L2-6, complete genome
contig-100_2388	754	N	0	0	NA	0	0	0	NA	NA	NA	NA
contig-100_2389	754	N	0	0	NA	0	0	0	NA	Bacteroides thetaiotaomicron VPI-5482, complete genome	gi|29345410|ref|NC_004663.1| Bacteroides thetaiotaomicron VPI-5482 chromosome, complete genome	gi|29345410|ref|NC_004663.1| Bacteroides thetaiotaomicron VPI-5482 chromosome, complete genome
contig-100_2390	753	N	0	0	NA	0	0	0	NA	NA	NA	NA

contig-100_2391	753	N	0	0	NA	0	0	0	NA	NA	NA	NA
contig-100_2392	753	N	2	0	NA	0	0	0	NA	Eubacterium cylindroides T2-87 draft genome	gi|479169109|ref|NC_021019.1| Eubacterium cylindroides T2-87 draft genome	gi|479169109|ref|NC_021019.1| Eubacterium cylindroides T2-87 draft genome
contig-100_2393	753	N	0	0	NA	0	0	0	NA	NA	NA	NA
contig-100_2394	753	N	0	0	NA	0	0	0	NA	NA	NA	NA
contig-100_2395	753	N	1	0	NA	0	0	0	NA	Haemophilus parainfluenzae T3T1 complete genome	gi|345428590|ref|NC_015964.1| Haemophilus parainfluenzae T3T1, complete genome	gi|345428590|ref|NC_015964.1| Haemophilus parainfluenzae T3T1, complete genome
contig-100_2396	753	N	1	0	NA	0	0	0	NA	NA	NA	NA
contig-100_2397	752	N	0	0	NA	0	0	0	NA	NA	NA	NA
contig-100_2398	752	N	0	0	NA	0	0	0	NA	NA	NA	NA
contig-100_2399	752	N	0	0	NA	0	0	0	NA	NA	NA	NA
contig-100_2400	752	N	0	0	NA	0	0	0	NA	Roseburia hominis A2-183, complete genome	gi|347530298|ref|NC_015977.1| Roseburia hominis A2-183 chromosome, complete genome	gi|347530298|ref|NC_015977.1| Roseburia hominis A2-183 chromosome, complete genome
contig-100_2401	752	N	0	0	NA	0	0	0	NA	NA	NA	NA
contig-100_2402	751	N	0	0	NA	0	0	0	NA	Haemophilus parainfluenzae T3T1 complete genome	gi|345428590|ref|NC_015964.1| Haemophilus parainfluenzae T3T1, complete genome	gi|345428590|ref|NC_015964.1| Haemophilus parainfluenzae T3T1, complete genome
contig-100_2404	751	N	0	0	NA	0	0	0	NA	NA	NA	NA
contig-100_2405	751	N	1	0	NA	0	0	0	NA	NA	NA	NA
contig-100_2406	751	N	1	0	NA	0	0	0	NA	NA	NA	NA

contig-100_2407	751	N	0	0	NA	0	0	0	NA	Haemophilus parainfluenzae T3T1 complete genome	gi|345428590|ref|NC_015964.1| Haemophilus parainfluenzae T3T1, complete genome	gi|345428590|ref|NC_015964.1| Haemophilus parainfluenzae T3T1, complete genome
contig-100_2408	751	N	0	0	NA	0	0	0	NA	NA	NA	NA
contig-100_2409	750	N	0	0	NA	0	0	0	NA	Faecalibacterium prausnitzii L2/6 draft genome	gi|479208076|ref|NC_021042.1| Faecalibacterium prausnitzii L2-6, complete genome	gi|479208076|ref|NC_021042.1| Faecalibacterium prausnitzii L2-6, complete genome
contig-100_241	3050	N	3	1	Sipho	0	0	2	NA	Bacteroides salanitronis DSM 18170, complete genome	gi|325297172|ref|NC_015164.1| Bacteroides salanitronis DSM 18170 chromosome, complete genome	gi|325297172|ref|NC_015164.1| Bacteroides salanitronis DSM 18170 chromosome, complete genome
contig-100_2411	749	N	0	0	NA	0	0	0	NA	Uncultured organism clone 1041059765996 genomic sequence	gi|150002608|ref|NC_009614.1| Bacteroides vulgatus ATCC 8482 chromosome, complete genome	gi|150002608|ref|NC_009614.1| Bacteroides vulgatus ATCC 8482 chromosome, complete genome
contig-100_2413	749	N	0	0	NA	0	0	0	NA	NA	NA	NA
contig-100_2414	749	N	0	0	NA	0	0	0	NA	NA	NA	NA
contig-100_2415	749	N	1	0	NA	0	0	0	NA	Bacteroides vulgatus ATCC 8482, complete genome	gi|150002608|ref|NC_009614.1| Bacteroides vulgatus ATCC 8482 chromosome, complete genome	gi|150002608|ref|NC_009614.1| Bacteroides vulgatus ATCC 8482 chromosome, complete genome
contig-100_2416	749	N	0	0	NA	0	0	0	NA	NA	NA	NA
contig-100_2417	749	N	0	0	NA	0	0	0	NA	NA	NA	NA
contig-100_2418	748	N	0	0	NA	0	0	0	NA	NA	NA	NA
contig-100_2419	748	N	0	0	NA	0	0	0	NA	NA	NA	NA

contig-100_242	3047	N	2	1	Sipho	1	0	1	NA	NA	NA	NA
contig-100_2420	748	N	1	0	NA	0	0	0	NA	NA	NA	NA
contig-100_2421	748	N	0	0	NA	0	0	0	NA	NA	NA	NA
contig-100_2423	747	N	0	0	NA	0	0	0	NA	NA	NA	NA
contig-100_2424	747	N	0	0	NA	0	0	0	NA	Eubacterium eligens ATCC 27750, complete genome	gi|238915976|ref|NC_012778.1| Eubacterium eligens ATCC 27750 chromosome, complete genome	gi|238915976|ref|NC_012778.1| Eubacterium eligens ATCC 27750 chromosome, complete genome
contig-100_2425	747	N	0	0	NA	0	0	0	NA	NA	NA	NA
contig-100_2426	747	N	0	0	NA	0	0	0	NA	NA	NA	NA
contig-100_2427	747	N	0	0	NA	0	0	0	NA	NA	NA	NA
contig-100_2428	746	N	0	0	NA	0	0	0	NA	NA	NA	NA
contig-100_2429	746	N	1	0	NA	0	0	0	NA	Lactobacillus bacteriophage phage AQ113, complete genome	gi|525706521|ref|NC_021744.1| Lactobacillus helveticus CNRZ32, complete genome	gi|525706521|ref|NC_021744.1| Lactobacillus helveticus CNRZ32, complete genome
contig-100_243	3040	N	2	0	NA	0	0	0	NA	NA	NA	NA
contig-100_2431	745	N	0	0	NA	0	0	0	NA	NA	NA	NA
contig-100_2432	745	N	0	0	NA	0	0	0	NA	NA	NA	NA
contig-100_2433	744	N	0	0	NA	0	0	0	NA	NA	NA	NA
contig-100_2434	744	N	0	0	NA	0	0	0	NA	NA	NA	NA
contig-100_2435	744	N	0	0	NA	0	0	0	NA	Bacteroides vulgatus ATCC 8482, complete genome	gi|150002608|ref|NC_009614.1| Bacteroides vulgatus ATCC 8482 chromosome, complete genome	gi|150002608|ref|NC_009614.1| Bacteroides vulgatus ATCC 8482 chromosome, complete genome
contig-100_2436	744	N	0	0	NA	0	0	0	NA	Bacteroides vulgatus ATCC 8482, complete genome	gi|150002608|ref|NC_009614.1| Bacteroides vulgatus ATCC 8482 chromosome, complete genome	gi|150002608|ref|NC_009614.1| Bacteroides vulgatus ATCC 8482 chromosome, complete genome

contig-100_2437	744	N	1	0	NA	0	0	0	NA	Streptococcus parauberis KCTC 11537, complete genome	gi|333904249|ref|NC_015558.1| Streptococcus parauberis KCTC 11537 chromosome, complete genome	gi|333904249|ref|NC_015558.1| Streptococcus parauberis KCTC 11537 chromosome, complete genome
contig-100_2438	744	N	1	0	NA	0	0	0	NA	Uncultured organism clone 1041059765231 genomic sequence	NA	Uncultured organism clone 1041059765231 genomic sequence
contig-100_2439	743	N	0	0	NA	0	0	0	NA	NA	NA	NA
contig-100_2440	743	N	0	0	NA	0	0	0	NA	NA	NA	NA
contig-100_2442	743	N	0	0	NA	0	0	0	NA	NA	NA	NA
contig-100_2444	742	N	0	0	NA	0	0	0	NA	NA	NA	NA
contig-100_2445	742	N	0	0	NA	0	0	0	NA	NA	NA	NA
contig-100_2446	742	N	0	0	NA	0	0	0	NA	NA	NA	NA
contig-100_2448	741	N	0	0	NA	0	0	0	NA	NA	NA	NA
contig-100_2449	741	N	0	0	NA	0	0	0	NA	Clostridiales sp. SM4/1 draft genome	gi|479181986|ref|NC_021024.1| Butyrate-producing bacterium SM4/1, complete genome	gi|479181986|ref|NC_021024.1| Butyrate-producing bacterium SM4/1, complete genome
contig-100_245	3020	N	2	0	NA	0	0	0	NA	Klebsiella pneumoniae subsp. pneumoniae HS11286, complete genome	gi|550443072|ref|NC_022566.1| Klebsiella pneumoniae CG43, complete genome	gi|550443072|ref|NC_022566.1| Klebsiella pneumoniae CG43, complete genome
contig-100_2450	741	N	0	0	NA	0	0	0	NA	NA	NA	NA
contig-100_2451	741	N	0	0	NA	0	0	0	NA	NA	NA	NA
contig-100_2453	741	N	1	0	NA	0	0	0	NA	NA	NA	NA
contig-100_2454	741	N	0	0	NA	0	0	0	NA	NA	NA	NA
contig-100_2455	740	N	0	0	NA	0	0	0	NA	Streptococcus thermophilus JIM 8232 complete genome	gi|386343608|ref|NC_017581.1| Streptococcus thermophilus JIM 8232, complete genome	gi|386343608|ref|NC_017581.1| Streptococcus thermophilus JIM 8232, complete genome

contig-100_2457	739	N	0	0	NA	0	0	0	NA	NA	NA	NA
contig-100_2458	739	N	0	0	NA	0	0	0	NA	NA	NA	NA
contig-100_2459	738	N	0	0	NA	0	0	0	NA	NA	NA	NA
contig-100_2461	738	N	0	0	NA	0	0	0	NA	NA	NA	NA
contig-100_2462	738	N	0	0	NA	0	0	0	NA	NA	NA	NA
contig-100_2463	737	N	0	0	NA	0	0	0	NA	NA	NA	NA
contig-100_2464	737	N	1	0	NA	0	0	0	NA	NA	NA	NA
contig-100_2465	737	N	0	0	NA	0	0	0	NA	Streptococcus thermophilus ND03, complete genome	gi|386085705|ref|NC_017563.1| Streptococcus thermophilus ND03 chromosome, complete genome	gi|386085705|ref|NC_017563.1| Streptococcus thermophilus ND03 chromosome, complete genome
contig-100_2467	737	N	1	0	NA	0	0	0	NA	Bacteroides vulgatus ATCC 8482, complete genome	gi|150002608|ref|NC_009614.1| Bacteroides vulgatus ATCC 8482 chromosome, complete genome	gi|150002608|ref|NC_009614.1| Bacteroides vulgatus ATCC 8482 chromosome, complete genome
contig-100_2469	736	N	1	0	NA	0	0	0	NA	NA	NA	NA
contig-100_2470	736	N	0	0	NA	0	0	0	NA	NA	NA	NA
contig-100_2471	736	N	0	0	NA	0	0	0	NA	NA	NA	NA
contig-100_2473	736	N	0	0	NA	0	0	0	NA	NA	NA	NA
contig-100_2474	736	N	0	0	NA	0	0	0	NA	NA	NA	NA
contig-100_2475	736	N	0	0	NA	0	0	0	NA	Macrococcus caseolyticus JCSC5402 DNA, complete genome	gi|222150250|ref|NC_011999.1| Macrococcus caseolyticus JCSC5402, complete genome	gi|222150250|ref|NC_011999.1| Macrococcus caseolyticus JCSC5402, complete genome
contig-100_2476	736	N	0	0	NA	0	0	0	NA	Bacteroides vulgatus ATCC 8482, complete genome	gi|150002608|ref|NC_009614.1| Bacteroides vulgatus ATCC 8482 chromosome, complete genome	gi|150002608|ref|NC_009614.1| Bacteroides vulgatus ATCC 8482 chromosome, complete genome

contig-100_2477	735	N	0	0	NA	0	0	0	NA	Ruminococcus bromii L2-63 draft genome	gi|479150083|ref|NC_021013.1| Ruminococcus bromii L2-63 draft genome	gi|479150083|ref|NC_021013.1| Ruminococcus bromii L2-63 draft genome
contig-100_2478	735	N	0	0	NA	0	0	0	NA	NA	NA	NA
contig-100_2479	735	N	0	0	NA	0	0	0	NA	NA	NA	NA
contig-100_248	2985	N	3	1	Sipho	1	0	1	NA	NA	NA	NA
contig-100_2480	735	N	1	0	NA	0	0	0	NA	NA	NA	NA
contig-100_2481	735	N	0	0	NA	0	0	0	NA	NA	NA	NA
contig-100_2482	734	N	0	0	NA	0	0	0	NA	NA	NA	NA
contig-100_2483	734	N	0	0	NA	0	0	0	NA	Uncultured organism clone 1041059765169 genomic sequence	NA	Uncultured organism clone 1041059765169 genomic sequence
contig-100_2484	734	N	0	0	NA	0	0	0	NA	Faecalibacterium prausnitzii SL3/3 draft genome	gi|479170689|ref|NC_021020.1| Faecalibacterium prausnitzii SL3/3 draft genome	gi|479170689|ref|NC_021020.1| Faecalibacterium prausnitzii SL3/3 draft genome
contig-100_2485	733	N	0	0	NA	0	0	0	NA	Faecalibacterium prausnitzii L2/6 draft genome	gi|479208076|ref|NC_021042.1| Faecalibacterium prausnitzii L2-6, complete genome	gi|479208076|ref|NC_021042.1| Faecalibacterium prausnitzii L2-6, complete genome
contig-100_2486	733	N	0	0	NA	0	0	0	NA	NA	NA	NA
contig-100_2487	733	N	0	0	NA	0	0	0	NA	NA	NA	NA
contig-100_2488	732	N	1	0	NA	0	0	0	NA	Uncultured organism clone 1041059766135 genomic sequence	gi|319899888|ref|NC_014933.1| Bacteroides helcogenes P 36-108 chromosome, complete genome	gi|319899888|ref|NC_014933.1| Bacteroides helcogenes P 36-108 chromosome, complete genome
contig-100_2489	732	N	0	0	NA	0	0	0	NA	NA	NA	NA
contig-100_249	2977	N	0	0	NA	0	0	0	NA	NA	NA	NA
contig-100_2490	732	N	0	0	NA	0	0	0	NA	NA	NA	NA
contig-100_2491	732	N	0	0	NA	0	0	0	NA	NA	NA	NA

contig-100_2492	732	N	0	0	NA	0	0	0	NA	NA	NA	NA
contig-100_2493	731	N	0	0	NA	0	0	0	NA	Eubacterium rectale DSM 17629 draft genome	gi|479140210|ref|NC_021010.1| Eubacterium rectale DSM 17629 draft genome	gi|479140210|ref|NC_021010.1| Eubacterium rectale DSM 17629 draft genome
contig-100_2494	731	N	0	0	NA	0	0	0	NA	NA	NA	NA
contig-100_2495	731	N	0	0	NA	0	0	0	NA	NA	NA	NA
contig-100_2497	730	N	0	0	NA	0	0	0	NA	NA	NA	NA
contig-100_2498	730	N	1	0	NA	0	0	0	NA	Faecalibacterium prausnitzii SL3/3 draft genome	gi|479170689|ref|NC_021020.1| Faecalibacterium prausnitzii SL3/3 draft genome	gi|479170689|ref|NC_021020.1| Faecalibacterium prausnitzii SL3/3 draft genome
contig-100_2499	730	N	0	0	NA	0	0	0	NA	Bacteroides thetaiotaomicron VPI-5482, complete genome	gi|29345410|ref|NC_004663.1| Bacteroides thetaiotaomicron VPI-5482 chromosome, complete genome	gi|29345410|ref|NC_004663.1| Bacteroides thetaiotaomicron VPI-5482 chromosome, complete genome
contig-100_25	12029	N	10	0	NA	0	0	0	NA	NA	NA	NA
contig-100_250	2971	N	3	3	Myo	0	0	2	NA	NA	NA	NA
contig-100_2500	730	N	0	0	NA	0	0	0	NA	NA	NA	NA
contig-100_2501	730	N	0	0	NA	0	0	0	NA	Faecalibacterium prausnitzii L2/6 draft genome	gi|479208076|ref|NC_021042.1| Faecalibacterium prausnitzii L2-6, complete genome	gi|479208076|ref|NC_021042.1| Faecalibacterium prausnitzii L2-6, complete genome
contig-100_2502	729	N	0	0	NA	0	0	0	NA	Uncultured bacterium clone HA0AAA5ZC10RM1 genomic sequence	NA	Uncultured bacterium clone HA0AAA5ZC10RM1 genomic sequence
contig-100_2504	729	N	0	0	NA	0	0	0	NA	NA	NA	NA

contig-100_2505	729	N	1	0	NA	0	0	0	NA	Eubacterium rectale M104/1 draft genome	gi|479213596|ref|NC_021044.1| Eubacterium rectale M104/1 draft genome	gi|479213596|ref|NC_021044.1| Eubacterium rectale M104/1 draft genome
contig-100_2506	729	N	1	0	NA	0	0	0	NA	NA	NA	NA
contig-100_2507	729	N	0	0	NA	0	0	0	NA	NA	NA	NA
contig-100_2508	729	N	0	0	NA	0	0	0	NA	NA	NA	NA
contig-100_2509	728	N	0	0	NA	0	0	0	NA	NA	NA	NA
contig-100_251	2962	N	3	1	Sipho	1	1	1	NA	NA	NA	NA
contig-100_2511	728	N	0	0	NA	0	0	0	NA	NA	NA	NA
contig-100_2512	728	N	0	0	NA	0	0	0	NA	NA	NA	NA
contig-100_2514	727	N	0	0	NA	0	0	0	NA	NA	NA	NA
contig-100_2515	727	N	0	0	NA	0	0	0	NA	NA	NA	NA
contig-100_2516	727	N	1	0	NA	0	0	0	NA	NA	NA	NA
contig-100_2517	726	N	0	0	NA	0	0	0	NA	NA	NA	NA
contig-100_2518	726	N	0	0	NA	0	0	0	NA	Alteromonas macleodii str. 'Ionian Sea UM4b', complete genome	gi|386311792|ref|NC_017566.1| Shewanella putrefaciens 200 chromosome, complete genome	gi|386311792|ref|NC_017566.1| Shewanella putrefaciens 200 chromosome, complete genome
contig-100_2519	726	N	0	0	NA	0	0	0	NA	NA	NA	NA
contig-100_252	2958	N	5	0	NA	0	0	1	NA	Unidentified phage clone 2019_scaffold132 genomic sequence	NA	Unidentified phage clone 2019_scaffold132 genomic sequence
contig-100_2520	726	N	0	0	NA	0	0	0	NA	Streptococcus phage Abc2, complete genome	gi|387783149|ref|NC_017595.1| Streptococcus salivarius JIM8777, complete genome	gi|387783149|ref|NC_017595.1| Streptococcus salivarius JIM8777, complete genome
contig-100_2521	725	N	1	0	NA	0	0	0	NA	NA	NA	NA
contig-100_2522	725	N	0	0	NA	0	0	0	NA	NA	NA	NA

contig-100_2524	725	N	0	0	NA	0	0	0	NA	Bacteroides xylanisolvens XB1A draft genome	gi|479162165|ref|NC_021017.1| Bacteroides xylanisolvens XB1A draft genome	gi|479162165|ref|NC_021017.1| Bacteroides xylanisolvens XB1A draft genome
contig-100_2525	725	N	0	0	NA	0	0	0	NA	Haemophilus parainfluenzae T3T1 complete genome	gi|345428590|ref|NC_015964.1| Haemophilus parainfluenzae T3T1, complete genome	gi|345428590|ref|NC_015964.1| Haemophilus parainfluenzae T3T1, complete genome
contig-100_2526	724	N	0	0	NA	0	0	0	NA	Faecalibacterium prausnitzii L2/6 draft genome	gi|479208076|ref|NC_021042.1| Faecalibacterium prausnitzii L2-6, complete genome	gi|479208076|ref|NC_021042.1| Faecalibacterium prausnitzii L2-6, complete genome
contig-100_2527	724	N	0	0	NA	0	0	0	NA	Faecalibacterium prausnitzii L2/6 draft genome	gi|479208076|ref|NC_021042.1| Faecalibacterium prausnitzii L2-6, complete genome	gi|479208076|ref|NC_021042.1| Faecalibacterium prausnitzii L2-6, complete genome
contig-100_2528	724	N	0	0	NA	0	0	0	NA	NA	NA	NA
contig-100_2529	724	N	0	0	NA	0	0	0	NA	Haemophilus parainfluenzae T3T1 complete genome	gi|345428590|ref|NC_015964.1| Haemophilus parainfluenzae T3T1, complete genome	gi|345428590|ref|NC_015964.1| Haemophilus parainfluenzae T3T1, complete genome
contig-100_253	2958	N	3	0	NA	0	0	0	NA	NA	NA	NA
contig-100_2530	724	N	0	0	NA	0	0	0	NA	Uncultured organism clone 1041059765897 genomic sequence	NA	Uncultured organism clone 1041059765897 genomic sequence
contig-100_2531	723	N	0	0	NA	0	0	0	NA	NA	NA	NA

contig-100_2532	723	N	0	0	NA	0	0	0	NA	Uncultured organism clone 1041059766418 genomic sequence	NA	Uncultured organism clone 1041059766418 genomic sequence
contig-100_2533	723	N	0	0	NA	0	0	0	NA	Uncultured organism clone 1041059767481 genomic sequence	NA	Uncultured organism clone 1041059767481 genomic sequence
contig-100_2534	723	N	0	0	NA	0	0	0	NA	NA	NA	NA
contig-100_2535	723	N	1	0	NA	0	0	0	NA	Faecalibacterium prausnitzii L2/6 draft genome	gi|479208076|ref|NC_021042.1| Faecalibacterium prausnitzii L2-6, complete genome	gi|479208076|ref|NC_021042.1| Faecalibacterium prausnitzii L2-6, complete genome
contig-100_2536	723	N	0	0	NA	0	0	0	NA	Acidovorax sp. KKS102, complete genome	gi|407936729|ref|NC_018708.1| Acidovorax sp. KKS102 chromosome, complete genome	gi|407936729|ref|NC_018708.1| Acidovorax sp. KKS102 chromosome, complete genome
contig-100_2537	722	N	0	0	NA	0	0	0	NA	Alistipes shahii WAL 8301 draft genome	gi|479185170|ref|NC_021030.1| Alistipes shahii WAL 8301 draft genome	gi|479185170|ref|NC_021030.1| Alistipes shahii WAL 8301 draft genome
contig-100_2538	722	N	0	0	NA	0	0	0	NA	Faecalibacterium prausnitzii L2/6 draft genome	gi|479208076|ref|NC_021042.1| Faecalibacterium prausnitzii L2-6, complete genome	gi|479208076|ref|NC_021042.1| Faecalibacterium prausnitzii L2-6, complete genome
contig-100_254	2939	N	1	0	NA	0	0	0	NA	NA	NA	NA
contig-100_2540	722	N	1	0	NA	0	0	0	NA	NA	NA	NA
contig-100_2541	721	N	0	0	NA	0	0	0	NA	Uncultured organism clone 1041059767067 genomic sequence	NA	Uncultured organism clone 1041059767067 genomic sequence

contig-100_2542	720	N	0	0	NA	0	0	0	NA	NA	NA	NA
contig-100_2543	720	N	0	0	NA	0	0	0	NA	NA	NA	NA
contig-100_2546	720	N	0	0	NA	0	0	0	NA	NA	NA	NA
contig-100_2547	720	N	0	0	NA	0	0	0	NA	Faecalibacterium prausnitzii L2/6 draft genome	gi|479208076|ref|NC_021042.1| Faecalibacterium prausnitzii L2-6, complete genome	gi|479208076|ref|NC_021042.1| Faecalibacterium prausnitzii L2-6, complete genome
contig-100_2548	720	N	0	0	NA	0	0	0	NA	Bacteroides vulgatus ATCC 8482, complete genome	gi|150002608|ref|NC_009614.1| Bacteroides vulgatus ATCC 8482 chromosome, complete genome	gi|150002608|ref|NC_009614.1| Bacteroides vulgatus ATCC 8482 chromosome, complete genome
contig-100_2549	719	N	0	0	NA	0	0	0	NA	NA	NA	NA
contig-100_255	2937	N	3	0	NA	0	0	0	NA	Faecalibacterium prausnitzii L2/6 draft genome	gi|479208076|ref|NC_021042.1| Faecalibacterium prausnitzii L2-6, complete genome	gi|479208076|ref|NC_021042.1| Faecalibacterium prausnitzii L2-6, complete genome
contig-100_2552	719	N	1	0	NA	0	0	0	NA	NA	NA	NA
contig-100_2553	719	N	0	0	NA	0	0	0	NA	NA	NA	NA
contig-100_2554	719	N	0	0	NA	0	0	0	NA	NA	NA	NA
contig-100_2555	719	N	1	0	NA	0	0	0	NA	NA	NA	NA
contig-100_2556	719	N	0	0	NA	0	0	0	NA	NA	NA	NA
contig-100_2557	718	N	0	0	NA	0	0	0	NA	NA	NA	NA
contig-100_2558	718	N	0	0	NA	0	0	0	NA	NA	NA	NA
contig-100_2559	718	N	0	0	NA	0	0	0	NA	NA	NA	NA
contig-100_256	2933	N	4	0	NA	0	0	0	NA	NA	NA	NA
contig-100_2560	718	N	0	0	NA	0	0	0	NA	NA	NA	NA
contig-100_2561	718	N	0	0	NA	0	0	0	NA	NA	NA	NA
contig-100_2562	718	N	0	0	NA	0	0	0	NA	Haemophilus parainfluenzae T3T1 complete genome	gi|345428590|ref|NC_015964.1| Haemophilus parainfluenzae T3T1, complete genome	gi|345428590|ref|NC_015964.1| Haemophilus parainfluenzae T3T1, complete genome

contig-100_2563	718	N	1	0	NA	0	0	0	NA	Bacteroides thetaiotaomicron VPI-5482, complete genome	gi|29345410|ref|NC_004663.1| Bacteroides thetaiotaomicron VPI-5482 chromosome, complete genome	gi|29345410|ref|NC_004663.1| Bacteroides thetaiotaomicron VPI-5482 chromosome, complete genome
contig-100_2564	717	N	0	0	NA	0	0	0	NA	NA	NA	NA
contig-100_2565	717	N	1	0	NA	0	0	0	NA	Uncultured bacterium clone LM0ACA22ZB10RM1 genomic sequence	gi|34539880|ref|NC_002950.2| Porphyromonas gingivalis W83 chromosome, complete genome	gi|34539880|ref|NC_002950.2| Porphyromonas gingivalis W83 chromosome, complete genome
contig-100_2567	717	N	0	0	NA	0	0	0	NA	NA	NA	NA
contig-100_2568	717	N	0	0	NA	0	0	0	NA	NA	NA	NA
contig-100_2569	717	N	0	0	NA	0	0	0	NA	Bacteroides vulgatus ATCC 8482, complete genome	gi|150002608|ref|NC_009614.1| Bacteroides vulgatus ATCC 8482 chromosome, complete genome	gi|150002608|ref|NC_009614.1| Bacteroides vulgatus ATCC 8482 chromosome, complete genome
contig-100_257	2923	N	2	1	Myo	0	0	1	NA	NA	NA	NA
contig-100_2570	716	N	0	0	NA	0	0	0	NA	Uncultured organism clone 1041059765229 genomic sequence	NA	Uncultured organism clone 1041059765229 genomic sequence
contig-100_2571	715	N	0	0	NA	0	0	0	NA	NA	NA	NA
contig-100_2572	715	N	0	0	NA	0	0	0	NA	Bacteroides vulgatus ATCC 8482, complete genome	gi|150002608|ref|NC_009614.1| Bacteroides vulgatus ATCC 8482 chromosome, complete genome	gi|150002608|ref|NC_009614.1| Bacteroides vulgatus ATCC 8482 chromosome, complete genome

contig-100_2573	715	N	1	0	NA	0	0	0	NA	Propionibacterium acnes ATCC 11828, complete genome	gi|386069650|ref|NC_017550.1| Propionibacterium acnes ATCC 11828 chromosome, complete genome	gi|386069650|ref|NC_017550.1| Propionibacterium acnes ATCC 11828 chromosome, complete genome
contig-100_2574	715	N	0	0	NA	0	0	0	NA	Bacteroides vulgatus ATCC 8482, complete genome	gi|150002608|ref|NC_009614.1| Bacteroides vulgatus ATCC 8482 chromosome, complete genome	gi|150002608|ref|NC_009614.1| Bacteroides vulgatus ATCC 8482 chromosome, complete genome
contig-100_2575	715	N	0	0	NA	0	0	0	NA	NA	NA	NA
contig-100_2576	714	N	0	0	NA	0	0	0	NA	NA	NA	NA
contig-100_2578	714	N	0	0	NA	0	0	0	NA	Bacteroides vulgatus ATCC 8482, complete genome	gi|150002608|ref|NC_009614.1| Bacteroides vulgatus ATCC 8482 chromosome, complete genome	gi|150002608|ref|NC_009614.1| Bacteroides vulgatus ATCC 8482 chromosome, complete genome
contig-100_258	2921	N	3	0	NA	0	1	1	NA	Streptococcus thermophilus ND03, complete genome	gi|386085705|ref|NC_017563.1| Streptococcus thermophilus ND03 chromosome, complete genome	gi|386085705|ref|NC_017563.1| Streptococcus thermophilus ND03 chromosome, complete genome
contig-100_2580	712	N	0	0	NA	0	0	0	NA	NA	NA	NA
contig-100_2581	712	N	0	0	NA	0	0	0	NA	NA	NA	NA
contig-100_2582	712	N	0	0	NA	0	0	0	NA	NA	NA	NA
contig-100_2583	712	N	0	0	NA	0	0	0	NA	NA	NA	NA

contig-100_2584	712	N	0	0	NA	0	0	0	NA	Bacteroides vulgatus ATCC 8482, complete genome	gi|150002608|ref|NC_009614.1| Bacteroides vulgatus ATCC 8482 chromosome, complete genome	gi|150002608|ref|NC_009614.1| Bacteroides vulgatus ATCC 8482 chromosome, complete genome
contig-100_2585	712	N	0	0	NA	0	0	0	NA	Uncultured organism clone VC1CP31TF genomic sequence	gi|325297172|ref|NC_015164.1| Bacteroides salanitronis DSM 18170 chromosome, complete genome	gi|325297172|ref|NC_015164.1| Bacteroides salanitronis DSM 18170 chromosome, complete genome
contig-100_2586	712	N	0	0	NA	0	0	0	NA	NA	NA	NA
contig-100_2587	712	N	0	0	NA	0	0	0	NA	Uncultured organism clone 1041059765826 genomic sequence	NA	Uncultured organism clone 1041059765826 genomic sequence
contig-100_2588	711	N	0	0	NA	0	0	0	NA	NA	NA	NA
contig-100_2589	711	N	0	0	NA	0	0	0	NA	Bacteroides fragilis YCH46 DNA, complete genome	gi|53711291|ref|NC_006347.1| Bacteroides fragilis YCH46 DNA, complete genome	gi|53711291|ref|NC_006347.1| Bacteroides fragilis YCH46 DNA, complete genome
contig-100_259	2882	N	1	0	NA	0	0	0	NA	NA	NA	NA
contig-100_2590	711	N	0	0	NA	0	0	0	NA	NA	NA	NA
contig-100_2591	711	N	0	0	NA	0	0	0	NA	NA	NA	NA
contig-100_2592	711	N	0	0	NA	0	0	0	NA	NA	NA	NA
contig-100_2593	711	N	0	0	NA	0	0	0	NA	Uncultured organism clone 1041059766776 genomic sequence	NA	Uncultured organism clone 1041059766776 genomic sequence

contig-100_2594	711	N	0	0	NA	0	0	0	NA	Clostridiales sp. SSC/2 draft genome	gi|479158859|ref|NC_021016.1| Butyrate-producing bacterium SSC/2, complete genome	gi|479158859|ref|NC_021016.1| Butyrate-producing bacterium SSC/2, complete genome
contig-100_2596	711	N	1	0	NA	0	0	0	NA	NA	NA	NA
contig-100_2597	711	N	0	0	NA	0	0	0	NA	NA	NA	NA
contig-100_2598	711	N	0	0	NA	0	0	0	NA	NA	NA	NA
contig-100_2599	710	N	2	0	NA	0	0	0	NA	NA	NA	NA
contig-100_260	2873	N	2	2	Podo	0	0	0	NA	NA	NA	NA
contig-100_2600	710	N	1	0	NA	0	0	0	NA	Odoribacter splanchnicus DSM 20712, complete genome	gi|325278757|ref|NC_015160.1| Odoribacter splanchnicus DSM 20712 chromosome, complete genome	gi|325278757|ref|NC_015160.1| Odoribacter splanchnicus DSM 20712 chromosome, complete genome
contig-100_2601	710	N	0	0	NA	0	0	0	NA	Uncultured organism clone 1041059767554 genomic sequence	gi|319899888|ref|NC_014933.1| Bacteroides helcogenes P 36-108 chromosome, complete genome	gi|319899888|ref|NC_014933.1| Bacteroides helcogenes P 36-108 chromosome, complete genome
contig-100_2602	710	N	0	0	NA	0	0	0	NA	Clostridiales sp. SS3/4 draft genome	gi|479146200|ref|NC_021012.1| Roseburia intestinalis XB6B4 draft genome	gi|479146200|ref|NC_021012.1| Roseburia intestinalis XB6B4 draft genome
contig-100_2603	710	N	1	0	NA	0	0	0	NA	Bacteroides fragilis clone 38-F transposon Tet element BTF-37, partial sequence	NA	Bacteroides fragilis clone 38-F transposon Tet element BTF-37, partial sequence

contig-100_2604	709	N	1	0	NA	0	0	0	NA	Faecalibacterium prausnitzii SL3/3 draft genome	gi|479170689|ref|NC_021020.1| Faecalibacterium prausnitzii SL3/3 draft genome	gi|479170689|ref|NC_021020.1| Faecalibacterium prausnitzii SL3/3 draft genome
contig-100_2605	709	N	0	0	NA	0	0	0	NA	Bacteroides vulgatus ATCC 8482, complete genome	gi|150002608|ref|NC_009614.1| Bacteroides vulgatus ATCC 8482 chromosome, complete genome	gi|150002608|ref|NC_009614.1| Bacteroides vulgatus ATCC 8482 chromosome, complete genome
contig-100_2607	709	N	0	0	NA	0	0	0	NA	Uncultured organism clone VC1CX60TF genomic sequence	gi|325297172|ref|NC_015164.1| Bacteroides salanitronis DSM 18170 chromosome, complete genome	gi|325297172|ref|NC_015164.1| Bacteroides salanitronis DSM 18170 chromosome, complete genome
contig-100_2608	709	N	0	0	NA	0	0	0	NA	NA	NA	NA
contig-100_2609	709	N	0	0	NA	0	0	0	NA	NA	NA	NA
contig-100_261	2868	N	1	0	NA	0	0	0	NA	Clostridiales sp. SM4/1 draft genome	gi|479181986|ref|NC_021024.1| Butyrate-producing bacterium SM4/1, complete genome	gi|479181986|ref|NC_021024.1| Butyrate-producing bacterium SM4/1, complete genome
contig-100_2610	709	N	0	0	NA	0	0	0	NA	NA	NA	NA
contig-100_2611	709	N	0	0	NA	0	0	0	NA	Clostridiales sp. SS3/4 draft genome	gi|479192860|ref|NC_021035.1| Butyrate-producing bacterium SS3/4, complete genome	gi|479192860|ref|NC_021035.1| Butyrate-producing bacterium SS3/4, complete genome
contig-100_2612	709	N	0	0	NA	0	0	0	NA	NA	NA	NA
contig-100_2613	708	N	0	0	NA	0	0	0	NA	NA	NA	NA
contig-100_2614	708	N	1	0	NA	0	0	0	NA	NA	NA	NA

contig-100_2615	708	N	1	0	NA	0	0	0	NA	Bacteroides salanitronis DSM 18170, complete genome	gi|325297172|ref|NC_015164.1| Bacteroides salanitronis DSM 18170 chromosome, complete genome	gi|325297172|ref|NC_015164.1| Bacteroides salanitronis DSM 18170 chromosome, complete genome
contig-100_2616	708	N	1	0	NA	0	0	0	NA	Faecalibacterium prausnitzii SL3/3 draft genome	gi|479170689|ref|NC_021020.1| Faecalibacterium prausnitzii SL3/3 draft genome	gi|479170689|ref|NC_021020.1| Faecalibacterium prausnitzii SL3/3 draft genome
contig-100_2617	708	N	1	0	NA	0	0	0	NA	NA	NA	NA
contig-100_2618	708	N	0	0	NA	0	0	0	NA	NA	NA	NA
contig-100_2619	707	N	0	0	NA	0	0	0	NA	Uncultured organism clone VC1CK75TF genomic sequence	gi|479170689|ref|NC_021020.1| Faecalibacterium prausnitzii SL3/3 draft genome	gi|479170689|ref|NC_021020.1| Faecalibacterium prausnitzii SL3/3 draft genome
contig-100_262	2857	N	0	0	NA	0	0	0	NA	NA	NA	NA
contig-100_2620	707	N	1	0	NA	0	0	0	NA	NA	NA	NA
contig-100_2621	707	N	0	0	NA	0	0	0	NA	NA	NA	NA
contig-100_2623	707	N	1	0	NA	0	0	0	NA	NA	NA	NA
contig-100_2624	707	N	0	0	NA	0	0	0	NA	Haemophilus influenzae KR494, complete genome	gi|543951066|ref|NC_022356.1| Haemophilus influenzae KR494, complete genome	gi|543951066|ref|NC_022356.1| Haemophilus influenzae KR494, complete genome
contig-100_2625	706	N	1	0	NA	0	0	0	NA	NA	NA	NA
contig-100_2626	706	N	0	0	NA	0	0	0	NA	Bacteroides vulgatus ATCC 8482, complete genome	gi|150002608|ref|NC_009614.1| Bacteroides vulgatus ATCC 8482 chromosome, complete genome	gi|150002608|ref|NC_009614.1| Bacteroides vulgatus ATCC 8482 chromosome, complete genome
contig-100_2627	706	N	1	0	NA	0	0	0	NA	NA	NA	NA
contig-100_2628	706	N	2	0	NA	0	0	0	NA	NA	NA	NA

contig-100_2629	706	N	1	0	NA	0	0	0	NA	Faecalibacterium prausnitzii SL3/3 draft genome	gi|479170689|ref|NC_021020.1| Faecalibacterium prausnitzii SL3/3 draft genome	gi|479170689|ref|NC_021020.1| Faecalibacterium prausnitzii SL3/3 draft genome
contig-100_2630	706	N	0	0	NA	0	0	0	NA	Bacteroides vulgatus ATCC 8482, complete genome	gi|150002608|ref|NC_009614.1| Bacteroides vulgatus ATCC 8482 chromosome, complete genome	gi|150002608|ref|NC_009614.1| Bacteroides vulgatus ATCC 8482 chromosome, complete genome
contig-100_2631	705	N	1	0	NA	0	0	0	NA	NA	NA	NA
contig-100_2632	705	N	1	0	NA	0	0	0	NA	Uncultured organism clone 1041059765713 genomic sequence	NA	Uncultured organism clone 1041059765713 genomic sequence
contig-100_2633	705	N	0	0	NA	0	0	0	NA	NA	NA	NA
contig-100_2634	705	N	0	0	NA	0	0	0	NA	NA	NA	NA
contig-100_2635	705	N	1	0	NA	0	0	0	NA	Uncultured bacterium clone LM0ACA20ZA06RM1 genomic sequence	NA	Uncultured bacterium clone LM0ACA20ZA06RM1 genomic sequence
contig-100_2637	704	N	0	0	NA	0	0	0	NA	NA	NA	NA
contig-100_2638	704	N	0	0	NA	0	0	0	NA	NA	NA	NA
contig-100_2639	704	N	1	0	NA	0	0	0	NA	Bacteroides vulgatus ATCC 8482, complete genome	gi|150002608|ref|NC_009614.1| Bacteroides vulgatus ATCC 8482 chromosome, complete genome	gi|150002608|ref|NC_009614.1| Bacteroides vulgatus ATCC 8482 chromosome, complete genome
contig-100_264	2849	N	2	0	NA	0	1	1	NA	Uncultured organism clone VC1CK33TF genomic sequence	gi|479150083|ref|NC_021013.1| Ruminococcus bromii L2-63 draft genome	gi|479150083|ref|NC_021013.1| Ruminococcus bromii L2-63 draft genome

contig-100_2640	704	N	0	0	NA	0	0	0	NA	NA	NA	NA
contig-100_2641	704	N	0	0	NA	0	0	0	NA	Uncultured organism clone 1041059766302 genomic sequence	gi|82701135|ref|NC_007614.1| Nitrosospira multiformis ATCC 25196 chromosome, complete genome	gi|82701135|ref|NC_007614.1| Nitrosospira multiformis ATCC 25196 chromosome, complete genome
contig-100_2642	704	N	0	0	NA	0	0	0	NA	NA	NA	NA
contig-100_2643	703	N	1	0	NA	0	0	0	NA	NA	NA	NA
contig-100_2644	703	N	1	0	NA	0	0	0	NA	NA	NA	NA
contig-100_2645	703	N	0	0	NA	0	0	0	NA	NA	NA	NA
contig-100_2646	703	N	1	0	NA	0	0	0	NA	Faecalibacterium prausnitzii L2/6 draft genome	gi|479208076|ref|NC_021042.1| Faecalibacterium prausnitzii L2-6, complete genome	gi|479208076|ref|NC_021042.1| Faecalibacterium prausnitzii L2-6, complete genome
contig-100_2647	702	N	0	0	NA	0	0	0	NA	NA	NA	NA
contig-100_2648	702	N	0	0	NA	0	0	0	NA	NA	NA	NA
contig-100_265	2848	N	3	0	NA	1	0	1	NA	NA	NA	NA
contig-100_2650	702	N	1	0	NA	0	0	0	NA	NA	NA	NA
contig-100_2651	702	N	0	0	NA	0	0	0	NA	Faecalibacterium prausnitzii L2/6 draft genome	gi|479208076|ref|NC_021042.1| Faecalibacterium prausnitzii L2-6, complete genome	gi|479208076|ref|NC_021042.1| Faecalibacterium prausnitzii L2-6, complete genome
contig-100_2652	701	N	0	0	NA	0	0	0	NA	NA	NA	NA
contig-100_2653	701	N	0	0	NA	0	0	0	NA	NA	NA	NA
contig-100_2654	701	N	0	0	NA	0	0	0	NA	NA	NA	NA
contig-100_2655	701	N	0	0	NA	0	0	0	NA	NA	NA	NA
contig-100_2656	700	N	0	0	NA	0	0	0	NA	NA	NA	NA
contig-100_2657	700	N	0	0	NA	0	0	0	NA	NA	NA	NA
contig-100_2658	700	N	0	0	NA	0	0	0	NA	NA	NA	NA

contig-100_2659	700	N	0	0	NA	0	0	0	NA	Haemophilus influenzae 10810 genome	gi|378696079|ref|NC_016809.1| Haemophilus influenzae 10810, complete genome	gi|378696079|ref|NC_016809.1| Haemophilus influenzae 10810, complete genome
contig-100_2660	700	N	1	0	NA	0	0	0	NA	NA	NA	NA
contig-100_2661	700	N	0	0	NA	0	0	0	NA	NA	NA	NA
contig-100_2663	700	N	0	0	NA	0	0	0	NA	NA	NA	NA
contig-100_2664	700	N	1	0	NA	0	0	0	NA	NA	NA	NA
contig-100_2665	700	N	0	0	NA	0	0	0	NA	NA	NA	NA
contig-100_2666	700	N	0	0	NA	0	0	0	NA	NA	NA	NA
contig-100_2667	700	N	2	0	NA	0	0	0	NA	NA	NA	NA
contig-100_2668	700	N	0	0	NA	0	0	0	NA	NA	NA	NA
contig-100_267	2840	N	2	0	NA	0	0	0	NA	Faecalibacterium prausnitzii SL3/3 draft genome	gi|479170689|ref|NC_021020.1| Faecalibacterium prausnitzii SL3/3 draft genome	gi|479170689|ref|NC_021020.1| Faecalibacterium prausnitzii SL3/3 draft genome
contig-100_2670	699	N	0	0	NA	0	0	0	NA	Conjugally transferable red recombinase vector pMJH46, complete sequence	gi|238899406|ref|NC_012759.1| Escherichia coli BW2952 chromosome, complete genome	gi|238899406|ref|NC_012759.1| Escherichia coli BW2952 chromosome, complete genome
contig-100_2671	699	N	0	0	NA	0	0	0	NA	NA	NA	NA
contig-100_2672	699	N	0	0	NA	0	0	0	NA	NA	NA	NA
contig-100_2673	699	N	2	0	NA	0	0	0	NA	NA	NA	NA
contig-100_2675	698	N	0	0	NA	0	0	0	NA	Actinobacillus suis H91-0380, complete genome	gi|407691594|ref|NC_018690.1| Actinobacillus suis H91-0380 chromosome, complete genome	gi|407691594|ref|NC_018690.1| Actinobacillus suis H91-0380 chromosome, complete genome
contig-100_2676	698	N	0	0	NA	0	0	0	NA	NA	NA	NA
contig-100_2677	698	N	0	0	NA	0	0	0	NA	NA	NA	NA
contig-100_2678	698	N	0	0	NA	0	0	0	NA	NA	NA	NA
contig-100_2679	697	N	0	0	NA	0	0	0	NA	NA	NA	NA
contig-100_268	2839	N	1	0	NA	0	0	0	NA	NA	NA	NA
contig-100_2680	697	N	0	0	NA	0	0	0	NA	NA	NA	NA

contig-100_2681	697	N	1	0	NA	0	0	0	NA	NA	NA	NA
contig-100_2682	697	N	0	0	NA	0	0	0	NA	Lactobacillus helveticus CNRZ32, complete genome	gi|525706521|ref|NC_021744.1| Lactobacillus helveticus CNRZ32, complete genome	gi|525706521|ref|NC_021744.1| Lactobacillus helveticus CNRZ32, complete genome
contig-100_2683	697	N	1	0	NA	0	0	0	NA	NA	NA	NA
contig-100_2684	697	N	1	0	NA	0	0	0	NA	NA	NA	NA
contig-100_2685	696	N	0	0	NA	0	0	0	NA	Desulfovibrio salexigens DSM 2638, complete genome	gi|242277482|ref|NC_012881.1| Desulfovibrio salexigens DSM 2638 chromosome, complete genome	gi|242277482|ref|NC_012881.1| Desulfovibrio salexigens DSM 2638 chromosome, complete genome
contig-100_2686	696	N	0	0	NA	0	0	0	NA	Bacteroides thetaiotaomicron VPI-5482, complete genome	gi|29345410|ref|NC_004663.1| Bacteroides thetaiotaomicron VPI-5482 chromosome, complete genome	gi|29345410|ref|NC_004663.1| Bacteroides thetaiotaomicron VPI-5482 chromosome, complete genome
contig-100_2687	696	N	0	0	NA	0	0	0	NA	NA	NA	NA
contig-100_2688	696	N	0	0	NA	0	0	0	NA	Faecalibacterium prausnitzii SL3/3 draft genome	gi|479170689|ref|NC_021020.1| Faecalibacterium prausnitzii SL3/3 draft genome	gi|479170689|ref|NC_021020.1| Faecalibacterium prausnitzii SL3/3 draft genome
contig-100_2689	696	N	2	0	NA	0	0	0	NA	Acidovorax sp. JS42, complete genome	gi|121592436|ref|NC_008782.1| Acidovorax sp. JS42 chromosome, complete genome	gi|121592436|ref|NC_008782.1| Acidovorax sp. JS42 chromosome, complete genome
contig-100_269	2837	N	3	3	Sipho	0	0	3	NA	NA	NA	NA
contig-100_2691	695	N	0	0	NA	0	0	0	NA	NA	NA	NA

contig-100_2692	695	N	0	0	NA	0	0	0	NA	Clostridium carboxidivorans P7 bifunctional alcohol/acetaldehyde dehydrogenase gene, complete cds	NA	Clostridium carboxidivorans P7 bifunctional alcohol/acetaldehyde dehydrogenase gene, complete cds
contig-100_2693	695	N	0	0	NA	0	0	0	NA	NA	NA	NA
contig-100_2695	695	N	0	0	NA	0	0	0	NA	Bacteroides vulgatus ATCC 8482, complete genome	gi|150002608|ref|NC_009614.1| Bacteroides vulgatus ATCC 8482 chromosome, complete genome	gi|150002608|ref|NC_009614.1| Bacteroides vulgatus ATCC 8482 chromosome, complete genome
contig-100_2696	694	N	0	0	NA	0	0	0	NA	NA	NA	NA
contig-100_2697	694	N	0	0	NA	0	0	0	NA	Bacteroides vulgatus ATCC 8482, complete genome	gi|150002608|ref|NC_009614.1| Bacteroides vulgatus ATCC 8482 chromosome, complete genome	gi|150002608|ref|NC_009614.1| Bacteroides vulgatus ATCC 8482 chromosome, complete genome
contig-100_2698	694	N	0	0	NA	0	0	0	NA	NA	NA	NA
contig-100_2699	693	N	0	0	NA	0	0	0	NA	NA	NA	NA
contig-100_2701	693	N	1	0	NA	0	0	0	NA	Uncultured bacterium clone LM0ABA4ZE02RM1 genomic sequence	gi|327312315|ref|NC_015311.1| Prevotella denticola F0289 chromosome, complete genome	gi|327312315|ref|NC_015311.1| Prevotella denticola F0289 chromosome, complete genome
contig-100_2702	693	N	0	0	NA	0	0	0	NA	NA	NA	NA
contig-100_2703	692	N	0	0	NA	0	0	0	NA	NA	NA	NA
contig-100_2704	692	N	0	0	NA	0	0	0	NA	Bacteroides vulgatus ATCC 8482, complete genome	gi|150002608|ref|NC_009614.1| Bacteroides vulgatus ATCC 8482 chromosome, complete genome	gi|150002608|ref|NC_009614.1| Bacteroides vulgatus ATCC 8482 chromosome, complete genome

contig-100_2705	692	N	0	0	NA	0	0	0	NA	Haemophilus influenzae KR494, complete genome	gi|543951066|ref|NC_022356.1| Haemophilus influenzae KR494, complete genome	gi|543951066|ref|NC_022356.1| Haemophilus influenzae KR494, complete genome
contig-100_2706	691	N	0	0	NA	0	0	0	NA	Eubacterium rectale ATCC 33656, complete genome	gi|238922432|ref|NC_012781.1| Eubacterium rectale ATCC 33656, complete genome	gi|238922432|ref|NC_012781.1| Eubacterium rectale ATCC 33656, complete genome
contig-100_2707	691	N	1	0	NA	0	0	0	NA	NA	NA	NA
contig-100_2708	690	N	0	0	NA	0	0	0	NA	Ruminococcus torques L2-14 draft genome	gi|479155735|ref|NC_021015.1| Ruminococcus torques L2-14 draft genome	gi|479155735|ref|NC_021015.1| Ruminococcus torques L2-14 draft genome
contig-100_2709	690	N	0	0	NA	0	0	0	NA	NA	NA	NA
contig-100_271	2828	N	1	0	NA	0	0	0	NA	Pasteurellaceae tandem reporter plasmid pMC-Tandem, complete sequence	gi|170717206|ref|NC_010519.1| Haemophilus somnus 2336 chromosome, complete genome	gi|170717206|ref|NC_010519.1| Haemophilus somnus 2336 chromosome, complete genome
contig-100_2711	689	N	0	0	NA	0	0	0	NA	NA	NA	NA
contig-100_2712	689	N	0	0	NA	0	0	0	NA	NA	NA	NA
contig-100_2713	689	N	0	0	NA	0	0	0	NA	Veillonella parvula DSM 2008, complete genome	gi|269797069|ref|NC_013520.1| Veillonella parvula DSM 2008 chromosome, complete genome	gi|269797069|ref|NC_013520.1| Veillonella parvula DSM 2008 chromosome, complete genome
contig-100_2714	689	N	0	0	NA	0	0	0	NA	NA	NA	NA
contig-100_2715	688	N	1	0	NA	0	0	0	NA	NA	NA	NA
contig-100_2716	688	N	0	0	NA	0	0	0	NA	NA	NA	NA
contig-100_2717	688	N	0	0	NA	0	0	0	NA	NA	NA	NA
contig-100_2718	688	N	0	0	NA	0	0	0	NA	NA	NA	NA

contig-100_2719	688	N	0	0	NA	0	0	0	NA	NA	NA	NA
contig-100_272	2824	N	2	0	NA	0	0	0	NA	Roseburia hominis A2-183, complete genome	gi|347530298|ref|NC_015977.1| Roseburia hominis A2-183 chromosome, complete genome	gi|347530298|ref|NC_015977.1| Roseburia hominis A2-183 chromosome, complete genome
contig-100_2720	688	N	0	0	NA	0	0	0	NA	NA	NA	NA
contig-100_2721	688	N	0	0	NA	0	0	0	NA	Faecalibacterium prausnitzii L2/6 draft genome	gi|479208076|ref|NC_021042.1| Faecalibacterium prausnitzii L2-6, complete genome	gi|479208076|ref|NC_021042.1| Faecalibacterium prausnitzii L2-6, complete genome
contig-100_2722	688	N	0	0	NA	0	0	0	NA	Bacteroides vulgatus ATCC 8482, complete genome	gi|150002608|ref|NC_009614.1| Bacteroides vulgatus ATCC 8482 chromosome, complete genome	gi|150002608|ref|NC_009614.1| Bacteroides vulgatus ATCC 8482 chromosome, complete genome
contig-100_2724	687	N	1	0	NA	0	0	0	NA	Veillonella parvula DSM 2008, complete genome	gi|269797069|ref|NC_013520.1| Veillonella parvula DSM 2008 chromosome, complete genome	gi|269797069|ref|NC_013520.1| Veillonella parvula DSM 2008 chromosome, complete genome
contig-100_2725	687	N	0	0	NA	0	0	0	NA	Streptococcus thermophilus bacteriophage Sfi19, complete genome	NA	Streptococcus thermophilus bacteriophage Sfi19, complete genome
contig-100_2727	687	N	0	0	NA	0	0	0	NA	Bacteroides thetaiotaomicron VPI-5482, complete genome	gi|29345410|ref|NC_004663.1| Bacteroides thetaiotaomicron VPI-5482 chromosome, complete genome	gi|29345410|ref|NC_004663.1| Bacteroides thetaiotaomicron VPI-5482 chromosome, complete genome

contig-100_2728	687	N	0	0	NA	0	0	0	NA	Haemophilus parainfluenzae T3T1 complete genome	gi|345428590|ref|NC_015964.1| Haemophilus parainfluenzae T3T1, complete genome	gi|345428590|ref|NC_015964.1| Haemophilus parainfluenzae T3T1, complete genome
contig-100_2729	687	N	1	0	NA	0	0	0	NA	NA	NA	NA
contig-100_273	2805	N	3	0	NA	0	0	0	NA	NA	NA	NA
contig-100_2730	687	N	0	0	NA	0	0	0	NA	NA	gi|302872922|ref|NC_014393.1| Clostridium cellulovorans 743B chromosome, complete genome	gi|302872922|ref|NC_014393.1| Clostridium cellulovorans 743B chromosome, complete genome
contig-100_2731	686	N	1	0	NA	0	0	0	NA	Faecalibacterium prausnitzii L2/6 draft genome	gi|479208076|ref|NC_021042.1| Faecalibacterium prausnitzii L2-6, complete genome	gi|479208076|ref|NC_021042.1| Faecalibacterium prausnitzii L2-6, complete genome
contig-100_2732	686	N	1	0	NA	0	0	0	NA	NA	NA	NA
contig-100_2733	686	N	0	0	NA	0	0	0	NA	Bacteroides vulgatus ATCC 8482, complete genome	gi|150002608|ref|NC_009614.1| Bacteroides vulgatus ATCC 8482 chromosome, complete genome	gi|150002608|ref|NC_009614.1| Bacteroides vulgatus ATCC 8482 chromosome, complete genome
contig-100_2735	686	N	0	0	NA	0	0	0	NA	NA	NA	NA
contig-100_2736	685	N	0	0	NA	0	0	0	NA	NA	NA	NA
contig-100_2737	685	N	1	0	NA	0	0	0	NA	Bacteroides vulgatus ATCC 8482, complete genome	gi|150002608|ref|NC_009614.1| Bacteroides vulgatus ATCC 8482 chromosome, complete genome	gi|150002608|ref|NC_009614.1| Bacteroides vulgatus ATCC 8482 chromosome, complete genome
contig-100_2738	685	N	0	0	NA	0	0	0	NA	NA	NA	NA
contig-100_2739	685	N	1	0	NA	0	0	0	NA	NA	NA	NA

contig-100_274	2803	N	2	0	NA	0	0	1	NA	Uncultured organism clone 1041059765627 genomic sequence	gi|29345410|ref|NC_004663.1| Bacteroides thetaiotaomicron VPI-5482 chromosome, complete genome	gi|29345410|ref|NC_004663.1| Bacteroides thetaiotaomicron VPI-5482 chromosome, complete genome
contig-100_2740	685	N	0	0	NA	0	0	0	NA	NA	NA	NA
contig-100_2741	685	N	1	0	NA	0	0	0	NA	Faecalibacterium prausnitzii L2/6 draft genome	gi|479208076|ref|NC_021042.1| Faecalibacterium prausnitzii L2-6, complete genome	gi|479208076|ref|NC_021042.1| Faecalibacterium prausnitzii L2-6, complete genome
contig-100_2742	684	N	0	0	NA	0	0	0	NA	NA	NA	NA
contig-100_2743	684	N	0	0	NA	0	0	0	NA	NA	NA	NA
contig-100_2745	683	N	1	0	NA	0	0	0	NA	NA	NA	NA
contig-100_2746	683	N	1	0	NA	0	0	0	NA	NA	NA	NA
contig-100_2747	683	N	1	0	NA	0	0	0	NA	Faecalibacterium prausnitzii SL3/3 draft genome	gi|479170689|ref|NC_021020.1| Faecalibacterium prausnitzii SL3/3 draft genome	gi|479170689|ref|NC_021020.1| Faecalibacterium prausnitzii SL3/3 draft genome
contig-100_2748	683	N	0	0	NA	0	0	0	NA	NA	NA	NA
contig-100_2749	683	N	0	0	NA	0	0	0	NA	Clostridiales sp. SSC/2 draft genome	gi|479158859|ref|NC_021016.1| Butyrate-producing bacterium SSC/2, complete genome	gi|479158859|ref|NC_021016.1| Butyrate-producing bacterium SSC/2, complete genome
contig-100_275	2796	N	2	0	NA	0	0	0	NA	Streptococcus pyogenes MGAS10750, complete genome	gi|94987631|ref|NC_008021.1| Streptococcus pyogenes MGAS9429 chromosome, complete genome	gi|94987631|ref|NC_008021.1| Streptococcus pyogenes MGAS9429 chromosome, complete genome
contig-100_2750	683	N	0	0	NA	0	0	0	NA	NA	NA	NA
contig-100_2751	682	N	0	0	NA	0	0	0	NA	NA	NA	NA

contig-100_2752	682	N	0	0	NA	0	0	0	NA	Faecalibacterium prausnitzii SL3/3 draft genome	gi|479170689|ref|NC_021020.1| Faecalibacterium prausnitzii SL3/3 draft genome	gi|479170689|ref|NC_021020.1| Faecalibacterium prausnitzii SL3/3 draft genome
contig-100_2753	682	N	0	0	NA	0	0	0	NA	Uncultured organism clone 1041059767808 genomic sequence	gi|479162165|ref|NC_021017.1| Bacteroides xylanisolvens XB1A draft genome	gi|479162165|ref|NC_021017.1| Bacteroides xylanisolvens XB1A draft genome
contig-100_2754	682	N	0	0	NA	0	0	0	NA	NA	NA	NA
contig-100_2755	682	N	1	0	NA	0	0	0	NA	Bacteroides fragilis YCH46 DNA, complete genome	gi|53711291|ref|NC_006347.1| Bacteroides fragilis YCH46 DNA, complete genome	gi|53711291|ref|NC_006347.1| Bacteroides fragilis YCH46 DNA, complete genome
contig-100_2756	682	N	0	0	NA	0	0	0	NA	NA	NA	NA
contig-100_2757	682	N	1	0	NA	0	0	0	NA	NA	NA	NA
contig-100_2758	682	N	0	0	NA	0	0	0	NA	NA	NA	NA
contig-100_2759	681	N	0	0	NA	0	0	0	NA	Bacteroides thetaiotaomicron VPI-5482, complete genome	gi|29345410|ref|NC_004663.1| Bacteroides thetaiotaomicron VPI-5482 chromosome, complete genome	gi|29345410|ref|NC_004663.1| Bacteroides thetaiotaomicron VPI-5482 chromosome, complete genome
contig-100_2760	681	N	0	0	NA	0	0	0	NA	NA	NA	NA
contig-100_2761	681	N	0	0	NA	0	0	0	NA	Faecalibacterium prausnitzii L2/6 draft genome	gi|479208076|ref|NC_021042.1| Faecalibacterium prausnitzii L2-6, complete genome	gi|479208076|ref|NC_021042.1| Faecalibacterium prausnitzii L2-6, complete genome

contig-100_2762	681	N	0	0	NA	0	0	0	NA	Clostridiales sp. SSC/2 draft genome	gi|479158859|ref|NC_021016.1| Butyrate-producing bacterium SSC/2, complete genome	gi|479158859|ref|NC_021016.1| Butyrate-producing bacterium SSC/2, complete genome
contig-100_2763	681	N	0	0	NA	0	0	0	NA	NA	NA	NA
contig-100_2764	681	N	0	0	NA	0	0	0	NA	Bacteroides vulgatus ATCC 8482, complete genome	gi|150002608|ref|NC_009614.1| Bacteroides vulgatus ATCC 8482 chromosome, complete genome	gi|150002608|ref|NC_009614.1| Bacteroides vulgatus ATCC 8482 chromosome, complete genome
contig-100_2765	680	N	1	0	NA	0	0	0	NA	NA	NA	NA
contig-100_2766	680	N	0	0	NA	0	0	0	NA	NA	NA	NA
contig-100_2767	680	N	0	0	NA	0	0	0	NA	NA	NA	NA
contig-100_2768	680	N	0	0	NA	0	0	0	NA	NA	NA	NA
contig-100_2769	680	N	0	0	NA	0	0	0	NA	Eubacterium rectale DSM 17629 draft genome	gi|479140210|ref|NC_021010.1| Eubacterium rectale DSM 17629 draft genome	gi|479140210|ref|NC_021010.1| Eubacterium rectale DSM 17629 draft genome
contig-100_2770	680	N	0	0	NA	0	0	0	NA	Propionibacterium acnes HL096PA1, complete genome	gi|365972921|ref|NC_016516.1| Propionibacterium acnes TypeIA2 P.acn33 chromosome, complete genome	gi|365972921|ref|NC_016516.1| Propionibacterium acnes TypeIA2 P.acn33 chromosome, complete genome
contig-100_2771	679	N	0	0	NA	0	0	0	NA	Bacteroides vulgatus ATCC 8482, complete genome	gi|150002608|ref|NC_009614.1| Bacteroides vulgatus ATCC 8482 chromosome, complete genome	gi|150002608|ref|NC_009614.1| Bacteroides vulgatus ATCC 8482 chromosome, complete genome
contig-100_2773	679	N	0	0	NA	0	0	0	NA	NA	NA	NA
contig-100_2774	679	N	1	0	NA	0	0	0	NA	NA	NA	NA

contig-100_2775	679	N	0	0	NA	0	0	0	NA	Uncultured bacterium clone LM0ABA39ZH01FM1 genomic sequence	gi|29345410|ref|NC_004663.1| Bacteroides thetaiotaomicron VPI-5482 chromosome, complete genome	gi|29345410|ref|NC_004663.1| Bacteroides thetaiotaomicron VPI-5482 chromosome, complete genome
contig-100_2776	678	N	0	0	NA	0	0	0	NA	Unidentified phage clone 1013_scaffold47 genomic sequence	NA	Unidentified phage clone 1013_scaffold47 genomic sequence
contig-100_2777	678	N	0	0	NA	0	0	0	NA	NA	NA	NA
contig-100_2778	678	N	1	0	NA	0	0	0	NA	Bacterium enrichment culture clone P47-5C putative regulator gene, partial cds; putative 6-phospho-beta-glucosidase A and suppressor of copper sensitivity protein A genes, complete cds; and suppressor of copper sensitivity protein B gene, partial cds	gi|529985600|ref|NC_021232.1| Klebsiella pneumoniae subsp. rhinoscleromatis strain SB3432, complete genome	gi|529985600|ref|NC_021232.1| Klebsiella pneumoniae subsp. rhinoscleromatis strain SB3432, complete genome
contig-100_2779	678	N	1	0	NA	0	0	0	NA	Uncultured bacterium clone HA0AAA18ZF02RM1 genomic sequence	NA	Uncultured bacterium clone HA0AAA18ZF02RM1 genomic sequence
contig-100_278	2785	N	1	1	NA	0	0	1	NA	Cloning vector pUC19c, complete sequence	gi|321311376|ref|NC_014976.1| Bacillus subtilis BSn5 chromosome, complete genome	gi|321311376|ref|NC_014976.1| Bacillus subtilis BSn5 chromosome, complete genome
contig-100_2780	678	N	0	0	NA	0	0	0	NA	NA	NA	NA
contig-100_2781	678	N	0	0	NA	0	0	0	NA	Unidentified phage clone 2019_scaffold132 genomic sequence	NA	Unidentified phage clone 2019_scaffold132 genomic sequence

contig-100_2782	678	N	0	0	NA	0	0	0	NA	Faecalibacterium prausnitzii SL3/3 draft genome	gi|479170689|ref|NC_021020.1| Faecalibacterium prausnitzii SL3/3 draft genome	gi|479170689|ref|NC_021020.1| Faecalibacterium prausnitzii SL3/3 draft genome
contig-100_2783	678	N	0	0	NA	0	0	0	NA	Bacteroides thetaiotaomicron VPI-5482, complete genome	gi|29345410|ref|NC_004663.1| Bacteroides thetaiotaomicron VPI-5482 chromosome, complete genome	gi|29345410|ref|NC_004663.1| Bacteroides thetaiotaomicron VPI-5482 chromosome, complete genome
contig-100_2784	677	N	0	0	NA	0	0	0	NA	Faecalibacterium prausnitzii L2/6 draft genome	gi|479208076|ref|NC_021042.1| Faecalibacterium prausnitzii L2-6, complete genome	gi|479208076|ref|NC_021042.1| Faecalibacterium prausnitzii L2-6, complete genome
contig-100_2785	677	N	0	0	NA	0	0	0	NA	Haemophilus influenzae F3031 complete genome	gi|319896422|ref|NC_014920.1| Haemophilus influenzae F3031 chromosome, complete genome	gi|319896422|ref|NC_014920.1| Haemophilus influenzae F3031 chromosome, complete genome
contig-100_2787	677	N	0	0	NA	0	0	0	NA	NA	NA	NA
contig-100_2789	677	N	0	0	NA	0	0	0	NA	Faecalibacterium prausnitzii SL3/3 draft genome	gi|479170689|ref|NC_021020.1| Faecalibacterium prausnitzii SL3/3 draft genome	gi|479170689|ref|NC_021020.1| Faecalibacterium prausnitzii SL3/3 draft genome
contig-100_2790	677	N	0	0	NA	0	0	0	NA	Clostridiales sp. SSC/2 draft genome	gi|479158859|ref|NC_021016.1| Butyrate-producing bacterium SSC/2, complete genome	gi|479158859|ref|NC_021016.1| Butyrate-producing bacterium SSC/2, complete genome

contig-100_2791	677	N	0	0	NA	0	0	0	NA	Bacteroides vulgatus ATCC 8482, complete genome	gi|150002608|ref|NC_009614.1| Bacteroides vulgatus ATCC 8482 chromosome, complete genome	gi|150002608|ref|NC_009614.1| Bacteroides vulgatus ATCC 8482 chromosome, complete genome
contig-100_2792	676	N	0	0	NA	0	0	0	NA	NA	NA	NA
contig-100_2793	676	N	0	0	NA	0	0	0	NA	NA	NA	NA
contig-100_2794	676	N	0	0	NA	0	0	0	NA	NA	NA	NA
contig-100_2795	676	N	1	0	NA	0	0	0	NA	NA	NA	NA
contig-100_2796	676	N	0	0	NA	0	0	0	NA	Uncultured organism clone 1041059766417 genomic sequence	NA	Uncultured organism clone 1041059766417 genomic sequence
contig-100_2797	676	N	1	0	NA	0	0	0	NA	NA	NA	NA
contig-100_2799	675	N	0	0	NA	0	0	0	NA	Eubacterium eligens ATCC 27750, complete genome	gi|238915976|ref|NC_012778.1| Eubacterium eligens ATCC 27750 chromosome, complete genome	gi|238915976|ref|NC_012778.1| Eubacterium eligens ATCC 27750 chromosome, complete genome
contig-100_28	11092	N	11	0	NA	0	3	1	NA	Klebsiella pneumoniae KCTC 2242, complete genome	gi|550443072|ref|NC_022566.1| Klebsiella pneumoniae CG43, complete genome	gi|550443072|ref|NC_022566.1| Klebsiella pneumoniae CG43, complete genome
contig-100_2800	675	N	0	0	NA	0	0	0	NA	NA	NA	NA
contig-100_2801	675	N	0	0	NA	0	0	0	NA	Faecalibacterium prausnitzii SL3/3 draft genome	gi|479170689|ref|NC_021020.1| Faecalibacterium prausnitzii SL3/3 draft genome	gi|479170689|ref|NC_021020.1| Faecalibacterium prausnitzii SL3/3 draft genome
contig-100_2802	675	N	1	0	NA	0	0	0	NA	NA	NA	NA
contig-100_2803	674	N	1	0	NA	0	0	0	NA	NA	NA	NA

contig-100_2804	674	N	0	0	NA	0	0	0	NA	Faecalibacterium prausnitzii SL3/3 draft genome	gi|479170689|ref|NC_021020.1| Faecalibacterium prausnitzii SL3/3 draft genome	gi|479170689|ref|NC_021020.1| Faecalibacterium prausnitzii SL3/3 draft genome
contig-100_2805	674	N	0	0	NA	0	0	0	NA	NA	NA	NA
contig-100_2806	674	N	0	0	NA	0	0	0	NA	NA	NA	NA
contig-100_2807	673	N	1	0	NA	0	0	0	NA	Uncultured organism clone VC1C616TF genomic sequence	gi|319899888|ref|NC_014933.1| Bacteroides helcogenes P 36-108 chromosome, complete genome	gi|319899888|ref|NC_014933.1| Bacteroides helcogenes P 36-108 chromosome, complete genome
contig-100_2808	673	N	0	0	NA	0	0	0	NA	Haemophilus parainfluenzae T3T1 complete genome	gi|345428590|ref|NC_015964.1| Haemophilus parainfluenzae T3T1, complete genome	gi|345428590|ref|NC_015964.1| Haemophilus parainfluenzae T3T1, complete genome
contig-100_2809	673	N	0	0	NA	0	0	0	NA	NA	NA	NA
contig-100_2810	673	N	0	0	NA	0	0	0	NA	NA	NA	NA
contig-100_2811	672	N	0	0	NA	0	0	0	NA	NA	NA	NA
contig-100_2812	672	N	0	0	NA	0	0	0	NA	NA	NA	NA
contig-100_2813	672	N	0	0	NA	0	0	0	NA	NA	NA	NA
contig-100_2814	672	N	0	0	NA	0	0	0	NA	NA	NA	NA
contig-100_2815	671	N	0	0	NA	0	0	0	NA	Clostridiales sp. SSC/2 draft genome	gi|479158859|ref|NC_021016.1| Butyrate-producing bacterium SSC/2, complete genome	gi|479158859|ref|NC_021016.1| Butyrate-producing bacterium SSC/2, complete genome
contig-100_2816	670	N	0	0	NA	0	0	0	NA	NA	NA	NA
contig-100_2817	670	N	0	0	NA	0	0	0	NA	NA	NA	NA
contig-100_2819	670	N	0	0	NA	0	0	0	NA	NA	NA	NA
contig-100_2821	670	N	0	0	NA	0	0	0	NA	NA	NA	NA
contig-100_2822	670	N	0	0	NA	0	0	0	NA	NA	NA	NA
contig-100_2823	670	N	0	0	NA	0	0	0	NA	NA	NA	NA
contig-100_2824	669	N	0	0	NA	0	0	0	NA	NA	NA	NA
contig-100_2826	669	N	1	0	NA	0	0	0	NA	NA	NA	NA

contig-100_2827	669	N	0	0	NA	0	0	0	NA	Uncultured organism clone VC1AM61TF genomic sequence	NA	Uncultured organism clone VC1AM61TF genomic sequence
contig-100_2828	669	N	0	0	NA	0	0	0	NA	NA	NA	NA
contig-100_2829	669	N	0	0	NA	0	0	0	NA	Uncultured bacterium clone LM0ABA1ZD05FM1 genomic sequence	NA	Uncultured bacterium clone LM0ABA1ZD05FM1 genomic sequence
contig-100_283	2749	N	2	0	NA	0	0	0	NA	Pseudomonas fluorescens SBW25 complete genome	gi|229587578|ref|NC_012660.1| Pseudomonas fluorescens SBW25 chromosome, complete genome	gi|229587578|ref|NC_012660.1| Pseudomonas fluorescens SBW25 chromosome, complete genome
contig-100_2830	669	N	1	0	NA	0	0	0	NA	NA	NA	NA
contig-100_2832	668	N	0	0	NA	0	0	0	NA	Bacteroides salanitronis DSM 18170, complete genome	gi|325297172|ref|NC_015164.1| Bacteroides salanitronis DSM 18170 chromosome, complete genome	gi|325297172|ref|NC_015164.1| Bacteroides salanitronis DSM 18170 chromosome, complete genome
contig-100_2834	668	N	0	0	NA	0	0	0	NA	NA	NA	NA
contig-100_2835	668	N	0	0	NA	0	0	0	NA	NA	NA	NA
contig-100_2836	668	N	1	0	NA	0	0	0	NA	Eubacterium rectale DSM 17629 draft genome	gi|479140210|ref|NC_021010.1| Eubacterium rectale DSM 17629 draft genome	gi|479140210|ref|NC_021010.1| Eubacterium rectale DSM 17629 draft genome
contig-100_2837	668	N	1	0	NA	0	0	0	NA	Haemophilus parainfluenzae T3T1 complete genome	gi|345428590|ref|NC_015964.1| Haemophilus parainfluenzae T3T1, complete genome	gi|345428590|ref|NC_015964.1| Haemophilus parainfluenzae T3T1, complete genome

contig-100_2838	668	N	0	0	NA	0	0	0	NA	Faecalibacterium prausnitzii L2/6 draft genome	gi|479208076|ref|NC_021042.1| Faecalibacterium prausnitzii L2-6, complete genome	gi|479208076|ref|NC_021042.1| Faecalibacterium prausnitzii L2-6, complete genome
contig-100_2839	668	N	0	0	NA	0	0	0	NA	NA	NA	NA
contig-100_284	2746	N	3	0	NA	0	0	0	NA	NA	NA	NA
contig-100_2840	667	N	0	0	NA	0	0	0	NA	Faecalibacterium prausnitzii L2/6 draft genome	gi|479208076|ref|NC_021042.1| Faecalibacterium prausnitzii L2-6, complete genome	gi|479208076|ref|NC_021042.1| Faecalibacterium prausnitzii L2-6, complete genome
contig-100_2841	667	N	0	0	NA	0	0	0	NA	NA	NA	NA
contig-100_2842	667	N	0	0	NA	0	0	0	NA	NA	NA	NA
contig-100_2843	667	N	1	0	NA	0	0	0	NA	NA	NA	NA
contig-100_2844	667	N	0	0	NA	0	0	0	NA	NA	NA	NA
contig-100_2846	666	N	0	0	NA	0	0	0	NA	NA	NA	NA
contig-100_2847	666	N	0	0	NA	0	0	0	NA	NA	NA	NA
contig-100_2848	666	N	0	0	NA	0	0	0	NA	NA	NA	NA
contig-100_2849	666	N	0	0	NA	0	0	0	NA	NA	NA	NA
contig-100_2850	666	N	0	0	NA	0	0	0	NA	NA	NA	NA
contig-100_2851	665	N	0	0	NA	0	0	0	NA	NA	NA	NA
contig-100_2852	665	N	1	0	NA	0	0	0	NA	NA	NA	NA
contig-100_2853	665	N	0	0	NA	0	0	0	NA	NA	NA	NA
contig-100_2854	665	N	1	0	NA	0	0	0	NA	Faecalibacterium prausnitzii L2/6 draft genome	gi|479208076|ref|NC_021042.1| Faecalibacterium prausnitzii L2-6, complete genome	gi|479208076|ref|NC_021042.1| Faecalibacterium prausnitzii L2-6, complete genome
contig-100_2855	664	N	0	0	NA	0	0	0	NA	NA	NA	NA
contig-100_2856	664	N	0	0	NA	0	0	0	NA	Haemophilus parainfluenzae T3T1 complete genome	gi|345428590|ref|NC_015964.1| Haemophilus parainfluenzae T3T1, complete genome	gi|345428590|ref|NC_015964.1| Haemophilus parainfluenzae T3T1, complete genome
contig-100_2857	664	N	0	0	NA	0	0	0	NA	NA	NA	NA

contig-100_2858	664	N	1	0	NA	0	0	0	NA	NA	NA	NA
contig-100_2859	664	N	1	0	NA	0	0	0	NA	Faecalibacterium prausnitzii L2/6 draft genome	gi|479208076|ref|NC_021042.1| Faecalibacterium prausnitzii L2-6, complete genome	gi|479208076|ref|NC_021042.1| Faecalibacterium prausnitzii L2-6, complete genome
contig-100_286	2741	N	2	1	Sipho	0	0	1	NA	NA	NA	NA
contig-100_2860	663	N	0	0	NA	0	0	0	NA	NA	NA	NA
contig-100_2861	663	N	1	0	NA	0	0	0	NA	NA	NA	NA
contig-100_2862	663	N	0	0	NA	0	0	0	NA	NA	NA	NA
contig-100_2863	663	N	0	0	NA	0	0	0	NA	NA	NA	NA
contig-100_2864	663	N	0	0	NA	0	0	0	NA	NA	NA	NA
contig-100_2865	663	N	0	0	NA	0	0	0	NA	Bacteroides thetaiotaomicron VPI-5482, complete genome	gi|29345410|ref|NC_004663.1| Bacteroides thetaiotaomicron VPI-5482 chromosome, complete genome	gi|29345410|ref|NC_004663.1| Bacteroides thetaiotaomicron VPI-5482 chromosome, complete genome
contig-100_2866	663	N	0	0	NA	0	0	0	NA	Haemophilus parainfluenzae T3T1 complete genome	gi|345428590|ref|NC_015964.1| Haemophilus parainfluenzae T3T1, complete genome	gi|345428590|ref|NC_015964.1| Haemophilus parainfluenzae T3T1, complete genome
contig-100_2867	663	N	0	0	NA	0	0	0	NA	NA	NA	NA
contig-100_2868	662	N	0	0	NA	0	0	0	NA	NA	NA	NA
contig-100_2869	662	N	0	0	NA	0	0	0	NA	Bacteroides thetaiotaomicron VPI-5482, complete genome	gi|29345410|ref|NC_004663.1| Bacteroides thetaiotaomicron VPI-5482 chromosome, complete genome	gi|29345410|ref|NC_004663.1| Bacteroides thetaiotaomicron VPI-5482 chromosome, complete genome

contig-100_2870	662	N	1	0	NA	0	0	0	NA	Faecalibacterium prausnitzii L2/6 draft genome	gi|479208076|ref|NC_021042.1| Faecalibacterium prausnitzii L2-6, complete genome	gi|479208076|ref|NC_021042.1| Faecalibacterium prausnitzii L2-6, complete genome
contig-100_2871	661	N	0	0	NA	0	0	0	NA	Eubacterium siraeum 70/3 draft genome	gi|479143419|ref|NC_021011.1| Eubacterium siraeum 70/3 draft genome	gi|479143419|ref|NC_021011.1| Eubacterium siraeum 70/3 draft genome
contig-100_2872	661	N	0	0	NA	0	0	0	NA	Bacteroides xylanisolvens XB1A draft genome	gi|479162165|ref|NC_021017.1| Bacteroides xylanisolvens XB1A draft genome	gi|479162165|ref|NC_021017.1| Bacteroides xylanisolvens XB1A draft genome
contig-100_2873	661	N	0	0	NA	0	0	0	NA	NA	NA	NA
contig-100_2874	661	N	0	0	NA	0	0	0	NA	NA	NA	NA
contig-100_2875	661	N	0	0	NA	0	0	0	NA	NA	NA	NA
contig-100_2876	661	N	0	0	NA	0	0	0	NA	NA	NA	NA
contig-100_2877	661	N	0	0	NA	0	0	0	NA	NA	NA	NA
contig-100_2878	660	N	1	0	NA	0	0	0	NA	NA	NA	NA
contig-100_2879	660	N	1	0	NA	0	0	0	NA	NA	NA	NA
contig-100_288	2717	N	2	2	Sipho	0	0	2	NA	NA	NA	NA
contig-100_2880	660	N	0	0	NA	0	0	0	NA	NA	NA	NA
contig-100_2881	660	N	0	0	NA	0	0	0	NA	NA	NA	NA
contig-100_2882	660	N	1	0	NA	0	0	0	NA	NA	NA	NA
contig-100_2883	659	N	0	0	NA	0	0	0	NA	NA	NA	NA
contig-100_2884	659	N	0	0	NA	0	0	0	NA	Veillonella parvula DSM 2008, complete genome	gi|269797069|ref|NC_013520.1| Veillonella parvula DSM 2008 chromosome, complete genome	gi|269797069|ref|NC_013520.1| Veillonella parvula DSM 2008 chromosome, complete genome
contig-100_2885	659	N	0	0	NA	0	0	0	NA	Eubacterium siraeum 70/3 draft genome	gi|479143419|ref|NC_021011.1| Eubacterium siraeum 70/3 draft genome	gi|479143419|ref|NC_021011.1| Eubacterium siraeum 70/3 draft genome

contig-100_2886	659	N	0	0	NA	0	0	0	NA	Bacteroides vulgatus ATCC 8482, complete genome	gi|150002608|ref|NC_009614.1| Bacteroides vulgatus ATCC 8482 chromosome, complete genome	gi|150002608|ref|NC_009614.1| Bacteroides vulgatus ATCC 8482 chromosome, complete genome
contig-100_2887	659	N	0	0	NA	0	0	0	NA	Acetobacter pasteurianus 386B, complete genome	gi|529230092|ref|NC_021991.1| Acetobacter pasteurianus 386B, complete genome	gi|529230092|ref|NC_021991.1| Acetobacter pasteurianus 386B, complete genome
contig-100_2888	659	N	0	0	NA	0	0	0	NA	NA	NA	NA
contig-100_2889	659	N	1	0	NA	0	0	0	NA	NA	NA	NA
contig-100_289	2708	N	2	0	NA	0	0	0	NA	Streptococcus phage SMP, complete genome	gi|251781468|ref|NC_012891.1| Streptococcus dysgalactiae subsp. equisimilis GGS_124 chromosome 1, complete sequence	gi|251781468|ref|NC_012891.1| Streptococcus dysgalactiae subsp. equisimilis GGS_124 chromosome 1, complete sequence
contig-100_2890	659	N	0	0	NA	0	0	0	NA	Uncultured bacterium clone LM0ABA3ZE07RM1 genomic sequence	NA	Uncultured bacterium clone LM0ABA3ZE07RM1 genomic sequence
contig-100_2891	659	N	0	0	NA	0	0	0	NA	NA	NA	NA
contig-100_2892	658	N	1	0	NA	0	0	0	NA	NA	NA	NA
contig-100_2893	658	N	0	0	NA	0	0	0	NA	Bacteroides salanitronis DSM 18170, complete genome	gi|325297172|ref|NC_015164.1| Bacteroides salanitronis DSM 18170 chromosome, complete genome	gi|325297172|ref|NC_015164.1| Bacteroides salanitronis DSM 18170 chromosome, complete genome
contig-100_2894	657	N	0	0	NA	0	0	0	NA	NA	NA	NA

contig-100_2895	657	N	0	0	NA	0	0	0	NA	Eubacterium siraeum 70/3 draft genome	gi|479143419|ref|NC_021011.1| Eubacterium siraeum 70/3 draft genome	gi|479143419|ref|NC_021011.1| Eubacterium siraeum 70/3 draft genome
contig-100_2896	657	N	0	0	NA	0	0	0	NA	Bacteroides xylanisolvens XB1A draft genome	gi|479162165|ref|NC_021017.1| Bacteroides xylanisolvens XB1A draft genome	gi|479162165|ref|NC_021017.1| Bacteroides xylanisolvens XB1A draft genome
contig-100_2897	657	N	0	0	NA	0	0	0	NA	NA	NA	NA
contig-100_2898	657	N	0	0	NA	0	0	0	NA	NA	NA	NA
contig-100_2899	657	N	0	0	NA	0	0	0	NA	NA	NA	NA
contig-100_290	2700	N	2	0	NA	0	0	0	NA	Bacteroides thetaiotaomicron VPI-5482, complete genome	gi|29345410|ref|NC_004663.1| Bacteroides thetaiotaomicron VPI-5482 chromosome, complete genome	gi|29345410|ref|NC_004663.1| Bacteroides thetaiotaomicron VPI-5482 chromosome, complete genome
contig-100_2900	657	N	0	0	NA	0	0	0	NA	NA	NA	NA
contig-100_2901	656	N	0	0	NA	0	0	0	NA	NA	NA	NA
contig-100_2903	655	N	0	0	NA	0	0	0	NA	NA	NA	NA
contig-100_2904	655	N	0	0	NA	0	0	0	NA	NA	NA	NA
contig-100_2905	655	N	0	0	NA	0	0	0	NA	Faecalibacterium prausnitzii SL3/3 draft genome	gi|479170689|ref|NC_021020.1| Faecalibacterium prausnitzii SL3/3 draft genome	gi|479170689|ref|NC_021020.1| Faecalibacterium prausnitzii SL3/3 draft genome
contig-100_2906	655	N	0	0	NA	0	0	0	NA	NA	NA	NA
contig-100_2907	655	N	1	0	NA	0	0	0	NA	NA	NA	NA
contig-100_2908	655	N	0	0	NA	0	0	0	NA	NA	NA	NA
contig-100_2909	655	N	2	0	NA	0	0	0	NA	NA	NA	NA
contig-100_2910	655	N	0	0	NA	0	0	0	NA	Clostridiales sp. SM4/1 draft genome	gi|479181986|ref|NC_021024.1| Butyrate-producing bacterium SM4/1, complete genome	gi|479181986|ref|NC_021024.1| Butyrate-producing bacterium SM4/1, complete genome
contig-100_2911	654	N	1	0	NA	0	0	0	NA	NA	NA	NA

contig-100_2912	654	N	2	0	NA	0	0	0	NA	NA	NA	NA
contig-100_2913	654	N	0	0	NA	0	0	0	NA	NA	NA	NA
contig-100_2914	654	N	1	0	NA	0	0	0	NA	Klebsiella pneumoniae JM45, complete genome	gi|530627845|ref|NC_022082.1| Klebsiella pneumoniae JM45, complete genome	gi|530627845|ref|NC_022082.1| Klebsiella pneumoniae JM45, complete genome
contig-100_2915	654	N	0	0	NA	0	0	0	NA	NA	NA	NA
contig-100_2916	653	N	0	0	NA	0	0	0	NA	NA	NA	NA
contig-100_2917	653	N	0	0	NA	0	0	0	NA	NA	NA	NA
contig-100_2918	653	N	0	0	NA	0	0	0	NA	NA	NA	NA
contig-100_2919	653	N	0	0	NA	0	0	0	NA	Uncultured organism clone 1041059767135 genomic sequence	NA	Uncultured organism clone 1041059767135 genomic sequence
contig-100_292	2693	N	2	1	Podo	0	0	1	NA	Uncultured bacterium EB1 genomic sequence	gi|479170689|ref|NC_021020.1| Faecalibacterium prausnitzii SL3/3 draft genome	gi|479170689|ref|NC_021020.1| Faecalibacterium prausnitzii SL3/3 draft genome
contig-100_2920	653	N	0	0	NA	0	0	0	NA	NA	NA	NA
contig-100_2922	653	N	0	0	NA	0	0	0	NA	Bacteroides vulgatus ATCC 8482, complete genome	gi|150002608|ref|NC_009614.1| Bacteroides vulgatus ATCC 8482 chromosome, complete genome	gi|150002608|ref|NC_009614.1| Bacteroides vulgatus ATCC 8482 chromosome, complete genome
contig-100_2923	652	N	0	0	NA	0	0	0	NA	NA	NA	NA
contig-100_2924	652	N	0	0	NA	0	0	0	NA	NA	NA	NA
contig-100_2925	652	N	0	0	NA	0	0	0	NA	NA	NA	NA
contig-100_2926	652	N	0	0	NA	0	0	0	NA	Clostridiales sp. SSC/2 draft genome	gi|479158859|ref|NC_021016.1| Butyrate-producing bacterium SSC/2, complete genome	gi|479158859|ref|NC_021016.1| Butyrate-producing bacterium SSC/2, complete genome
contig-100_2928	652	N	0	0	NA	0	0	0	NA	NA	NA	NA
contig-100_2929	652	N	0	0	NA	0	0	0	NA	NA	NA	NA

contig-100_2930	651	N	0	0	NA	0	0	0	NA	NA	NA	NA
contig-100_2931	650	N	0	0	NA	0	0	0	NA	Uncultured bacterium clone HA0AAA13ZE12RM1 genomic sequence	NA	Uncultured bacterium clone HA0AAA13ZE12RM1 genomic sequence
contig-100_2932	650	N	0	0	NA	0	0	0	NA	NA	NA	NA
contig-100_2934	650	N	0	0	NA	0	0	0	NA	NA	NA	NA
contig-100_2935	650	N	0	0	NA	0	0	0	NA	NA	NA	NA
contig-100_2936	649	N	0	0	NA	0	0	0	NA	NA	NA	NA
contig-100_2937	649	N	0	0	NA	0	0	0	NA	Bacteroides thetaiotaomicron VPI-5482, complete genome	gi|29345410|ref|NC_004663.1| Bacteroides thetaiotaomicron VPI-5482 chromosome, complete genome	gi|29345410|ref|NC_004663.1| Bacteroides thetaiotaomicron VPI-5482 chromosome, complete genome
contig-100_2938	649	N	0	0	NA	0	0	0	NA	Faecalibacterium prausnitzii L2/6 draft genome	gi|479208076|ref|NC_021042.1| Faecalibacterium prausnitzii L2-6, complete genome	gi|479208076|ref|NC_021042.1| Faecalibacterium prausnitzii L2-6, complete genome
contig-100_2939	649	N	0	0	NA	0	0	0	NA	NA	NA	NA
contig-100_294	2687	N	2	2	Sipho	0	0	2	NA	NA	NA	NA
contig-100_2940	649	N	0	0	NA	0	0	0	NA	Eubacterium rectale M104/1 draft genome	gi|479213596|ref|NC_021044.1| Eubacterium rectale M104/1 draft genome	gi|479213596|ref|NC_021044.1| Eubacterium rectale M104/1 draft genome
contig-100_2941	649	N	1	0	NA	0	0	0	NA	NA	NA	NA
contig-100_2942	648	N	1	0	NA	0	0	0	NA	Uncultured organism clone VC1C529TF genomic sequence	NA	Uncultured organism clone VC1C529TF genomic sequence
contig-100_2943	648	N	1	0	NA	0	0	0	NA	NA	NA	NA
contig-100_2944	648	N	0	0	NA	0	0	0	NA	NA	NA	NA

contig-100_2945	648	N	0	0	NA	0	0	0	NA	Faecalibacterium prausnitzii L2/6 draft genome	gi|479208076|ref|NC_021042.1| Faecalibacterium prausnitzii L2-6, complete genome	gi|479208076|ref|NC_021042.1| Faecalibacterium prausnitzii L2-6, complete genome
contig-100_2947	647	N	0	0	NA	0	0	0	NA	Haemophilus influenzae KR494, complete genome	gi|543951066|ref|NC_022356.1| Haemophilus influenzae KR494, complete genome	gi|543951066|ref|NC_022356.1| Haemophilus influenzae KR494, complete genome
contig-100_2948	647	N	0	0	NA	0	0	0	NA	NA	NA	NA
contig-100_295	2678	N	2	1	Sipho	1	0	2	NA	NA	NA	NA
contig-100_2950	647	N	0	0	NA	0	0	0	NA	NA	NA	NA
contig-100_2951	647	N	0	0	NA	0	0	0	NA	Faecalibacterium prausnitzii L2/6 draft genome	gi|479208076|ref|NC_021042.1| Faecalibacterium prausnitzii L2-6, complete genome	gi|479208076|ref|NC_021042.1| Faecalibacterium prausnitzii L2-6, complete genome
contig-100_2952	647	N	0	0	NA	0	0	0	NA	NA	NA	NA
contig-100_2953	647	N	0	0	NA	0	0	0	NA	Haemophilus influenzae 10810 genome	gi|378696079|ref|NC_016809.1| Haemophilus influenzae 10810, complete genome	gi|378696079|ref|NC_016809.1| Haemophilus influenzae 10810, complete genome
contig-100_2954	647	N	2	0	NA	0	0	0	NA	Faecalibacterium prausnitzii L2/6 draft genome	gi|479208076|ref|NC_021042.1| Faecalibacterium prausnitzii L2-6, complete genome	gi|479208076|ref|NC_021042.1| Faecalibacterium prausnitzii L2-6, complete genome
contig-100_2955	647	N	0	0	NA	0	0	0	NA	Roseburia hominis A2-183, complete genome	gi|479146200|ref|NC_021012.1| Roseburia intestinalis XB6B4 draft genome	gi|479146200|ref|NC_021012.1| Roseburia intestinalis XB6B4 draft genome

contig-100_2956	646	N	1	0	NA	0	0	0	NA	NA	NA	NA
contig-100_2957	646	N	0	0	NA	0	0	0	NA	NA	NA	NA
contig-100_2958	646	N	1	0	NA	0	0	0	NA	NA	NA	NA
contig-100_2959	646	N	0	0	NA	0	0	0	NA	NA	NA	NA
contig-100_296	2677	N	2	0	NA	0	0	0	NA	NA	NA	NA
contig-100_2960	646	N	0	0	NA	0	0	0	NA	NA	NA	NA
contig-100_2961	646	N	0	0	NA	0	0	0	NA	NA	NA	NA
contig-100_2962	646	N	0	0	NA	0	0	0	NA	Bacteroides vulgatus ATCC 8482, complete genome	gi|150002608|ref|NC_009614.1| Bacteroides vulgatus ATCC 8482 chromosome, complete genome	gi|150002608|ref|NC_009614.1| Bacteroides vulgatus ATCC 8482 chromosome, complete genome
contig-100_2963	645	N	2	0	NA	0	0	0	NA	NA	NA	NA
contig-100_2964	645	N	1	0	NA	0	0	0	NA	NA	NA	NA
contig-100_2965	645	N	1	0	NA	0	0	0	NA	NA	NA	NA
contig-100_2966	645	N	0	0	NA	0	0	0	NA	Bacteroides helcogenes P 36-108, complete genome	gi|319899888|ref|NC_014933.1| Bacteroides helcogenes P 36-108 chromosome, complete genome	gi|319899888|ref|NC_014933.1| Bacteroides helcogenes P 36-108 chromosome, complete genome
contig-100_2967	645	N	0	0	NA	0	0	0	NA	NA	NA	NA
contig-100_2968	645	N	0	0	NA	0	0	0	NA	NA	NA	NA
contig-100_2969	645	N	0	0	NA	0	0	0	NA	NA	NA	NA
contig-100_297	2675	N	4	0	NA	0	0	0	NA	Klebsiella aerogenes nitrite reductase subunit B (nirB) gene, partial cds; and nitrite reductase subunit D (nirD), siroheme synthase (cysG), tryptophanyl-tRNA synthetase (trpS), and Gph (gph) genes, complete cds	gi|550443072|ref|NC_022566.1| Klebsiella pneumoniae CG43, complete genome	gi|550443072|ref|NC_022566.1| Klebsiella pneumoniae CG43, complete genome
contig-100_2970	644	N	0	0	NA	0	0	0	NA	Lactobacillus reuteri TD1, complete genome	gi|526230725|ref|NC_021872.1| Lactobacillus reuteri TD1, complete genome	gi|526230725|ref|NC_021872.1| Lactobacillus reuteri TD1, complete genome

contig-100_2972	644	N	0	0	NA	0	0	0	NA	Bacteroides thetaiotaomicron VPI-5482, complete genome	gi|29345410|ref|NC_004663.1| Bacteroides thetaiotaomicron VPI-5482 chromosome, complete genome	gi|29345410|ref|NC_004663.1| Bacteroides thetaiotaomicron VPI-5482 chromosome, complete genome
contig-100_2973	644	N	0	0	NA	0	0	0	NA	Bacteroides thetaiotaomicron VPI-5482, complete genome	gi|29345410|ref|NC_004663.1| Bacteroides thetaiotaomicron VPI-5482 chromosome, complete genome	gi|29345410|ref|NC_004663.1| Bacteroides thetaiotaomicron VPI-5482 chromosome, complete genome
contig-100_2974	644	N	0	0	NA	0	0	0	NA	NA	NA	NA
contig-100_2975	644	N	0	0	NA	0	0	0	NA	NA	NA	NA
contig-100_2976	643	N	0	0	NA	0	0	0	NA	Uncultured organism clone VC1CV74TR genomic sequence	NA	Uncultured organism clone VC1CV74TR genomic sequence
contig-100_2977	643	N	1	0	NA	0	0	0	NA	NA	NA	NA
contig-100_2978	643	N	0	0	NA	0	0	0	NA	Parabacteroides distasonis ATCC 8503, complete genome	gi|150006674|ref|NC_009615.1| Parabacteroides distasonis ATCC 8503 chromosome, complete genome	gi|150006674|ref|NC_009615.1| Parabacteroides distasonis ATCC 8503 chromosome, complete genome
contig-100_2979	642	N	0	0	NA	0	0	0	NA	NA	NA	NA
contig-100_298	2671	N	4	0	NA	0	0	0	NA	Uncultured organism clone VC1AL90TR genomic sequence	NA	Uncultured organism clone VC1AL90TR genomic sequence
contig-100_2982	642	N	0	0	NA	0	0	0	NA	Uncultured organism clone VC1BT04TF genomic sequence	NA	Uncultured organism clone VC1BT04TF genomic sequence

contig-100_2983	642	N	0	0	NA	0	0	0	NA	Unidentified phage clone 2020_scaffold1264 genomic sequence	NA	Unidentified phage clone 2020_scaffold 1264 genomic sequence
contig-100_2984	642	N	0	0	NA	0	0	0	NA	Roseburia hominis A2-183, complete genome	gi|347530298|ref|NC_015977.1| Roseburia hominis A2-183 chromosome, complete genome	gi|347530298|ref|NC_015977.1| Roseburia hominis A2-183 chromosome, complete genome
contig-100_2985	642	N	2	0	NA	0	0	0	NA	Uncultured organism clone 1041059765674 genomic sequence	gi|479162165|ref|NC_021017.1| Bacteroides xylanisolvens XB1A draft genome	gi|479162165|ref|NC_021017.1| Bacteroides xylanisolvens XB1A draft genome
contig-100_2986	641	N	0	0	NA	0	0	0	NA	NA	NA	NA
contig-100_2988	641	N	1	0	NA	0	0	0	NA	NA	NA	NA
contig-100_2989	641	N	1	0	NA	0	0	0	NA	NA	NA	NA
contig-100_2990	641	N	0	0	NA	0	0	0	NA	Clostridium saccharolyticum WM1, complete genome	gi|302384444|ref|NC_014376.1| Clostridium saccharolyticum WM1 chromosome, complete genome	gi|302384444|ref|NC_014376.1| Clostridium saccharolyticum WM1 chromosome, complete genome
contig-100_2992	640	N	1	0	NA	0	0	0	NA	Bacteroides vulgatus ATCC 8482, complete genome	gi|150002608|ref|NC_009614.1| Bacteroides vulgatus ATCC 8482 chromosome, complete genome	gi|150002608|ref|NC_009614.1| Bacteroides vulgatus ATCC 8482 chromosome, complete genome
contig-100_2993	640	N	0	0	NA	0	0	0	NA	NA	NA	NA
contig-100_2994	640	N	0	0	NA	0	0	0	NA	NA	NA	NA
contig-100_2995	640	N	1	0	NA	0	0	0	NA	NA	NA	NA
contig-100_2996	640	N	0	0	NA	0	0	0	NA	NA	NA	NA
contig-100_2997	640	N	1	0	NA	0	0	0	NA	NA	NA	NA
contig-100_2998	640	N	0	0	NA	0	0	0	NA	NA	NA	NA
contig-100_2999	640	N	0	0	NA	0	0	0	NA	NA	NA	NA

contig-100_3000	640	N	0	0	NA	0	0	0	NA	Bacteroides vulgatus ATCC 8482, complete genome	gi|150002608|ref|NC_009614.1| Bacteroides vulgatus ATCC 8482 chromosome, complete genome	gi|150002608|ref|NC_009614.1| Bacteroides vulgatus ATCC 8482 chromosome, complete genome
contig-100_3001	640	N	2	0	NA	0	0	0	NA	NA	NA	NA
contig-100_3002	639	N	0	0	NA	0	0	0	NA	NA	NA	NA
contig-100_3003	639	N	1	0	NA	0	0	0	NA	Faecalibacterium prausnitzii L2/6 draft genome	gi|479208076|ref|NC_021042.1| Faecalibacterium prausnitzii L2-6, complete genome	gi|479208076|ref|NC_021042.1| Faecalibacterium prausnitzii L2-6, complete genome
contig-100_3004	639	N	0	0	NA	0	0	0	NA	Uncultured organism clone VC1A060TF genomic sequence	gi|150002608|ref|NC_009614.1| Bacteroides vulgatus ATCC 8482 chromosome, complete genome	gi|150002608|ref|NC_009614.1| Bacteroides vulgatus ATCC 8482 chromosome, complete genome
contig-100_3005	639	N	0	0	NA	0	0	0	NA	NA	NA	NA
contig-100_3006	639	N	0	0	NA	0	0	0	NA	Klebsiella pneumoniae JM45, complete genome	gi|550443072|ref|NC_022566.1| Klebsiella pneumoniae CG43, complete genome	gi|550443072|ref|NC_022566.1| Klebsiella pneumoniae CG43, complete genome
contig-100_3007	639	N	0	0	NA	0	0	0	NA	NA	NA	NA
contig-100_3009	638	N	0	0	NA	0	0	0	NA	NA	NA	NA
contig-100_3010	638	N	0	0	NA	0	0	0	NA	NA	NA	NA
contig-100_3011	638	N	0	0	NA	0	0	0	NA	Eubacterium eligens ATCC 27750, complete genome	gi|238915976|ref|NC_012778.1| Eubacterium eligens ATCC 27750 chromosome, complete genome	gi|238915976|ref|NC_012778.1| Eubacterium eligens ATCC 27750 chromosome, complete genome
contig-100_3015	638	N	0	0	NA	0	0	0	NA	NA	NA	NA

contig-100_3016	638	N	0	0	NA	0	0	0	NA	Bacteroides vulgatus ATCC 8482, complete genome	gi|150002608|ref|NC_009614.1| Bacteroides vulgatus ATCC 8482 chromosome, complete genome	gi|150002608|ref|NC_009614.1| Bacteroides vulgatus ATCC 8482 chromosome, complete genome
contig-100_3017	638	N	0	0	NA	0	0	0	NA	NA	NA	NA
contig-100_3018	638	N	0	0	NA	0	0	0	NA	Lactobacillus paracasei subsp. paracasei JCM 8130 DNA, complete genome	gi|523512490|ref|NC_021721.1| Lactobacillus casei LOCK919, complete genome	gi|523512490|ref|NC_021721.1| Lactobacillus casei LOCK919, complete genome
contig-100_3019	637	N	0	0	NA	0	0	0	NA	NA	NA	NA
contig-100_302	2650	N	2	2	Sipho	1	0	2	NA	NA	NA	NA
contig-100_3020	637	N	0	0	NA	0	0	0	NA	Clostridium sp. SY8519 DNA, complete genome	gi|339441064|ref|NC_015737.1| Clostridium sp. SY8519, complete genome	gi|339441064|ref|NC_015737.1| Clostridium sp. SY8519, complete genome
contig-100_3021	637	N	0	0	NA	0	0	0	NA	Faecalibacterium prausnitzii L2/6 draft genome	gi|479208076|ref|NC_021042.1| Faecalibacterium prausnitzii L2-6, complete genome	gi|479208076|ref|NC_021042.1| Faecalibacterium prausnitzii L2-6, complete genome
contig-100_3022	637	N	0	0	NA	0	0	0	NA	Bacteroides vulgatus ATCC 8482, complete genome	gi|150002608|ref|NC_009614.1| Bacteroides vulgatus ATCC 8482 chromosome, complete genome	gi|150002608|ref|NC_009614.1| Bacteroides vulgatus ATCC 8482 chromosome, complete genome
contig-100_3023	637	N	1	0	NA	0	0	0	NA	Eubacterium rectale M104/1 draft genome	gi|479213596|ref|NC_021044.1| Eubacterium rectale M104/1 draft genome	gi|479213596|ref|NC_021044.1| Eubacterium rectale M104/1 draft genome

contig-100_3024	636	N	1	0	NA	0	0	0	NA	NA	NA	NA
contig-100_3025	636	N	0	0	NA	0	0	0	NA	Bacteroides fragilis 638R genome	gi|60679597|ref|NC_003228.3| Bacteroides fragilis NCTC 9343, complete genome	gi|60679597|ref|NC_003228.3| Bacteroides fragilis NCTC 9343, complete genome
contig-100_3026	636	N	1	0	NA	0	0	0	NA	NA	NA	NA
contig-100_3027	636	N	0	0	NA	0	0	0	NA	NA	NA	NA
contig-100_3028	636	N	0	0	NA	0	0	0	NA	NA	NA	NA
contig-100_3029	636	N	0	0	NA	0	0	0	NA	NA	NA	NA
contig-100_303	2632	N	2	1	Myo	0	0	1	NA	NA	NA	NA
contig-100_3030	636	N	1	0	NA	0	0	0	NA	NA	NA	NA
contig-100_3031	635	N	0	0	NA	0	0	0	NA	Uncultured organism clone 1041059765007 genomic sequence	gi|319899888|ref|NC_014933.1| Bacteroides helcogenes P 36-108 chromosome, complete genome	gi|319899888|ref|NC_014933.1| Bacteroides helcogenes P 36-108 chromosome, complete genome
contig-100_3032	635	N	1	0	NA	0	0	0	NA	NA	NA	NA
contig-100_3034	635	N	0	0	NA	0	0	0	NA	NA	NA	NA
contig-100_3035	635	N	0	0	NA	0	0	0	NA	NA	NA	NA
contig-100_3036	635	N	1	0	NA	0	0	0	NA	NA	NA	NA
contig-100_3037	635	N	0	0	NA	0	0	0	NA	NA	NA	NA
contig-100_3038	635	N	0	0	NA	0	0	0	NA	NA	NA	NA
contig-100_304	2627	N	3	1	Pox	0	1	1	NA	NA	NA	NA
contig-100_3040	634	N	0	0	NA	0	0	0	NA	Haemophilus parainfluenzae T3T1 complete genome	gi|345428590|ref|NC_015964.1| Haemophilus parainfluenzae T3T1, complete genome	gi|345428590|ref|NC_015964.1| Haemophilus parainfluenzae T3T1, complete genome
contig-100_3041	634	N	0	0	NA	0	0	0	NA	Clostridiales sp. SSC/2 draft genome	gi|479158859|ref|NC_021016.1| Butyrate-producing bacterium SSC/2, complete genome	gi|479158859|ref|NC_021016.1| Butyrate-producing bacterium SSC/2, complete genome

contig-100_3042	634	N	0	0	NA	0	0	0	NA	Uncultured organism clone 1041059767023 genomic sequence	NA	Uncultured organism clone 104105976702 3 genomic sequence
contig-100_3043	634	N	0	0	NA	0	0	0	NA	NA	NA	NA
contig-100_3044	634	N	0	0	NA	0	0	0	NA	NA	NA	NA
contig-100_3045	634	N	0	0	NA	0	0	0	NA	Parabacteroides distasonis ATCC 8503, complete genome	gi|150006674|ref|N C_009615.1| Parabacteroides distasonis ATCC 8503 chromosome, complete genome	gi|150006674|r ef|NC_009615 .1| Parabacteroide s distasonis ATCC 8503 chromosome, complete genome
contig-100_3046	634	N	1	0	NA	0	0	0	NA	Unidentified phage clone 2019_scaffold132 genomic sequence	NA	Unidentified phage clone 2019_scaffold 132 genomic sequence
contig-100_3047	634	N	0	0	NA	0	0	0	NA	NA	NA	NA
contig-100_3048	634	N	1	0	NA	0	0	0	NA	NA	NA	NA
contig-100_3049	633	N	1	0	NA	0	0	0	NA	NA	NA	NA
contig-100_305	2624	N	2	1	Sipho	0	0	1	NA	NA	NA	NA
contig-100_3051	633	N	0	0	NA	0	0	0	NA	NA	NA	NA
contig-100_3053	633	N	1	0	NA	0	0	0	NA	Bacteroides xylanisolvens XB1A draft genome	gi|479162165|ref|N C_021017.1| Bacteroides xylanisolvens XB1A draft genome	gi|479162165|r ef|NC_021017 .1| Bacteroides xylanisolvens XB1A draft genome
contig-100_3054	633	N	1	0	NA	0	0	0	NA	NA	NA	NA
contig-100_3055	633	N	1	0	NA	0	0	0	NA	NA	NA	NA
contig-100_3056	633	N	0	0	NA	0	0	0	NA	NA	NA	NA
contig-100_3057	632	N	2	0	NA	0	0	0	NA	NA	NA	NA
contig-100_3058	632	N	0	0	NA	0	0	0	NA	Haemophilus parainfluenzae T3T1 complete genome	gi|345428590|ref|N C_015964.1| Haemophilus parainfluenzae T3T1, complete genome	gi|345428590|r ef|NC_015964 .1| Haemophilus parainfluenzae T3T1, complete genome
contig-100_3059	632	N	0	0	NA	0	0	0	NA	NA	NA	NA

contig-100_3060	632	N	0	0	NA	0	0	0	NA	Haemophilus parainfluenzae T3T1 complete genome	gi|345428590|ref|NC_015964.1| Haemophilus parainfluenzae T3T1, complete genome	gi|345428590|ref|NC_015964.1| Haemophilus parainfluenzae T3T1, complete genome
contig-100_3061	632	N	1	0	NA	0	0	0	NA	Faecalibacterium prausnitzii L2/6 draft genome	gi|479208076|ref|NC_021042.1| Faecalibacterium prausnitzii L2-6, complete genome	gi|479208076|ref|NC_021042.1| Faecalibacterium prausnitzii L2-6, complete genome
contig-100_3062	632	N	0	0	NA	0	0	0	NA	Ruminococcus obeum A2-162 draft genome	gi|479176048|ref|NC_021022.1| Ruminococcus obeum A2-162 draft genome	gi|479176048|ref|NC_021022.1| Ruminococcus obeum A2-162 draft genome
contig-100_3064	632	N	0	0	NA	0	0	0	NA	NA	NA	NA
contig-100_3066	631	N	0	0	NA	0	0	0	NA	Clostridium saccharolyticum-like K10 draft genome	gi|479336697|ref|NC_021047.1| Clostridium cf. saccharolyticum K10, complete genome	gi|479336697|ref|NC_021047.1| Clostridium cf. saccharolyticum K10, complete genome
contig-100_3067	631	N	1	0	NA	0	0	0	NA	NA	NA	NA
contig-100_3068	631	N	0	0	NA	0	0	0	NA	NA	NA	NA
contig-100_3069	631	N	0	0	NA	0	0	0	NA	NA	NA	NA
contig-100_307	2595	N	0	0	NA	0	0	0	NA	Enterobacter agglomerans ColE1-like plasmid RNA one modulator (rom) gene, complete cds	gi|449306421|ref|NC_020262.1| Cronobacter sakazakii Sp291 plasmid pSP291-3, complete sequence	gi|449306421|ref|NC_020262.1| Cronobacter sakazakii Sp291 plasmid pSP291-3, complete sequence

contig-100_3070	630	N	1	0	NA	0	0	0	NA	Faecalibacterium prausnitzii L2/6 draft genome	gi|479208076|ref|NC_021042.1| Faecalibacterium prausnitzii L2-6, complete genome	gi|479208076|ref|NC_021042.1| Faecalibacterium prausnitzii L2-6, complete genome
contig-100_3071	630	N	0	0	NA	0	0	0	NA	Parabacteroides distasonis ATCC 8503, complete genome	gi|150006674|ref|NC_009615.1| Parabacteroides distasonis ATCC 8503 chromosome, complete genome	gi|150006674|ref|NC_009615.1| Parabacteroides distasonis ATCC 8503 chromosome, complete genome
contig-100_3072	630	N	0	0	NA	0	0	0	NA	NA	NA	NA
contig-100_3073	630	N	0	0	NA	0	0	0	NA	NA	NA	NA
contig-100_3074	629	N	0	0	NA	0	0	0	NA	NA	NA	NA
contig-100_3075	629	N	0	0	NA	0	0	0	NA	NA	NA	NA
contig-100_3076	629	N	1	0	NA	0	0	0	NA	NA	NA	NA
contig-100_3077	629	N	0	0	NA	0	0	0	NA	Unidentified phage clone 2020_scaffold1264 genomic sequence	NA	Unidentified phage clone 2020_scaffold1264 genomic sequence
contig-100_3078	629	N	0	0	NA	0	0	0	NA	NA	NA	NA
contig-100_3079	629	N	0	0	NA	0	0	0	NA	NA	NA	NA
contig-100_308	2589	N	1	0	NA	0	0	0	NA	NA	NA	NA
contig-100_3080	629	N	0	0	NA	0	0	0	NA	Bacteroides vulgatus ATCC 8482, complete genome	gi|150002608|ref|NC_009614.1| Bacteroides vulgatus ATCC 8482 chromosome, complete genome	gi|150002608|ref|NC_009614.1| Bacteroides vulgatus ATCC 8482 chromosome, complete genome
contig-100_3083	629	N	0	0	NA	0	0	0	NA	NA	NA	NA
contig-100_3084	629	N	0	0	NA	0	0	0	NA	Faecalibacterium prausnitzii L2/6 draft genome	gi|479208076|ref|NC_021042.1| Faecalibacterium prausnitzii L2-6, complete genome	gi|479208076|ref|NC_021042.1| Faecalibacterium prausnitzii L2-6, complete genome

contig-100_3085	628	N	0	0	NA	0	0	0	NA	Ruminococcus obeum A2-162 draft genome	gi|479176048|ref|NC_021022.1| Ruminococcus obeum A2-162 draft genome	gi|479176048|ref|NC_021022.1| Ruminococcus obeum A2-162 draft genome
contig-100_3087	628	N	0	0	NA	0	0	0	NA	Uncultured organism clone VC1CB79TF genomic sequence	NA	Uncultured organism clone VC1CB79TF genomic sequence
contig-100_3088	628	N	0	0	NA	0	0	0	NA	NA	NA	NA
contig-100_3089	628	N	1	0	NA	0	0	0	NA	NA	NA	NA
contig-100_309	2587	N	2	0	NA	0	0	1	NA	NA	NA	NA
contig-100_3090	628	N	0	0	NA	0	0	0	NA	NA	NA	NA
contig-100_3092	627	N	1	0	NA	0	0	0	NA	NA	NA	NA
contig-100_3093	627	N	0	0	NA	0	0	0	NA	Clostridium saccharolyticum-like K10 draft genome	gi|479336697|ref|NC_021047.1| Clostridium cf. saccharolyticum K10, complete genome	gi|479336697|ref|NC_021047.1| Clostridium cf. saccharolyticum K10, complete genome
contig-100_3094	627	N	1	0	NA	0	0	0	NA	Uncultured bacterium clone HA0AAA3ZB10RM1 genomic sequence	NA	Uncultured bacterium clone HA0AAA3ZB10RM1 genomic sequence
contig-100_3095	626	N	1	0	NA	0	0	0	NA	NA	NA	NA
contig-100_3096	626	N	0	0	NA	0	0	0	NA	NA	NA	NA
contig-100_3098	626	N	0	0	NA	0	0	0	NA	Uncultured organism clone 1041059765561 genomic sequence	gi|60650141|ref|NC_006873.1| Bacteroides fragilis NCTC 9343 plasmid pBF9343, complete sequence	gi|60650141|ref|NC_006873.1| Bacteroides fragilis NCTC 9343 plasmid pBF9343, complete sequence
contig-100_3099	625	N	0	0	NA	0	0	0	NA	NA	NA	NA
contig-100_310	2587	N	2	0	NA	0	0	0	NA	NA	NA	NA

contig-100_3100	625	N	0	0	NA	0	0	0	NA	Haemophilus parainfluenzae T3T1 complete genome	gi|345428590|ref|NC_015964.1| Haemophilus parainfluenzae T3T1, complete genome	gi|345428590|ref|NC_015964.1| Haemophilus parainfluenzae T3T1, complete genome
contig-100_3101	625	N	0	0	NA	0	0	0	NA	NA	NA	NA
contig-100_3102	625	N	0	0	NA	0	0	0	NA	NA	NA	NA
contig-100_3103	625	N	0	0	NA	0	0	0	NA	NA	NA	NA
contig-100_3104	624	N	1	0	NA	0	0	0	NA	Uncultured organism clone VC1DB64TF genomic sequence	NA	Uncultured organism clone VC1DB64TF genomic sequence
contig-100_3105	624	N	0	0	NA	0	0	0	NA	NA	NA	NA
contig-100_3106	624	N	0	0	NA	0	0	0	NA	Clostridiales sp. SS3/4 draft genome	gi|479192860|ref|NC_021035.1| Butyrate-producing bacterium SS3/4, complete genome	gi|479192860|ref|NC_021035.1| Butyrate-producing bacterium SS3/4, complete genome
contig-100_3107	624	N	0	0	NA	0	0	0	NA	Faecalibacterium prausnitzii L2/6 draft genome	gi|479208076|ref|NC_021042.1| Faecalibacterium prausnitzii L2-6, complete genome	gi|479208076|ref|NC_021042.1| Faecalibacterium prausnitzii L2-6, complete genome
contig-100_3108	624	N	0	0	NA	0	0	0	NA	NA	NA	NA
contig-100_3109	624	N	0	0	NA	0	0	0	NA	Propionibacterium acnes ATCC 11828, complete genome	gi|386069650|ref|NC_017550.1| Propionibacterium acnes ATCC 11828 chromosome, complete genome	gi|386069650|ref|NC_017550.1| Propionibacterium acnes ATCC 11828 chromosome, complete genome
contig-100_311	2579	N	1	1	Sipho	0	0	1	NA	NA	NA	NA
contig-100_3110	624	N	0	0	NA	0	0	0	NA	NA	NA	NA
contig-100_3111	624	N	0	0	NA	0	0	0	NA	NA	NA	NA
contig-100_3112	624	N	0	0	NA	0	0	0	NA	NA	NA	NA
contig-100_3113	624	N	1	0	NA	0	0	0	NA	NA	NA	NA

contig-100_3114	623	N	0	0	NA	0	0	0	NA	NA	NA	NA
contig-100_3115	623	N	0	0	NA	0	0	0	NA	NA	NA	NA
contig-100_3116	623	N	0	0	NA	0	0	0	NA	NA	NA	NA
contig-100_3117	623	N	0	0	NA	0	0	0	NA	NA	NA	NA
contig-100_3118	623	N	0	0	NA	0	0	0	NA	Acidovorax citrulli AAC00-1, complete genome	gi|120608714|ref|NC_008752.1| Acidovorax citrulli AAC00-1 chromosome, complete genome	gi|120608714|ref|NC_008752.1| Acidovorax citrulli AAC00-1 chromosome, complete genome
contig-100_3119	623	N	0	0	NA	0	0	0	NA	Bacteroides vulgatus ATCC 8482, complete genome	gi|150002608|ref|NC_009614.1| Bacteroides vulgatus ATCC 8482 chromosome, complete genome	gi|150002608|ref|NC_009614.1| Bacteroides vulgatus ATCC 8482 chromosome, complete genome
contig-100_3120	623	N	0	0	NA	0	0	0	NA	NA	NA	NA
contig-100_3121	623	N	0	0	NA	0	0	0	NA	NA	NA	NA
contig-100_3122	623	N	1	0	NA	0	0	0	NA	Bacteroides vulgatus ATCC 8482, complete genome	gi|150002608|ref|NC_009614.1| Bacteroides vulgatus ATCC 8482 chromosome, complete genome	gi|150002608|ref|NC_009614.1| Bacteroides vulgatus ATCC 8482 chromosome, complete genome
contig-100_3123	623	N	0	0	NA	0	0	0	NA	NA	NA	NA
contig-100_3124	622	N	1	0	NA	0	0	0	NA	NA	NA	NA
contig-100_3125	622	N	0	0	NA	0	0	0	NA	NA	NA	NA
contig-100_3126	622	N	0	0	NA	0	0	0	NA	NA	NA	NA
contig-100_3127	622	N	1	0	NA	0	0	0	NA	NA	NA	NA
contig-100_3128	622	N	0	0	NA	0	0	0	NA	NA	NA	NA
contig-100_3129	622	N	0	0	NA	0	0	0	NA	Bifidobacterium bifidum PRL2010, complete genome	gi|311063459|ref|NC_014638.1| Bifidobacterium bifidum PRL2010 chromosome, complete genome	gi|311063459|ref|NC_014638.1| Bifidobacterium bifidum PRL2010 chromosome, complete genome
contig-100_313	2568	N	3	0	NA	0	0	1	NA	NA	NA	NA

contig-100_3130	622	N	0	0	NA	0	0	0	NA	Dendroctonus ponderosae Seq01005302, whole genome shotgun sequence	NA	Dendroctonus ponderosae Seq01005302, whole genome shotgun sequence
contig-100_3131	622	N	0	0	NA	0	0	0	NA	Bacteroides salanitronis DSM 18170, complete genome	gi|325297172|ref|NC_015164.1| Bacteroides salanitronis DSM 18170 chromosome, complete genome	gi|325297172|ref|NC_015164.1| Bacteroides salanitronis DSM 18170 chromosome, complete genome
contig-100_3132	622	N	0	0	NA	0	0	0	NA	NA	NA	NA
contig-100_3133	621	N	1	0	NA	0	0	0	NA	Bacteroides vulgatus ATCC 8482, complete genome	gi|150002608|ref|NC_009614.1| Bacteroides vulgatus ATCC 8482 chromosome, complete genome	gi|150002608|ref|NC_009614.1| Bacteroides vulgatus ATCC 8482 chromosome, complete genome
contig-100_3134	621	N	0	0	NA	0	0	0	NA	Clostridiales sp. SM4/1 draft genome	gi|479181986|ref|NC_021024.1| Butyrate-producing bacterium SM4/1, complete genome	gi|479181986|ref|NC_021024.1| Butyrate-producing bacterium SM4/1, complete genome
contig-100_3135	621	N	1	0	NA	0	0	0	NA	Faecalibacterium prausnitzii SL3/3 draft genome	gi|479170689|ref|NC_021020.1| Faecalibacterium prausnitzii SL3/3 draft genome	gi|479170689|ref|NC_021020.1| Faecalibacterium prausnitzii SL3/3 draft genome
contig-100_3136	620	N	0	0	NA	0	0	0	NA	NA	NA	NA
contig-100_3137	620	N	0	0	NA	0	0	0	NA	NA	NA	NA
contig-100_3138	620	N	0	0	NA	0	0	0	NA	NA	NA	NA
contig-100_3139	620	N	0	0	NA	0	0	0	NA	NA	NA	NA
contig-100_314	2566	N	1	1	Sipho	0	0	1	NA	NA	NA	NA
contig-100_3140	619	N	0	0	NA	0	0	0	NA	NA	NA	NA
contig-100_3141	619	N	0	0	NA	0	0	0	NA	NA	NA	NA
contig-100_3142	619	N	0	0	NA	0	0	0	NA	NA	NA	NA
contig-100_3143	619	N	0	0	NA	0	0	0	NA	NA	NA	NA

contig-100_3144	619	N	0	0	NA	0	0	0	NA	NA	NA	NA
contig-100_3145	619	N	0	0	NA	0	0	0	NA	NA	NA	NA
contig-100_3146	618	N	0	0	NA	0	0	0	NA	NA	NA	NA
contig-100_3147	618	N	0	0	NA	0	0	0	NA	NA	NA	NA
contig-100_3148	618	N	1	0	NA	0	0	0	NA	NA	NA	NA
contig-100_3149	618	N	0	0	NA	0	0	0	NA	NA	NA	NA
contig-100_315	2564	N	1	0	NA	0	0	0	NA	NA	NA	NA
contig-100_3151	618	N	0	0	NA	0	0	0	NA	NA	NA	NA
contig-100_3152	618	N	0	0	NA	0	0	0	NA	NA	NA	NA
contig-100_3153	618	N	1	0	NA	0	0	0	NA	NA	NA	NA
contig-100_3154	617	N	0	0	NA	0	0	0	NA	NA	NA	NA
contig-100_3155	617	N	0	0	NA	0	0	0	NA	NA	NA	NA
contig-100_3156	617	N	1	0	NA	0	0	0	NA	TPA_exp: Clostridium difficile strain QCD-66C26 transposon Tn6110, complete sequence	gi|260685375|ref|NC_013316.1| Clostridium difficile R20291 chromosome, complete genome	gi|260685375|ref|NC_013316.1| Clostridium difficile R20291 chromosome, complete genome
contig-100_3157	617	N	1	0	NA	0	0	0	NA	Faecalibacterium prausnitzii L2/6 draft genome	gi|479208076|ref|NC_021042.1| Faecalibacterium prausnitzii L2-6, complete genome	gi|479208076|ref|NC_021042.1| Faecalibacterium prausnitzii L2-6, complete genome
contig-100_3158	617	N	0	0	NA	0	0	0	NA	NA	NA	NA
contig-100_3159	617	N	0	0	NA	0	0	0	NA	NA	NA	NA
contig-100_3160	617	N	0	0	NA	0	0	0	NA	Uncultured organism clone VC1A604TF genomic sequence	NA	Uncultured organism clone VC1A604TF genomic sequence
contig-100_3161	617	N	0	0	NA	0	0	0	NA	NA	NA	NA
contig-100_3162	617	N	0	0	NA	0	0	0	NA	NA	NA	NA
contig-100_3163	617	N	0	0	NA	0	0	0	NA	NA	NA	NA
contig-100_3164	617	N	0	0	NA	0	0	0	NA	Haemophilus parainfluenzae T3T1 complete genome	gi|345428590|ref|NC_015964.1| Haemophilus parainfluenzae T3T1, complete genome	gi|345428590|ref|NC_015964.1| Haemophilus parainfluenzae T3T1, complete genome

contig-100_3165	616	N	0	0	NA	0	0	0	NA	Lactobacillus helveticus CNRZ32, complete genome	gi|525706521|ref|NC_021744.1| Lactobacillus helveticus CNRZ32, complete genome	gi|525706521|ref|NC_021744.1| Lactobacillus helveticus CNRZ32, complete genome
contig-100_3166	616	N	0	0	NA	0	0	0	NA	NA	NA	NA
contig-100_3167	616	N	0	0	NA	0	0	0	NA	NA	NA	NA
contig-100_3168	616	N	0	0	NA	0	0	0	NA	Uncultured organism clone VC1CI43TR genomic sequence	NA	Uncultured organism clone VC1CI43TR genomic sequence
contig-100_3169	616	N	0	0	NA	0	0	0	NA	NA	NA	NA
contig-100_3170	616	N	0	0	NA	0	0	0	NA	Faecalibacterium prausnitzii SL3/3 draft genome	gi|479170689|ref|NC_021020.1| Faecalibacterium prausnitzii SL3/3 draft genome	gi|479170689|ref|NC_021020.1| Faecalibacterium prausnitzii SL3/3 draft genome
contig-100_3173	615	N	0	0	NA	0	0	0	NA	NA	NA	NA
contig-100_3176	614	N	0	0	NA	0	0	0	NA	NA	NA	NA
contig-100_3177	614	N	0	0	NA	0	0	0	NA	Klebsiella pneumoniae plasmid pLVPK, complete sequence	gi|57639934|ref|NC_006625.1| Klebsiella pneumoniae NTUH-K2044 plasmid pK2044, complete sequence	gi|57639934|ref|NC_006625.1| Klebsiella pneumoniae NTUH-K2044 plasmid pK2044, complete sequence
contig-100_3178	614	N	0	0	NA	0	0	0	NA	NA	NA	NA
contig-100_3179	613	N	0	0	NA	0	0	0	NA	Bacteroides vulgatus ATCC 8482, complete genome	gi|150002608|ref|NC_009614.1| Bacteroides vulgatus ATCC 8482 chromosome, complete genome	gi|150002608|ref|NC_009614.1| Bacteroides vulgatus ATCC 8482 chromosome, complete genome

contig-100_3180	613	N	0	0	NA	0	0	0	NA	Faecalibacterium prausnitzii L2/6 draft genome	gi|479208076|ref|NC_021042.1| Faecalibacterium prausnitzii L2-6, complete genome	gi|479208076|ref|NC_021042.1| Faecalibacterium prausnitzii L2-6, complete genome
contig-100_3181	613	N	1	0	NA	0	0	0	NA	NA	NA	NA
contig-100_3182	613	N	0	0	NA	0	0	0	NA	NA	NA	NA
contig-100_3183	613	N	0	0	NA	0	0	0	NA	NA	NA	NA
contig-100_3184	613	N	0	0	NA	0	0	0	NA	NA	NA	NA
contig-100_3185	612	N	0	0	NA	0	0	0	NA	Eubacterium eligens ATCC 27750, complete genome	gi|238915976|ref|NC_012778.1| Eubacterium eligens ATCC 27750 chromosome, complete genome	gi|238915976|ref|NC_012778.1| Eubacterium eligens ATCC 27750 chromosome, complete genome
contig-100_3186	612	N	0	0	NA	0	0	0	NA	NA	NA	NA
contig-100_3187	612	N	1	0	NA	0	0	0	NA	NA	NA	NA
contig-100_3188	612	N	0	0	NA	0	0	0	NA	Odoribacter splanchnicus DSM 20712, complete genome	gi|325278757|ref|NC_015160.1| Odoribacter splanchnicus DSM 20712 chromosome, complete genome	gi|325278757|ref|NC_015160.1| Odoribacter splanchnicus DSM 20712 chromosome, complete genome
contig-100_3189	612	N	0	0	NA	0	0	0	NA	NA	NA	NA
contig-100_319	2536	N	2	1	Sipho	1	0	1	NA	NA	NA	NA
contig-100_3190	612	N	0	0	NA	0	0	0	NA	NA	NA	NA
contig-100_3191	612	N	0	0	NA	0	0	0	NA	Uncultured organism clone 1041059765583 genomic sequence	gi|479162165|ref|NC_021017.1| Bacteroides xylanisolvens XB1A draft genome	gi|479162165|ref|NC_021017.1| Bacteroides xylanisolvens XB1A draft genome
contig-100_3192	611	N	0	0	NA	0	0	0	NA	Uncultured organism clone VC1BX01TR genomic sequence	gi|479162165|ref|NC_021017.1| Bacteroides xylanisolvens XB1A draft genome	gi|479162165|ref|NC_021017.1| Bacteroides xylanisolvens XB1A draft genome

contig-100_3193	611	N	0	0	NA	0	0	0	NA	NA	NA	NA
contig-100_3194	611	N	0	0	NA	0	0	0	NA	NA	NA	NA
contig-100_3195	611	N	0	0	NA	0	0	0	NA	Streptococcus phage TP-J34 complete genome	NA	Streptococcus phage TP-J34 complete genome
contig-100_3196	611	N	1	0	NA	0	0	0	NA	NA	NA	NA
contig-100_3197	611	N	0	0	NA	0	0	0	NA	NA	NA	NA
contig-100_3198	611	N	0	0	NA	0	0	0	NA	NA	NA	NA
contig-100_3199	611	N	1	0	NA	0	0	0	NA	NA	NA	NA
contig-100_320	2517	N	1	0	NA	0	0	0	NA	Unidentified phage clone 2019_scaffold132 genomic sequence	NA	Unidentified phage clone 2019_scaffold 132 genomic sequence
contig-100_3200	610	N	1	0	NA	0	0	0	NA	Bacteroides xylanisolvens XB1A draft genome	gi|479162165|ref|NC_021017.1| Bacteroides xylanisolvens XB1A draft genome	gi|479162165|ref|NC_021017.1| Bacteroides xylanisolvens XB1A draft genome
contig-100_3201	610	N	1	0	NA	0	0	0	NA	NA	NA	NA
contig-100_3202	610	N	0	0	NA	0	0	0	NA	NA	NA	NA
contig-100_3203	609	N	0	0	NA	0	0	0	NA	Roseburia intestinalis XB6B4 draft genome	gi|479146200|ref|NC_021012.1| Roseburia intestinalis XB6B4 draft genome	gi|479146200|ref|NC_021012.1| Roseburia intestinalis XB6B4 draft genome
contig-100_3204	609	N	0	0	NA	0	0	0	NA	NA	NA	NA
contig-100_3205	609	N	0	0	NA	0	0	0	NA	NA	NA	NA
contig-100_3206	609	N	0	0	NA	0	0	0	NA	NA	NA	NA
contig-100_3207	608	N	0	0	NA	0	0	0	NA	NA	NA	NA
contig-100_3208	608	N	1	0	NA	0	0	0	NA	Clostridiales sp. SM4/1 draft genome	gi|479181986|ref|NC_021024.1| Butyrate-producing bacterium SM4/1, complete genome	gi|479181986|ref|NC_021024.1| Butyrate-producing bacterium SM4/1, complete genome
contig-100_3210	608	N	0	0	NA	0	0	0	NA	NA	NA	NA
contig-100_3211	608	N	1	0	NA	0	0	0	NA	NA	NA	NA

contig-100_3212	608	N	0	0	NA	0	0	0	NA	Faecalibacterium prausnitzii L2/6 draft genome	gi|479208076|ref|NC_021042.1| Faecalibacterium prausnitzii L2-6, complete genome	gi|479208076|ref|NC_021042.1| Faecalibacterium prausnitzii L2-6, complete genome
contig-100_3214	607	N	0	0	NA	0	0	0	NA	NA	NA	NA
contig-100_3215	607	N	0	0	NA	0	0	0	NA	NA	NA	NA
contig-100_3216	607	N	0	0	NA	0	0	0	NA	NA	NA	NA
contig-100_3217	607	N	1	0	NA	0	0	0	NA	NA	NA	NA
contig-100_3218	607	N	1	0	NA	0	0	0	NA	Uncultured organism clone 1 genomic sequence	NA	Uncultured organism clone 1 genomic sequence
contig-100_3219	607	N	0	0	NA	0	0	0	NA	NA	NA	NA
contig-100_322	2514	N	1	0	NA	0	0	0	NA	NA	NA	NA
contig-100_3220	606	N	0	0	NA	0	0	0	NA	NA	NA	NA
contig-100_3221	606	N	0	0	NA	0	0	0	NA	Uncultured bacterium clone LM0ABA3ZG03FM1 genomic sequence	gi|302346166|ref|NC_014371.1| Prevotella melaninogenica ATCC 25845 chromosome II, complete sequence	gi|302346166|ref|NC_014371.1| Prevotella melaninogenica ATCC 25845 chromosome II, complete sequence
contig-100_3222	606	N	0	0	NA	0	0	0	NA	Unidentified phage clone 1013_scaffold1877 genomic sequence	NA	Unidentified phage clone 1013_scaffold1877 genomic sequence
contig-100_3223	606	N	0	0	NA	0	0	0	NA	Faecalibacterium prausnitzii SL3/3 draft genome	gi|479170689|ref|NC_021020.1| Faecalibacterium prausnitzii SL3/3 draft genome	gi|479170689|ref|NC_021020.1| Faecalibacterium prausnitzii SL3/3 draft genome
contig-100_3225	605	N	0	0	NA	0	0	0	NA	NA	NA	NA
contig-100_3226	605	N	0	0	NA	0	0	0	NA	Clostridiales sp. SM4/1 draft genome	gi|479181986|ref|NC_021024.1| Butyrate-producing bacterium SM4/1, complete genome	gi|479181986|ref|NC_021024.1| Butyrate-producing bacterium SM4/1, complete genome

contig-100_3227	605	N	0	0	NA	0	0	0	NA	NA	NA	NA
contig-100_3228	605	N	0	0	NA	0	0	0	NA	NA	NA	NA
contig-100_3229	605	N	0	0	NA	0	0	0	NA	NA	NA	NA
contig-100_323	2504	N	0	0	NA	0	0	0	NA	NA	NA	NA
contig-100_3230	605	N	1	0	NA	0	0	0	NA	Bacteroides vulgatus ATCC 8482, complete genome	gi|150002608|ref|NC_009614.1| Bacteroides vulgatus ATCC 8482 chromosome, complete genome	gi|150002608|ref|NC_009614.1| Bacteroides vulgatus ATCC 8482 chromosome, complete genome
contig-100_3231	605	N	0	0	NA	0	0	0	NA	NA	NA	NA
contig-100_3232	605	N	0	0	NA	0	0	0	NA	NA	NA	NA
contig-100_3233	605	N	0	0	NA	0	0	0	NA	NA	NA	NA
contig-100_3234	604	N	0	0	NA	0	0	0	NA	Pseudomonas fluorescens SBW25 complete genome	gi|568136993|ref|NC_023064.1| Pseudomonas sp. TKP, complete genome	gi|568136993|ref|NC_023064.1| Pseudomonas sp. TKP, complete genome
contig-100_3235	604	N	0	0	NA	0	0	0	NA	NA	NA	NA
contig-100_3236	604	N	0	0	NA	0	0	0	NA	Clostridiales sp. SSC/2 draft genome	gi|479158859|ref|NC_021016.1| Butyrate-producing bacterium SSC/2, complete genome	gi|479158859|ref|NC_021016.1| Butyrate-producing bacterium SSC/2, complete genome
contig-100_3237	604	N	0	0	NA	0	0	0	NA	NA	NA	NA
contig-100_3238	604	N	0	0	NA	0	0	0	NA	Uncultured organism clone VC1AE49TF genomic sequence	NA	Uncultured organism clone VC1AE49TF genomic sequence
contig-100_3239	604	N	0	0	NA	0	0	0	NA	NA	NA	NA
contig-100_3240	604	N	0	0	NA	0	0	0	NA	NA	NA	NA
contig-100_3241	604	N	0	0	NA	0	0	0	NA	NA	NA	NA
contig-100_3243	603	N	1	0	NA	0	0	0	NA	NA	NA	NA

contig-100_3244	603	N	0	0	NA	0	0	0	NA	Haemophilus parainfluenzae T3T1 complete genome	gi|345428590|ref|NC_015964.1| Haemophilus parainfluenzae T3T1, complete genome	gi|345428590|ref|NC_015964.1| Haemophilus parainfluenzae T3T1, complete genome
contig-100_3245	603	N	0	0	NA	0	0	0	NA	NA	NA	NA
contig-100_3246	603	N	0	0	NA	0	0	0	NA	NA	NA	NA
contig-100_3247	603	N	1	0	NA	0	0	0	NA	NA	NA	NA
contig-100_3248	603	N	0	0	NA	0	0	0	NA	NA	NA	NA
contig-100_3251	603	N	0	0	NA	0	0	0	NA	NA	NA	NA
contig-100_3252	603	N	0	0	NA	0	0	0	NA	NA	NA	NA
contig-100_3253	602	N	0	0	NA	0	0	0	NA	NA	NA	NA
contig-100_3254	602	N	0	0	NA	0	0	0	NA	NA	NA	NA
contig-100_3255	602	N	1	0	NA	0	0	0	NA	Bacteroides xylanisolvens XB1A draft genome	gi|479162165|ref|NC_021017.1| Bacteroides xylanisolvens XB1A draft genome	gi|479162165|ref|NC_021017.1| Bacteroides xylanisolvens XB1A draft genome
contig-100_3256	602	N	0	0	NA	0	0	0	NA	NA	NA	NA
contig-100_3257	602	N	1	0	NA	0	0	0	NA	Bacteroides thetaiotaomicron VPI-5482, complete genome	gi|29345410|ref|NC_004663.1| Bacteroides thetaiotaomicron VPI-5482 chromosome, complete genome	gi|29345410|ref|NC_004663.1| Bacteroides thetaiotaomicron VPI-5482 chromosome, complete genome
contig-100_3258	602	N	0	0	NA	0	0	0	NA	NA	NA	NA
contig-100_3259	602	N	0	0	NA	0	0	0	NA	NA	NA	NA
contig-100_326	2485	N	1	0	NA	0	0	0	NA	Aggregatibacter aphrophilus NJ8700, complete genome	gi|251791816|ref|NC_012913.1| Aggregatibacter aphrophilus NJ8700 chromosome, complete genome	gi|251791816|ref|NC_012913.1| Aggregatibacter aphrophilus NJ8700 chromosome, complete genome
contig-100_3260	601	N	0	0	NA	0	0	0	NA	NA	NA	NA
contig-100_3261	601	N	0	0	NA	0	0	0	NA	NA	NA	NA

contig-100_3262	601	N	0	0	NA	0	0	0	NA	Streptococcus salivarius JIM8777 complete genome	gi|387783149|ref|NC_017595.1| Streptococcus salivarius JIM8777, complete genome	gi|387783149|ref|NC_017595.1| Streptococcus salivarius JIM8777, complete genome
contig-100_3263	601	N	0	0	NA	0	0	0	NA	Pseudomonas fluorescens A506, complete genome	gi|387891255|ref|NC_017911.1| Pseudomonas fluorescens A506 chromosome, complete genome	gi|387891255|ref|NC_017911.1| Pseudomonas fluorescens A506 chromosome, complete genome
contig-100_3264	601	N	0	0	NA	0	0	0	NA	NA	NA	NA
contig-100_3266	600	N	2	0	NA	0	0	0	NA	Clostridiales sp. SSC/2 draft genome	gi|479158859|ref|NC_021016.1| Butyrate-producing bacterium SSC/2, complete genome	gi|479158859|ref|NC_021016.1| Butyrate-producing bacterium SSC/2, complete genome
contig-100_3267	600	N	0	0	NA	0	0	0	NA	Parabacteroides distasonis ATCC 8503, complete genome	gi|150006674|ref|NC_009615.1| Parabacteroides distasonis ATCC 8503 chromosome, complete genome	gi|150006674|ref|NC_009615.1| Parabacteroides distasonis ATCC 8503 chromosome, complete genome
contig-100_3268	600	N	0	0	NA	0	0	0	NA	Bacteroides vulgatus ATCC 8482, complete genome	gi|150002608|ref|NC_009614.1| Bacteroides vulgatus ATCC 8482 chromosome, complete genome	gi|150002608|ref|NC_009614.1| Bacteroides vulgatus ATCC 8482 chromosome, complete genome

contig-100_3269	600	N	0	0	NA	0	0	0	NA	Clostridiales sp. SSC/2 draft genome	gi|479158859|ref|NC_021016.1| Butyrate-producing bacterium SSC/2, complete genome	gi|479158859|ref|NC_021016.1| Butyrate-producing bacterium SSC/2, complete genome
contig-100_3270	600	N	0	0	NA	0	0	0	NA	Faecalibacterium prausnitzii L2/6 draft genome	gi|479208076|ref|NC_021042.1| Faecalibacterium prausnitzii L2-6, complete genome	gi|479208076|ref|NC_021042.1| Faecalibacterium prausnitzii L2-6, complete genome
contig-100_3271	600	N	2	0	NA	0	0	0	NA	Bacteroides vulgatus ATCC 8482, complete genome	gi|150002608|ref|NC_009614.1| Bacteroides vulgatus ATCC 8482 chromosome, complete genome	gi|150002608|ref|NC_009614.1| Bacteroides vulgatus ATCC 8482 chromosome, complete genome
contig-100_3272	599	N	0	0	NA	0	0	0	NA	NA	NA	NA
contig-100_3273	599	N	0	0	NA	0	0	0	NA	NA	NA	NA
contig-100_3274	599	N	0	0	NA	0	0	0	NA	NA	NA	NA
contig-100_3275	598	N	0	0	NA	0	0	0	NA	Faecalibacterium prausnitzii SL3/3 draft genome	gi|479170689|ref|NC_021020.1| Faecalibacterium prausnitzii SL3/3 draft genome	gi|479170689|ref|NC_021020.1| Faecalibacterium prausnitzii SL3/3 draft genome
contig-100_3276	598	N	0	0	NA	0	0	0	NA	NA	NA	NA
contig-100_3279	598	N	0	0	NA	0	0	0	NA	Bacteroides vulgatus ATCC 8482, complete genome	gi|150002608|ref|NC_009614.1| Bacteroides vulgatus ATCC 8482 chromosome, complete genome	gi|150002608|ref|NC_009614.1| Bacteroides vulgatus ATCC 8482 chromosome, complete genome
contig-100_328	2483	N	1	0	NA	0	0	0	NA	NA	NA	NA

contig-100_3280	598	N	0	0	NA	0	0	0	NA	Uncultured organism clone VC1C182TR genomic sequence	gi|479208076|ref|NC_021042.1| Faecalibacterium prausnitzii L2-6, complete genome	gi|479208076|ref|NC_021042.1| Faecalibacterium prausnitzii L2-6, complete genome
contig-100_3281	598	N	0	0	NA	0	0	0	NA	NA	NA	NA
contig-100_3282	598	N	0	0	NA	0	0	0	NA	NA	NA	NA
contig-100_3283	598	N	0	0	NA	0	0	0	NA	NA	NA	NA
contig-100_3286	597	N	0	0	NA	0	0	0	NA	Bacteroides vulgatus ATCC 8482, complete genome	gi|150002608|ref|NC_009614.1| Bacteroides vulgatus ATCC 8482 chromosome, complete genome	gi|150002608|ref|NC_009614.1| Bacteroides vulgatus ATCC 8482 chromosome, complete genome
contig-100_3287	597	N	0	0	NA	0	0	0	NA	NA	NA	NA
contig-100_3288	597	N	0	0	NA	0	0	0	NA	NA	NA	NA
contig-100_3289	597	N	0	0	NA	0	0	0	NA	NA	NA	NA
contig-100_329	2480	N	2	0	NA	0	0	0	NA	NA	NA	NA
contig-100_3290	597	N	0	0	NA	0	0	0	NA	NA	NA	NA
contig-100_3291	596	N	0	0	NA	0	0	0	NA	Faecalibacterium prausnitzii SL3/3 draft genome	gi|479170689|ref|NC_021020.1| Faecalibacterium prausnitzii SL3/3 draft genome	gi|479170689|ref|NC_021020.1| Faecalibacterium prausnitzii SL3/3 draft genome
contig-100_3292	596	N	0	0	NA	0	0	0	NA	Eubacterium eligens ATCC 27750, complete genome	gi|238915976|ref|NC_012778.1| Eubacterium eligens ATCC 27750 chromosome, complete genome	gi|238915976|ref|NC_012778.1| Eubacterium eligens ATCC 27750 chromosome, complete genome
contig-100_3293	596	N	0	0	NA	0	0	0	NA	NA	NA	NA
contig-100_3294	596	N	0	0	NA	0	0	0	NA	NA	NA	NA
contig-100_3295	596	N	0	0	NA	0	0	0	NA	NA	NA	NA
contig-100_3296	596	N	0	0	NA	0	0	0	NA	NA	NA	NA
contig-100_3297	596	N	0	0	NA	0	0	0	NA	NA	NA	NA

contig-100_3298	595	N	0	0	NA	0	0	0	NA	Faecalibacterium prausnitzii SL3/3 draft genome	gi|479170689|ref|NC_021020.1| Faecalibacterium prausnitzii SL3/3 draft genome	gi|479170689|ref|NC_021020.1| Faecalibacterium prausnitzii SL3/3 draft genome
contig-100_3299	595	N	0	0	NA	0	0	0	NA	Clostridium saccharolyticum-like K10 draft genome	gi|479336697|ref|NC_021047.1| Clostridium cf. saccharolyticum K10, complete genome	gi|479336697|ref|NC_021047.1| Clostridium cf. saccharolyticum K10, complete genome
contig-100_330	2478	N	2	0	NA	0	1	0	NA	Propionibacterium acnes 6609, complete genome	gi|50841496|ref|NC_006085.1| Propionibacterium acnes KPA171202 chromosome, complete genome	gi|50841496|ref|NC_006085.1| Propionibacterium acnes KPA171202 chromosome, complete genome
contig-100_3300	594	N	0	0	NA	0	0	0	NA	NA	NA	NA
contig-100_3301	594	N	0	0	NA	0	0	0	NA	NA	NA	NA
contig-100_3302	594	N	0	0	NA	0	0	0	NA	Bacteroides vulgatus ATCC 8482, complete genome	gi|150002608|ref|NC_009614.1| Bacteroides vulgatus ATCC 8482 chromosome, complete genome	gi|150002608|ref|NC_009614.1| Bacteroides vulgatus ATCC 8482 chromosome, complete genome
contig-100_3303	594	N	0	0	NA	0	0	0	NA	Bacteroides vulgatus ATCC 8482, complete genome	gi|150002608|ref|NC_009614.1| Bacteroides vulgatus ATCC 8482 chromosome, complete genome	gi|150002608|ref|NC_009614.1| Bacteroides vulgatus ATCC 8482 chromosome, complete genome
contig-100_3304	594	N	0	0	NA	0	0	0	NA	NA	NA	NA
contig-100_3305	593	N	0	0	NA	0	0	0	NA	NA	NA	NA
contig-100_3306	593	N	0	0	NA	0	0	0	NA	NA	NA	NA
contig-100_3308	593	N	0	0	NA	0	0	0	NA	NA	NA	NA
contig-100_3309	593	N	1	0	NA	0	0	0	NA	NA	NA	NA

contig-100_331	2476	N	1	0	NA	0	0	0	NA	NA	NA	NA
contig-100_3310	593	N	0	0	NA	0	0	0	NA	NA	NA	NA
contig-100_3311	593	N	0	0	NA	0	0	0	NA	NA	NA	NA
contig-100_3312	593	N	0	0	NA	0	0	0	NA	NA	NA	NA
contig-100_3313	593	N	1	0	NA	0	0	0	NA	Bacteroides vulgatus ATCC 8482, complete genome	gi|150002608|ref|NC_009614.1| Bacteroides vulgatus ATCC 8482 chromosome, complete genome	gi|150002608|ref|NC_009614.1| Bacteroides vulgatus ATCC 8482 chromosome, complete genome
contig-100_3314	592	N	0	0	NA	0	0	0	NA	NA	NA	NA
contig-100_3315	592	N	0	0	NA	0	0	0	NA	NA	NA	NA
contig-100_3317	592	N	1	0	NA	0	0	0	NA	NA	NA	NA
contig-100_3318	592	N	0	0	NA	0	0	0	NA	NA	NA	NA
contig-100_3319	592	N	0	0	NA	0	0	0	NA	NA	NA	NA
contig-100_332	2476	N	0	0	NA	0	0	0	NA	NA	NA	NA
contig-100_3321	591	N	0	0	NA	0	0	0	NA	Eubacterium rectale ATCC 33656, complete genome	gi|238922432|ref|NC_012781.1| Eubacterium rectale ATCC 33656, complete genome	gi|238922432|ref|NC_012781.1| Eubacterium rectale ATCC 33656, complete genome
contig-100_3322	591	N	0	0	NA	0	0	0	NA	NA	NA	NA
contig-100_3323	591	N	0	0	NA	0	0	0	NA	Faecalibacterium prausnitzii L2/6 draft genome	gi|479208076|ref|NC_021042.1| Faecalibacterium prausnitzii L2-6, complete genome	gi|479208076|ref|NC_021042.1| Faecalibacterium prausnitzii L2-6, complete genome
contig-100_3324	591	N	0	0	NA	0	0	0	NA	Faecalibacterium prausnitzii SL3/3 draft genome	gi|479170689|ref|NC_021020.1| Faecalibacterium prausnitzii SL3/3 draft genome	gi|479170689|ref|NC_021020.1| Faecalibacterium prausnitzii SL3/3 draft genome

contig-100_3326	591	N	0	0	NA	0	0	0	NA	Eubacterium rectale ATCC 33656, complete genome	gi|238922432|ref|NC_012781.1| Eubacterium rectale ATCC 33656, complete genome	gi|238922432|ref|NC_012781.1| Eubacterium rectale ATCC 33656, complete genome
contig-100_3327	591	N	0	0	NA	0	0	0	NA	Uncultured organism clone 1041059764718 genomic sequence	NA	Uncultured organism clone 1041059764718 genomic sequence
contig-100_3328	590	N	0	0	NA	0	0	0	NA	NA	NA	NA
contig-100_3329	590	N	0	0	NA	0	0	0	NA	NA	NA	NA
contig-100_333	2472	N	1	0	NA	0	0	0	NA	NA	NA	NA
contig-100_3330	590	N	0	0	NA	0	0	0	NA	Uncultured organism clone VC1CJ42TF genomic sequence	NA	Uncultured organism clone VC1CJ42TF genomic sequence
contig-100_3331	590	N	0	0	NA	0	0	0	NA	NA	NA	NA
contig-100_3332	590	N	1	0	NA	0	0	0	NA	Bacteroides helcogenes P 36-108, complete genome	gi|319899888|ref|NC_014933.1| Bacteroides helcogenes P 36-108 chromosome, complete genome	gi|319899888|ref|NC_014933.1| Bacteroides helcogenes P 36-108 chromosome, complete genome
contig-100_3334	590	N	0	0	NA	0	0	0	NA	Streptococcus phage TP-J34 complete genome	NA	Streptococcus phage TP-J34 complete genome
contig-100_3335	589	N	0	0	NA	0	0	0	NA	Faecalibacterium prausnitzii SL3/3 draft genome	gi|479170689|ref|NC_021020.1| Faecalibacterium prausnitzii SL3/3 draft genome	gi|479170689|ref|NC_021020.1| Faecalibacterium prausnitzii SL3/3 draft genome

contig-100_3336	589	N	0	0	NA	0	0	0	NA	Bacteroides vulgatus ATCC 8482, complete genome	gi|150002608|ref|NC_009614.1| Bacteroides vulgatus ATCC 8482 chromosome, complete genome	gi|150002608|ref|NC_009614.1| Bacteroides vulgatus ATCC 8482 chromosome, complete genome
contig-100_3337	589	N	0	0	NA	0	0	0	NA	NA	NA	NA
contig-100_3338	589	N	0	0	NA	0	0	0	NA	Clostridiales sp. SM4/1 draft genome	gi|479181986|ref|NC_021024.1| Butyrate-producing bacterium SM4/1, complete genome	gi|479181986|ref|NC_021024.1| Butyrate-producing bacterium SM4/1, complete genome
contig-100_3339	589	N	0	0	NA	0	0	0	NA	Clostridiales sp. SM4/1 draft genome	gi|479181986|ref|NC_021024.1| Butyrate-producing bacterium SM4/1, complete genome	gi|479181986|ref|NC_021024.1| Butyrate-producing bacterium SM4/1, complete genome
contig-100_3340	589	N	0	0	NA	0	0	0	NA	NA	NA	NA
contig-100_3342	588	N	0	0	NA	0	0	0	NA	Eubacterium eligens ATCC 27750, complete genome	gi|238915976|ref|NC_012778.1| Eubacterium eligens ATCC 27750 chromosome, complete genome	gi|238915976|ref|NC_012778.1| Eubacterium eligens ATCC 27750 chromosome, complete genome
contig-100_3343	588	N	1	0	NA	0	0	0	NA	Uncultured organism clone VC1C773TR genomic sequence	NA	Uncultured organism clone VC1C773TR genomic sequence
contig-100_3344	588	N	1	0	NA	0	0	0	NA	NA	NA	NA

contig-100_3345	588	N	0	0	NA	0	0	0	NA	Bacteroides vulgatus ATCC 8482, complete genome	gi|150002608|ref|NC_009614.1| Bacteroides vulgatus ATCC 8482 chromosome, complete genome	gi|150002608|ref|NC_009614.1| Bacteroides vulgatus ATCC 8482 chromosome, complete genome
contig-100_3346	588	N	1	0	NA	0	0	0	NA	NA	NA	NA
contig-100_3347	588	N	0	0	NA	0	0	0	NA	NA	NA	NA
contig-100_3348	588	N	0	0	NA	0	0	0	NA	NA	NA	NA
contig-100_3349	587	N	0	0	NA	0	0	0	NA	Veillonella parvula DSM 2008, complete genome	gi|269797069|ref|NC_013520.1| Veillonella parvula DSM 2008 chromosome, complete genome	gi|269797069|ref|NC_013520.1| Veillonella parvula DSM 2008 chromosome, complete genome
contig-100_335	2467	N	4	1	Podo	0	0	1	NA	NA	NA	NA
contig-100_3350	587	N	0	0	NA	0	0	0	NA	Faecalibacterium prausnitzii L2/6 draft genome	gi|479208076|ref|NC_021042.1| Faecalibacterium prausnitzii L2-6, complete genome	gi|479208076|ref|NC_021042.1| Faecalibacterium prausnitzii L2-6, complete genome
contig-100_3351	587	N	0	0	NA	0	0	0	NA	NA	NA	NA
contig-100_3352	587	N	0	0	NA	0	0	0	NA	NA	NA	NA
contig-100_3353	587	N	0	0	NA	0	0	0	NA	Clostridiales sp. SM4/1 draft genome	gi|479181986|ref|NC_021024.1| Butyrate-producing bacterium SM4/1, complete genome	gi|479181986|ref|NC_021024.1| Butyrate-producing bacterium SM4/1, complete genome
contig-100_3354	587	N	0	0	NA	0	0	0	NA	Acidovorax sp. JS42, complete genome	gi|121592436|ref|NC_008782.1| Acidovorax sp. JS42 chromosome, complete genome	gi|121592436|ref|NC_008782.1| Acidovorax sp. JS42 chromosome, complete genome
contig-100_3355	587	N	0	0	NA	0	0	0	NA	NA	NA	NA

contig-100_3358	587	N	0	0	NA	0	0	0	NA	Faecalibacterium prausnitzii SL3/3 draft genome	gi|479170689|ref|NC_021020.1| Faecalibacterium prausnitzii SL3/3 draft genome	gi|479170689|ref|NC_021020.1| Faecalibacterium prausnitzii SL3/3 draft genome
contig-100_3359	587	N	0	0	NA	0	0	0	NA	NA	NA	NA
contig-100_3361	587	N	0	0	NA	0	0	0	NA	NA	NA	NA
contig-100_3362	586	N	0	0	NA	0	0	0	NA	NA	NA	NA
contig-100_3363	586	N	0	0	NA	0	0	0	NA	NA	NA	NA
contig-100_3364	586	N	0	0	NA	0	0	0	NA	NA	NA	NA
contig-100_3365	586	N	0	0	NA	0	0	0	NA	Haemophilus parainfluenzae T3T1 complete genome	gi|345428590|ref|NC_015964.1| Haemophilus parainfluenzae T3T1, complete genome	gi|345428590|ref|NC_015964.1| Haemophilus parainfluenzae T3T1, complete genome
contig-100_3366	585	N	0	0	NA	0	0	0	NA	Lactobacillus casei W56 complete genome	gi|409995665|ref|NC_018641.1| Lactobacillus casei W56, complete genome	gi|409995665|ref|NC_018641.1| Lactobacillus casei W56, complete genome
contig-100_3367	585	N	0	0	NA	0	0	0	NA	Bacteroides vulgatus ATCC 8482, complete genome	gi|150002608|ref|NC_009614.1| Bacteroides vulgatus ATCC 8482 chromosome, complete genome	gi|150002608|ref|NC_009614.1| Bacteroides vulgatus ATCC 8482 chromosome, complete genome
contig-100_3368	585	N	0	0	NA	0	0	0	NA	NA	NA	NA
contig-100_3369	585	N	0	0	NA	0	0	0	NA	NA	NA	NA
contig-100_337	2462	N	2	0	NA	0	0	0	NA	NA	NA	NA
contig-100_3370	585	N	0	0	NA	0	0	0	NA	NA	NA	NA
contig-100_3371	584	N	0	0	NA	0	0	0	NA	NA	NA	NA
contig-100_3372	584	N	0	0	NA	0	0	0	NA	NA	NA	NA

contig-100_3373	584	N	2	0	NA	0	0	0	NA	Faecalibacterium prausnitzii L2/6 draft genome	gi|479208076|ref|NC_021042.1| Faecalibacterium prausnitzii L2-6, complete genome	gi|479208076|ref|NC_021042.1| Faecalibacterium prausnitzii L2-6, complete genome
contig-100_3374	584	N	0	0	NA	0	0	0	NA	Faecalibacterium prausnitzii SL3/3 draft genome	gi|479170689|ref|NC_021020.1| Faecalibacterium prausnitzii SL3/3 draft genome	gi|479170689|ref|NC_021020.1| Faecalibacterium prausnitzii SL3/3 draft genome
contig-100_3375	584	N	0	0	NA	0	0	0	NA	NA	NA	NA
contig-100_3376	584	N	1	0	NA	0	0	0	NA	Uncultured bacterium clone HA0AAA15ZH10FM1 genomic sequence	NA	Uncultured bacterium clone HA0AAA15ZH10FM1 genomic sequence
contig-100_3377	584	N	0	0	NA	0	0	0	NA	NA	NA	NA
contig-100_3378	583	N	0	0	NA	0	0	0	NA	NA	NA	NA
contig-100_3379	583	N	0	0	NA	0	0	0	NA	Eubacterium rectale ATCC 33656, complete genome	gi|238922432|ref|NC_012781.1| Eubacterium rectale ATCC 33656, complete genome	gi|238922432|ref|NC_012781.1| Eubacterium rectale ATCC 33656, complete genome
contig-100_338	2458	N	2	1	Sipho	1	0	1	NA	NA	NA	NA
contig-100_3380	583	N	0	0	NA	0	0	0	NA	Faecalibacterium prausnitzii SL3/3 draft genome	gi|479170689|ref|NC_021020.1| Faecalibacterium prausnitzii SL3/3 draft genome	gi|479170689|ref|NC_021020.1| Faecalibacterium prausnitzii SL3/3 draft genome
contig-100_3381	583	N	0	0	NA	0	0	0	NA	Faecalibacterium prausnitzii L2/6 draft genome	gi|479208076|ref|NC_021042.1| Faecalibacterium prausnitzii L2-6, complete genome	gi|479208076|ref|NC_021042.1| Faecalibacterium prausnitzii L2-6, complete genome

contig-100_3382	583	N	0	0	NA	0	0	0	NA	Unidentified phage clone 2019_scaffold132 genomic sequence	NA	Unidentified phage clone 2019_scaffold 132 genomic sequence
contig-100_3384	583	N	1	0	NA	0	0	0	NA	NA	NA	NA
contig-100_3385	582	N	0	0	NA	0	0	0	NA	Uncultured organism clone 1041059766314 genomic sequence	NA	Uncultured organism clone 104105976631 4 genomic sequence
contig-100_3387	582	N	0	0	NA	0	0	0	NA	NA	NA	NA
contig-100_3388	582	N	1	0	NA	0	0	0	NA	Pantoea vagans C9-1, complete genome	gi|308185541|ref|NC_014562.1| Pantoea vagans C9-1 chromosome, complete genome	gi|308185541|ref|NC_014562 .1| Pantoea vagans C9-1 chromosome, complete genome
contig-100_3389	581	N	0	0	NA	0	0	0	NA	NA	NA	NA
contig-100_339	2451	N	4	2	Myo	0	0	2	NA	NA	NA	NA
contig-100_3390	581	N	0	0	NA	0	0	0	NA	NA	NA	NA
contig-100_3391	581	N	1	0	NA	0	0	0	NA	NA	NA	NA
contig-100_3392	581	N	0	0	NA	0	0	0	NA	Eubacterium siraeum 70/3 draft genome	gi|479143419|ref|NC_021011.1| Eubacterium siraeum 70/3 draft genome	gi|479143419|ref|NC_021011 .1| Eubacterium siraeum 70/3 draft genome
contig-100_3393	581	N	0	0	NA	0	0	0	NA	NA	NA	NA
contig-100_3394	581	N	0	0	NA	0	0	0	NA	NA	NA	NA
contig-100_3395	581	N	0	0	NA	0	0	0	NA	Haemophilus parainfluenzae T3T1 complete genome	gi|345428590|ref|NC_015964.1| Haemophilus parainfluenzae T3T1, complete genome	gi|345428590|ref|NC_015964 .1| Haemophilus parainfluenzae T3T1, complete genome
contig-100_3396	581	N	0	0	NA	0	0	0	NA	NA	NA	NA
contig-100_3397	580	N	1	0	NA	0	0	0	NA	Uncultured organism clone 1041059764514 genomic sequence	NA	Uncultured organism clone 104105976451 4 genomic sequence

contig-100_3398	580	N	0	0	NA	0	0	0	NA	Faecalibacterium prausnitzii L2/6 draft genome	gi|479208076|ref|NC_021042.1| Faecalibacterium prausnitzii L2-6, complete genome	gi|479208076|ref|NC_021042.1| Faecalibacterium prausnitzii L2-6, complete genome
contig-100_340	2450	N	0	0	NA	0	0	0	NA	Clostridiales sp. SSC/2 draft genome	gi|479158859|ref|NC_021016.1| Butyrate-producing bacterium SSC/2, complete genome	gi|479158859|ref|NC_021016.1| Butyrate-producing bacterium SSC/2, complete genome
contig-100_3400	580	N	0	0	NA	0	0	0	NA	NA	NA	NA
contig-100_3401	580	N	0	0	NA	0	0	0	NA	NA	NA	NA
contig-100_3402	579	N	0	0	NA	0	0	0	NA	Parabacteroides distasonis ATCC 8503, complete genome	gi|150006674|ref|NC_009615.1| Parabacteroides distasonis ATCC 8503 chromosome, complete genome	gi|150006674|ref|NC_009615.1| Parabacteroides distasonis ATCC 8503 chromosome, complete genome
contig-100_3403	579	N	0	0	NA	0	0	0	NA	NA	NA	NA
contig-100_3404	579	N	2	0	NA	0	0	0	NA	NA	NA	NA
contig-100_3405	578	N	0	0	NA	0	0	0	NA	NA	NA	NA
contig-100_3406	578	N	0	0	NA	0	0	0	NA	NA	NA	NA
contig-100_3407	578	N	0	0	NA	0	0	0	NA	NA	NA	NA
contig-100_3408	577	N	0	0	NA	0	0	0	NA	NA	NA	NA
contig-100_3409	577	N	0	0	NA	0	0	0	NA	Bacteroides salanitronis DSM 18170, complete genome	gi|325297172|ref|NC_015164.1| Bacteroides salanitronis DSM 18170 chromosome, complete genome	gi|325297172|ref|NC_015164.1| Bacteroides salanitronis DSM 18170 chromosome, complete genome
contig-100_3410	577	N	0	0	NA	0	0	0	NA	NA	NA	NA
contig-100_3412	577	N	0	0	NA	0	0	0	NA	NA	NA	NA
contig-100_3413	576	N	0	0	NA	0	0	0	NA	NA	NA	NA
contig-100_3415	576	N	0	0	NA	0	0	0	NA	NA	NA	NA
contig-100_3417	576	N	0	0	NA	0	0	0	NA	NA	NA	NA

contig-100_3418	576	N	0	0	NA	0	0	0	NA	Parabacteroides distasonis ATCC 8503, complete genome	gi|150006674|ref|NC_009615.1| Parabacteroides distasonis ATCC 8503 chromosome, complete genome	gi|150006674|ref|NC_009615.1| Parabacteroides distasonis ATCC 8503 chromosome, complete genome
contig-100_3419	576	N	0	0	NA	0	0	0	NA	NA	NA	NA
contig-100_342	2447	N	2	0	NA	0	0	0	NA	NA	NA	NA
contig-100_3420	576	N	0	0	NA	0	0	0	NA	Faecalibacterium prausnitzii SL3/3 draft genome	gi|479170689|ref|NC_021020.1| Faecalibacterium prausnitzii SL3/3 draft genome	gi|479170689|ref|NC_021020.1| Faecalibacterium prausnitzii SL3/3 draft genome
contig-100_3421	575	N	0	0	NA	0	0	0	NA	NA	NA	NA
contig-100_3422	575	N	0	0	NA	0	0	0	NA	NA	NA	NA
contig-100_3423	575	N	0	0	NA	0	0	0	NA	Eubacterium rectale ATCC 33656, complete genome	gi|238922432|ref|NC_012781.1| Eubacterium rectale ATCC 33656, complete genome	gi|238922432|ref|NC_012781.1| Eubacterium rectale ATCC 33656, complete genome
contig-100_3424	575	N	0	0	NA	0	0	0	NA	NA	NA	NA
contig-100_3425	575	N	1	0	NA	0	0	0	NA	NA	NA	NA
contig-100_3426	575	N	0	0	NA	0	0	0	NA	Bifidobacterium breve ACS-071-V-Sch8b, complete genome	gi|384196195|ref|NC_017218.1| Bifidobacterium breve ACS-071-V-Sch8b chromosome, complete genome	gi|384196195|ref|NC_017218.1| Bifidobacterium breve ACS-071-V-Sch8b chromosome, complete genome
contig-100_3427	574	N	0	0	NA	0	0	0	NA	NA	NA	NA
contig-100_3428	574	N	0	0	NA	0	0	0	NA	NA	NA	NA
contig-100_3429	574	N	0	0	NA	0	0	0	NA	Roseburia hominis A2-183, complete genome	gi|347530298|ref|NC_015977.1| Roseburia hominis A2-183 chromosome, complete genome	gi|347530298|ref|NC_015977.1| Roseburia hominis A2-183 chromosome, complete genome
contig-100_343	2446	N	2	2	Asco	0	1	1	NA	NA	NA	NA

contig-100_3430	574	N	0	0	NA	0	0	0	NA	NA	NA	NA
contig-100_3431	574	N	0	0	NA	0	0	0	NA	NA	NA	NA
contig-100_3432	574	N	1	0	NA	0	0	0	NA	NA	NA	NA
contig-100_3433	574	N	0	0	NA	0	0	0	NA	Candidatus Saccharimonas aalborgensis, complete genome	gi|563711419|ref|NC_023004.1| Candidatus Saccharibacteria bacterium RAAC3_TM7_1, complete genome	gi|563711419|ref|NC_023004.1| Candidatus Saccharibacteria bacterium RAAC3_TM7_1, complete genome
contig-100_3434	574	N	0	0	NA	0	0	0	NA	NA	NA	NA
contig-100_3436	573	N	1	0	NA	0	0	0	NA	Faecalibacterium prausnitzii SL3/3 draft genome	gi|479170689|ref|NC_021020.1| Faecalibacterium prausnitzii SL3/3 draft genome	gi|479170689|ref|NC_021020.1| Faecalibacterium prausnitzii SL3/3 draft genome
contig-100_3437	573	N	1	0	NA	0	0	0	NA	NA	NA	NA
contig-100_3438	573	N	0	0	NA	0	0	0	NA	NA	NA	NA
contig-100_3439	573	N	0	0	NA	0	0	0	NA	NA	NA	NA
contig-100_3440	573	N	0	0	NA	0	0	0	NA	NA	NA	NA
contig-100_3441	573	N	0	0	NA	0	0	0	NA	NA	NA	NA
contig-100_3442	573	N	0	0	NA	0	0	0	NA	Delftia acidovorans plasmid pUO1 DNA, complete sequence	gi|407936729|ref|NC_018708.1| Acidovorax sp. KKS102 chromosome, complete genome	gi|407936729|ref|NC_018708.1| Acidovorax sp. KKS102 chromosome, complete genome
contig-100_3443	573	N	1	0	NA	0	0	0	NA	NA	NA	NA
contig-100_3444	572	N	0	0	NA	0	0	0	NA	NA	NA	NA
contig-100_3445	572	N	0	0	NA	0	0	0	NA	NA	NA	NA
contig-100_3446	572	N	0	0	NA	0	0	0	NA	NA	NA	NA
contig-100_3447	572	N	0	0	NA	0	0	0	NA	NA	NA	NA
contig-100_3448	572	N	0	0	NA	0	0	0	NA	NA	NA	NA
contig-100_3449	572	N	0	0	NA	0	0	0	NA	Faecalibacterium prausnitzii L2/6 draft genome	gi|479208076|ref|NC_021042.1| Faecalibacterium prausnitzii L2-6, complete genome	gi|479208076|ref|NC_021042.1| Faecalibacterium prausnitzii L2-6, complete genome
contig-100_3450	571	N	1	0	NA	0	0	0	NA	NA	NA	NA

contig-100_3451	571	N	0	0	NA	0	0	0	NA	NA	NA	NA
contig-100_3452	571	N	0	0	NA	0	0	0	NA	NA	NA	NA
contig-100_3453	571	N	1	0	NA	0	0	0	NA	NA	NA	NA
contig-100_3454	571	N	0	0	NA	0	0	0	NA	Lactobacillus paracasei subsp. paracasei JCM 8130 DNA, complete genome	gi|523512490|ref|NC_021721.1| Lactobacillus casei LOCK919, complete genome	gi|523512490|ref|NC_021721.1| Lactobacillus casei LOCK919, complete genome
contig-100_3455	571	N	0	0	NA	0	0	0	NA	NA	NA	NA
contig-100_3456	571	N	0	0	NA	0	0	0	NA	NA	NA	NA
contig-100_3457	571	N	0	0	NA	0	0	0	NA	Haemophilus parainfluenzae T3T1 complete genome	gi|345428590|ref|NC_015964.1| Haemophilus parainfluenzae T3T1, complete genome	gi|345428590|ref|NC_015964.1| Haemophilus parainfluenzae T3T1, complete genome
contig-100_3458	571	N	0	0	NA	0	0	0	NA	NA	NA	NA
contig-100_3459	570	N	0	0	NA	0	0	0	NA	NA	NA	NA
contig-100_346	2433	N	2	0	NA	0	0	0	NA	NA	NA	NA
contig-100_3460	570	N	1	0	NA	0	0	0	NA	NA	NA	NA
contig-100_3461	570	N	0	0	NA	0	0	0	NA	Ruminococcus obeum A2-162 draft genome	gi|479176048|ref|NC_021022.1| Ruminococcus obeum A2-162 draft genome	gi|479176048|ref|NC_021022.1| Ruminococcus obeum A2-162 draft genome
contig-100_3462	570	N	0	0	NA	0	0	0	NA	NA	NA	NA
contig-100_3463	570	N	0	0	NA	0	0	0	NA	Clostridiales sp. SSC/2 draft genome	gi|479158859|ref|NC_021016.1| Butyrate-producing bacterium SSC/2, complete genome	gi|479158859|ref|NC_021016.1| Butyrate-producing bacterium SSC/2, complete genome
contig-100_3464	570	N	0	0	NA	0	0	0	NA	Uncultured organism clone 1041059764562 genomic sequence	NA	Uncultured organism clone 1041059764562 genomic sequence
contig-100_3465	570	N	0	0	NA	0	0	0	NA	NA	NA	NA
contig-100_3466	570	N	0	0	NA	0	0	0	NA	NA	NA	NA

contig-100_3467	570	N	0	0	NA	0	0	0	NA	Uncultured organism clone VC1D676TF genomic sequence	NA	Uncultured organism clone VC1D676TF genomic sequence
contig-100_3468	570	N	1	0	NA	0	0	0	NA	Klebsiella pneumoniae subsp. pneumoniae 1084, complete genome	gi|550443072|ref|NC_022566.1| Klebsiella pneumoniae CG43, complete genome	gi|550443072|ref|NC_022566.1| Klebsiella pneumoniae CG43, complete genome
contig-100_3469	570	N	0	0	NA	0	0	0	NA	NA	NA	NA
contig-100_347	2432	N	1	1	Sipho	1	0	1	NA	Unidentified phage clone 2019_scaffold132 genomic sequence	NA	Unidentified phage clone 2019_scaffold132 genomic sequence
contig-100_3471	570	N	0	0	NA	0	0	0	NA	NA	NA	NA
contig-100_3472	569	N	0	0	NA	0	0	0	NA	Faecalibacterium prausnitzii L2/6 draft genome	gi|479208076|ref|NC_021042.1| Faecalibacterium prausnitzii L2-6, complete genome	gi|479208076|ref|NC_021042.1| Faecalibacterium prausnitzii L2-6, complete genome
contig-100_3473	569	N	0	0	NA	0	0	0	NA	NA	NA	NA
contig-100_3474	569	N	0	0	NA	0	0	0	NA	NA	NA	NA
contig-100_3475	569	N	0	0	NA	0	0	0	NA	NA	NA	NA
contig-100_3477	568	N	0	0	NA	0	0	0	NA	Uncultured organism clone VC1BT95TR genomic sequence	NA	Uncultured organism clone VC1BT95TR genomic sequence
contig-100_3478	568	N	0	0	NA	0	0	0	NA	NA	NA	NA
contig-100_3479	568	N	1	0	NA	0	0	0	NA	NA	NA	NA
contig-100_348	2428	N	3	1	Sipho	1	0	1	NA	NA	NA	NA
contig-100_3481	568	N	0	0	NA	0	0	0	NA	Odoribacter splanchnicus DSM 20712, complete genome	gi|325278757|ref|NC_015160.1| Odoribacter splanchnicus DSM 20712 chromosome, complete genome	gi|325278757|ref|NC_015160.1| Odoribacter splanchnicus DSM 20712 chromosome, complete genome

contig-100_3482	568	N	0	0	NA	0	0	0	NA	Bacteroides vulgatus ATCC 8482, complete genome	gi|150002608|ref|NC_009614.1| Bacteroides vulgatus ATCC 8482 chromosome, complete genome	gi|150002608|ref|NC_009614.1| Bacteroides vulgatus ATCC 8482 chromosome, complete genome
contig-100_3483	568	N	1	0	NA	0	0	0	NA	Faecalibacterium prausnitzii SL3/3 draft genome	gi|479170689|ref|NC_021020.1| Faecalibacterium prausnitzii SL3/3 draft genome	gi|479170689|ref|NC_021020.1| Faecalibacterium prausnitzii SL3/3 draft genome
contig-100_3484	567	N	1	0	NA	0	0	0	NA	Uncultured organism clone 1041059765897 genomic sequence	NA	Uncultured organism clone 1041059765897 genomic sequence
contig-100_3485	567	N	0	0	NA	0	0	0	NA	NA	NA	NA
contig-100_3486	567	N	0	0	NA	0	0	0	NA	NA	NA	NA
contig-100_3487	567	N	0	0	NA	0	0	0	NA	NA	NA	NA
contig-100_3488	567	N	0	0	NA	0	0	0	NA	Haemophilus parainfluenzae T3T1 complete genome	gi|345428590|ref|NC_015964.1| Haemophilus parainfluenzae T3T1, complete genome	gi|345428590|ref|NC_015964.1| Haemophilus parainfluenzae T3T1, complete genome
contig-100_3489	567	N	0	0	NA	0	0	0	NA	NA	NA	NA
contig-100_349	2425	N	3	0	NA	0	0	0	NA	Faecalibacterium prausnitzii SL3/3 draft genome	gi|479170689|ref|NC_021020.1| Faecalibacterium prausnitzii SL3/3 draft genome	gi|479170689|ref|NC_021020.1| Faecalibacterium prausnitzii SL3/3 draft genome
contig-100_3490	566	N	0	0	NA	0	0	0	NA	NA	NA	NA
contig-100_3491	566	N	0	0	NA	0	0	0	NA	Uncultured bacterium clone HA0AAA9ZH05FM1 genomic sequence	NA	Uncultured bacterium clone HA0AAA9ZH05FM1 genomic sequence
contig-100_3492	566	N	0	0	NA	0	0	0	NA	NA	NA	NA

contig-100_3493	566	N	0	0	NA	0	0	0	NA	NA	NA	NA
contig-100_3494	566	N	2	0	NA	0	0	0	NA	Parabacteroides distasonis ATCC 8503, complete genome	gi|150006674|ref|NC_009615.1| Parabacteroides distasonis ATCC 8503 chromosome, complete genome	gi|150006674|ref|NC_009615.1| Parabacteroides distasonis ATCC 8503 chromosome, complete genome
contig-100_3496	566	N	0	0	NA	0	0	0	NA	Haemophilus parainfluenzae T3T1 complete genome	gi|345428590|ref|NC_015964.1| Haemophilus parainfluenzae T3T1, complete genome	gi|345428590|ref|NC_015964.1| Haemophilus parainfluenzae T3T1, complete genome
contig-100_3497	565	N	0	0	NA	0	0	0	NA	NA	NA	NA
contig-100_3498	565	N	0	0	NA	0	0	0	NA	NA	NA	NA
contig-100_3499	565	N	0	0	NA	0	0	0	NA	Uncultured organism clone VC1AB46TF genomic sequence	gi|325297172|ref|NC_015164.1| Bacteroides salanitronis DSM 18170 chromosome, complete genome	gi|325297172|ref|NC_015164.1| Bacteroides salanitronis DSM 18170 chromosome, complete genome
contig-100_3500	565	N	0	0	NA	0	0	0	NA	NA	NA	NA
contig-100_3501	565	N	0	0	NA	0	0	0	NA	Faecalibacterium prausnitzii SL3/3 draft genome	gi|479170689|ref|NC_021020.1| Faecalibacterium prausnitzii SL3/3 draft genome	gi|479170689|ref|NC_021020.1| Faecalibacterium prausnitzii SL3/3 draft genome
contig-100_3502	565	N	1	0	NA	0	0	0	NA	Uncultured organism clone 19 genomic sequence	gi|479192860|ref|NC_021035.1| Butyrate-producing bacterium SS3/4, complete genome	gi|479192860|ref|NC_021035.1| Butyrate-producing bacterium SS3/4, complete genome
contig-100_3503	565	N	0	0	NA	0	0	0	NA	NA	NA	NA
contig-100_3504	565	N	0	0	NA	0	0	0	NA	NA	NA	NA

contig-100_3507	564	N	0	0	NA	0	0	0	NA	Faecalibacterium prausnitzii L2/6 draft genome	gi|479208076|ref|NC_021042.1| Faecalibacterium prausnitzii L2-6, complete genome	gi|479208076|ref|NC_021042.1| Faecalibacterium prausnitzii L2-6, complete genome
contig-100_3508	564	N	0	0	NA	0	0	0	NA	NA	NA	NA
contig-100_3509	563	N	1	0	NA	0	0	0	NA	NA	NA	NA
contig-100_351	2403	N	3	0	NA	0	0	0	NA	NA	NA	NA
contig-100_3510	563	N	1	0	NA	0	0	0	NA	NA	NA	NA
contig-100_3512	563	N	0	0	NA	0	0	0	NA	NA	NA	NA
contig-100_3513	563	N	0	0	NA	0	0	0	NA	Bacteroides vulgatus ATCC 8482, complete genome	gi|150002608|ref|NC_009614.1| Bacteroides vulgatus ATCC 8482 chromosome, complete genome	gi|150002608|ref|NC_009614.1| Bacteroides vulgatus ATCC 8482 chromosome, complete genome
contig-100_3514	563	N	0	0	NA	0	0	0	NA	NA	NA	NA
contig-100_3515	563	N	0	0	NA	0	0	0	NA	NA	NA	NA
contig-100_3516	562	N	0	0	NA	0	0	0	NA	NA	NA	NA
contig-100_3518	562	N	1	0	NA	0	0	0	NA	Uncultured organism clone 1041059766717 genomic sequence	NA	Uncultured organism clone 1041059766717 genomic sequence
contig-100_3519	562	N	0	0	NA	0	0	0	NA	NA	NA	NA
contig-100_352	2402	N	1	1	Sipho	0	0	1	NA	Bacteroides vulgatus ATCC 8482, complete genome	gi|150002608|ref|NC_009614.1| Bacteroides vulgatus ATCC 8482 chromosome, complete genome	gi|150002608|ref|NC_009614.1| Bacteroides vulgatus ATCC 8482 chromosome, complete genome
contig-100_3520	562	N	0	0	NA	0	0	0	NA	NA	gi|121607004|ref|NC_008786.1| Verminephrobacter eiseniae EF01-2 chromosome, complete genome	gi|121607004|ref|NC_008786.1| Verminephrobacter eiseniae EF01-2 chromosome, complete genome

contig-100_3521	562	N	0	0	NA	0	0	0	NA	Uncultured bacterium clone HA0AAA14ZB12FM1 genomic sequence	NA	Uncultured bacterium clone HA0AAA14ZB12FM1 genomic sequence
contig-100_3522	561	N	0	0	NA	0	0	0	NA	NA	NA	NA
contig-100_3523	561	N	0	0	NA	0	0	0	NA	Unidentified phage clone 1013_scaffold47 genomic sequence	NA	Unidentified phage clone 1013_scaffold47 genomic sequence
contig-100_3524	561	N	0	0	NA	0	0	0	NA	NA	NA	NA
contig-100_3525	561	N	0	0	NA	0	0	0	NA	NA	NA	NA
contig-100_3526	561	N	0	0	NA	0	0	0	NA	Streptococcus phage Abc2, complete genome	NA	Streptococcus phage Abc2, complete genome
contig-100_3527	561	N	0	0	NA	0	0	0	NA	NA	NA	NA
contig-100_3529	560	N	0	0	NA	0	0	0	NA	Uncultured organism clone 1041059767488 genomic sequence	NA	Uncultured organism clone 1041059767488 genomic sequence
contig-100_353	2399	N	2	0	NA	0	0	0	NA	Faecalibacterium prausnitzii SL3/3 draft genome	gi|479170689|ref|NC_021020.1| Faecalibacterium prausnitzii SL3/3 draft genome	gi|479170689|ref|NC_021020.1| Faecalibacterium prausnitzii SL3/3 draft genome
contig-100_3530	560	N	0	0	NA	0	0	0	NA	Escherichia coli str. K-12 substr. MG1655, complete genome	gi|388476123|ref|NC_007779.1| Escherichia coli str. K-12 substr. W3110, complete genome	gi|388476123|ref|NC_007779.1| Escherichia coli str. K-12 substr. W3110, complete genome
contig-100_3531	560	N	0	0	NA	0	0	0	NA	NA	NA	NA
contig-100_3532	560	N	1	0	NA	0	0	0	NA	Uncultured bacterium clone LM0ACA13ZA08RM1 genomic sequence	NA	Uncultured bacterium clone LM0ACA13ZA08RM1 genomic sequence

contig-100_3533	560	N	0	0	NA	0	0	0	NA	NA	NA	NA
contig-100_3534	560	N	0	0	NA	0	0	0	NA	NA	NA	NA
contig-100_3535	559	N	1	0	NA	0	0	0	NA	NA	NA	NA
contig-100_3536	559	N	0	0	NA	0	0	0	NA	NA	NA	NA
contig-100_3537	559	N	0	0	NA	0	0	0	NA	Bacteroides salanitronis DSM 18170, complete genome	gi|325297172|ref|NC_015164.1| Bacteroides salanitronis DSM 18170 chromosome, complete genome	gi|325297172|ref|NC_015164.1| Bacteroides salanitronis DSM 18170 chromosome, complete genome
contig-100_3539	559	N	0	0	NA	0	0	0	NA	Faecalibacterium prausnitzii SL3/3 draft genome	gi|479170689|ref|NC_021020.1| Faecalibacterium prausnitzii SL3/3 draft genome	gi|479170689|ref|NC_021020.1| Faecalibacterium prausnitzii SL3/3 draft genome
contig-100_354	2398	N	2	0	NA	0	0	0	NA	NA	NA	NA
contig-100_3540	559	N	0	0	NA	0	0	0	NA	NA	NA	NA
contig-100_3541	558	N	0	0	NA	0	0	0	NA	Odoribacter splanchnicus DSM 20712, complete genome	gi|325278757|ref|NC_015160.1| Odoribacter splanchnicus DSM 20712 chromosome, complete genome	gi|325278757|ref|NC_015160.1| Odoribacter splanchnicus DSM 20712 chromosome, complete genome
contig-100_3542	558	N	0	0	NA	0	0	0	NA	NA	NA	NA
contig-100_3543	558	N	0	0	NA	0	0	0	NA	Faecalibacterium prausnitzii SL3/3 draft genome	gi|479170689|ref|NC_021020.1| Faecalibacterium prausnitzii SL3/3 draft genome	gi|479170689|ref|NC_021020.1| Faecalibacterium prausnitzii SL3/3 draft genome
contig-100_3544	558	N	0	0	NA	0	0	0	NA	NA	NA	NA
contig-100_3545	558	N	0	0	NA	0	0	0	NA	NA	NA	NA
contig-100_3546	558	N	1	0	NA	0	0	0	NA	NA	NA	NA
contig-100_3547	558	N	1	0	NA	0	0	0	NA	NA	NA	NA

contig-100_3549	557	N	0	0	NA	0	0	0	NA	Faecalibacterium prausnitzii SL3/3 draft genome	gi|479170689|ref|NC_021020.1| Faecalibacterium prausnitzii SL3/3 draft genome	gi|479170689|ref|NC_021020.1| Faecalibacterium prausnitzii SL3/3 draft genome
contig-100_355	2398	N	1	0	NA	0	0	0	NA	Haemophilus parainfluenzae T3T1 complete genome	gi|345428590|ref|NC_015964.1| Haemophilus parainfluenzae T3T1, complete genome	gi|345428590|ref|NC_015964.1| Haemophilus parainfluenzae T3T1, complete genome
contig-100_3551	557	N	0	0	NA	0	0	0	NA	NA	gi|568136993|ref|NC_023064.1| Pseudomonas sp. TKP, complete genome	gi|568136993|ref|NC_023064.1| Pseudomonas sp. TKP, complete genome
contig-100_3552	557	N	1	0	NA	0	0	0	NA	Faecalibacterium prausnitzii SL3/3 draft genome	gi|479170689|ref|NC_021020.1| Faecalibacterium prausnitzii SL3/3 draft genome	gi|479170689|ref|NC_021020.1| Faecalibacterium prausnitzii SL3/3 draft genome
contig-100_3554	557	N	0	0	NA	0	0	0	NA	NA	NA	NA
contig-100_3555	557	N	0	0	NA	0	0	0	NA	NA	NA	NA
contig-100_3556	557	N	1	0	NA	0	0	0	NA	Bacteroides vulgatus ATCC 8482, complete genome	gi|150002608|ref|NC_009614.1| Bacteroides vulgatus ATCC 8482 chromosome, complete genome	gi|150002608|ref|NC_009614.1| Bacteroides vulgatus ATCC 8482 chromosome, complete genome
contig-100_3557	556	N	0	0	NA	0	0	0	NA	NA	NA	NA
contig-100_3558	556	N	0	0	NA	0	0	0	NA	Uncultured bacterium clone LM0ABA40ZB01FM1 genomic sequence	NA	Uncultured bacterium clone LM0ABA40ZB01FM1 genomic sequence

contig-100_3559	556	N	0	0	NA	0	0	0	NA	Clostridiales sp. SSC/2 draft genome	gi|479158859|ref|NC_021016.1| Butyrate-producing bacterium SSC/2, complete genome	gi|479158859|ref|NC_021016.1| Butyrate-producing bacterium SSC/2, complete genome
contig-100_356	2397	N	3	1	Sipho	0	0	0	NA	NA	NA	NA
contig-100_3561	556	N	1	0	NA	0	0	0	NA	NA	NA	NA
contig-100_3562	556	N	0	0	NA	0	0	0	NA	Eubacterium rectale DSM 17629 draft genome	gi|479140210|ref|NC_021010.1| Eubacterium rectale DSM 17629 draft genome	gi|479140210|ref|NC_021010.1| Eubacterium rectale DSM 17629 draft genome
contig-100_3563	555	N	0	0	NA	0	0	0	NA	NA	NA	NA
contig-100_3564	555	N	0	0	NA	0	0	0	NA	Bacteroides salanitronis DSM 18170, complete genome	gi|325297172|ref|NC_015164.1| Bacteroides salanitronis DSM 18170 chromosome, complete genome	gi|325297172|ref|NC_015164.1| Bacteroides salanitronis DSM 18170 chromosome, complete genome
contig-100_3565	555	N	0	0	NA	0	0	0	NA	Bacteroides vulgatus ATCC 8482, complete genome	gi|150002608|ref|NC_009614.1| Bacteroides vulgatus ATCC 8482 chromosome, complete genome	gi|150002608|ref|NC_009614.1| Bacteroides vulgatus ATCC 8482 chromosome, complete genome
contig-100_3566	555	N	0	0	NA	0	0	0	NA	NA	NA	NA
contig-100_3567	555	N	1	0	NA	0	0	0	NA	NA	NA	NA
contig-100_3568	555	N	1	0	NA	0	0	0	NA	Faecalibacterium prausnitzii L2/6 draft genome	gi|479208076|ref|NC_021042.1| Faecalibacterium prausnitzii L2-6, complete genome	gi|479208076|ref|NC_021042.1| Faecalibacterium prausnitzii L2-6, complete genome
contig-100_3569	555	N	0	0	NA	0	0	0	NA	NA	NA	NA
contig-100_357	2395	N	1	0	NA	0	0	0	NA	NA	NA	NA
contig-100_3570	555	N	0	0	NA	0	0	0	NA	NA	NA	NA
contig-100_3571	555	N	0	0	NA	0	0	0	NA	NA	NA	NA

contig-100_3572	555	N	1	0	NA	0	0	0	NA	Bacteroides vulgatus ATCC 8482, complete genome	gi|150002608|ref|NC_009614.1| Bacteroides vulgatus ATCC 8482 chromosome, complete genome	gi|150002608|ref|NC_009614.1| Bacteroides vulgatus ATCC 8482 chromosome, complete genome
contig-100_3574	554	N	0	0	NA	0	0	0	NA	NA	NA	NA
contig-100_3575	554	N	0	0	NA	0	0	0	NA	NA	NA	NA
contig-100_3576	554	N	0	0	NA	0	0	0	NA	NA	NA	NA
contig-100_3577	554	N	0	0	NA	0	0	0	NA	Uncultured organism clone VC1BS79TF genomic sequence	NA	Uncultured organism clone VC1BS79TF genomic sequence
contig-100_3578	553	N	0	0	NA	0	0	0	NA	Faecalibacterium prausnitzii L2/6 draft genome	gi|479208076|ref|NC_021042.1| Faecalibacterium prausnitzii L2-6, complete genome	gi|479208076|ref|NC_021042.1| Faecalibacterium prausnitzii L2-6, complete genome
contig-100_3579	553	N	0	0	NA	0	0	0	NA	NA	NA	NA
contig-100_3580	553	N	0	0	NA	0	0	0	NA	Roseburia hominis A2-183, complete genome	gi|347530298|ref|NC_015977.1| Roseburia hominis A2-183 chromosome, complete genome	gi|347530298|ref|NC_015977.1| Roseburia hominis A2-183 chromosome, complete genome
contig-100_3581	553	N	0	0	NA	0	0	0	NA	Bacteroides helcogenes P 36-108, complete genome	gi|319899888|ref|NC_014933.1| Bacteroides helcogenes P 36-108 chromosome, complete genome	gi|319899888|ref|NC_014933.1| Bacteroides helcogenes P 36-108 chromosome, complete genome
contig-100_3583	553	N	0	0	NA	0	0	0	NA	NA	NA	NA
contig-100_3584	553	N	0	0	NA	0	0	0	NA	NA	NA	NA
contig-100_3586	553	N	0	0	NA	0	0	0	NA	NA	NA	NA
contig-100_3587	553	N	0	0	NA	0	0	0	NA	NA	NA	NA
contig-100_3588	553	N	0	0	NA	0	0	0	NA	NA	NA	NA

contig-100_359	2389	N	3	0	NA	0	0	0	NA	Odoribacter splanchnicus DSM 20712, complete genome	gi|325278757|ref|NC_015160.1| Odoribacter splanchnicus DSM 20712 chromosome, complete genome	gi|325278757|ref|NC_015160.1| Odoribacter splanchnicus DSM 20712 chromosome, complete genome
contig-100_3590	553	N	0	0	NA	0	0	0	NA	Ethanoligenens harbinense YUAN-3, complete genome	gi|317131008|ref|NC_014828.1| Ethanoligenens harbinense YUAN-3 chromosome, complete genome	gi|317131008|ref|NC_014828.1| Ethanoligenens harbinense YUAN-3 chromosome, complete genome
contig-100_3591	553	N	0	0	NA	0	0	0	NA	NA	NA	NA
contig-100_3592	553	N	0	0	NA	0	0	0	NA	NA	NA	NA
contig-100_3593	553	N	0	0	NA	0	0	0	NA	NA	NA	NA
contig-100_3594	552	N	0	0	NA	0	0	0	NA	Faecalibacterium prausnitzii SL3/3 draft genome	gi|479170689|ref|NC_021020.1| Faecalibacterium prausnitzii SL3/3 draft genome	gi|479170689|ref|NC_021020.1| Faecalibacterium prausnitzii SL3/3 draft genome
contig-100_3595	552	N	0	0	NA	0	0	0	NA	Bacteroides salanitronis DSM 18170, complete genome	gi|325297172|ref|NC_015164.1| Bacteroides salanitronis DSM 18170 chromosome, complete genome	gi|325297172|ref|NC_015164.1| Bacteroides salanitronis DSM 18170 chromosome, complete genome
contig-100_3596	552	N	0	0	NA	0	0	0	NA	NA	NA	NA
contig-100_3597	552	N	0	0	NA	0	0	0	NA	Bacteroides vulgatus ATCC 8482, complete genome	gi|150002608|ref|NC_009614.1| Bacteroides vulgatus ATCC 8482 chromosome, complete genome	gi|150002608|ref|NC_009614.1| Bacteroides vulgatus ATCC 8482 chromosome, complete genome

contig-100_3598	551	N	0	0	NA	0	0	0	NA	Escherichia coli str. K-12 substr. MG1655, complete genome	gi|386703215|ref|NC_017663.1| Escherichia coli P12b chromosome, complete genome	gi|386703215|ref|NC_017663.1| Escherichia coli P12b chromosome, complete genome
contig-100_3599	551	N	0	0	NA	0	0	0	NA	NA	NA	NA
contig-100_36	9492	N	7	3	Sipho	0	0	3	NA	Klebsiella pneumoniae JM45, complete genome	gi|530627845|ref|NC_022082.1| Klebsiella pneumoniae JM45, complete genome	gi|530627845|ref|NC_022082.1| Klebsiella pneumoniae JM45, complete genome
contig-100_3600	551	N	0	0	NA	0	0	0	NA	NA	NA	NA
contig-100_3601	551	N	2	0	NA	0	0	0	NA	NA	NA	NA
contig-100_3602	551	N	0	0	NA	0	0	0	NA	NA	NA	NA
contig-100_3603	551	N	0	0	NA	0	0	0	NA	NA	NA	NA
contig-100_3604	550	N	1	0	NA	0	0	0	NA	NA	NA	NA
contig-100_3607	550	N	1	0	NA	0	0	0	NA	NA	NA	NA
contig-100_3608	550	N	1	0	NA	0	0	0	NA	Faecalibacterium prausnitzii L2/6 draft genome	gi|479208076|ref|NC_021042.1| Faecalibacterium prausnitzii L2-6, complete genome	gi|479208076|ref|NC_021042.1| Faecalibacterium prausnitzii L2-6, complete genome
contig-100_3609	550	N	0	0	NA	0	0	0	NA	Uncultured bacterium clone LM0ABA28ZF01FM1 genomic sequence	NA	Uncultured bacterium clone LM0ABA28ZF01FM1 genomic sequence
contig-100_3610	550	N	0	0	NA	0	0	0	NA	NA	NA	NA
contig-100_3611	550	N	0	0	NA	0	0	0	NA	NA	NA	NA
contig-100_3612	550	N	0	0	NA	0	0	0	NA	NA	NA	NA
contig-100_3613	550	N	0	0	NA	0	0	0	NA	NA	NA	NA
contig-100_3614	550	N	0	0	NA	0	0	0	NA	Clostridiales sp. SSC/2 draft genome	gi|479158859|ref|NC_021016.1| Butyrate-producing bacterium SSC/2, complete genome	gi|479158859|ref|NC_021016.1| Butyrate-producing bacterium SSC/2, complete genome

contig-100_3615	550	N	0	0	NA	0	0	0	NA	NA	NA	NA
contig-100_3616	550	N	0	0	NA	0	0	0	NA	Uncultured organism clone VC1CG39TR genomic sequence	NA	Uncultured organism clone VC1CG39TR genomic sequence
contig-100_3617	549	N	1	0	NA	0	0	0	NA	Odoribacter splanchnicus DSM 20712, complete genome	gi|325278757|ref|NC_015160.1| Odoribacter splanchnicus DSM 20712 chromosome, complete genome	gi|325278757|ref|NC_015160.1| Odoribacter splanchnicus DSM 20712 chromosome, complete genome
contig-100_3618	549	N	0	0	NA	0	0	0	NA	NA	NA	NA
contig-100_3620	549	N	1	0	NA	0	0	0	NA	NA	NA	NA
contig-100_3621	549	N	0	0	NA	0	0	0	NA	complete chromosome Acholeplasma palmae	gi|651865246|ref|NC_022538.1| complete chromosome Acholeplasma palmae	gi|651865246|ref|NC_022538.1| complete chromosome Acholeplasma palmae
contig-100_3622	549	N	1	0	NA	0	0	0	NA	NA	NA	NA
contig-100_3623	548	N	0	0	NA	0	0	0	NA	Uncultured organism clone VC1C529TF genomic sequence	NA	Uncultured organism clone VC1C529TF genomic sequence
contig-100_3624	548	N	1	0	NA	0	0	0	NA	NA	NA	NA
contig-100_3625	548	N	0	0	NA	0	0	0	NA	NA	NA	NA
contig-100_3626	548	N	0	0	NA	0	0	0	NA	NA	NA	NA
contig-100_3627	548	N	0	0	NA	0	0	0	NA	NA	NA	NA
contig-100_3628	548	N	1	0	NA	0	0	0	NA	NA	NA	NA
contig-100_3629	547	N	0	0	NA	0	0	0	NA	NA	NA	NA
contig-100_3631	547	N	0	0	NA	0	0	0	NA	NA	NA	NA
contig-100_3632	547	N	0	0	NA	0	0	0	NA	Faecalibacterium prausnitzii SL3/3 draft genome	gi|479170689|ref|NC_021020.1| Faecalibacterium prausnitzii SL3/3 draft genome	gi|479170689|ref|NC_021020.1| Faecalibacterium prausnitzii SL3/3 draft genome
contig-100_3633	547	N	0	0	NA	0	0	0	NA	NA	NA	NA
contig-100_3634	547	N	0	0	NA	0	0	0	NA	NA	NA	NA
contig-100_3635	547	N	0	0	NA	0	0	0	NA	NA	NA	NA

contig-100_3636	547	N	0	0	NA	0	0	0	NA	NA	NA	NA
contig-100_3637	546	N	0	0	NA	0	0	0	NA	NA	NA	NA
contig-100_3638	546	N	0	0	NA	0	0	0	NA	NA	NA	NA
contig-100_3639	546	N	0	0	NA	0	0	0	NA	NA	NA	NA
contig-100_3640	546	N	1	0	NA	0	0	0	NA	NA	NA	NA
contig-100_3641	545	N	0	0	NA	0	0	0	NA	Ruminococcus torques L2-14 draft genome	gi|479155735|ref|NC_021015.1| Ruminococcus torques L2-14 draft genome	gi|479155735|ref|NC_021015.1| Ruminococcus torques L2-14 draft genome
contig-100_3642	545	N	0	0	NA	0	0	0	NA	NA	NA	NA
contig-100_3643	545	N	0	0	NA	0	0	0	NA	Bacteriophage EJ-1 proviral DNA, complete genome	NA	Bacteriophage EJ-1 proviral DNA, complete genome
contig-100_3645	545	N	0	0	NA	0	0	0	NA	NA	NA	NA
contig-100_3646	545	N	2	0	NA	0	0	0	NA	NA	NA	NA
contig-100_3647	545	N	0	0	NA	0	0	0	NA	NA	NA	NA
contig-100_3648	545	N	0	0	NA	0	0	0	NA	Faecalibacterium prausnitzii SL3/3 draft genome	gi|479170689|ref|NC_021020.1| Faecalibacterium prausnitzii SL3/3 draft genome	gi|479170689|ref|NC_021020.1| Faecalibacterium prausnitzii SL3/3 draft genome
contig-100_3649	545	N	1	0	NA	0	0	0	NA	NA	NA	NA
contig-100_365	2368	N	2	1	NA	0	0	0	NA	Faecalibacterium prausnitzii SL3/3 draft genome	gi|479170689|ref|NC_021020.1| Faecalibacterium prausnitzii SL3/3 draft genome	gi|479170689|ref|NC_021020.1| Faecalibacterium prausnitzii SL3/3 draft genome
contig-100_3651	545	N	1	0	NA	0	0	0	NA	Uncultured organism clone VC1DH31TR genomic sequence	NA	Uncultured organism clone VC1DH31TR genomic sequence
contig-100_3652	544	N	0	0	NA	0	0	0	NA	Uncultured organism clone VC1C391TR genomic sequence	NA	Uncultured organism clone VC1C391TR genomic sequence

contig-100_3653	544	N	1	0	NA	0	0	0	NA	Faecalibacterium prausnitzii L2/6 draft genome	gi|479208076|ref|NC_021042.1| Faecalibacterium prausnitzii L2-6, complete genome	gi|479208076|ref|NC_021042.1| Faecalibacterium prausnitzii L2-6, complete genome
contig-100_3654	544	N	0	0	NA	0	0	0	NA	NA	NA	NA
contig-100_3655	544	N	0	0	NA	0	0	0	NA	Propionibacterium acnes ATCC 11828, complete genome	gi|386069650|ref|NC_017550.1| Propionibacterium acnes ATCC 11828 chromosome, complete genome	gi|386069650|ref|NC_017550.1| Propionibacterium acnes ATCC 11828 chromosome, complete genome
contig-100_3656	544	N	1	0	NA	0	0	0	NA	NA	NA	NA
contig-100_3657	544	N	1	0	NA	0	0	0	NA	NA	NA	NA
contig-100_3658	543	N	0	0	NA	0	0	0	NA	NA	NA	NA
contig-100_366	2368	N	1	0	NA	0	0	0	NA	NA	NA	NA
contig-100_3660	543	N	0	0	NA	0	0	0	NA	NA	NA	NA
contig-100_3661	543	N	0	0	NA	0	0	0	NA	NA	NA	NA
contig-100_3662	543	N	0	0	NA	0	0	0	NA	Faecalibacterium prausnitzii L2/6 draft genome	gi|479208076|ref|NC_021042.1| Faecalibacterium prausnitzii L2-6, complete genome	gi|479208076|ref|NC_021042.1| Faecalibacterium prausnitzii L2-6, complete genome
contig-100_3663	543	N	0	0	NA	0	0	0	NA	NA	NA	NA
contig-100_3664	543	N	0	0	NA	0	0	0	NA	NA	NA	NA
contig-100_3666	543	N	0	0	NA	0	0	0	NA	Klebsiella pneumoniae subsp. pneumoniae MGH 78578, complete sequence	gi|550443072|ref|NC_022566.1| Klebsiella pneumoniae CG43, complete genome	gi|550443072|ref|NC_022566.1| Klebsiella pneumoniae CG43, complete genome
contig-100_3667	543	N	0	0	NA	0	0	0	NA	NA	NA	NA
contig-100_3668	542	N	0	0	NA	0	0	0	NA	NA	NA	NA
contig-100_3671	542	N	0	0	NA	0	0	0	NA	NA	NA	NA
contig-100_3672	542	N	0	0	NA	0	0	0	NA	NA	NA	NA

contig-100_3673	542	N	0	0	NA	0	0	0	NA	Eubacterium eligens ATCC 27750 plasmid, complete sequence	gi|238921767|ref|NC_012780.1| Eubacterium eligens ATCC 27750 plasmid unnamed, complete sequence	gi|238921767|ref|NC_012780.1| Eubacterium eligens ATCC 27750 plasmid unnamed, complete sequence
contig-100_3674	542	N	0	0	NA	0	0	0	NA	Eubacterium rectale DSM 17629 draft genome	gi|479140210|ref|NC_021010.1| Eubacterium rectale DSM 17629 draft genome	gi|479140210|ref|NC_021010.1| Eubacterium rectale DSM 17629 draft genome
contig-100_3675	542	N	0	0	NA	0	0	0	NA	NA	NA	NA
contig-100_3676	542	N	0	0	NA	0	0	0	NA	NA	NA	NA
contig-100_3677	542	N	0	0	NA	0	0	0	NA	NA	NA	NA
contig-100_3679	541	N	0	0	NA	0	0	0	NA	NA	NA	NA
contig-100_368	2361	N	1	0	NA	0	0	0	NA	NA	NA	NA
contig-100_3680	541	N	0	0	NA	0	0	0	NA	NA	NA	NA
contig-100_3681	541	N	0	0	NA	0	0	0	NA	NA	NA	NA
contig-100_3682	541	N	0	0	NA	0	0	0	NA	Haemophilus parainfluenzae T3T1 complete genome	gi|345428590|ref|NC_015964.1| Haemophilus parainfluenzae T3T1, complete genome	gi|345428590|ref|NC_015964.1| Haemophilus parainfluenzae T3T1, complete genome
contig-100_3683	541	N	0	0	NA	0	0	0	NA	Roseburia hominis A2-183, complete genome	gi|347530298|ref|NC_015977.1| Roseburia hominis A2-183 chromosome, complete genome	gi|347530298|ref|NC_015977.1| Roseburia hominis A2-183 chromosome, complete genome
contig-100_3684	541	N	0	0	NA	0	0	0	NA	NA	NA	NA
contig-100_3685	540	N	0	0	NA	0	0	0	NA	NA	NA	NA
contig-100_3686	540	N	0	0	NA	0	0	0	NA	Faecalibacterium prausnitzii SL3/3 draft genome	gi|479170689|ref|NC_021020.1| Faecalibacterium prausnitzii SL3/3 draft genome	gi|479170689|ref|NC_021020.1| Faecalibacterium prausnitzii SL3/3 draft genome

contig-100_3688	539	N	0	0	NA	0	0	0	NA	Bacteroides vulgatus ATCC 8482, complete genome	gi|150002608|ref|NC_009614.1| Bacteroides vulgatus ATCC 8482 chromosome, complete genome	gi|150002608|ref|NC_009614.1| Bacteroides vulgatus ATCC 8482 chromosome, complete genome
contig-100_369	2357	N	3	0	NA	0	0	0	NA	NA	NA	NA
contig-100_3690	539	N	0	0	NA	0	0	0	NA	Uncultured organism clone 20 genomic sequence	gi|479155735|ref|NC_021015.1| Ruminococcus torques L2-14 draft genome	gi|479155735|ref|NC_021015.1| Ruminococcus torques L2-14 draft genome
contig-100_3691	539	N	0	0	NA	0	0	0	NA	Haemophilus parainfluenzae T3T1 complete genome	gi|345428590|ref|NC_015964.1| Haemophilus parainfluenzae T3T1, complete genome	gi|345428590|ref|NC_015964.1| Haemophilus parainfluenzae T3T1, complete genome
contig-100_3693	539	N	0	0	NA	0	0	0	NA	NA	NA	NA
contig-100_3694	539	N	1	0	NA	0	0	0	NA	Parabacteroides distasonis ATCC 8503, complete genome	gi|150006674|ref|NC_009615.1| Parabacteroides distasonis ATCC 8503 chromosome, complete genome	gi|150006674|ref|NC_009615.1| Parabacteroides distasonis ATCC 8503 chromosome, complete genome
contig-100_3695	539	N	0	0	NA	0	0	0	NA	NA	NA	NA
contig-100_3696	538	N	0	0	NA	0	0	0	NA	NA	NA	NA
contig-100_3697	538	N	0	0	NA	0	0	0	NA	Enterobacteria phage HK629, complete genome	gi|253771435|ref|NC_012947.1| Escherichia coli 'BL21-Gold(DE3)pLysS AG' chromosome, complete genome	gi|253771435|ref|NC_012947.1| Escherichia coli 'BL21-Gold(DE3)pLysS AG' chromosome, complete genome
contig-100_3698	538	N	1	0	NA	0	0	0	NA	NA	NA	NA
contig-100_3699	538	N	1	0	NA	0	0	0	NA	NA	NA	NA

contig-100_370	2355	N	1	0	NA	0	0	0	NA	Haemophilus parainfluenzae T3T1 complete genome	gi|345428590|ref|NC_015964.1| Haemophilus parainfluenzae T3T1, complete genome	gi|345428590|ref|NC_015964.1| Haemophilus parainfluenzae T3T1, complete genome
contig-100_3700	538	N	0	0	NA	0	0	0	NA	NA	NA	NA
contig-100_3701	538	N	0	0	NA	0	0	0	NA	NA	NA	NA
contig-100_3702	537	N	1	0	NA	0	0	0	NA	Bacteroides vulgatus ATCC 8482, complete genome	gi|150002608|ref|NC_009614.1| Bacteroides vulgatus ATCC 8482 chromosome, complete genome	gi|150002608|ref|NC_009614.1| Bacteroides vulgatus ATCC 8482 chromosome, complete genome
contig-100_3703	537	N	0	0	NA	0	0	0	NA	Uncultured organism clone 20 genomic sequence	gi|479170689|ref|NC_021020.1| Faecalibacterium prausnitzii SL3/3 draft genome	gi|479170689|ref|NC_021020.1| Faecalibacterium prausnitzii SL3/3 draft genome
contig-100_3704	537	N	2	0	NA	0	0	0	NA	NA	NA	NA
contig-100_3705	537	N	0	0	NA	0	0	0	NA	Bacteroides vulgatus ATCC 8482, complete genome	gi|150002608|ref|NC_009614.1| Bacteroides vulgatus ATCC 8482 chromosome, complete genome	gi|150002608|ref|NC_009614.1| Bacteroides vulgatus ATCC 8482 chromosome, complete genome
contig-100_3707	536	N	1	0	NA	0	0	0	NA	Uncultured bacterium clone LM0ABA7ZA10RM1 genomic sequence	gi|319899888|ref|NC_014933.1| Bacteroides helcogenes P 36-108 chromosome, complete genome	gi|319899888|ref|NC_014933.1| Bacteroides helcogenes P 36-108 chromosome, complete genome
contig-100_3709	536	N	0	0	NA	0	0	0	NA	NA	NA	NA
contig-100_371	2352	N	2	0	NA	0	1	1	NA	NA	NA	NA
contig-100_3710	536	N	0	0	NA	0	0	0	NA	NA	NA	NA
contig-100_3711	536	N	0	0	NA	0	0	0	NA	NA	NA	NA
contig-100_3712	536	N	0	0	NA	0	0	0	NA	NA	NA	NA

contig-100_3713	536	N	0	0	NA	0	0	0	NA	NA	NA	NA
contig-100_3714	535	N	0	0	NA	0	0	0	NA	NA	NA	NA
contig-100_3715	535	N	0	0	NA	0	0	0	NA	Uncultured bacterium clone HA0AAA9ZC05RM1 genomic sequence	NA	Uncultured bacterium clone HA0AAA9ZC 05RM1 genomic sequence
contig-100_3716	535	N	0	0	NA	0	0	0	NA	Bacteroides vulgatus ATCC 8482, complete genome	gi|150002608|ref|N C_009614.1| Bacteroides vulgatus ATCC 8482 chromosome, complete genome	gi|150002608|r ef|NC_009614 .1| Bacteroides vulgatus ATCC 8482 chromosome, complete genome
contig-100_3717	535	N	0	0	NA	0	0	0	NA	NA	NA	NA
contig-100_3718	535	N	0	0	NA	0	0	0	NA	NA	NA	NA
contig-100_3719	534	N	0	0	NA	0	0	0	NA	NA	NA	NA
contig-100_3720	534	N	0	0	NA	0	0	0	NA	NA	NA	NA
contig-100_3721	534	N	0	0	NA	0	0	0	NA	Bacteroides vulgatus ATCC 8482, complete genome	gi|150002608|ref|N C_009614.1| Bacteroides vulgatus ATCC 8482 chromosome, complete genome	gi|150002608|r ef|NC_009614 .1| Bacteroides vulgatus ATCC 8482 chromosome, complete genome
contig-100_3722	534	N	0	0	NA	0	0	0	NA	NA	NA	NA
contig-100_3724	534	N	0	0	NA	0	0	0	NA	Cryptobacterium curtum DSM 15641, complete genome	gi|256826460|ref|N C_013170.1| Cryptobacterium curtum DSM 15641 chromosome, complete genome	gi|256826460|r ef|NC_013170 .1| Cryptobacteriu m curtum DSM 15641 chromosome, complete genome
contig-100_3725	533	N	1	0	NA	0	0	0	NA	NA	NA	NA
contig-100_3727	533	N	0	0	NA	0	0	0	NA	NA	NA	NA
contig-100_3730	532	N	0	0	NA	0	0	0	NA	NA	NA	NA

contig-100_3731	532	N	0	0	NA	0	0	0	NA	Ruminococcus torques L2-14 draft genome	gi|479155735|ref|NC_021015.1| Ruminococcus torques L2-14 draft genome	gi|479155735|ref|NC_021015.1| Ruminococcus torques L2-14 draft genome
contig-100_3732	532	N	0	0	NA	0	0	0	NA	NA	NA	NA
contig-100_3733	531	N	0	0	NA	0	0	0	NA	Uncultured organism clone VC1CN40TR genomic sequence	gi|319899888|ref|NC_014933.1| Bacteroides helcogenes P 36-108 chromosome, complete genome	gi|319899888|ref|NC_014933.1| Bacteroides helcogenes P 36-108 chromosome, complete genome
contig-100_3734	531	N	0	0	NA	0	0	0	NA	Ruminococcus bromii L2-63 draft genome	gi|479150083|ref|NC_021013.1| Ruminococcus bromii L2-63 draft genome	gi|479150083|ref|NC_021013.1| Ruminococcus bromii L2-63 draft genome
contig-100_3735	531	N	2	0	NA	0	0	0	NA	NA	NA	NA
contig-100_3736	531	N	0	0	NA	0	0	0	NA	NA	NA	NA
contig-100_3737	531	N	0	0	NA	0	0	0	NA	NA	NA	NA
contig-100_3738	531	N	0	0	NA	0	0	0	NA	Bacteroides vulgatus ATCC 8482, complete genome	gi|150002608|ref|NC_009614.1| Bacteroides vulgatus ATCC 8482 chromosome, complete genome	gi|150002608|ref|NC_009614.1| Bacteroides vulgatus ATCC 8482 chromosome, complete genome
contig-100_3739	530	N	0	0	NA	0	0	0	NA	NA	NA	NA
contig-100_3740	530	N	0	0	NA	0	0	0	NA	NA	NA	NA
contig-100_3741	530	N	0	0	NA	0	0	0	NA	Clostridiales sp. SM4/1 draft genome	gi|479181986|ref|NC_021024.1| Butyrate-producing bacterium SM4/1, complete genome	gi|479181986|ref|NC_021024.1| Butyrate-producing bacterium SM4/1, complete genome
contig-100_3742	530	N	0	0	NA	0	0	0	NA	NA	NA	NA
contig-100_3744	530	N	0	0	NA	0	0	0	NA	NA	NA	NA
contig-100_3746	529	N	1	0	NA	0	0	0	NA	NA	NA	NA

contig-100_3747	529	N	0	0	NA	0	0	0	NA	NA	NA	NA
contig-100_3748	529	N	0	0	NA	0	0	0	NA	NA	NA	NA
contig-100_3749	528	N	0	0	NA	0	0	0	NA	NA	NA	NA
contig-100_375	2348	N	1	0	NA	0	0	0	NA	NA	NA	NA
contig-100_3750	528	N	0	0	NA	0	0	0	NA	NA	NA	NA
contig-100_3752	528	N	0	0	NA	0	0	0	NA	Coprococcus sp. ART55/1 draft genome	gi|479166807|ref|NC_021018.1| Coprococcus sp. ART55/1 draft genome	gi|479166807|ref|NC_021018.1| Coprococcus sp. ART55/1 draft genome
contig-100_3753	528	N	0	0	NA	0	0	0	NA	Escherichia coli str. K-12 substr. MG1655, complete genome	gi|388476123|ref|NC_007779.1| Escherichia coli str. K-12 substr. W3110, complete genome	gi|388476123|ref|NC_007779.1| Escherichia coli str. K-12 substr. W3110, complete genome
contig-100_3754	528	N	0	0	NA	0	0	0	NA	NA	NA	NA
contig-100_3755	528	N	0	0	NA	0	0	0	NA	Bacteroides thetaiotaomicron VPI-5482, complete genome	gi|29345410|ref|NC_004663.1| Bacteroides thetaiotaomicron VPI-5482 chromosome, complete genome	gi|29345410|ref|NC_004663.1| Bacteroides thetaiotaomicron VPI-5482 chromosome, complete genome
contig-100_3756	528	N	0	0	NA	0	0	0	NA	NA	NA	NA
contig-100_3757	528	N	0	0	NA	0	0	0	NA	Clostridiales sp. SS3/4 draft genome	gi|479192860|ref|NC_021035.1| Butyrate-producing bacterium SS3/4, complete genome	gi|479192860|ref|NC_021035.1| Butyrate-producing bacterium SS3/4, complete genome
contig-100_3758	527	N	0	0	NA	0	0	0	NA	NA	NA	NA
contig-100_3759	527	N	0	0	NA	0	0	0	NA	NA	NA	NA
contig-100_3761	527	N	0	0	NA	0	0	0	NA	NA	NA	NA
contig-100_3762	527	N	0	0	NA	0	0	0	NA	NA	NA	NA
contig-100_3763	527	N	0	0	NA	0	0	0	NA	NA	NA	NA
contig-100_3764	527	N	0	0	NA	0	0	0	NA	NA	NA	NA

contig-100_3765	526	N	0	0	NA	0	0	0	NA	Bacteroides thetaiotaomicron VPI-5482, complete genome	gi|29345410|ref|NC_004663.1| Bacteroides thetaiotaomicron VPI-5482 chromosome, complete genome	gi|29345410|ref|NC_004663.1| Bacteroides thetaiotaomicron VPI-5482 chromosome, complete genome
contig-100_3766	526	N	0	0	NA	0	0	0	NA	Uncultured organism clone VC1BV77TF genomic sequence	gi|150002608|ref|NC_009614.1| Bacteroides vulgatus ATCC 8482 chromosome, complete genome	gi|150002608|ref|NC_009614.1| Bacteroides vulgatus ATCC 8482 chromosome, complete genome
contig-100_3767	526	N	0	0	NA	0	0	0	NA	NA	NA	NA
contig-100_3768	526	N	0	0	NA	0	0	0	NA	NA	NA	NA
contig-100_377	2347	N	4	0	NA	0	0	1	NA	Faecalibacterium prausnitzii SL3/3 draft genome	gi|479170689|ref|NC_021020.1| Faecalibacterium prausnitzii SL3/3 draft genome	gi|479170689|ref|NC_021020.1| Faecalibacterium prausnitzii SL3/3 draft genome
contig-100_3770	526	N	0	0	NA	0	0	0	NA	NA	NA	NA
contig-100_3771	526	N	0	0	NA	0	0	0	NA	Faecalibacterium prausnitzii SL3/3 draft genome	gi|479170689|ref|NC_021020.1| Faecalibacterium prausnitzii SL3/3 draft genome	gi|479170689|ref|NC_021020.1| Faecalibacterium prausnitzii SL3/3 draft genome
contig-100_3772	525	N	0	0	NA	0	0	0	NA	NA	NA	NA
contig-100_3773	525	N	0	0	NA	0	0	0	NA	NA	NA	NA
contig-100_3774	525	N	0	0	NA	0	0	0	NA	NA	NA	NA
contig-100_3775	524	N	1	0	NA	0	0	0	NA	Uncultured organism clone VC1BY71TR genomic sequence	gi|479170689|ref|NC_021020.1| Faecalibacterium prausnitzii SL3/3 draft genome	gi|479170689|ref|NC_021020.1| Faecalibacterium prausnitzii SL3/3 draft genome
contig-100_3777	524	N	1	0	NA	0	0	0	NA	NA	NA	NA

contig-100_3778	524	N	0	0	NA	0	0	0	NA	TPA_exp: Clostridium difficile strain QCD-66C26 transposon Tn6110, complete sequence	gi|260685375|ref|NC_013316.1| Clostridium difficile R20291 chromosome, complete genome	gi|260685375|ref|NC_013316.1| Clostridium difficile R20291 chromosome, complete genome
contig-100_3779	524	N	0	0	NA	0	0	0	NA	NA	NA	NA
contig-100_378	2342	N	1	1	Sipho	1	0	1	NA	NA	NA	NA
contig-100_3780	524	N	0	0	NA	0	0	0	NA	NA	NA	NA
contig-100_3782	524	N	0	0	NA	0	0	0	NA	NA	NA	NA
contig-100_3783	524	N	0	0	NA	0	0	0	NA	Bacteroides vulgatus ATCC 8482, complete genome	gi|150002608|ref|NC_009614.1| Bacteroides vulgatus ATCC 8482 chromosome, complete genome	gi|150002608|ref|NC_009614.1| Bacteroides vulgatus ATCC 8482 chromosome, complete genome
contig-100_3784	524	N	0	0	NA	0	0	0	NA	NA	NA	NA
contig-100_3785	524	N	0	0	NA	0	0	0	NA	Bacteroides vulgatus ATCC 8482, complete genome	gi|150002608|ref|NC_009614.1| Bacteroides vulgatus ATCC 8482 chromosome, complete genome	gi|150002608|ref|NC_009614.1| Bacteroides vulgatus ATCC 8482 chromosome, complete genome
contig-100_3786	523	N	1	0	NA	0	0	0	NA	NA	NA	NA
contig-100_3787	523	N	0	0	NA	0	0	0	NA	NA	NA	NA
contig-100_3788	523	N	0	0	NA	0	0	0	NA	NA	NA	NA
contig-100_3789	523	N	0	0	NA	0	0	0	NA	Uncultured organism clone VC1CH11TR genomic sequence	NA	Uncultured organism clone VC1CH11TR genomic sequence
contig-100_3790	523	N	0	0	NA	0	0	0	NA	NA	NA	NA
contig-100_3791	523	N	0	0	NA	0	0	0	NA	NA	NA	NA

contig-100_3792	522	N	1	0	NA	0	0	0	NA	Eubacterium siraeum V10Sc8a draft genome	gi|479150083|ref|NC_021013.1| Ruminococcus bromii L2-63 draft genome	gi|479150083|ref|NC_021013.1| Ruminococcus bromii L2-63 draft genome
contig-100_3793	522	N	0	0	NA	0	0	0	NA	NA	NA	NA
contig-100_3795	522	N	0	0	NA	0	0	0	NA	NA	NA	NA
contig-100_3796	522	N	0	0	NA	0	0	0	NA	NA	NA	NA
contig-100_3797	522	N	0	0	NA	0	0	0	NA	NA	NA	NA
contig-100_3798	522	N	0	0	NA	0	0	0	NA	Coprococcus catus GD/7 draft genome	gi|479136967|ref|NC_021009.1| Coprococcus catus GD/7 draft genome	gi|479136967|ref|NC_021009.1| Coprococcus catus GD/7 draft genome
contig-100_3799	522	N	0	0	NA	0	0	0	NA	NA	NA	NA
contig-100_380	2333	N	2	0	NA	0	1	0	NA	Faecalibacterium prausnitzii L2/6 draft genome	gi|479208076|ref|NC_021042.1| Faecalibacterium prausnitzii L2-6, complete genome	gi|479208076|ref|NC_021042.1| Faecalibacterium prausnitzii L2-6, complete genome
contig-100_3800	522	N	0	0	NA	0	0	0	NA	Bacteroides thetaiotaomicron VPI-5482, complete genome	gi|29345410|ref|NC_004663.1| Bacteroides thetaiotaomicron VPI-5482 chromosome, complete genome	gi|29345410|ref|NC_004663.1| Bacteroides thetaiotaomicron VPI-5482 chromosome, complete genome
contig-100_3801	522	N	1	0	NA	0	0	0	NA	NA	NA	NA
contig-100_3802	522	N	0	0	NA	0	0	0	NA	Eubacterium eligens ATCC 27750 plasmid, complete sequence	gi|238921767|ref|NC_012780.1| Eubacterium eligens ATCC 27750 plasmid unnamed, complete sequence	gi|238921767|ref|NC_012780.1| Eubacterium eligens ATCC 27750 plasmid unnamed, complete sequence
contig-100_3803	521	N	0	0	NA	0	0	0	NA	NA	NA	NA

contig-100_3804	521	N	0	0	NA	0	0	0	NA	Uncultured organism clone VC1AK41TR genomic sequence	NA	Uncultured organism clone VC1AK41TR genomic sequence
contig-100_3805	521	N	0	0	NA	0	0	0	NA	NA	NA	NA
contig-100_3806	521	N	0	0	NA	0	0	0	NA	NA	NA	NA
contig-100_3807	521	N	0	0	NA	0	0	0	NA	NA	NA	NA
contig-100_3808	521	N	0	0	NA	0	0	0	NA	Odoribacter splanchnicus DSM 20712, complete genome	gi|325278757|ref|NC_015160.1| Odoribacter splanchnicus DSM 20712 chromosome, complete genome	gi|325278757|ref|NC_015160.1| Odoribacter splanchnicus DSM 20712 chromosome, complete genome
contig-100_381	2332	N	1	1	Sipho	0	0	0	NA	Clostridiales sp. SSC/2 draft genome	gi|479158859|ref|NC_021016.1| Butyrate-producing bacterium SSC/2, complete genome	gi|479158859|ref|NC_021016.1| Butyrate-producing bacterium SSC/2, complete genome
contig-100_3811	521	N	0	0	NA	0	0	0	NA	NA	NA	NA
contig-100_3812	521	N	0	0	NA	0	0	0	NA	NA	NA	NA
contig-100_3813	520	N	0	0	NA	0	0	0	NA	NA	NA	NA
contig-100_3814	520	N	0	0	NA	0	0	0	NA	NA	NA	NA
contig-100_3817	520	N	0	0	NA	0	0	0	NA	NA	NA	NA
contig-100_3818	520	N	0	0	NA	0	0	0	NA	Aggregatibacter aphrophilus NJ8700, complete genome	gi|251791816|ref|NC_012913.1| Aggregatibacter aphrophilus NJ8700 chromosome, complete genome	gi|251791816|ref|NC_012913.1| Aggregatibacter aphrophilus NJ8700 chromosome, complete genome
contig-100_3819	520	N	0	0	NA	0	0	0	NA	NA	NA	NA
contig-100_3820	520	N	1	0	NA	0	0	0	NA	NA	NA	NA
contig-100_3823	519	N	0	0	NA	0	0	0	NA	NA	NA	NA
contig-100_3824	519	N	0	0	NA	0	0	0	NA	NA	NA	NA
contig-100_3825	519	N	0	0	NA	0	0	0	NA	NA	NA	NA
contig-100_3826	519	N	1	0	NA	0	0	0	NA	NA	NA	NA

contig-100_3827	519	N	0	0	NA	0	0	0	NA	Uncultured organism clone VC1CJ10TR genomic sequence	gi|150002608|ref|NC_009614.1| Bacteroides vulgatus ATCC 8482 chromosome, complete genome	gi|150002608|ref|NC_009614.1| Bacteroides vulgatus ATCC 8482 chromosome, complete genome
contig-100_3828	518	N	1	0	NA	0	0	0	NA	Uncultured organism clone VC1DA45TF genomic sequence	gi|53711291|ref|NC_006347.1| Bacteroides fragilis YCH46 DNA, complete genome	gi|53711291|ref|NC_006347.1| Bacteroides fragilis YCH46 DNA, complete genome
contig-100_383	2321	N	1	0	NA	0	0	0	NA	NA	NA	NA
contig-100_3830	518	N	0	0	NA	0	0	0	NA	NA	NA	NA
contig-100_3833	517	N	1	0	NA	0	0	0	NA	NA	NA	NA
contig-100_3834	517	N	0	0	NA	0	0	0	NA	Propionibacterium acnes HL096PA1, complete genome	gi|365972921|ref|NC_016516.1| Propionibacterium acnes TypeIA2 P.acn33 chromosome, complete genome	gi|365972921|ref|NC_016516.1| Propionibacterium acnes TypeIA2 P.acn33 chromosome, complete genome
contig-100_3835	517	N	0	0	NA	0	0	0	NA	Ruminococcus sp. SR1/5 draft genome	gi|479152295|ref|NC_021014.1| Ruminococcus sp. SR1/5 draft genome	gi|479152295|ref|NC_021014.1| Ruminococcus sp. SR1/5 draft genome
contig-100_3836	517	N	0	0	NA	0	0	0	NA	NA	NA	NA
contig-100_3837	516	N	0	0	NA	0	0	0	NA	NA	NA	NA
contig-100_3838	516	N	0	0	NA	0	0	0	NA	NA	NA	NA
contig-100_3839	516	N	0	0	NA	0	0	0	NA	Ruminococcus champanellensis type strain 18P13T draft genome	gi|479199283|ref|NC_021039.1| Ruminococcus champanellensis 18P13, complete genome	gi|479199283|ref|NC_021039.1| Ruminococcus champanellensis 18P13, complete genome

contig-100_384	2318	N	2	0	NA	0	0	0	NA	Uncultured organism clone 1041059765897 genomic sequence	NA	Uncultured organism clone 104105976589 7 genomic sequence
contig-100_3840	516	N	0	0	NA	0	0	0	NA	NA	NA	NA
contig-100_3841	516	N	0	0	NA	0	0	0	NA	NA	NA	NA
contig-100_3842	516	N	0	0	NA	0	0	0	NA	NA	NA	NA
contig-100_3844	515	N	0	0	NA	0	0	0	NA	Ruminococcus torques L2-14 draft genome	gi|479155735|ref|NC_021015.1| Ruminococcus torques L2-14 draft genome	gi|479155735|ref|NC_021015 .1| Ruminococcus torques L2-14 draft genome
contig-100_3845	515	N	0	0	NA	0	0	0	NA	Eubacterium eligens ATCC 27750 plasmid, complete sequence	gi|238921767|ref|NC_012780.1| Eubacterium eligens ATCC 27750 plasmid unnamed, complete sequence	gi|238921767|ref|NC_012780 .1| Eubacterium eligens ATCC 27750 plasmid unnamed, complete sequence
contig-100_3846	515	N	1	0	NA	0	0	0	NA	NA	NA	NA
contig-100_3847	515	N	0	0	NA	0	0	0	NA	Bacteroides vulgatus ATCC 8482, complete genome	gi|150002608|ref|NC_009614.1| Bacteroides vulgatus ATCC 8482 chromosome, complete genome	gi|150002608|ref|NC_009614 .1| Bacteroides vulgatus ATCC 8482 chromosome, complete genome
contig-100_3848	515	N	0	0	NA	0	0	0	NA	NA	NA	NA
contig-100_3849	515	N	0	0	NA	0	0	0	NA	NA	NA	NA
contig-100_385	2315	N	2	0	NA	0	0	0	NA	Bacteroides vulgatus ATCC 8482, complete genome	gi|150002608|ref|NC_009614.1| Bacteroides vulgatus ATCC 8482 chromosome, complete genome	gi|150002608|ref|NC_009614 .1| Bacteroides vulgatus ATCC 8482 chromosome, complete genome
contig-100_3850	515	N	0	0	NA	0	0	0	NA	NA	NA	NA

contig-100_3851	515	N	0	0	NA	0	0	0	NA	Lactobacillus rhamnosus Lc-Nu-like prophage Orf65 gene, partial cds; Orf139, Orf126, Orf203, Orf116, Orf76, putative tape measure protein, Orf630, putative host-specificity protein, Orf96, Orf43, putative holin, and Orf56 genes, complete cds; and putative holin gene, partial cds	gi|258506995|ref|NC_013198.1| Lactobacillus rhamnosus GG chromosome, complete genome	gi|258506995|ref|NC_013198.1| Lactobacillus rhamnosus GG chromosome, complete genome
contig-100_3852	515	N	1	0	NA	0	0	0	NA	NA	NA	NA
contig-100_3854	514	N	1	0	NA	0	0	0	NA	NA	NA	NA
contig-100_3855	514	N	1	0	NA	0	0	0	NA	Verminephrobacter eiseniae EF01-2, complete genome	gi|121607004|ref|NC_008786.1| Verminephrobacter eiseniae EF01-2 chromosome, complete genome	gi|121607004|ref|NC_008786.1| Verminephrobacter eiseniae EF01-2 chromosome, complete genome
contig-100_3856	514	N	0	0	NA	0	0	0	NA	NA	NA	NA
contig-100_3857	514	N	0	0	NA	0	0	0	NA	NA	NA	NA
contig-100_3858	514	N	0	0	NA	0	0	0	NA	NA	NA	NA
contig-100_3859	514	N	2	0	NA	0	0	0	NA	Bacteroides vulgatus ATCC 8482, complete genome	gi|150002608|ref|NC_009614.1| Bacteroides vulgatus ATCC 8482 chromosome, complete genome	gi|150002608|ref|NC_009614.1| Bacteroides vulgatus ATCC 8482 chromosome, complete genome
contig-100_3860	514	N	0	0	NA	0	0	0	NA	NA	NA	NA
contig-100_3861	514	N	0	0	NA	0	0	0	NA	Bacteroides xylanisolvens XB1A draft genome	gi|479162165|ref|NC_021017.1| Bacteroides xylanisolvens XB1A draft genome	gi|479162165|ref|NC_021017.1| Bacteroides xylanisolvens XB1A draft genome
contig-100_3862	514	N	0	0	NA	0	0	0	NA	NA	NA	NA
contig-100_3865	514	N	1	0	NA	0	0	0	NA	NA	NA	NA

contig-100_3867	514	N	1	0	NA	0	0	0	NA	Faecalibacterium prausnitzii SL3/3 draft genome	gi|479170689|ref|NC_021020.1| Faecalibacterium prausnitzii SL3/3 draft genome	gi|479170689|ref|NC_021020.1| Faecalibacterium prausnitzii SL3/3 draft genome
contig-100_3868	514	N	0	0	NA	0	0	0	NA	Faecalibacterium prausnitzii L2/6 draft genome	gi|479208076|ref|NC_021042.1| Faecalibacterium prausnitzii L2-6, complete genome	gi|479208076|ref|NC_021042.1| Faecalibacterium prausnitzii L2-6, complete genome
contig-100_3869	513	N	0	0	NA	0	0	0	NA	NA	NA	NA
contig-100_3870	513	N	0	0	NA	0	0	0	NA	NA	NA	NA
contig-100_3871	513	N	0	0	NA	0	0	0	NA	Alcanivorax dieselolei B5, complete genome	gi|407693844|ref|NC_018691.1| Alcanivorax dieselolei B5 chromosome, complete genome	gi|407693844|ref|NC_018691.1| Alcanivorax dieselolei B5 chromosome, complete genome
contig-100_3872	513	N	0	0	NA	0	0	0	NA	NA	NA	NA
contig-100_3873	513	N	0	0	NA	0	0	0	NA	NA	NA	NA
contig-100_3874	513	N	0	0	NA	0	0	0	NA	NA	NA	NA
contig-100_3875	513	N	0	0	NA	0	0	0	NA	Eubacterium eligens ATCC 27750, complete genome	gi|238915976|ref|NC_012778.1| Eubacterium eligens ATCC 27750 chromosome, complete genome	gi|238915976|ref|NC_012778.1| Eubacterium eligens ATCC 27750 chromosome, complete genome
contig-100_3876	513	N	0	0	NA	0	0	0	NA	NA	NA	NA
contig-100_3877	512	N	0	0	NA	0	0	0	NA	Bacteroides vulgatus ATCC 8482, complete genome	gi|150002608|ref|NC_009614.1| Bacteroides vulgatus ATCC 8482 chromosome, complete genome	gi|150002608|ref|NC_009614.1| Bacteroides vulgatus ATCC 8482 chromosome, complete genome
contig-100_3878	512	N	0	0	NA	0	0	0	NA	NA	NA	NA
contig-100_3879	512	N	0	0	NA	0	0	0	NA	NA	NA	NA
contig-100_3880	512	N	0	0	NA	0	0	0	NA	NA	NA	NA

contig-100_3881	512	N	0	0	NA	0	0	0	NA	NA	NA	NA
contig-100_3882	512	N	0	0	NA	0	0	0	NA	NA	NA	NA
contig-100_3883	511	N	0	0	NA	0	0	0	NA	NA	NA	NA
contig-100_3884	511	N	1	0	NA	0	0	0	NA	NA	NA	NA
contig-100_3885	511	N	0	0	NA	0	0	0	NA	NA	NA	NA
contig-100_3886	511	N	2	0	NA	0	0	0	NA	NA	NA	NA
contig-100_3887	511	N	0	0	NA	0	0	0	NA	NA	NA	NA
contig-100_3888	511	N	0	0	NA	0	0	0	NA	NA	NA	NA
contig-100_3889	511	N	0	0	NA	0	0	0	NA	Faecalibacterium prausnitzii L2/6 draft genome	gi|479208076|ref|NC_021042.1| Faecalibacterium prausnitzii L2-6, complete genome	gi|479208076|ref|NC_021042.1| Faecalibacterium prausnitzii L2-6, complete genome
contig-100_389	2301	N	1	0	NA	0	0	0	NA	NA	NA	NA
contig-100_3890	510	N	0	0	NA	0	0	0	NA	NA	NA	NA
contig-100_3891	510	N	0	0	NA	0	0	0	NA	NA	NA	NA
contig-100_3893	510	N	1	0	NA	0	0	0	NA	NA	NA	NA
contig-100_3894	510	N	0	0	NA	0	0	0	NA	Ruminococcus champanellensis type strain 18P13T draft genome	gi|479199283|ref|NC_021039.1| Ruminococcus champanellensis 18P13, complete genome	gi|479199283|ref|NC_021039.1| Ruminococcus champanellensis 18P13, complete genome
contig-100_3895	510	N	0	0	NA	0	0	0	NA	Bacteroides vulgatus ATCC 8482, complete genome	gi|150002608|ref|NC_009614.1| Bacteroides vulgatus ATCC 8482 chromosome, complete genome	gi|150002608|ref|NC_009614.1| Bacteroides vulgatus ATCC 8482 chromosome, complete genome
contig-100_3896	510	N	1	0	NA	0	0	0	NA	NA	NA	NA
contig-100_3897	510	N	1	0	NA	0	0	0	NA	NA	NA	NA
contig-100_3899	509	N	1	0	NA	0	0	0	NA	NA	NA	NA
contig-100_39	9291	N	8	0	NA	2	0	1	NA	Uncultured organism clone VC1BS83TR genomic sequence	NA	Uncultured organism clone VC1BS83TR genomic sequence

contig-100_390	2294	N	2	0	NA	0	0	0	NA	Odoribacter splanchnicus DSM 20712, complete genome	gi|325278757|ref|NC_015160.1| Odoribacter splanchnicus DSM 20712 chromosome, complete genome	gi|325278757|ref|NC_015160.1| Odoribacter splanchnicus DSM 20712 chromosome, complete genome
contig-100_3900	509	N	1	1	Sipho	0	0	0	NA	NA	NA	NA
contig-100_3901	509	N	1	0	NA	0	0	0	NA	Bacteroides vulgatus ATCC 8482, complete genome	gi|150002608|ref|NC_009614.1| Bacteroides vulgatus ATCC 8482 chromosome, complete genome	gi|150002608|ref|NC_009614.1| Bacteroides vulgatus ATCC 8482 chromosome, complete genome
contig-100_3902	509	N	0	0	NA	0	0	0	NA	NA	NA	NA
contig-100_3903	508	N	1	0	NA	0	0	0	NA	NA	NA	NA
contig-100_3904	508	N	0	0	NA	0	0	0	NA	NA	NA	NA
contig-100_3905	508	N	0	0	NA	0	0	0	NA	Uncultured organism clone 1041059766474 genomic sequence	NA	Uncultured organism clone 1041059766474 genomic sequence
contig-100_3906	508	N	0	0	NA	0	0	0	NA	Clostridium botulinum F str. 230613, complete genome	gi|153937894|ref|NC_009699.1| Clostridium botulinum F str. Langeland chromosome, complete genome	gi|153937894|ref|NC_009699.1| Clostridium botulinum F str. Langeland chromosome, complete genome
contig-100_3907	507	N	0	0	NA	0	0	0	NA	Faecalibacterium prausnitzii SL3/3 draft genome	gi|479170689|ref|NC_021020.1| Faecalibacterium prausnitzii SL3/3 draft genome	gi|479170689|ref|NC_021020.1| Faecalibacterium prausnitzii SL3/3 draft genome

contig-100_3910	507	N	0	0	NA	0	0	0	NA	Faecalibacterium prausnitzii L2/6 draft genome	gi|479208076|ref|NC_021042.1| Faecalibacterium prausnitzii L2-6, complete genome	gi|479208076|ref|NC_021042.1| Faecalibacterium prausnitzii L2-6, complete genome
contig-100_3911	507	N	1	0	NA	0	0	0	NA	NA	NA	NA
contig-100_3912	507	N	0	0	NA	0	0	0	NA	NA	NA	NA
contig-100_3913	507	N	0	0	NA	0	0	0	NA	NA	NA	NA
contig-100_3914	507	N	0	0	NA	0	0	0	NA	NA	NA	NA
contig-100_3915	507	N	0	0	NA	0	0	0	NA	NA	NA	NA
contig-100_3916	507	N	0	0	NA	0	0	0	NA	NA	NA	NA
contig-100_3917	507	N	0	0	NA	0	0	0	NA	NA	NA	NA
contig-100_3918	506	N	0	0	NA	0	0	0	NA	NA	NA	NA
contig-100_3919	506	N	0	0	NA	0	0	0	NA	Eubacterium rectale M104/1 draft genome	gi|479213596|ref|NC_021044.1| Eubacterium rectale M104/1 draft genome	gi|479213596|ref|NC_021044.1| Eubacterium rectale M104/1 draft genome
contig-100_392	2276	N	2	0	NA	0	0	0	NA	NA	NA	NA
contig-100_3920	506	N	0	0	NA	0	0	0	NA	NA	NA	NA
contig-100_3921	506	N	0	0	NA	0	0	0	NA	NA	NA	NA
contig-100_3923	506	N	0	0	NA	0	0	0	NA	Haemophilus parainfluenzae T3T1 complete genome	gi|345428590|ref|NC_015964.1| Haemophilus parainfluenzae T3T1, complete genome	gi|345428590|ref|NC_015964.1| Haemophilus parainfluenzae T3T1, complete genome
contig-100_3924	506	N	1	0	NA	0	0	0	NA	Klebsiella pneumoniae plasmid pLVPK, complete sequence	gi|57639934|ref|NC_006625.1| Klebsiella pneumoniae NTUH-K2044 plasmid pK2044, complete sequence	gi|57639934|ref|NC_006625.1| Klebsiella pneumoniae NTUH-K2044 plasmid pK2044, complete sequence

contig-100_3925	505	N	0	0	NA	0	0	0	NA	Uncultured bacterium clone LM0ABA38ZA05FM1 genomic sequence	NA	Uncultured bacterium clone LM0ABA38ZA05FM1 genomic sequence
contig-100_3926	505	N	0	0	NA	0	0	0	NA	NA	NA	NA
contig-100_3927	505	N	0	0	NA	0	0	0	NA	NA	NA	NA
contig-100_3928	505	N	1	0	NA	0	0	0	NA	NA	NA	NA
contig-100_3929	504	N	1	0	NA	0	0	0	NA	NA	NA	NA
contig-100_3930	504	N	0	0	NA	0	0	0	NA	NA	NA	NA
contig-100_3931	504	N	0	0	NA	0	0	0	NA	NA	NA	NA
contig-100_3932	504	N	0	0	NA	0	0	0	NA	NA	NA	NA
contig-100_3933	504	N	0	0	NA	0	0	0	NA	Uncultured organism clone 1041059767233 genomic sequence	NA	Uncultured organism clone 1041059767233 genomic sequence
contig-100_3934	504	N	0	0	NA	0	0	0	NA	NA	NA	NA
contig-100_3935	504	N	0	0	NA	0	0	0	NA	Eubacterium cylindroides T2-87 draft genome	gi|479201824|ref|NC_021040.1| Roseburia intestinalis M50/1 draft genome	gi|479201824|ref|NC_021040.1| Roseburia intestinalis M50/1 draft genome
contig-100_3936	504	N	0	0	NA	0	0	0	NA	NA	NA	NA
contig-100_3937	504	N	0	0	NA	0	0	0	NA	Bacteroides xylanisolvens XB1A draft genome	gi|479162165|ref|NC_021017.1| Bacteroides xylanisolvens XB1A draft genome	gi|479162165|ref|NC_021017.1| Bacteroides xylanisolvens XB1A draft genome
contig-100_3938	504	N	1	0	NA	0	0	0	NA	Bacteroides vulgatus ATCC 8482, complete genome	gi|150002608|ref|NC_009614.1| Bacteroides vulgatus ATCC 8482 chromosome, complete genome	gi|150002608|ref|NC_009614.1| Bacteroides vulgatus ATCC 8482 chromosome, complete genome
contig-100_3939	503	N	0	0	NA	0	0	0	NA	NA	NA	NA

contig-100_394	2266	N	1	0	NA	0	0	0	NA	Alistipes shahii WAL 8301 draft genome	gi|479185170|ref|NC_021030.1| Alistipes shahii WAL 8301 draft genome	gi|479185170|ref|NC_021030.1| Alistipes shahii WAL 8301 draft genome
contig-100_3940	503	N	0	0	NA	0	0	0	NA	NA	NA	NA
contig-100_3941	503	N	1	0	NA	0	0	0	NA	NA	NA	NA
contig-100_3942	503	N	1	0	NA	0	0	0	NA	NA	NA	NA
contig-100_3943	503	N	0	0	NA	0	0	0	NA	NA	NA	NA
contig-100_3944	503	N	0	0	NA	0	0	0	NA	NA	NA	NA
contig-100_3945	503	N	0	0	NA	0	0	0	NA	Bacteroides vulgatus ATCC 8482, complete genome	gi|150002608|ref|NC_009614.1| Bacteroides vulgatus ATCC 8482 chromosome, complete genome	gi|150002608|ref|NC_009614.1| Bacteroides vulgatus ATCC 8482 chromosome, complete genome
contig-100_3946	503	N	0	0	NA	0	0	0	NA	NA	NA	NA
contig-100_3947	503	N	0	0	NA	0	0	0	NA	NA	NA	NA
contig-100_3948	503	N	0	0	NA	0	0	0	NA	NA	NA	NA
contig-100_395	2263	N	1	0	NA	0	0	0	NA	Bacteroides salanitronis DSM 18170, complete genome	gi|325297172|ref|NC_015164.1| Bacteroides salanitronis DSM 18170 chromosome, complete genome	gi|325297172|ref|NC_015164.1| Bacteroides salanitronis DSM 18170 chromosome, complete genome
contig-100_3950	502	N	0	0	NA	0	0	0	NA	NA	NA	NA
contig-100_3951	502	N	2	0	NA	0	0	0	NA	Acidovorax sp. KKS102, complete genome	gi|407936729|ref|NC_018708.1| Acidovorax sp. KKS102 chromosome, complete genome	gi|407936729|ref|NC_018708.1| Acidovorax sp. KKS102 chromosome, complete genome
contig-100_3952	502	N	0	0	NA	0	0	0	NA	Clostridiales sp. SSC/2 draft genome	gi|479158859|ref|NC_021016.1| Butyrate-producing bacterium SSC/2, complete genome	gi|479158859|ref|NC_021016.1| Butyrate-producing bacterium SSC/2, complete genome

contig-100_3953	502	N	0	0	NA	0	0	0	NA	Clostridiales sp. SSC/2 draft genome	gi|479158859|ref|NC_021016.1| Butyrate-producing bacterium SSC/2, complete genome	gi|479158859|ref|NC_021016.1| Butyrate-producing bacterium SSC/2, complete genome
contig-100_3954	502	N	0	0	NA	0	0	0	NA	Haemophilus parainfluenzae T3T1 complete genome	gi|345428590|ref|NC_015964.1| Haemophilus parainfluenzae T3T1, complete genome	gi|345428590|ref|NC_015964.1| Haemophilus parainfluenzae T3T1, complete genome
contig-100_3955	502	N	0	0	NA	0	0	0	NA	NA	NA	NA
contig-100_3956	502	N	1	0	NA	0	0	0	NA	Faecalibacterium prausnitzii L2/6 draft genome	gi|479208076|ref|NC_021042.1| Faecalibacterium prausnitzii L2-6, complete genome	gi|479208076|ref|NC_021042.1| Faecalibacterium prausnitzii L2-6, complete genome
contig-100_3957	502	N	0	0	NA	0	0	0	NA	Eubacterium eligens ATCC 27750, complete genome	gi|238915976|ref|NC_012778.1| Eubacterium eligens ATCC 27750 chromosome, complete genome	gi|238915976|ref|NC_012778.1| Eubacterium eligens ATCC 27750 chromosome, complete genome
contig-100_3958	502	N	0	0	NA	0	0	0	NA	NA	NA	NA
contig-100_3959	501	N	0	0	NA	0	0	0	NA	NA	NA	NA
contig-100_396	2262	N	2	0	NA	0	0	0	NA	NA	NA	NA
contig-100_3960	501	N	0	0	NA	0	0	0	NA	Faecalibacterium prausnitzii L2/6 draft genome	gi|479208076|ref|NC_021042.1| Faecalibacterium prausnitzii L2-6, complete genome	gi|479208076|ref|NC_021042.1| Faecalibacterium prausnitzii L2-6, complete genome

contig-100_3961	501	N	0	0	NA	0	0	0	NA	Geobacillus thermodenitrificans NG80-2, complete genome	gi|138893679|ref|NC_009328.1| Geobacillus thermodenitrificans NG80-2 chromosome, complete genome	gi|138893679|ref|NC_009328.1| Geobacillus thermodenitrificans NG80-2 chromosome, complete genome
contig-100_3962	501	N	0	0	NA	0	0	0	NA	NA	NA	NA
contig-100_3964	501	N	0	0	NA	0	0	0	NA	NA	NA	NA
contig-100_3965	501	N	0	0	NA	0	0	0	NA	NA	NA	NA
contig-100_3966	501	N	1	0	NA	0	0	0	NA	Uncultured bacterium clone LM0ACA5ZG05RM1 genomic sequence	NA	Uncultured bacterium clone LM0ACA5ZG05RM1 genomic sequence
contig-100_3967	501	N	0	0	NA	0	0	0	NA	Faecalibacterium prausnitzii SL3/3 draft genome	gi|479170689|ref|NC_021020.1| Faecalibacterium prausnitzii SL3/3 draft genome	gi|479170689|ref|NC_021020.1| Faecalibacterium prausnitzii SL3/3 draft genome
contig-100_3969	500	N	0	0	NA	0	0	0	NA	NA	NA	NA
contig-100_3970	500	N	0	0	NA	0	0	0	NA	Bacteroides thetaiotaomicron VPI-5482, complete genome	gi|29345410|ref|NC_004663.1| Bacteroides thetaiotaomicron VPI-5482 chromosome, complete genome	gi|29345410|ref|NC_004663.1| Bacteroides thetaiotaomicron VPI-5482 chromosome, complete genome
contig-100_3971	500	N	0	0	NA	0	0	0	NA	NA	NA	NA
contig-100_3972	500	N	2	0	NA	0	0	0	NA	NA	NA	NA
contig-100_3973	500	N	0	0	NA	0	0	0	NA	Uncultured organism clone VC1CW70TF genomic sequence	gi|479208076|ref|NC_021042.1| Faecalibacterium prausnitzii L2-6, complete genome	gi|479208076|ref|NC_021042.1| Faecalibacterium prausnitzii L2-6, complete genome

contig-100_3974	500	N	0	0	NA	0	0	0	NA	Bacteroides vulgatus ATCC 8482, complete genome	gi|150002608|ref|NC_009614.1| Bacteroides vulgatus ATCC 8482 chromosome, complete genome	gi|150002608|ref|NC_009614.1| Bacteroides vulgatus ATCC 8482 chromosome, complete genome
contig-100_3975	500	N	1	0	NA	0	0	0	NA	NA	NA	NA
contig-100_3976	500	N	0	0	NA	0	0	0	NA	Eubacterium rectale M104/1 draft genome	gi|479213596|ref|NC_021044.1| Eubacterium rectale M104/1 draft genome	gi|479213596|ref|NC_021044.1| Eubacterium rectale M104/1 draft genome
contig-100_3977	500	N	0	0	NA	0	0	0	NA	Bacteroides vulgatus ATCC 8482, complete genome	gi|150002608|ref|NC_009614.1| Bacteroides vulgatus ATCC 8482 chromosome, complete genome	gi|150002608|ref|NC_009614.1| Bacteroides vulgatus ATCC 8482 chromosome, complete genome
contig-100_3978	500	N	0	0	NA	0	0	0	NA	Uncultured organism clone VC1CK46TF genomic sequence	gi|150002608|ref|NC_009614.1| Bacteroides vulgatus ATCC 8482 chromosome, complete genome	gi|150002608|ref|NC_009614.1| Bacteroides vulgatus ATCC 8482 chromosome, complete genome
contig-100_3979	500	N	1	0	NA	0	0	0	NA	NA	NA	NA
contig-100_398	2260	N	2	2	Sipho	1	1	2	NA	Propionibacterium acnes HL096PA1, complete genome	gi|50841496|ref|NC_006085.1| Propionibacterium acnes KPA171202 chromosome, complete genome	gi|50841496|ref|NC_006085.1| Propionibacterium acnes KPA171202 chromosome, complete genome

contig-100_400	2252	N	2	0	NA	0	0	0	NA	Bacteroides xylanisolvens XB1A draft genome	gi|29345410|ref|NC_004663.1| Bacteroides thetaiotaomicron VPI-5482 chromosome, complete genome	gi|29345410|ref|NC_004663.1| Bacteroides thetaiotaomicron VPI-5482 chromosome, complete genome
contig-100_402	2212	N	1	0	NA	0	0	0	NA	NA	NA	NA
contig-100_404	2211	N	2	0	NA	0	0	0	NA	NA	NA	NA
contig-100_405	2211	N	1	0	NA	0	0	0	NA	Odoribacter splanchnicus DSM 20712, complete genome	gi|325278757|ref|NC_015160.1| Odoribacter splanchnicus DSM 20712 chromosome, complete genome	gi|325278757|ref|NC_015160.1| Odoribacter splanchnicus DSM 20712 chromosome, complete genome
contig-100_406	2210	N	0	0	NA	0	0	0	NA	NA	NA	NA
contig-100_407	2204	N	2	0	NA	0	0	0	NA	NA	NA	NA
contig-100_41	9055	N	2	0	NA	0	0	0	NA	NA	NA	NA
contig-100_411	2192	N	2	0	NA	0	0	0	NA	Faecalibacterium prausnitzii L2/6 draft genome	gi|479208076|ref|NC_021042.1| Faecalibacterium prausnitzii L2-6, complete genome	gi|479208076|ref|NC_021042.1| Faecalibacterium prausnitzii L2-6, complete genome
contig-100_412	2189	N	0	0	NA	0	0	0	NA	Alistipes shahii WAL 8301 draft genome	gi|479185170|ref|NC_021030.1| Alistipes shahii WAL 8301 draft genome	gi|479185170|ref|NC_021030.1| Alistipes shahii WAL 8301 draft genome
contig-100_415	2178	N	0	0	NA	0	0	0	NA	NA	NA	NA
contig-100_416	2173	N	6	0	NA	0	0	0	NA	NA	NA	NA
contig-100_417	2167	N	3	0	NA	0	0	0	NA	Uncultured organism clone VC1AO86TF genomic sequence	NA	Uncultured organism clone VC1AO86TF genomic sequence
contig-100_420	2159	N	1	0	NA	0	0	0	NA	NA	NA	NA

contig-100_421	2157	N	1	0	NA	0	0	0	NA	Unidentified phage clone 2019_scaffold132 genomic sequence	NA	Unidentified phage clone 2019_scaffold 132 genomic sequence
contig-100_422	2151	N	2	0	NA	0	0	0	NA	Shuttle vector pTBR101-CM DNA, complete sequence	gi|384200575|ref|NC_017221.1| Bifidobacterium longum subsp. longum KACC 91563 chromosome, complete genome	gi|384200575|ref|NC_017221.1| Bifidobacterium longum subsp. longum KACC 91563 chromosome, complete genome
contig-100_423	2145	N	3	0	NA	0	0	0	NA	Roseburia hominis A2-183, complete genome	gi|347530298|ref|NC_015977.1| Roseburia hominis A2-183 chromosome, complete genome	gi|347530298|ref|NC_015977.1| Roseburia hominis A2-183 chromosome, complete genome
contig-100_424	2142	N	1	0	NA	0	0	0	NA	Clostridiales sp. SM4/1 draft genome	gi|479181986|ref|NC_021024.1| Butyrate-producing bacterium SM4/1, complete genome	gi|479181986|ref|NC_021024.1| Butyrate-producing bacterium SM4/1, complete genome
contig-100_425	2142	N	2	0	NA	0	0	0	NA	NA	NA	NA
contig-100_426	2137	N	0	0	NA	0	0	0	NA	NA	NA	NA
contig-100_427	2136	N	4	1	Sipho	0	0	1	NA	Bradyrhizobium japonicum USDA 6 DNA, complete genome	gi|384213726|ref|NC_017249.1| Bradyrhizobium japonicum USDA 6, complete genome	gi|384213726|ref|NC_017249.1| Bradyrhizobium japonicum USDA 6, complete genome
contig-100_428	2135	N	1	0	NA	0	0	0	NA	NA	NA	NA
contig-100_429	2132	N	1	0	NA	0	0	0	NA	NA	NA	NA
contig-100_43	8956	N	6	1	Adeno	0	1	1	NA	Uncultured organism clone 1041059767364 genomic sequence	NA	Uncultured organism clone 1041059767364 genomic sequence

contig-100_430	2123	N	1	0	NA	0	0	0	NA	Candidatus Saccharimonas aalborgensis, complete genome	gi|501435806|ref|NC_021219.1| Candidatus Saccharimonas aalborgensis, complete genome	gi|501435806|ref|NC_021219.1| Candidatus Saccharimonas aalborgensis, complete genome
contig-100_431	2121	N	2	0	NA	0	0	0	NA	Faecalibacterium prausnitzii L2/6 draft genome	gi|479208076|ref|NC_021042.1| Faecalibacterium prausnitzii L2-6, complete genome	gi|479208076|ref|NC_021042.1| Faecalibacterium prausnitzii L2-6, complete genome
contig-100_433	2113	N	2	0	NA	0	0	0	NA	Bacteroides thetaiotaomicron VPI-5482, complete genome	gi|29345410|ref|NC_004663.1| Bacteroides thetaiotaomicron VPI-5482 chromosome, complete genome	gi|29345410|ref|NC_004663.1| Bacteroides thetaiotaomicron VPI-5482 chromosome, complete genome
contig-100_434	2109	N	2	0	NA	0	0	0	NA	NA	NA	NA
contig-100_435	2109	N	1	0	NA	0	0	0	NA	Unidentified phage clone 2019_scaffold132 genomic sequence	NA	Unidentified phage clone 2019_scaffold132 genomic sequence
contig-100_438	2096	N	1	0	NA	0	0	0	NA	Clostridiales sp. SM4/1 draft genome	gi|479181986|ref|NC_021024.1| Butyrate-producing bacterium SM4/1, complete genome	gi|479181986|ref|NC_021024.1| Butyrate-producing bacterium SM4/1, complete genome
contig-100_439	2095	N	1	0	NA	0	0	0	NA	Bacteroides vulgatus ATCC 8482, complete genome	gi|150002608|ref|NC_009614.1| Bacteroides vulgatus ATCC 8482 chromosome, complete genome	gi|150002608|ref|NC_009614.1| Bacteroides vulgatus ATCC 8482 chromosome, complete genome
contig-100_441	2079	N	1	0	NA	0	0	0	NA	NA	NA	NA
contig-100_442	2079	N	1	0	NA	0	0	0	NA	NA	NA	NA

contig-100_444	2078	N	2	0	NA	0	0	0	NA	Haemophilus parainfluenzae T3T1 complete genome	gi|345428590|ref|NC_015964.1| Haemophilus parainfluenzae T3T1, complete genome	gi|345428590|ref|NC_015964.1| Haemophilus parainfluenzae T3T1, complete genome
contig-100_445	2076	N	3	0	NA	0	0	0	NA	Alicycliphilus denitrificans K601, complete genome	gi|330822653|ref|NC_015422.1| Alicycliphilus denitrificans K601 chromosome, complete genome	gi|330822653|ref|NC_015422.1| Alicycliphilus denitrificans K601 chromosome, complete genome
contig-100_446	2075	N	2	0	NA	0	0	0	NA	Streptococcus suis D12, complete genome	gi|386585202|ref|NC_017621.1| Streptococcus suis D12 chromosome, complete genome	gi|386585202|ref|NC_017621.1| Streptococcus suis D12 chromosome, complete genome
contig-100_447	2072	N	1	0	NA	0	0	0	NA	Faecalibacterium prausnitzii SL3/3 draft genome	gi|479170689|ref|NC_021020.1| Faecalibacterium prausnitzii SL3/3 draft genome	gi|479170689|ref|NC_021020.1| Faecalibacterium prausnitzii SL3/3 draft genome
contig-100_448	2065	N	1	1	Myo	0	0	1	NA	NA	NA	NA
contig-100_449	2059	N	4	0	NA	0	0	0	NA	Eubacterium rectale DSM 17629 draft genome	gi|479140210|ref|NC_021010.1| Eubacterium rectale DSM 17629 draft genome	gi|479140210|ref|NC_021010.1| Eubacterium rectale DSM 17629 draft genome
contig-100_45	8759	N	11	1	Sipho	0	0	1	NA	NA	NA	NA
contig-100_450	2059	N	4	0	NA	0	0	1	NA	Faecalibacterium prausnitzii SL3/3 draft genome	gi|479170689|ref|NC_021020.1| Faecalibacterium prausnitzii SL3/3 draft genome	gi|479170689|ref|NC_021020.1| Faecalibacterium prausnitzii SL3/3 draft genome

contig-100_451	2058	N	1	0	NA	0	0	0	NA	Unidentified phage clone 2204_scaffold812 genomic sequence	NA	Unidentified phage clone 2204_scaffold 812 genomic sequence
contig-100_454	2054	N	0	0	NA	0	0	0	NA	NA	NA	NA
contig-100_455	2054	N	0	0	NA	0	0	0	NA	NA	NA	NA
contig-100_456	2052	N	2	0	NA	0	1	1	NA	Faecalibacterium prausnitzii SL3/3 draft genome	gi|479170689|ref|NC_021020.1| Faecalibacterium prausnitzii SL3/3 draft genome	gi|479170689|ref|NC_021020 .1| Faecalibacterium prausnitzii SL3/3 draft genome
contig-100_457	2051	N	2	1	Myo	0	0	1	NA	NA	NA	NA
contig-100_458	2050	N	1	0	NA	0	0	0	NA	Bacteroides fragilis 638R genome	gi|60679597|ref|NC_003228.3| Bacteroides fragilis NCTC 9343, complete genome	gi|60679597|ref|NC_003228. 3| Bacteroides fragilis NCTC 9343, complete genome
contig-100_459	2044	N	2	2	Podo	0	1	0	NA	Faecalibacterium prausnitzii L2/6 draft genome	gi|479208076|ref|NC_021042.1| Faecalibacterium prausnitzii L2-6, complete genome	gi|479208076|ref|NC_021042 .1| Faecalibacterium prausnitzii L2-6, complete genome
contig-100_46	8671	N	10	3	Sipho	1	0	4	NA	Unidentified phage clone 2019_scaffold132 genomic sequence	NA	Unidentified phage clone 2019_scaffold 132 genomic sequence
contig-100_460	2033	N	1	0	NA	0	0	0	NA	Salmonella enteritidis plasmid pB, complete sequence	gi|291575210|ref|NC_013954.1| Erwinia pyrifoliae Ep1/96 plasmid pEP2.6, complete sequence	gi|291575210|ref|NC_013954 .1| Erwinia pyrifoliae Ep1/96 plasmid pEP2.6, complete sequence
contig-100_461	2031	N	4	1	Sipho	0	0	1	NA	NA	NA	NA

contig-100_463	2021	N	1	0	NA	0	0	0	NA	Uncultured bacterium clone HA0AAA3ZF12RM1 genomic sequence	gi|479170689|ref|NC_021020.1| Faecalibacterium prausnitzii SL3/3 draft genome	gi|479170689|ref|NC_021020.1| Faecalibacterium prausnitzii SL3/3 draft genome
contig-100_464	2017	N	2	0	NA	0	0	0	NA	NA	NA	NA
contig-100_466	2009	N	1	0	NA	0	0	0	NA	NA	NA	NA
contig-100_467	2008	N	2	0	NA	0	0	0	NA	Roseburia hominis A2-183, complete genome	gi|347530298|ref|NC_015977.1| Roseburia hominis A2-183 chromosome, complete genome	gi|347530298|ref|NC_015977.1| Roseburia hominis A2-183 chromosome, complete genome
contig-100_468	2006	N	2	0	NA	0	0	0	NA	Faecalibacterium prausnitzii L2/6 draft genome	gi|479208076|ref|NC_021042.1| Faecalibacterium prausnitzii L2-6, complete genome	gi|479208076|ref|NC_021042.1| Faecalibacterium prausnitzii L2-6, complete genome
contig-100_469	2003	N	2	1	Sipho	0	0	1	NA	NA	NA	NA
contig-100_47	8662	Y	5	2	Podo	1	1	3	NA	Neisseria gonorrhoeae NCCP11945, complete genome	gi|194097589|ref|NC_011035.1| Neisseria gonorrhoeae NCCP11945 chromosome, complete genome	gi|194097589|ref|NC_011035.1| Neisseria gonorrhoeae NCCP11945 chromosome, complete genome
contig-100_471	2001	N	2	0	NA	0	0	0	NA	NA	NA	NA
contig-100_474	1990	N	2	0	NA	0	0	0	NA	NA	NA	NA
contig-100_475	1990	N	1	1	Sipho	0	0	1	NA	NA	NA	NA
contig-100_476	1983	N	2	0	NA	0	0	0	NA	NA	NA	NA
contig-100_478	1977	N	2	0	NA	0	0	0	NA	NA	NA	NA
contig-100_479	1974	N	3	0	NA	0	0	0	NA	NA	NA	NA
contig-100_48	8596	N	7	4	Sipho	1	0	4	NA	NA	NA	NA

contig-100_480	1973	N	1	0	NA	0	0	0	NA	Bacteroides vulgatus ATCC 8482, complete genome	gi|150002608|ref|NC_009614.1| Bacteroides vulgatus ATCC 8482 chromosome, complete genome	gi|150002608|ref|NC_009614.1| Bacteroides vulgatus ATCC 8482 chromosome, complete genome
contig-100_482	1972	N	1	0	NA	0	0	0	NA	Faecalibacterium prausnitzii SL3/3 draft genome	gi|479170689|ref|NC_021020.1| Faecalibacterium prausnitzii SL3/3 draft genome	gi|479170689|ref|NC_021020.1| Faecalibacterium prausnitzii SL3/3 draft genome
contig-100_484	1966	N	3	1	Sipho	0	0	1	NA	NA	NA	NA
contig-100_485	1956	N	1	0	NA	0	0	0	NA	Uncultured organism clone VC1AO86TF genomic sequence	NA	Uncultured organism clone VC1AO86TF genomic sequence
contig-100_486	1954	N	2	0	NA	0	0	0	NA	Uncultured bacterium clone SJTU_A2_05_87 16S ribosomal RNA gene, partial sequence	gi|150002608|ref|NC_009614.1| Bacteroides vulgatus ATCC 8482 chromosome, complete genome	gi|150002608|ref|NC_009614.1| Bacteroides vulgatus ATCC 8482 chromosome, complete genome
contig-100_487	1954	N	1	0	NA	0	0	0	NA	Faecalibacterium prausnitzii SL3/3 draft genome	gi|479170689|ref|NC_021020.1| Faecalibacterium prausnitzii SL3/3 draft genome	gi|479170689|ref|NC_021020.1| Faecalibacterium prausnitzii SL3/3 draft genome
contig-100_488	1950	N	1	0	NA	0	0	1	NA	NA	NA	NA
contig-100_49	8533	N	11	0	NA	0	0	0	NA	NA	NA	NA
contig-100_490	1943	N	2	1	Sipho	0	1	1	NA	Haemophilus parainfluenzae T3T1 complete genome	gi|345428590|ref|NC_015964.1| Haemophilus parainfluenzae T3T1, complete genome	gi|345428590|ref|NC_015964.1| Haemophilus parainfluenzae T3T1, complete genome

contig-100_491	1942	N	1	0	NA	0	0	0	NA	Haemophilus parainfluenzae T3T1 complete genome	gi|345428590|ref|NC_015964.1| Haemophilus parainfluenzae T3T1, complete genome	gi|345428590|ref|NC_015964.1| Haemophilus parainfluenzae T3T1, complete genome
contig-100_492	1940	N	1	0	NA	0	0	0	NA	Faecalibacterium prausnitzii L2/6 draft genome	gi|479208076|ref|NC_021042.1| Faecalibacterium prausnitzii L2-6, complete genome	gi|479208076|ref|NC_021042.1| Faecalibacterium prausnitzii L2-6, complete genome
contig-100_493	1939	N	1	1	Pox	0	1	1	NA	Haemophilus parainfluenzae T3T1 complete genome	gi|345428590|ref|NC_015964.1| Haemophilus parainfluenzae T3T1, complete genome	gi|345428590|ref|NC_015964.1| Haemophilus parainfluenzae T3T1, complete genome
contig-100_494	1938	N	0	0	NA	0	0	0	NA	NA	NA	NA
contig-100_495	1936	N	0	0	NA	0	0	0	NA	Uncultured organism clone VC1AE44TF genomic sequence	NA	Uncultured organism clone VC1AE44TF genomic sequence
contig-100_496	1934	N	1	0	NA	0	0	0	NA	Ruminococcus torques L2-14 draft genome	gi|479155735|ref|NC_021015.1| Ruminococcus torques L2-14 draft genome	gi|479155735|ref|NC_021015.1| Ruminococcus torques L2-14 draft genome
contig-100_497	1931	N	2	0	NA	0	0	1	NA	NA	NA	NA
contig-100_5	29834	N	16	3	Sipho	0	0	4	NA	Odoribacter splanchnicus DSM 20712, complete genome	gi|325278757|ref|NC_015160.1| Odoribacter splanchnicus DSM 20712 chromosome, complete genome	gi|325278757|ref|NC_015160.1| Odoribacter splanchnicus DSM 20712 chromosome, complete genome
contig-100_500	1921	N	2	0	NA	0	0	0	NA	NA	NA	NA

contig-100_501	1920	N	1	0	NA	0	0	0	NA	Unidentified phage clone 2011_scaffold3 genomic sequence	NA	Unidentified phage clone 2011_scaffold 3 genomic sequence
contig-100_502	1920	N	2	1	Sipho	0	1	1	NA	Uncultured organism clone VC1D713TF genomic sequence	gi|479170689|ref|NC_021020.1| Faecalibacterium prausnitzii SL3/3 draft genome	gi|479170689|ref|NC_021020.1| Faecalibacterium prausnitzii SL3/3 draft genome
contig-100_504	1917	N	2	0	NA	0	0	0	NA	NA	NA	NA
contig-100_505	1917	N	1	0	NA	0	0	0	NA	NA	NA	NA
contig-100_506	1916	N	2	0	NA	0	0	0	NA	Uncultured organism clone VC1BP36TF genomic sequence	NA	Uncultured organism clone VC1BP36TF genomic sequence
contig-100_507	1916	N	1	0	NA	0	0	1	NA	Haemophilus influenzae 86-028NP, complete genome	gi|148826757|ref|NC_009567.1| Haemophilus influenzae PittGG chromosome, complete genome	gi|148826757|ref|NC_009567.1| Haemophilus influenzae PittGG chromosome, complete genome
contig-100_51	8443	N	6	5	Sipho	0	0	5	NA	NA	NA	NA
contig-100_510	1907	N	2	0	NA	0	0	0	NA	NA	NA	NA
contig-100_511	1906	N	2	0	NA	0	0	0	NA	NA	NA	NA
contig-100_513	1899	N	1	0	NA	0	0	0	NA	Bacteroides salanitronis DSM 18170, complete genome	gi|325297172|ref|NC_015164.1| Bacteroides salanitronis DSM 18170 chromosome, complete genome	gi|325297172|ref|NC_015164.1| Bacteroides salanitronis DSM 18170 chromosome, complete genome
contig-100_514	1897	N	1	0	NA	0	0	0	NA	NA	NA	NA
contig-100_515	1897	N	0	0	NA	0	0	0	NA	NA	NA	NA
contig-100_517	1894	N	1	0	NA	0	0	0	NA	Faecalibacterium prausnitzii SL3/3 draft genome	gi|479170689|ref|NC_021020.1| Faecalibacterium prausnitzii SL3/3 draft genome	gi|479170689|ref|NC_021020.1| Faecalibacterium prausnitzii SL3/3 draft genome

contig-100_524	1885	N	2	0	NA	0	0	1	NA	NA	NA	NA
contig-100_526	1875	N	1	1	Sipho	0	0	1	NA	Clostridiales sp. SSC/2 draft genome	gi|479158859|ref|NC_021016.1| Butyrate-producing bacterium SSC/2, complete genome	gi|479158859|ref|NC_021016.1| Butyrate-producing bacterium SSC/2, complete genome
contig-100_527	1874	N	2	0	NA	0	0	0	NA	NA	NA	NA
contig-100_528	1874	N	1	0	NA	0	0	0	NA	NA	NA	NA
contig-100_529	1872	N	1	0	NA	0	0	0	NA	NA	NA	NA
contig-100_531	1865	N	1	0	NA	0	0	0	NA	NA	NA	NA
contig-100_532	1859	N	2	1	Myo	0	1	1	NA	NA	NA	NA
contig-100_533	1857	N	0	0	NA	0	0	0	NA	NA	NA	NA
contig-100_536	1853	N	1	0	NA	0	0	0	NA	NA	NA	NA
contig-100_537	1851	N	2	1	Myo	0	0	1	NA	NA	NA	NA
contig-100_538	1845	N	0	0	NA	0	0	0	NA	Propionibacterium acnes HL096PA1, complete genome	gi|365972921|ref|NC_016516.1| Propionibacterium acnes TypeIA2 P.acn33 chromosome, complete genome	gi|365972921|ref|NC_016516.1| Propionibacterium acnes TypeIA2 P.acn33 chromosome, complete genome
contig-100_539	1845	N	1	0	NA	0	0	0	NA	NA	NA	NA
contig-100_540	1844	N	0	0	NA	0	0	0	NA	Bacteroides vulgatus ATCC 8482, complete genome	gi|150002608|ref|NC_009614.1| Bacteroides vulgatus ATCC 8482 chromosome, complete genome	gi|150002608|ref|NC_009614.1| Bacteroides vulgatus ATCC 8482 chromosome, complete genome
contig-100_541	1842	N	2	0	NA	0	0	0	NA	Uncultured bacterium clone LM0ACA22ZB10FM1 genomic sequence	NA	Uncultured bacterium clone LM0ACA22ZB10FM1 genomic sequence
contig-100_542	1841	N	2	0	NA	0	0	0	NA	NA	NA	NA

contig-100_543	1837	N	0	0	NA	0	0	0	NA	Clostridiales sp. SS3/4 draft genome	gi|479192860|ref|NC_021035.1| Butyrate-producing bacterium SS3/4, complete genome	gi|479192860|ref|NC_021035.1| Butyrate-producing bacterium SS3/4, complete genome
contig-100_544	1832	N	2	0	NA	0	0	0	NA	NA	NA	NA
contig-100_545	1831	N	0	0	NA	0	0	0	NA	NA	NA	NA
contig-100_546	1830	N	1	0	NA	0	0	0	NA	Haemophilus parainfluenzae T3T1 complete genome	gi|345428590|ref|NC_015964.1| Haemophilus parainfluenzae T3T1, complete genome	gi|345428590|ref|NC_015964.1| Haemophilus parainfluenzae T3T1, complete genome
contig-100_547	1829	N	1	0	NA	0	0	0	NA	Staphylococcus aureus strain MSSA476, complete genome	gi|21281729|ref|NC_003923.1| Staphylococcus aureus subsp. aureus MW2, complete genome	gi|21281729|ref|NC_003923.1| Staphylococcus aureus subsp. aureus MW2, complete genome
contig-100_549	1828	N	2	1	Sipho	0	0	2	NA	NA	NA	NA
contig-100_55	7701	N	8	4	Sipho	0	0	4	NA	Unidentified phage clone 2019_scaffold132 genomic sequence	NA	Unidentified phage clone 2019_scaffold132 genomic sequence
contig-100_550	1828	N	1	0	NA	0	0	0	NA	Bacteroides thetaiotaomicron VPI-5482, complete genome	gi|29345410|ref|NC_004663.1| Bacteroides thetaiotaomicron VPI-5482 chromosome, complete genome	gi|29345410|ref|NC_004663.1| Bacteroides thetaiotaomicron VPI-5482 chromosome, complete genome
contig-100_551	1826	N	1	0	NA	0	0	0	NA	NA	NA	NA
contig-100_552	1826	N	1	0	NA	0	0	0	NA	NA	NA	NA
contig-100_555	1823	N	2	0	NA	0	0	0	NA	NA	NA	NA
contig-100_556	1819	N	2	0	NA	0	0	0	NA	NA	NA	NA
contig-100_557	1818	N	0	0	NA	0	0	0	NA	NA	NA	NA

contig-100_558	1818	N	2	0	NA	0	0	0	NA	Haemophilus parainfluenzae T3T1 complete genome	gi|345428590|ref|NC_015964.1| Haemophilus parainfluenzae T3T1, complete genome	gi|345428590|ref|NC_015964.1| Haemophilus parainfluenzae T3T1, complete genome
contig-100_559	1815	N	0	0	NA	0	0	0	NA	NA	NA	NA
contig-100_56	7693	N	5	4	Sipho	2	0	4	NA	Enterobacteria phage lambda, complete genome	gi|238899406|ref|NC_012759.1| Escherichia coli BW2952 chromosome, complete genome	gi|238899406|ref|NC_012759.1| Escherichia coli BW2952 chromosome, complete genome
contig-100_560	1813	N	1	0	NA	0	0	0	NA	Uncultured organism clone 1041059765029 genomic sequence	gi|319899888|ref|NC_014933.1| Bacteroides helcogenes P 36-108 chromosome, complete genome	gi|319899888|ref|NC_014933.1| Bacteroides helcogenes P 36-108 chromosome, complete genome
contig-100_561	1810	N	3	0	NA	0	0	0	NA	NA	NA	NA
contig-100_563	1809	N	2	0	NA	0	0	0	NA	Ruminococcus obeum A2-162 draft genome	gi|479176048|ref|NC_021022.1| Ruminococcus obeum A2-162 draft genome	gi|479176048|ref|NC_021022.1| Ruminococcus obeum A2-162 draft genome
contig-100_566	1804	N	0	0	NA	0	0	0	NA	Uncultured bacterium clone LM0ABA6ZH03RM1 genomic sequence	gi|479176048|ref|NC_021022.1| Ruminococcus obeum A2-162 draft genome	gi|479176048|ref|NC_021022.1| Ruminococcus obeum A2-162 draft genome
contig-100_567	1803	N	1	0	NA	0	0	0	NA	Parabacteroides distasonis ATCC 8503, complete genome	gi|150006674|ref|NC_009615.1| Parabacteroides distasonis ATCC 8503 chromosome, complete genome	gi|150006674|ref|NC_009615.1| Parabacteroides distasonis ATCC 8503 chromosome, complete genome

contig-100_569	1796	N	2	1	Sipho	1	0	2	NA	Acidaminococcus intestini RyC-MR95, complete genome	gi|550916528|ref|NC_022571.1| Clostridium saccharobutylicum DSM 13864, complete genome	gi|550916528|ref|NC_022571.1| Clostridium saccharobutylicum DSM 13864, complete genome
contig-100_57	7673	N	8	2	Sipho	0	0	3	NA	Unidentified phage clone 2019_scaffold132 genomic sequence	NA	Unidentified phage clone 2019_scaffold132 genomic sequence
contig-100_572	1785	N	2	0	NA	0	0	0	NA	NA	NA	NA
contig-100_574	1782	N	1	0	NA	0	0	0	NA	Uncultured organism clone 1041059767240 genomic sequence	NA	Uncultured organism clone 1041059767240 genomic sequence
contig-100_575	1782	N	1	1	Sipho	0	0	1	NA	Streptococcus phage 858, complete genome	gi|399498678|ref|NC_018285.1| Streptococcus phage YMC-2011, complete genome	gi|399498678|ref|NC_018285.1| Streptococcus phage YMC-2011, complete genome
contig-100_577	1777	N	0	0	NA	0	0	0	NA	Uncultured organism clone VC1BZ28TR genomic sequence	gi|319899888|ref|NC_014933.1| Bacteroides helcogenes P 36-108 chromosome, complete genome	gi|319899888|ref|NC_014933.1| Bacteroides helcogenes P 36-108 chromosome, complete genome
contig-100_580	1770	N	2	0	NA	0	0	0	NA	NA	NA	NA
contig-100_581	1769	N	2	0	NA	0	0	0	NA	NA	NA	NA
contig-100_583	1766	N	0	0	NA	0	0	0	NA	NA	NA	NA
contig-100_585	1764	N	1	0	NA	0	0	0	NA	NA	NA	NA
contig-100_586	1761	N	0	0	NA	0	0	0	NA	NA	NA	NA
contig-100_587	1760	N	2	0	NA	0	1	1	NA	NA	NA	NA

contig-100_588	1760	N	1	0	NA	0	0	0	NA	Propionibacterium acnes TypeIA2 P.acn33, complete genome	gi|365972921|ref|NC_016516.1| Propionibacterium acnes TypeIA2 P.acn33 chromosome, complete genome	gi|365972921|ref|NC_016516.1| Propionibacterium acnes TypeIA2 P.acn33 chromosome, complete genome
contig-100_589	1759	N	1	0	NA	0	0	0	NA	Uncultured organism clone 1041059765097 genomic sequence	NA	Uncultured organism clone 1041059765097 genomic sequence
contig-100_594	1751	N	1	0	NA	0	0	0	NA	Unidentified phage clone 2020_scaffold1264 genomic sequence	NA	Unidentified phage clone 2020_scaffold1264 genomic sequence
contig-100_595	1751	N	2	0	NA	0	0	0	NA	Haemophilus parainfluenzae T3T1 complete genome	gi|345428590|ref|NC_015964.1| Haemophilus parainfluenzae T3T1, complete genome	gi|345428590|ref|NC_015964.1| Haemophilus parainfluenzae T3T1, complete genome
contig-100_597	1744	N	0	0	NA	0	0	0	NA	Streptococcus phage YMC-2011, complete genome	gi|399498678|ref|NC_018285.1| Streptococcus phage YMC-2011, complete genome	gi|399498678|ref|NC_018285.1| Streptococcus phage YMC-2011, complete genome
contig-100_598	1741	N	1	0	NA	0	0	0	NA	NA	NA	NA
contig-100_599	1740	N	1	0	NA	0	0	0	NA	NA	NA	NA
contig-100_6	29056	N	26	9	Sipho	1	1	10	NA	Unidentified phage clone 2204_scaffold812 genomic sequence	NA	Unidentified phage clone 2204_scaffold812 genomic sequence
contig-100_600	1735	N	1	0	NA	0	0	0	NA	NA	NA	NA
contig-100_601	1734	N	3	0	NA	0	0	0	NA	NA	NA	NA
contig-100_603	1724	N	1	0	NA	0	0	0	NA	NA	NA	NA
contig-100_605	1720	N	1	0	NA	0	0	0	NA	NA	NA	NA
contig-100_607	1717	N	2	0	NA	0	0	0	NA	NA	NA	NA
contig-100_608	1716	N	2	0	NA	0	0	0	NA	NA	NA	NA

contig-100_609	1716	N	2	0	NA	0	0	0	NA	NA	NA	NA
contig-100_611	1712	N	1	0	NA	0	0	0	NA	NA	NA	NA
contig-100_613	1706	N	1	0	NA	0	0	0	NA	Uncultured organism clone 1041059764488 genomic sequence	gi|53711291|ref|NC_006347.1| Bacteroides fragilis YCH46 DNA, complete genome	gi|53711291|ref|NC_006347.1| Bacteroides fragilis YCH46 DNA, complete genome
contig-100_615	1702	N	1	0	NA	0	0	0	NA	NA	NA	NA
contig-100_617	1699	N	2	0	NA	0	0	1	NA	Burkholderia xenovorans LB400 chromosome 3, complete sequence	gi|91780071|ref|NC_007953.1| Burkholderia xenovorans LB400 chromosome 3, complete genome	gi|91780071|ref|NC_007953.1| Burkholderia xenovorans LB400 chromosome 3, complete genome
contig-100_618	1697	N	0	0	NA	0	0	0	NA	NA	NA	NA
contig-100_619	1692	N	3	0	NA	0	0	0	NA	Klebsiella pneumoniae subsp. pneumoniae 1084, complete genome	gi|550443072|ref|NC_022566.1| Klebsiella pneumoniae CG43, complete genome	gi|550443072|ref|NC_022566.1| Klebsiella pneumoniae CG43, complete genome
contig-100_620	1690	N	2	1	NA	0	0	0	NA	Uncultured organism clone 1041059767458 genomic sequence	gi|188993864|ref|NC_010729.1| Porphyromonas gingivalis ATCC 33277, complete genome	gi|188993864|ref|NC_010729.1| Porphyromonas gingivalis ATCC 33277, complete genome
contig-100_621	1690	N	2	0	NA	0	0	0	NA	Uncultured organism clone VC1C429TR genomic sequence	gi|479208076|ref|NC_021042.1| Faecalibacterium prausnitzii L2-6, complete genome	gi|479208076|ref|NC_021042.1| Faecalibacterium prausnitzii L2-6, complete genome
contig-100_622	1689	N	1	0	NA	0	0	0	NA	NA	NA	NA

contig-100_623	1688	N	0	0	NA	0	0	0	NA	Parabacteroides distasonis ATCC 8503, complete genome	gi|150006674|ref|NC_009615.1| Parabacteroides distasonis ATCC 8503 chromosome, complete genome	gi|150006674|ref|NC_009615.1| Parabacteroides distasonis ATCC 8503 chromosome, complete genome
contig-100_624	1688	N	2	0	NA	0	0	0	NA	Klebsiella pneumoniae subsp. rhinoscleromatis strain SB3432, complete genome	gi|550443072|ref|NC_022566.1| Klebsiella pneumoniae CG43, complete genome	gi|550443072|ref|NC_022566.1| Klebsiella pneumoniae CG43, complete genome
contig-100_625	1686	N	1	0	NA	0	0	0	NA	Haemophilus ducreyi strain 35000HP complete genome	gi|33151282|ref|NC_002940.2| Haemophilus ducreyi 35000HP chromosome, complete genome	gi|33151282|ref|NC_002940.2| Haemophilus ducreyi 35000HP chromosome, complete genome
contig-100_626	1681	N	2	0	NA	0	0	0	NA	Clostridiales sp. SSC/2 draft genome	gi|479158859|ref|NC_021016.1| Butyrate-producing bacterium SSC/2, complete genome	gi|479158859|ref|NC_021016.1| Butyrate-producing bacterium SSC/2, complete genome
contig-100_627	1680	N	1	0	NA	0	0	0	NA	Faecalibacterium prausnitzii SL3/3 draft genome	gi|479170689|ref|NC_021020.1| Faecalibacterium prausnitzii SL3/3 draft genome	gi|479170689|ref|NC_021020.1| Faecalibacterium prausnitzii SL3/3 draft genome
contig-100_628	1675	N	1	0	NA	0	0	0	NA	Uncultured organism clone VC1A837TR genomic sequence	NA	Uncultured organism clone VC1A837TR genomic sequence

contig-100_63	7045	N	6	0	NA	0	0	0	NA	Clostridium saccharolyticum-like K10 draft genome	gi|479336697|ref|NC_021047.1| Clostridium cf. saccharolyticum K10, complete genome	gi|479336697|ref|NC_021047.1| Clostridium cf. saccharolyticum K10, complete genome
contig-100_631	1660	N	1	0	NA	0	0	0	NA	Klebsiella pneumoniae KCTC 2242, complete genome	gi|386032579|ref|NC_017540.1| Klebsiella pneumoniae KCTC 2242 chromosome, complete genome	gi|386032579|ref|NC_017540.1| Klebsiella pneumoniae KCTC 2242 chromosome, complete genome
contig-100_634	1657	N	3	0	NA	0	0	0	NA	Bacteroides vulgatus ATCC 8482, complete genome	gi|150002608|ref|NC_009614.1| Bacteroides vulgatus ATCC 8482 chromosome, complete genome	gi|150002608|ref|NC_009614.1| Bacteroides vulgatus ATCC 8482 chromosome, complete genome
contig-100_636	1654	N	1	0	NA	0	0	0	NA	NA	NA	NA
contig-100_638	1652	N	1	0	NA	0	0	0	NA	Roseburia intestinalis XB6B4 draft genome	gi|479146200|ref|NC_021012.1| Roseburia intestinalis XB6B4 draft genome	gi|479146200|ref|NC_021012.1| Roseburia intestinalis XB6B4 draft genome
contig-100_640	1650	N	3	0	NA	0	0	1	NA	NA	NA	NA
contig-100_642	1647	N	1	0	NA	0	0	0	NA	Bacteroides vulgatus ATCC 8482, complete genome	gi|150002608|ref|NC_009614.1| Bacteroides vulgatus ATCC 8482 chromosome, complete genome	gi|150002608|ref|NC_009614.1| Bacteroides vulgatus ATCC 8482 chromosome, complete genome
contig-100_644	1646	N	2	0	NA	0	0	0	NA	NA	NA	NA

contig-100_645	1646	N	1	0	NA	0	0	0	NA	Pasteurella aerogenes plasmid pB1000, complete sequence	gi|190151409|ref|NC_010942.1| Actinobacillus pleuropneumoniae serovar 7 str. AP76 plasmid APP7_A, complete sequence	gi|190151409|ref|NC_010942.1| Actinobacillus pleuropneumoniae serovar 7 str. AP76 plasmid APP7_A, complete sequence
contig-100_647	1643	N	2	0	NA	0	0	0	NA	Desulfomicrobium baculatum DSM 4028, complete genome	gi|256827818|ref|NC_013173.1| Desulfomicrobium baculatum DSM 4028, complete genome	gi|256827818|ref|NC_013173.1| Desulfomicrobium baculatum DSM 4028, complete genome
contig-100_648	1642	N	1	0	NA	0	0	0	NA	NA	NA	NA
contig-100_649	1639	N	2	0	NA	0	0	0	NA	NA	NA	NA
contig-100_650	1636	N	3	0	NA	0	0	0	NA	Uncultured organism clone VC1BT17TR genomic sequence	NA	Uncultured organism clone VC1BT17TR genomic sequence
contig-100_651	1635	N	0	0	NA	0	0	0	NA	NA	NA	NA
contig-100_652	1632	N	0	0	NA	0	0	0	NA	Bacteroides thetaiotaomicron VPI-5482, complete genome	gi|29345410|ref|NC_004663.1| Bacteroides thetaiotaomicron VPI-5482 chromosome, complete genome	gi|29345410|ref|NC_004663.1| Bacteroides thetaiotaomicron VPI-5482 chromosome, complete genome
contig-100_653	1626	N	2	1	Sipho	0	0	1	NA	NA	NA	NA
contig-100_654	1626	N	1	0	NA	0	0	0	NA	Uncultured organism clone CY_F2DDb01_06 putative D-alanyl-alanine synthetase A gene, partial cds	NA	Uncultured organism clone CY_F2DDb01_06 putative D-alanyl-alanine synthetase A gene, partial cds
contig-100_655	1623	N	1	0	NA	0	0	0	NA	NA	NA	NA

contig-100_656	1622	N	0	0	NA	0	0	0	NA	NA	NA	NA
contig-100_657	1618	N	1	1	Sipho	1	0	1	NA	NA	NA	NA
contig-100_659	1615	N	2	0	NA	0	0	0	NA	Pseudomonas fluorescens SBW25 complete genome	gi|229587578|ref|NC_012660.1| Pseudomonas fluorescens SBW25 chromosome, complete genome	gi|229587578|ref|NC_012660.1| Pseudomonas fluorescens SBW25 chromosome, complete genome
contig-100_660	1615	N	1	0	NA	0	0	0	NA	NA	NA	NA
contig-100_661	1614	N	1	0	NA	0	0	0	NA	Faecalibacterium prausnitzii SL3/3 draft genome	gi|479170689|ref|NC_021020.1| Faecalibacterium prausnitzii SL3/3 draft genome	gi|479170689|ref|NC_021020.1| Faecalibacterium prausnitzii SL3/3 draft genome
contig-100_662	1612	N	2	0	NA	0	0	0	NA	NA	NA	NA
contig-100_663	1612	N	3	0	NA	0	0	0	NA	NA	NA	NA
contig-100_664	1611	N	1	0	NA	0	0	0	NA	NA	NA	NA
contig-100_666	1608	N	1	1	Sipho	0	1	1	NA	NA	NA	NA
contig-100_668	1606	N	0	0	NA	0	0	0	NA	Faecalibacterium prausnitzii SL3/3 draft genome	gi|479170689|ref|NC_021020.1| Faecalibacterium prausnitzii SL3/3 draft genome	gi|479170689|ref|NC_021020.1| Faecalibacterium prausnitzii SL3/3 draft genome
contig-100_669	1606	N	1	0	NA	0	0	0	NA	NA	NA	NA
contig-100_67	6584	N	2	0	NA	0	0	0	NA	Bacteroides vulgatus ATCC 8482, complete genome	gi|150002608|ref|NC_009614.1| Bacteroides vulgatus ATCC 8482 chromosome, complete genome	gi|150002608|ref|NC_009614.1| Bacteroides vulgatus ATCC 8482 chromosome, complete genome
contig-100_670	1606	N	1	0	NA	0	0	0	NA	NA	NA	NA
contig-100_671	1605	N	1	0	NA	0	0	0	NA	NA	NA	NA
contig-100_672	1605	N	1	0	NA	0	0	0	NA	NA	NA	NA

contig-100_673	1600	N	2	0	NA	0	0	0	NA	Clostridiales sp. SSC/2 draft genome	gi|479158859|ref|NC_021016.1| Butyrate-producing bacterium SSC/2, complete genome	gi|479158859|ref|NC_021016.1| Butyrate-producing bacterium SSC/2, complete genome
contig-100_674	1600	N	1	0	NA	0	0	0	NA	NA	NA	NA
contig-100_676	1598	N	1	0	NA	0	0	0	NA	NA	NA	NA
contig-100_677	1597	N	3	0	NA	0	0	0	NA	Roseburia hominis A2-183, complete genome	gi|347530298|ref|NC_015977.1| Roseburia hominis A2-183 chromosome, complete genome	gi|347530298|ref|NC_015977.1| Roseburia hominis A2-183 chromosome, complete genome
contig-100_678	1596	N	1	0	NA	0	0	0	NA	NA	NA	NA
contig-100_679	1595	N	1	0	NA	0	0	0	NA	NA	NA	NA
contig-100_681	1592	N	2	0	NA	0	0	0	NA	NA	NA	NA
contig-100_683	1591	N	1	0	NA	0	0	0	NA	NA	NA	NA
contig-100_684	1590	N	0	0	NA	0	0	0	NA	Eubacterium rectale ATCC 33656, complete genome	gi|238922432|ref|NC_012781.1| Eubacterium rectale ATCC 33656, complete genome	gi|238922432|ref|NC_012781.1| Eubacterium rectale ATCC 33656, complete genome
contig-100_685	1589	N	0	0	NA	0	0	0	NA	NA	NA	NA
contig-100_686	1585	N	2	0	NA	0	0	0	NA	Bacteroides vulgatus ATCC 8482, complete genome	gi|150002608|ref|NC_009614.1| Bacteroides vulgatus ATCC 8482 chromosome, complete genome	gi|150002608|ref|NC_009614.1| Bacteroides vulgatus ATCC 8482 chromosome, complete genome
contig-100_689	1580	N	2	0	NA	0	0	0	NA	NA	NA	NA
contig-100_69	6485	N	5	3	Sipho	0	0	3	NA	Unidentified phage clone 2019_scaffold132 genomic sequence	NA	Unidentified phage clone 2019_scaffold132 genomic sequence
contig-100_690	1579	N	2	0	NA	0	0	0	NA	NA	NA	NA

contig-100_691	1571	N	2	0	NA	0	0	0	NA	Uncultured organism clone VC1CF22TR genomic sequence	gi|53711291|ref|NC_006347.1| Bacteroides fragilis YCH46 DNA, complete genome	gi|53711291|ref|NC_006347.1| Bacteroides fragilis YCH46 DNA, complete genome
contig-100_692	1571	N	0	0	NA	0	0	0	NA	NA	NA	NA
contig-100_693	1569	N	1	0	NA	0	0	0	NA	NA	NA	NA
contig-100_694	1568	N	1	0	NA	0	0	0	NA	NA	NA	NA
contig-100_695	1566	N	0	0	NA	0	0	0	NA	NA	NA	NA
contig-100_696	1565	N	0	0	NA	0	0	0	NA	NA	NA	NA
contig-100_697	1565	N	1	0	NA	0	0	0	NA	NA	NA	NA
contig-100_698	1564	N	0	0	NA	0	0	0	NA	NA	NA	NA
contig-100_699	1562	N	1	0	NA	0	0	0	NA	NA	NA	NA
contig-100_700	1562	N	1	0	NA	0	0	0	NA	NA	NA	NA
contig-100_701	1561	N	0	0	NA	0	0	0	NA	NA	NA	NA
contig-100_702	1561	N	1	0	NA	0	0	0	NA	Ruminococcus torques L2-14 draft genome	gi|479155735|ref|NC_021015.1| Ruminococcus torques L2-14 draft genome	gi|479155735|ref|NC_021015.1| Ruminococcus torques L2-14 draft genome
contig-100_704	1556	N	0	0	NA	0	0	0	NA	NA	NA	NA
contig-100_705	1552	N	1	0	NA	0	0	0	NA	Escherichia coli str. K-12 substr. MG1655, complete genome	gi|388476123|ref|NC_007779.1| Escherichia coli str. K-12 substr. W3110, complete genome	gi|388476123|ref|NC_007779.1| Escherichia coli str. K-12 substr. W3110, complete genome
contig-100_706	1552	N	2	1	Sipho	1	0	1	NA	NA	NA	NA
contig-100_707	1551	N	2	0	NA	0	0	0	NA	Haemophilus parainfluenzae T3T1 complete genome	gi|345428590|ref|NC_015964.1| Haemophilus parainfluenzae T3T1, complete genome	gi|345428590|ref|NC_015964.1| Haemophilus parainfluenzae T3T1, complete genome
contig-100_708	1550	N	0	0	NA	0	0	0	NA	NA	NA	NA

contig-100_71	6395	N	5	2	Sipho	0	0	3	NA	Unidentified phage clone 2019_scaffold132 genomic sequence	NA	Unidentified phage clone 2019_scaffold 132 genomic sequence
contig-100_710	1549	N	1	0	NA	0	0	0	NA	NA	NA	NA
contig-100_711	1548	N	0	0	NA	0	0	0	NA	Bacteroides vulgatus ATCC 8482, complete genome	gi|150002608|ref|NC_009614.1| Bacteroides vulgatus ATCC 8482 chromosome, complete genome	gi|150002608|ref|NC_009614.1| Bacteroides vulgatus ATCC 8482 chromosome, complete genome
contig-100_712	1546	N	0	0	NA	0	0	0	NA	NA	NA	NA
contig-100_713	1544	N	1	1	Sipho	0	0	1	NA	NA	NA	NA
contig-100_714	1541	N	2	0	NA	0	0	0	NA	NA	NA	NA
contig-100_715	1540	N	1	0	NA	0	0	0	NA	NA	NA	NA
contig-100_719	1534	N	1	0	NA	0	1	1	NA	NA	NA	NA
contig-100_72	6360	N	8	1	Sipho	0	0	2	NA	NA	NA	NA
contig-100_720	1532	N	1	0	NA	0	0	0	NA	NA	NA	NA
contig-100_721	1531	N	2	0	NA	0	0	0	NA	NA	NA	NA
contig-100_722	1530	N	1	0	NA	0	0	0	NA	Uncultured organism clone 19 genomic sequence	gi|479210985|ref|NC_021043.1| Eubacterium siraeum V10Sc8a draft genome	gi|479210985|ref|NC_021043.1| Eubacterium siraeum V10Sc8a draft genome
contig-100_723	1530	N	1	0	NA	0	0	0	NA	Haemophilus parainfluenzae T3T1 complete genome	gi|345428590|ref|NC_015964.1| Haemophilus parainfluenzae T3T1, complete genome	gi|345428590|ref|NC_015964.1| Haemophilus parainfluenzae T3T1, complete genome
contig-100_724	1528	N	0	0	NA	0	0	0	NA	NA	NA	NA
contig-100_725	1527	N	1	0	NA	0	0	0	NA	NA	NA	NA

contig-100_727	1522	N	1	0	NA	0	0	0	NA	Klebsiella pneumoniae subsp. rhinoscleromatis strain SB3432, complete genome	gi|529985600|ref|NC_021232.1| Klebsiella pneumoniae subsp. rhinoscleromatis strain SB3432, complete genome	gi|529985600|ref|NC_021232.1| Klebsiella pneumoniae subsp. rhinoscleromatis strain SB3432, complete genome
contig-100_729	1518	N	1	0	NA	0	0	0	NA	Clostridium saccharolyticum-like K10 draft genome	gi|479336697|ref|NC_021047.1| Clostridium cf. saccharolyticum K10, complete genome	gi|479336697|ref|NC_021047.1| Clostridium cf. saccharolyticum K10, complete genome
contig-100_73	6308	N	6	3	Myo	0	0	4	NA	Uncultured organism clone VC1CQ56TF genomic sequence	NA	Uncultured organism clone VC1CQ56TF genomic sequence
contig-100_730	1517	N	1	0	NA	0	0	0	NA	Bacteroides xylanisolvens XB1A draft genome	gi|479162165|ref|NC_021017.1| Bacteroides xylanisolvens XB1A draft genome	gi|479162165|ref|NC_021017.1| Bacteroides xylanisolvens XB1A draft genome
contig-100_731	1511	N	1	0	NA	0	0	0	NA	Bacteroides xylanisolvens XB1A draft genome	gi|479162165|ref|NC_021017.1| Bacteroides xylanisolvens XB1A draft genome	gi|479162165|ref|NC_021017.1| Bacteroides xylanisolvens XB1A draft genome
contig-100_732	1510	N	1	0	NA	0	0	0	NA	NA	gi|554463091|ref|NC_022593.1| Lactococcus lactis subsp. lactis KLDS 4.0325, complete genome	gi|554463091|ref|NC_022593.1| Lactococcus lactis subsp. lactis KLDS 4.0325, complete genome

contig-100_734	1510	N	0	0	NA	0	0	0	NA	Veillonella parvula DSM 2008, complete genome	gi|269797069|ref|NC_013520.1| Veillonella parvula DSM 2008 chromosome, complete genome	gi|269797069|ref|NC_013520.1| Veillonella parvula DSM 2008 chromosome, complete genome
contig-100_735	1510	N	0	0	NA	0	0	0	NA	NA	NA	NA
contig-100_736	1510	N	1	0	NA	0	0	0	NA	Bacteroides vulgatus ATCC 8482, complete genome	gi|150002608|ref|NC_009614.1| Bacteroides vulgatus ATCC 8482 chromosome, complete genome	gi|150002608|ref|NC_009614.1| Bacteroides vulgatus ATCC 8482 chromosome, complete genome
contig-100_737	1509	N	1	0	NA	0	0	0	NA	NA	NA	NA
contig-100_738	1508	N	0	0	NA	0	0	0	NA	NA	NA	NA
contig-100_739	1508	N	1	0	NA	0	0	0	NA	Eubacterium siraeum V10Sc8a draft genome	gi|479210985|ref|NC_021043.1| Eubacterium siraeum V10Sc8a draft genome	gi|479210985|ref|NC_021043.1| Eubacterium siraeum V10Sc8a draft genome
contig-100_74	6279	N	1	0	NA	0	0	0	NA	NA	NA	NA
contig-100_740	1501	N	0	0	NA	0	0	0	NA	Clostridiales sp. SSC/2 draft genome	gi|479158859|ref|NC_021016.1| Butyrate-producing bacterium SSC/2, complete genome	gi|479158859|ref|NC_021016.1| Butyrate-producing bacterium SSC/2, complete genome
contig-100_742	1498	N	2	0	NA	0	0	0	NA	Bacteroides fragilis plasmid pBFUK1 DNA, complete genome, strain: GAI92082	NA	Bacteroides fragilis plasmid pBFUK1 DNA, complete genome, strain: GAI92082
contig-100_744	1496	N	1	0	NA	0	0	0	NA	NA	NA	NA
contig-100_745	1495	N	1	0	NA	0	0	0	NA	NA	NA	NA
contig-100_746	1493	N	0	0	NA	0	0	0	NA	NA	NA	NA
contig-100_747	1492	N	0	0	NA	0	0	0	NA	NA	NA	NA

contig-100_748	1491	N	0	0	NA	0	0	0	NA	NA	NA	NA
contig-100_749	1491	N	2	0	NA	0	0	0	NA	NA	NA	NA
contig-100_750	1490	N	2	0	NA	0	0	0	NA	Clostridium saccharolyticum-like K10 draft genome	gi|479336697|ref|NC_021047.1| Clostridium cf. saccharolyticum K10, complete genome	gi|479336697|ref|NC_021047.1| Clostridium cf. saccharolyticum K10, complete genome
contig-100_751	1488	N	2	0	NA	0	0	0	NA	NA	NA	NA
contig-100_752	1484	N	0	0	NA	0	0	0	NA	NA	NA	NA
contig-100_753	1483	N	2	0	NA	0	0	0	NA	Escherichia coli str. K-12 substr. MG1655, complete genome	gi|388476123|ref|NC_007779.1| Escherichia coli str. K-12 substr. W3110, complete genome	gi|388476123|ref|NC_007779.1| Escherichia coli str. K-12 substr. W3110, complete genome
contig-100_756	1482	N	2	0	NA	0	0	0	NA	NA	NA	NA
contig-100_757	1481	N	0	0	NA	0	0	0	NA	NA	NA	NA
contig-100_758	1480	N	0	0	NA	0	0	0	NA	NA	NA	NA
contig-100_759	1477	N	1	0	NA	0	0	0	NA	NA	NA	NA
contig-100_760	1470	N	1	0	NA	0	0	0	NA	NA	NA	NA
contig-100_761	1470	N	1	0	NA	0	0	0	NA	Clostridiales sp. SM4/1 draft genome	gi|479181986|ref|NC_021024.1| Butyrate-producing bacterium SM4/1, complete genome	gi|479181986|ref|NC_021024.1| Butyrate-producing bacterium SM4/1, complete genome
contig-100_765	1467	N	1	0	NA	0	0	0	NA	Odoribacter splanchnicus DSM 20712, complete genome	gi|325278757|ref|NC_015160.1| Odoribacter splanchnicus DSM 20712 chromosome, complete genome	gi|325278757|ref|NC_015160.1| Odoribacter splanchnicus DSM 20712 chromosome, complete genome
contig-100_766	1465	N	1	0	NA	0	0	0	NA	NA	NA	NA
contig-100_767	1464	N	3	0	NA	0	0	0	NA	NA	NA	NA
contig-100_768	1464	N	2	0	NA	0	0	0	NA	NA	NA	NA
contig-100_769	1463	N	1	0	NA	0	0	0	NA	NA	NA	NA

contig-100_77	6042	N	5	1	Myo	0	0	2	NA	Propionibacterium acnes ATCC 11828, complete genome	gi|386069650|ref|NC_017550.1| Propionibacterium acnes ATCC 11828 chromosome, complete genome	gi|386069650|ref|NC_017550.1| Propionibacterium acnes ATCC 11828 chromosome, complete genome
contig-100_772	1461	N	1	0	NA	0	0	0	NA	Bacteroides helcogenes P 36-108, complete genome	gi|319899888|ref|NC_014933.1| Bacteroides helcogenes P 36-108 chromosome, complete genome	gi|319899888|ref|NC_014933.1| Bacteroides helcogenes P 36-108 chromosome, complete genome
contig-100_773	1460	N	1	0	NA	0	0	0	NA	NA	NA	NA
contig-100_774	1459	N	1	1	Sipho	0	0	1	NA	Aggregatibacter actinomycetemcomitans ANH9381, complete genome	gi|365966241|ref|NC_016513.1| Aggregatibacter actinomycetemcomitans ANH9381 chromosome, complete genome	gi|365966241|ref|NC_016513.1| Aggregatibacter actinomycetemcomitans ANH9381 chromosome, complete genome
contig-100_775	1459	N	1	0	NA	0	0	0	NA	Uncultured bacterium clone HA0AAA4ZF07RM1 genomic sequence	NA	Uncultured bacterium clone HA0AAA4ZF07RM1 genomic sequence
contig-100_776	1459	N	1	0	NA	0	0	0	NA	NA	NA	NA
contig-100_777	1458	N	2	0	NA	0	0	0	NA	NA	NA	NA
contig-100_778	1457	N	1	0	NA	0	0	0	NA	Uncultured organism clone 1041059765702 genomic sequence	gi|319899888|ref|NC_014933.1| Bacteroides helcogenes P 36-108 chromosome, complete genome	gi|319899888|ref|NC_014933.1| Bacteroides helcogenes P 36-108 chromosome, complete genome

contig-100_779	1457	N	3	0	NA	0	0	0	NA	Bacteroides vulgatus ATCC 8482, complete genome	gi|150002608|ref|NC_009614.1| Bacteroides vulgatus ATCC 8482 chromosome, complete genome	gi|150002608|ref|NC_009614.1| Bacteroides vulgatus ATCC 8482 chromosome, complete genome
contig-100_78	5863	N	5	0	NA	0	0	1	NA	NA	NA	NA
contig-100_780	1454	N	1	0	NA	0	0	0	NA	Uncultured organism clone VC1A837TR genomic sequence	gi|319899888|ref|NC_014933.1| Bacteroides helcogenes P 36-108 chromosome, complete genome	gi|319899888|ref|NC_014933.1| Bacteroides helcogenes P 36-108 chromosome, complete genome
contig-100_781	1454	N	3	0	NA	0	0	0	NA	NA	NA	NA
contig-100_782	1452	N	2	0	NA	0	0	0	NA	Ruminococcus obeum A2-162 draft genome	gi|479176048|ref|NC_021022.1| Ruminococcus obeum A2-162 draft genome	gi|479176048|ref|NC_021022.1| Ruminococcus obeum A2-162 draft genome
contig-100_783	1452	N	1	0	NA	0	0	0	NA	Eubacterium rectale DSM 17629 draft genome	gi|479140210|ref|NC_021010.1| Eubacterium rectale DSM 17629 draft genome	gi|479140210|ref|NC_021010.1| Eubacterium rectale DSM 17629 draft genome
contig-100_784	1450	N	1	0	NA	0	0	0	NA	Uncultured organism clone VC1A303TR genomic sequence	gi|325297172|ref|NC_015164.1| Bacteroides salanitronis DSM 18170 chromosome, complete genome	gi|325297172|ref|NC_015164.1| Bacteroides salanitronis DSM 18170 chromosome, complete genome
contig-100_785	1450	N	2	0	NA	0	0	0	NA	NA	NA	NA
contig-100_786	1449	N	1	0	NA	0	0	0	NA	NA	NA	NA
contig-100_787	1449	N	0	0	NA	0	0	0	NA	NA	NA	NA

contig-100_788	1445	N	2	0	NA	0	0	1	NA	Uncultured organism clone 1041059765405 genomic sequence	gi|387131618|ref|NC_017860.1| Prevotella intermedia 17 chromosome chromosome I, complete sequence	gi|387131618|ref|NC_017860.1| Prevotella intermedia 17 chromosome chromosome I, complete sequence
contig-100_789	1445	N	2	0	NA	0	0	0	NA	Haemophilus influenzae F3047 complete genome	gi|319774951|ref|NC_014922.1| Haemophilus influenzae F3047 chromosome, complete genome	gi|319774951|ref|NC_014922.1| Haemophilus influenzae F3047 chromosome, complete genome
contig-100_790	1443	N	1	0	NA	0	0	0	NA	NA	NA	NA
contig-100_791	1442	N	3	0	NA	0	0	0	NA	Bacteroides vulgatus ATCC 8482, complete genome	gi|150002608|ref|NC_009614.1| Bacteroides vulgatus ATCC 8482 chromosome, complete genome	gi|150002608|ref|NC_009614.1| Bacteroides vulgatus ATCC 8482 chromosome, complete genome
contig-100_793	1442	N	1	0	NA	0	0	0	NA	Unidentified phage clone 1013_scaffold1563 genomic sequence	NA	Unidentified phage clone 1013_scaffold1563 genomic sequence
contig-100_795	1441	N	2	0	NA	0	0	0	NA	Unidentified phage clone 2019_scaffold132 genomic sequence	NA	Unidentified phage clone 2019_scaffold132 genomic sequence
contig-100_796	1439	N	2	1	NA	0	1	1	NA	Bacteroides salanitronis DSM 18170, complete genome	gi|325297172|ref|NC_015164.1| Bacteroides salanitronis DSM 18170 chromosome, complete genome	gi|325297172|ref|NC_015164.1| Bacteroides salanitronis DSM 18170 chromosome, complete genome
contig-100_799	1438	N	3	0	NA	0	0	0	NA	NA	NA	NA

contig-100_8	24355	N	15	6	Sipho	0	0	3	NA	Faecalibacterium prausnitzii SL3/3 draft genome	gi|479170689|ref|NC_021020.1| Faecalibacterium prausnitzii SL3/3 draft genome	gi|479170689|ref|NC_021020.1| Faecalibacterium prausnitzii SL3/3 draft genome
contig-100_80	5833	N	7	5	Podo	0	0	5	NA	NA	NA	NA
contig-100_801	1435	N	0	0	NA	0	0	0	NA	NA	NA	NA
contig-100_802	1435	N	1	0	NA	0	0	0	NA	Uncultured organism clone VC1CK33TR genomic sequence	NA	Uncultured organism clone VC1CK33TR genomic sequence
contig-100_803	1433	N	1	0	NA	0	0	0	NA	Faecalibacterium prausnitzii SL3/3 draft genome	gi|479170689|ref|NC_021020.1| Faecalibacterium prausnitzii SL3/3 draft genome	gi|479170689|ref|NC_021020.1| Faecalibacterium prausnitzii SL3/3 draft genome
contig-100_805	1429	N	1	0	NA	0	0	0	NA	NA	NA	NA
contig-100_806	1429	N	1	0	NA	0	0	0	NA	NA	NA	NA
contig-100_807	1427	N	1	0	NA	0	0	0	NA	Haemophilus parainfluenzae T3T1 complete genome	gi|345428590|ref|NC_015964.1| Haemophilus parainfluenzae T3T1, complete genome	gi|345428590|ref|NC_015964.1| Haemophilus parainfluenzae T3T1, complete genome
contig-100_808	1426	N	3	0	NA	0	0	0	NA	NA	NA	NA
contig-100_809	1426	N	1	0	NA	0	0	0	NA	NA	NA	NA
contig-100_81	5794	N	4	0	NA	0	1	0	NA	Faecalibacterium prausnitzii L2/6 draft genome	gi|479208076|ref|NC_021042.1| Faecalibacterium prausnitzii L2-6, complete genome	gi|479208076|ref|NC_021042.1| Faecalibacterium prausnitzii L2-6, complete genome
contig-100_810	1422	N	1	0	NA	0	0	0	NA	NA	NA	NA
contig-100_812	1418	N	1	0	NA	0	0	0	NA	NA	NA	NA
contig-100_813	1418	N	1	0	NA	0	0	0	NA	NA	NA	NA
contig-100_814	1417	N	1	0	NA	0	0	0	NA	NA	NA	NA
contig-100_816	1414	N	0	0	NA	0	0	0	NA	NA	NA	NA
contig-100_817	1413	N	1	0	NA	0	0	0	NA	NA	NA	NA
contig-100_818	1413	N	0	0	NA	0	0	0	NA	NA	NA	NA

contig-100_819	1412	N	0	0	NA	0	0	0	NA	Faecalibacterium prausnitzii L2/6 draft genome	gi|479208076|ref|NC_021042.1| Faecalibacterium prausnitzii L2-6, complete genome	gi|479208076|ref|NC_021042.1| Faecalibacterium prausnitzii L2-6, complete genome
contig-100_820	1412	N	1	0	NA	0	0	0	NA	NA	NA	NA
contig-100_821	1412	N	2	0	NA	0	0	0	NA	NA	NA	NA
contig-100_823	1410	N	2	0	NA	0	0	0	NA	NA	NA	NA
contig-100_824	1410	N	1	0	NA	0	0	0	NA	NA	NA	NA
contig-100_825	1410	N	0	0	NA	0	0	0	NA	NA	NA	NA
contig-100_826	1410	N	2	0	NA	0	0	0	NA	NA	NA	NA
contig-100_827	1408	N	1	0	NA	0	0	0	NA	Haemophilus influenzae KR494, complete genome	gi|543951066|ref|NC_022356.1| Haemophilus influenzae KR494, complete genome	gi|543951066|ref|NC_022356.1| Haemophilus influenzae KR494, complete genome
contig-100_828	1408	N	0	0	NA	0	0	0	NA	NA	NA	NA
contig-100_829	1407	N	0	0	NA	0	0	0	NA	NA	NA	NA
contig-100_83	5744	Y	6	3	Micro	0	0	3	NA	NA	NA	NA
contig-100_830	1407	N	1	0	NA	0	0	0	NA	NA	NA	NA
contig-100_832	1406	N	0	0	NA	0	0	0	NA	NA	NA	NA
contig-100_833	1405	N	1	0	NA	0	0	0	NA	Haemophilus parainfluenzae strain 72322 plasmid, complete sequence	NA	Haemophilus parainfluenzae strain 72322 plasmid, complete sequence
contig-100_834	1405	N	1	0	NA	0	0	0	NA	Faecalibacterium prausnitzii L2/6 draft genome	gi|479208076|ref|NC_021042.1| Faecalibacterium prausnitzii L2-6, complete genome	gi|479208076|ref|NC_021042.1| Faecalibacterium prausnitzii L2-6, complete genome
contig-100_835	1405	N	1	0	NA	0	0	0	NA	NA	NA	NA
contig-100_836	1405	N	1	0	NA	0	0	0	NA	NA	NA	NA
contig-100_839	1401	N	0	0	NA	0	0	0	NA	NA	NA	NA
contig-100_840	1401	N	2	0	NA	0	0	0	NA	NA	NA	NA

contig-100_841	1401	N	1	0	NA	0	0	0	NA	Odoribacter splanchnicus DSM 20712, complete genome	gi|325278757|ref|NC_015160.1| Odoribacter splanchnicus DSM 20712 chromosome, complete genome	gi|325278757|ref|NC_015160.1| Odoribacter splanchnicus DSM 20712 chromosome, complete genome
contig-100_842	1400	N	2	0	NA	0	0	0	NA	Faecalibacterium prausnitzii SL3/3 draft genome	gi|479170689|ref|NC_021020.1| Faecalibacterium prausnitzii SL3/3 draft genome	gi|479170689|ref|NC_021020.1| Faecalibacterium prausnitzii SL3/3 draft genome
contig-100_843	1394	N	1	0	NA	0	0	0	NA	TPA_exp: Clostridium difficile strain QCD-66C26 transposon Tn6110, complete sequence	gi|479150083|ref|NC_021013.1| Ruminococcus bromii L2-63 draft genome	gi|479150083|ref|NC_021013.1| Ruminococcus bromii L2-63 draft genome
contig-100_844	1392	N	1	0	NA	0	0	0	NA	NA	NA	NA
contig-100_845	1392	N	0	0	NA	0	0	0	NA	Bacteroides vulgatus ATCC 8482, complete genome	gi|150002608|ref|NC_009614.1| Bacteroides vulgatus ATCC 8482 chromosome, complete genome	gi|150002608|ref|NC_009614.1| Bacteroides vulgatus ATCC 8482 chromosome, complete genome
contig-100_846	1391	N	2	0	NA	0	0	0	NA	NA	NA	NA
contig-100_848	1389	N	1	0	NA	0	0	0	NA	Faecalibacterium prausnitzii L2/6 draft genome	gi|479208076|ref|NC_021042.1| Faecalibacterium prausnitzii L2-6, complete genome	gi|479208076|ref|NC_021042.1| Faecalibacterium prausnitzii L2-6, complete genome
contig-100_849	1387	N	0	0	NA	0	0	0	NA	Bacteroides vulgatus ATCC 8482, complete genome	gi|150002608|ref|NC_009614.1| Bacteroides vulgatus ATCC 8482 chromosome, complete genome	gi|150002608|ref|NC_009614.1| Bacteroides vulgatus ATCC 8482 chromosome, complete genome

contig-100_85	5735	N	3	0	NA	0	0	0	NA	Klebsiella pneumoniae plasmid pLVPK, complete sequence	gi|57639934|ref|NC_006625.1| Klebsiella pneumoniae NTUH-K2044 plasmid pK2044, complete sequence	gi|57639934|ref|NC_006625.1| Klebsiella pneumoniae NTUH-K2044 plasmid pK2044, complete sequence
contig-100_850	1386	N	0	0	NA	0	0	0	NA	NA	NA	NA
contig-100_852	1384	N	0	0	NA	0	0	0	NA	NA	NA	NA
contig-100_853	1383	N	1	0	NA	0	0	0	NA	NA	NA	NA
contig-100_854	1379	N	1	0	NA	0	0	0	NA	NA	NA	NA
contig-100_855	1378	N	1	0	NA	0	0	0	NA	NA	NA	NA
contig-100_856	1378	N	1	0	NA	0	0	0	NA	NA	NA	NA
contig-100_857	1378	N	1	0	NA	0	0	0	NA	NA	NA	NA
contig-100_858	1377	N	1	0	NA	0	0	0	NA	NA	NA	NA
contig-100_859	1377	N	1	0	NA	0	0	0	NA	NA	NA	NA
contig-100_86	5714	N	2	1	NA	1	0	1	NA	Uncultured bacterium 52B7 8-amino-7-oxononanoate synthase gene, partial cds; and hypothetical protein, efflux ABC transporter permease protein, putative ABC transporter ATP-binding protein, efflux ABC transporter permease proteins, hypothetical protein, efflux ABC transporter permease protein, hypothetical protein, putative outer membrane efflux protein, hypothetical proteins, putative two-component system response regulators, two-component system sensor histidine kinase, hypothetical proteins, putative dinitrogenase iron-molybdenum cofactor, hypothetical protein, putative integrase, hypothetical proteins, putative lipoprotein, and	gi|150002608|ref|NC_009614.1| Bacteroides vulgatus ATCC 8482 chromosome, complete genome	gi|150002608|ref|NC_009614.1| Bacteroides vulgatus ATCC 8482 chromosome, complete genome

contig-100_860	1376	N	1	0	NA	0	0	0	NA	Streptococcus phage Abc2, complete genome	gi|387783149|ref|NC_017595.1| Streptococcus salivarius JIM8777, complete genome	gi|387783149|ref|NC_017595.1| Streptococcus salivarius JIM8777, complete genome
contig-100_861	1376	N	0	0	NA	0	0	0	NA	NA	NA	NA
contig-100_862	1375	N	2	0	NA	0	0	0	NA	NA	NA	NA
contig-100_863	1369	N	1	0	NA	0	0	0	NA	Eubacterium rectale ATCC 33656, complete genome	gi|238922432|ref|NC_012781.1| Eubacterium rectale ATCC 33656, complete genome	gi|238922432|ref|NC_012781.1| Eubacterium rectale ATCC 33656, complete genome
contig-100_864	1366	N	1	0	NA	0	0	0	NA	Bacteroides thetaiotaomicron VPI-5482, complete genome	gi|29345410|ref|NC_004663.1| Bacteroides thetaiotaomicron VPI-5482 chromosome, complete genome	gi|29345410|ref|NC_004663.1| Bacteroides thetaiotaomicron VPI-5482 chromosome, complete genome
contig-100_865	1365	N	1	0	NA	0	0	0	NA	NA	NA	NA
contig-100_866	1363	N	1	0	NA	0	0	0	NA	NA	NA	NA
contig-100_868	1361	N	0	0	NA	0	0	0	NA	NA	NA	NA
contig-100_869	1361	N	1	0	NA	0	0	0	NA	NA	NA	NA
contig-100_870	1360	N	1	0	NA	0	0	0	NA	NA	NA	NA
contig-100_871	1360	N	1	0	NA	0	0	0	NA	NA	NA	NA
contig-100_872	1358	N	1	0	NA	0	0	0	NA	Bacteroides salanitronis DSM 18170, complete genome	gi|325297172|ref|NC_015164.1| Bacteroides salanitronis DSM 18170 chromosome, complete genome	gi|325297172|ref|NC_015164.1| Bacteroides salanitronis DSM 18170 chromosome, complete genome
contig-100_873	1357	N	0	0	NA	0	0	0	NA	Roseburia hominis A2-183, complete genome	gi|347530298|ref|NC_015977.1| Roseburia hominis A2-183 chromosome, complete genome	gi|347530298|ref|NC_015977.1| Roseburia hominis A2-183 chromosome, complete genome

contig-100_875	1355	N	1	0	NA	0	0	0	NA	NA	NA	NA
contig-100_876	1355	N	1	0	NA	0	0	0	NA	NA	NA	NA
contig-100_877	1355	N	0	0	NA	0	0	0	NA	Uncultured organism clone VC1DC74TR genomic sequence	NA	Uncultured organism clone VC1DC74TR genomic sequence
contig-100_878	1355	N	1	0	NA	0	0	0	NA	Unidentified phage clone 2019_scaffold132 genomic sequence	NA	Unidentified phage clone 2019_scaffold 132 genomic sequence
contig-100_879	1354	N	0	0	NA	0	0	0	NA	Clostridiales sp. SM4/1 draft genome	gi|479181986|ref|NC_021024.1| Butyrate-producing bacterium SM4/1, complete genome	gi|479181986|ref|NC_021024.1| Butyrate-producing bacterium SM4/1, complete genome
contig-100_88	5693	Y	5	0	NA	0	0	0	NA	Bacteroides fragilis strain P35 plasmid pBFP35, complete sequence	gi|150002608|ref|NC_009614.1| Bacteroides vulgatus ATCC 8482 chromosome, complete genome	gi|150002608|ref|NC_009614.1| Bacteroides vulgatus ATCC 8482 chromosome, complete genome
contig-100_880	1352	N	2	0	NA	0	0	0	NA	NA	NA	NA
contig-100_881	1352	N	1	0	NA	0	0	0	NA	NA	NA	NA
contig-100_882	1351	N	1	0	NA	0	0	0	NA	Streptococcus salivarius JIM8777 complete genome	gi|387783149|ref|NC_017595.1| Streptococcus salivarius JIM8777, complete genome	gi|387783149|ref|NC_017595.1| Streptococcus salivarius JIM8777, complete genome
contig-100_883	1351	N	1	0	NA	0	0	0	NA	NA	NA	NA
contig-100_884	1350	N	1	0	NA	0	0	0	NA	Streptococcus phage 5093, complete genome	NA	Streptococcus phage 5093, complete genome
contig-100_885	1349	N	2	0	NA	0	0	0	NA	NA	NA	NA

contig-100_886	1349	N	1	0	NA	0	0	0	NA	Haemophilus parainfluenzae T3T1 complete genome	gi|345428590|ref|NC_015964.1| Haemophilus parainfluenzae T3T1, complete genome	gi|345428590|ref|NC_015964.1| Haemophilus parainfluenzae T3T1, complete genome
contig-100_888	1348	N	1	0	NA	0	0	0	NA	NA	NA	NA
contig-100_889	1348	N	0	0	NA	0	0	0	NA	Haemophilus parainfluenzae T3T1 complete genome	gi|345428590|ref|NC_015964.1| Haemophilus parainfluenzae T3T1, complete genome	gi|345428590|ref|NC_015964.1| Haemophilus parainfluenzae T3T1, complete genome
contig-100_89	5691	Y	5	0	NA	0	0	0	NA	NA	NA	NA
contig-100_890	1347	N	2	0	NA	0	0	0	NA	NA	NA	NA
contig-100_892	1346	N	2	0	NA	0	0	0	NA	Faecalibacterium prausnitzii L2/6 draft genome	gi|479208076|ref|NC_021042.1| Faecalibacterium prausnitzii L2-6, complete genome	gi|479208076|ref|NC_021042.1| Faecalibacterium prausnitzii L2-6, complete genome
contig-100_893	1345	N	2	0	NA	0	0	0	NA	NA	NA	NA
contig-100_894	1342	N	2	0	NA	0	0	0	NA	NA	NA	NA
contig-100_897	1337	N	1	0	NA	0	0	0	NA	Ralstonia pickettii 12D chromosome 2, complete sequence	gi|187925958|ref|NC_010678.1| Ralstonia pickettii 12J chromosome 2, complete sequence	gi|187925958|ref|NC_010678.1| Ralstonia pickettii 12J chromosome 2, complete sequence
contig-100_898	1336	N	1	0	NA	0	0	0	NA	Eubacterium rectale DSM 17629 draft genome	gi|479140210|ref|NC_021010.1| Eubacterium rectale DSM 17629 draft genome	gi|479140210|ref|NC_021010.1| Eubacterium rectale DSM 17629 draft genome
contig-100_899	1336	N	1	0	NA	0	0	0	NA	NA	NA	NA

contig-100_901	1333	N	1	0	NA	0	0	0	NA	Faecalibacterium prausnitzii SL3/3 draft genome	gi|479170689|ref|NC_021020.1| Faecalibacterium prausnitzii SL3/3 draft genome	gi|479170689|ref|NC_021020.1| Faecalibacterium prausnitzii SL3/3 draft genome
contig-100_902	1333	N	0	0	NA	0	0	0	NA	Uncultured organism clone 1041059765496 genomic sequence	gi|150002608|ref|NC_009614.1| Bacteroides vulgatus ATCC 8482 chromosome, complete genome	gi|150002608|ref|NC_009614.1| Bacteroides vulgatus ATCC 8482 chromosome, complete genome
contig-100_903	1333	N	2	0	NA	0	0	0	NA	Faecalibacterium prausnitzii L2/6 draft genome	gi|479208076|ref|NC_021042.1| Faecalibacterium prausnitzii L2-6, complete genome	gi|479208076|ref|NC_021042.1| Faecalibacterium prausnitzii L2-6, complete genome
contig-100_905	1332	N	1	0	NA	0	0	0	NA	NA	NA	NA
contig-100_906	1332	N	2	0	NA	0	0	0	NA	Odoribacter splanchnicus DSM 20712, complete genome	gi|325278757|ref|NC_015160.1| Odoribacter splanchnicus DSM 20712 chromosome, complete genome	gi|325278757|ref|NC_015160.1| Odoribacter splanchnicus DSM 20712 chromosome, complete genome
contig-100_907	1332	N	1	0	NA	0	0	0	NA	NA	NA	NA
contig-100_909	1329	N	1	0	NA	0	0	0	NA	NA	NA	NA
contig-100_910	1328	N	1	0	NA	0	0	0	NA	Haemophilus influenzae 10810 genome	gi|378696079|ref|NC_016809.1| Haemophilus influenzae 10810, complete genome	gi|378696079|ref|NC_016809.1| Haemophilus influenzae 10810, complete genome
contig-100_911	1327	N	1	0	NA	0	0	0	NA	NA	NA	NA
contig-100_912	1325	N	2	0	NA	0	0	0	NA	NA	NA	NA

contig-100_915	1322	N	3	0	NA	0	0	0	NA	Bacteroides vulgatus ATCC 8482, complete genome	gi|150002608|ref|NC_009614.1| Bacteroides vulgatus ATCC 8482 chromosome, complete genome	gi|150002608|ref|NC_009614.1| Bacteroides vulgatus ATCC 8482 chromosome, complete genome
contig-100_916	1322	N	1	0	NA	0	0	0	NA	NA	NA	NA
contig-100_917	1321	N	1	0	NA	0	0	0	NA	NA	NA	NA
contig-100_918	1321	N	1	0	NA	0	0	0	NA	NA	NA	NA
contig-100_922	1317	N	0	0	NA	0	0	0	NA	NA	NA	NA
contig-100_923	1317	N	0	0	NA	0	0	0	NA	Faecalibacterium prausnitzii SL3/3 draft genome	gi|479170689|ref|NC_021020.1| Faecalibacterium prausnitzii SL3/3 draft genome	gi|479170689|ref|NC_021020.1| Faecalibacterium prausnitzii SL3/3 draft genome
contig-100_924	1315	N	1	0	NA	0	0	0	NA	NA	NA	NA
contig-100_925	1314	N	1	0	NA	0	0	0	NA	NA	NA	NA
contig-100_926	1314	N	3	0	NA	0	0	0	NA	Azospirillum sp. B510 DNA, complete genome	gi|288956841|ref|NC_013854.1| Azospirillum sp. B510 chromosome, complete genome	gi|288956841|ref|NC_013854.1| Azospirillum sp. B510 chromosome, complete genome
contig-100_927	1314	N	0	0	NA	0	0	0	NA	NA	NA	NA
contig-100_928	1313	N	1	0	NA	0	0	0	NA	Faecalibacterium prausnitzii L2/6 draft genome	gi|479208076|ref|NC_021042.1| Faecalibacterium prausnitzii L2-6, complete genome	gi|479208076|ref|NC_021042.1| Faecalibacterium prausnitzii L2-6, complete genome
contig-100_93	5526	N	6	0	NA	0	1	1	NA	Roseburia hominis A2-183, complete genome	gi|347530298|ref|NC_015977.1| Roseburia hominis A2-183 chromosome, complete genome	gi|347530298|ref|NC_015977.1| Roseburia hominis A2-183 chromosome, complete genome
contig-100_930	1312	N	1	0	NA	0	0	0	NA	NA	NA	NA

contig-100_931	1309	N	0	0	NA	0	0	0	NA	Haemophilus parainfluenzae T3T1 complete genome	gi|345428590|ref|NC_015964.1| Haemophilus parainfluenzae T3T1, complete genome	gi|345428590|ref|NC_015964.1| Haemophilus parainfluenzae T3T1, complete genome
contig-100_932	1309	N	0	0	NA	0	0	0	NA	Bacteroides vulgatus ATCC 8482, complete genome	gi|150002608|ref|NC_009614.1| Bacteroides vulgatus ATCC 8482 chromosome, complete genome	gi|150002608|ref|NC_009614.1| Bacteroides vulgatus ATCC 8482 chromosome, complete genome
contig-100_933	1308	N	2	0	NA	0	0	0	NA	NA	NA	NA
contig-100_934	1308	N	1	0	NA	0	1	1	NA	NA	NA	NA
contig-100_935	1307	N	1	0	NA	0	0	0	NA	NA	NA	NA
contig-100_936	1307	N	1	0	NA	0	0	0	NA	NA	NA	NA
contig-100_937	1305	N	2	0	NA	0	0	0	NA	Bacteroides vulgatus ATCC 8482, complete genome	gi|150002608|ref|NC_009614.1| Bacteroides vulgatus ATCC 8482 chromosome, complete genome	gi|150002608|ref|NC_009614.1| Bacteroides vulgatus ATCC 8482 chromosome, complete genome
contig-100_938	1305	N	0	0	NA	0	0	0	NA	NA	NA	NA
contig-100_939	1304	N	1	0	NA	0	0	0	NA	NA	NA	NA
contig-100_94	5497	Y	4	3	Micro	0	0	2	NA	NA	NA	NA
contig-100_940	1303	N	1	0	NA	0	0	0	NA	NA	NA	NA
contig-100_942	1303	N	1	0	NA	0	0	0	NA	Neisseria gonorrhoeae strain GP08-MUS-021 plasmid pEM1, complete sequence	NA	Neisseria gonorrhoeae strain GP08-MUS-021 plasmid pEM1, complete sequence
contig-100_943	1302	N	1	0	NA	0	0	0	NA	NA	NA	NA

contig-100_944	1302	N	1	0	NA	0	0	0	NA	Bacteroides salanitronis DSM 18170, complete genome	gi|325297172|ref|NC_015164.1| Bacteroides salanitronis DSM 18170 chromosome, complete genome	gi|325297172|ref|NC_015164.1| Bacteroides salanitronis DSM 18170 chromosome, complete genome
contig-100_945	1302	N	2	0	NA	0	0	0	NA	NA	NA	NA
contig-100_946	1301	N	0	0	NA	0	0	0	NA	NA	NA	NA
contig-100_948	1300	N	1	0	NA	0	0	0	NA	Bacteroides fragilis plasmid pBFUK1 DNA, complete genome, strain: GAI92082	NA	Bacteroides fragilis plasmid pBFUK1 DNA, complete genome, strain: GAI92082
contig-100_949	1298	N	1	0	NA	0	0	0	NA	Bacteroides vulgatus ATCC 8482, complete genome	gi|150002608|ref|NC_009614.1| Bacteroides vulgatus ATCC 8482 chromosome, complete genome	gi|150002608|ref|NC_009614.1| Bacteroides vulgatus ATCC 8482 chromosome, complete genome
contig-100_95	5465	N	4	0	NA	0	1	1	NA	Uncultured organism clone 1041059765396 genomic sequence	gi|29345410|ref|NC_004663.1| Bacteroides thetaiotaomicron VPI-5482 chromosome, complete genome	gi|29345410|ref|NC_004663.1| Bacteroides thetaiotaomicron VPI-5482 chromosome, complete genome
contig-100_950	1297	N	1	0	NA	0	0	0	NA	NA	NA	NA
contig-100_951	1296	N	1	0	NA	0	0	0	NA	Clostridiales sp. SS3/4 draft genome	gi|479192860|ref|NC_021035.1| Butyrate-producing bacterium SS3/4, complete genome	gi|479192860|ref|NC_021035.1| Butyrate-producing bacterium SS3/4, complete genome
contig-100_952	1295	N	1	0	NA	0	0	0	NA	NA	NA	NA

contig-100_953	1295	N	0	0	NA	0	0	0	NA	Bacteroides thetaiotaomicron VPI-5482, complete genome	gi|29345410|ref|NC_004663.1| Bacteroides thetaiotaomicron VPI-5482 chromosome, complete genome	gi|29345410|ref|NC_004663.1| Bacteroides thetaiotaomicron VPI-5482 chromosome, complete genome
contig-100_955	1289	N	1	0	NA	0	0	0	NA	NA	NA	NA
contig-100_956	1288	N	1	0	NA	0	0	0	NA	Faecalibacterium prausnitzii SL3/3 draft genome	gi|479170689|ref|NC_021020.1| Faecalibacterium prausnitzii SL3/3 draft genome	gi|479170689|ref|NC_021020.1| Faecalibacterium prausnitzii SL3/3 draft genome
contig-100_957	1287	N	2	0	NA	0	0	0	NA	Neisseria gonorrhoeae MS11, complete genome	gi|194097589|ref|NC_011035.1| Neisseria gonorrhoeae NCCP11945 chromosome, complete genome	gi|194097589|ref|NC_011035.1| Neisseria gonorrhoeae NCCP11945 chromosome, complete genome
contig-100_958	1287	N	0	0	NA	0	0	0	NA	Clostridiales sp. SS3/4 draft genome	gi|479192860|ref|NC_021035.1| Butyrate-producing bacterium SS3/4, complete genome	gi|479192860|ref|NC_021035.1| Butyrate-producing bacterium SS3/4, complete genome
contig-100_959	1287	N	1	0	NA	0	0	0	NA	Eubacterium rectale DSM 17629 draft genome	gi|479140210|ref|NC_021010.1| Eubacterium rectale DSM 17629 draft genome	gi|479140210|ref|NC_021010.1| Eubacterium rectale DSM 17629 draft genome
contig-100_960	1286	N	1	0	NA	0	0	0	NA	Odoribacter splanchnicus DSM 20712, complete genome	gi|325278757|ref|NC_015160.1| Odoribacter splanchnicus DSM 20712 chromosome, complete genome	gi|325278757|ref|NC_015160.1| Odoribacter splanchnicus DSM 20712 chromosome, complete genome
contig-100_961	1286	N	0	0	NA	0	0	0	NA	NA	NA	NA
contig-100_962	1285	N	1	0	NA	0	0	0	NA	NA	NA	NA

contig-100_963	1284	N	0	0	NA	0	0	0	NA	NA	NA	NA
contig-100_964	1284	N	2	2	Sipho	2	2	2	NA	NA	NA	NA
contig-100_965	1284	N	1	0	NA	0	0	0	NA	NA	NA	NA
contig-100_967	1282	N	1	0	NA	0	0	0	NA	Klebsiella pneumoniae subsp. pneumoniae 1084, complete genome	gi|550443072|ref|NC_022566.1| Klebsiella pneumoniae CG43, complete genome	gi|550443072|ref|NC_022566.1| Klebsiella pneumoniae CG43, complete genome
contig-100_968	1282	N	1	0	NA	0	0	0	NA	NA	NA	NA
contig-100_969	1281	N	1	1	NA	0	0	1	NA	Haemophilus influenzae F3031 complete genome	gi|319896422|ref|NC_014920.1| Haemophilus influenzae F3031 chromosome, complete genome	gi|319896422|ref|NC_014920.1| Haemophilus influenzae F3031 chromosome, complete genome
contig-100_970	1280	N	1	0	NA	0	0	0	NA	Bacteroides salanitronis DSM 18170, complete genome	gi|325297172|ref|NC_015164.1| Bacteroides salanitronis DSM 18170 chromosome, complete genome	gi|325297172|ref|NC_015164.1| Bacteroides salanitronis DSM 18170 chromosome, complete genome
contig-100_972	1276	N	1	0	NA	0	0	0	NA	Roseburia hominis A2-183, complete genome	gi|347530298|ref|NC_015977.1| Roseburia hominis A2-183 chromosome, complete genome	gi|347530298|ref|NC_015977.1| Roseburia hominis A2-183 chromosome, complete genome
contig-100_973	1276	N	1	0	NA	0	0	0	NA	Bacteroides thetaiotaomicron VPI-5482, complete genome	gi|29345410|ref|NC_004663.1| Bacteroides thetaiotaomicron VPI-5482 chromosome, complete genome	gi|29345410|ref|NC_004663.1| Bacteroides thetaiotaomicron VPI-5482 chromosome, complete genome
contig-100_974	1273	N	1	0	NA	0	0	0	NA	NA	NA	NA
contig-100_978	1271	N	1	0	NA	0	0	0	NA	NA	NA	NA

contig-100_979	1271	N	0	0	NA	0	0	0	NA	Faecalibacterium prausnitzii L2/6 draft genome	gi|479208076|ref|NC_021042.1| Faecalibacterium prausnitzii L2-6, complete genome	gi|479208076|ref|NC_021042.1| Faecalibacterium prausnitzii L2-6, complete genome
contig-100_980	1269	N	0	0	NA	0	0	0	NA	NA	NA	NA
contig-100_981	1269	N	1	0	NA	0	0	0	NA	Candidatus Saccharimonas aalborgensis, complete genome	gi|563711419|ref|NC_023004.1| Candidatus Saccharibacteria bacterium RAAC3_TM7_1, complete genome	gi|563711419|ref|NC_023004.1| Candidatus Saccharibacteria bacterium RAAC3_TM7_1, complete genome
contig-100_982	1268	N	1	0	NA	0	0	0	NA	NA	NA	NA
contig-100_984	1267	N	1	0	NA	0	0	0	NA	NA	NA	NA
contig-100_985	1267	N	0	0	NA	0	0	0	NA	NA	NA	NA
contig-100_987	1266	N	0	0	NA	0	0	0	NA	NA	NA	NA
contig-100_988	1264	N	1	0	NA	0	0	0	NA	NA	NA	NA
contig-100_989	1264	N	2	0	NA	0	0	0	NA	Uncultured organism clone 1041059765627 genomic sequence	NA	Uncultured organism clone 1041059765627 genomic sequence
contig-100_990	1262	N	1	0	NA	0	0	0	NA	NA	NA	NA
contig-100_991	1261	N	2	0	NA	0	0	0	NA	NA	NA	NA
contig-100_992	1261	N	0	0	NA	0	0	0	NA	NA	NA	NA
contig-100_993	1260	N	1	0	NA	0	0	0	NA	NA	NA	NA
contig-100_996	1259	N	2	0	NA	0	0	0	NA	Unidentified phage clone 2019_scaffold132 genomic sequence	NA	Unidentified phage clone 2019_scaffold132 genomic sequence
contig-100_997	1259	N	0	0	NA	0	0	0	NA	Ruminococcus obeum A2-162 draft genome	gi|479176048|ref|NC_021022.1| Ruminococcus obeum A2-162 draft genome	gi|479176048|ref|NC_021022.1| Ruminococcus obeum A2-162 draft genome

contig-100_998	1259	N	1	0	NA	0	0	0	NA	Haemophilus influenzae F3031 complete genome	gi|319896422|ref|NC_014920.1| Haemophilus influenzae F3031 chromosome, complete genome	gi|319896422|ref|NC_014920.1| Haemophilus influenzae F3031 chromosome, complete genome
