# Supplementary material for: Transfer of Viral Communities between Human Individuals during Fecal Microbiota Transplantation
Source: mBio. 2016 Mar 29;7(2):e00322-16. doi: 10.1128/mBio.00322-16 (PMC4817255; doi:10.1128/mBio.00322-16)
Supplement: Table S4 — Patient pre-FMT contigs. Data represent information on contigs of >3,000 bp separately built from the patient 1 pre-FMT sample, patient 2 pre-FMT sample, and patient 3 pre-FMT sample. [file mbo002162747st4.pdf]

Contig Name	Patient (pre-FMT)	Length (bp)	Circular	Number of ORFs	Number of Viral ORFs	Putative Family	Number of Integrase gene matches	Number of Virulence Factor database matches	Number of ACLAME database matches	Top match to NCBI reference viral database	Top match to NCBI nucleotide (nt) database	Top match to NCBI reference bacterial database	Possible host
1	P1	32952	N	20	24	Podo	1	1	17	gi|374531191|ref|NC_016761.1| Salmonella phage SPN1S, complete genome	Escherichia coli 0127:H6 E2348/69 complete genome, strain E2348/69	gi|215485161|ref|NC_011601.1| Escherichia coli O127:H6 str. E2348/69 chromosome, complete genome	gi|374531191|ref|NC_016761.1| Salmonella phage SPN1S, complete genome
2	P1	13964	N	14	19	Sipho	2	0	5	NA	Oscillibacter valericigenes Sjm18-20 DNA, complete genome	gi|350268398|ref|NC_016048.1| Oscillibacter valericigenes Sjm18-20, complete genome	gi|350268398|ref|NC_016048.1| Oscillibacter valericigenes Sjm18-20, complete genome
3	P1	14681	N	9	7	Sipho	0	0	0	NA	Bacteroides thetaiotaomicron VPI-5482, complete genome	gi|29345410|ref|NC_004663.1| Bacteroides thetaiotaomicron VPI-5482 chromosome, complete genome	gi|29345410|ref|NC_004663.1| Bacteroides thetaiotaomicron VPI-5482 chromosome, complete genome
4	P1	9530	N	7	9	Sipho	0	0	1	NA	NA	NA	NA
5	P1	7314	N	5	10	Sipho	0	0	2	NA	NA	NA	NA
6	P1	8170	N	4	15	Sipho	0	0	3	NA	NA	NA	NA
7	P1	6692	N	6	11	Sipho	0	0	0	NA	NA	NA	NA
8	P1	22241	N	13	18	Sipho	0	0	4	NA	Bacteroides thetaiotaomicron VPI-5482, complete genome	gi|29345410|ref|NC_004663.1| Bacteroides thetaiotaomicron VPI-5482 chromosome, complete genome	gi|29345410|ref|NC_004663.1| Bacteroides thetaiotaomicron VPI-5482 chromosome, complete genome

9	P1	11312	N	6	5	Sipho	0	0	0	NA	Uncultured bacterium clone HA0AAA20ZG01FM1 genomic sequence	NA	Uncultured bacterium clone HA0AAA20ZG01FM1 genomic sequence
10	P1	12727	N	6	10	Sipho	0	0	0	NA	NA	NA	NA
11	P1	8673	N	2	4	Sipho	0	0	0	NA	NA	NA	NA
12	P1	6356	N	4	1	Sipho	0	0	0	NA	NA	NA	NA
13	P1	4729	N	3	2	Micro	0	0	0	NA	NA	NA	NA
14	P1	4884	Y	3	7	Sipho	0	0	0	NA	Bacteroides fragilis plasmid pBFUK1 DNA, complete genome, strain: GAI92082	NA	Bacteroides fragilis plasmid pBFUK1 DNA, complete genome, strain: GAI92082
15	P1	4850	N	2	5	Sipho	0	0	0	NA	Uncultured bacterium clone LM0ACA14ZD05RM1 genomic sequence	gi|479162165|ref|NC_021017.1| Bacteroides xylanisolvens XB1A draft genome	gi|479162165|ref|NC_021017.1| Bacteroides xylanisolvens XB1A draft genome
16	P1	4472	N	4	4	Sipho	0	0	1	NA	NA	NA	NA
17	P1	3967	N	3	2	Podo	0	0	0	NA	NA	NA	NA
18	P1	11401	N	6	10	Sipho	0	0	3	gi|358356482|ref|NC_016163.1| Yersinia phage phiR1-37, complete genome	NA	NA	gi|358356482|ref|NC_016163.1| Yersinia phage phiR1-37, complete genome
19	P1	5959	N	2	2	Myo	0	0	0	NA	NA	NA	NA
20	P1	5285	N	4	4	Myo	0	0	1	NA	NA	NA	NA
21	P1	3990	N	3	2	Podo	0	0	0	NA	NA	NA	NA
23	P1	5405	N	4	9	Sipho	0	0	2	NA	NA	NA	NA
24	P1	3038	N	2	6	Sipho	0	0	0	NA	Klebsiella oxytoca E718, complete genome	gi|397655102|ref|NC_018106.1| Klebsiella oxytoca E718 chromosome, complete genome	gi|397655102|ref|NC_018106.1| Klebsiella oxytoca E718 chromosome, complete genome
contig-100_1	P1	15940	N	3	10	Sipho	0	0	0	NA	NA	NA	NA
contig-100_10	P1	5824	N	3	2	Micro	0	0	2	NA	NA	NA	NA
contig-100_11	P1	5744	Y	5	3	Micro	0	0	3	NA	NA	NA	NA
contig-100_15	P1	5597	N	2	5	Sipho	0	0	0	NA	NA	NA	NA
contig-100_16	P1	5189	N	6	4	Sipho	0	0	4	NA	NA	NA	NA

contig-100_18	P1	4892	N	3	8	Sipho	0	0	1	NA	NA	NA	NA
contig-100_20	P1	4498	N	3	6	Sipho	0	0	1	NA	NA	NA	NA
contig-100_21	P1	4382	N	4	6	Podo	1	1	2	NA	Klebsiella pneumoniae 342, complete genome	gi|206575712|ref|NC_011283.1| Klebsiella pneumoniae 342 chromosome, complete genome	gi|206575712|ref|NC_011283.1| Klebsiella pneumoniae 342 chromosome, complete genome
contig-100_25	P1	3789	Y	2	0	NA	0	0	0	NA	NA	NA	NA
contig-100_26	P1	3727	N	4	1	Sipho	0	0	1	NA	NA	NA	NA
contig-100_28	P1	3513	N	3	2	Myo	0	0	2	NA	NA	NA	NA
contig-100_29	P1	3425	N	1	0	NA	0	0	0	NA	Klebsiella pneumoniae JM45, complete genome	gi|530627845|ref|NC_022082.1| Klebsiella pneumoniae JM45, complete genome	gi|530627845|ref|NC_022082.1| Klebsiella pneumoniae JM45, complete genome
contig-100_33	P1	3116	N	1	0	NA	0	0	0	NA	NA	NA	NA
contig-100_4	P1	9093	N	6	6	Podo	2	1	6	gi|374531191|ref|NC_016761.1| Salmonella phage SPN1S, complete genome	Escherichia coli 0127:H6 E2348/69 complete genome, strain E2348/69	gi|215485161|ref|NC_011601.1| Escherichia coli O127:H6 str. E2348/69 chromosome, complete genome	gi|374531191|ref|NC_016761.1| Salmonella phage SPN1S, complete genome
contig-100_9	P1	6071	Y	4	3	Micro	0	0	3	NA	NA	NA	NA
1	P2	34676	N	24	25	Podo	0	11	8	NA	Klebsiella pneumoniae subsp. pneumoniae HS11286, complete genome	gi|378976159|ref|NC_016845.1| Klebsiella pneumoniae subsp. pneumoniae HS11286 chromosome, complete genome	gi|378976159|ref|NC_016845.1| Klebsiella pneumoniae subsp. pneumoniae HS11286 chromosome, complete genome
2	P2	30940	N	16	27	Sipho	6	0	11	gi|77999988|ref|NC_007501.1| Lactobacillus phage Lc-Nu, complete genome	Lactobacillus rhamnosus ATCC 53103 DNA, complete genome	gi|258506995|ref|NC_013198.1| Lactobacillus rhamnosus GG chromosome, complete genome	gi|77999988|ref|NC_007501.1| Lactobacillus phage Lc-Nu, complete genome

3	P2	22239	N	12	14	Sipho	0	0	9	gi|9634119|ref|NC_002166.1| Enterobacteria phage HK022, complete genome	Enterobacteria phage mEp235, complete genome	gi|525855729|ref|NC_021818.1| Salmonella enterica subsp. enterica Serovar Cubana str. CFSAN002050, complete genome	gi|9634119|ref|NC_002166.1| Enterobacteria phage HK022, complete genome
4	P2	24439	N	15	12	Sipho	0	2	4	gi|169257267|ref|NC_010393.1| Phage Gifsy-2, complete genome	Salmonella bongori N268-08, complete genome	gi|526225953|ref|NC_021870.1| Salmonella bongori N268-08, complete genome	gi|169257267|ref|NC_010393.1| Phage Gifsy-2, complete genome
5	P2	31203	N	20	11	Sipho	0	10	2	NA	Citrobacter koseri ATCC BAA-895, complete genome	gi|157144296|ref|NC_009792.1| Citrobacter koseri ATCC BAA-895 chromosome, complete genome	gi|157144296|ref|NC_009792.1| Citrobacter koseri ATCC BAA-895 chromosome, complete genome
6	P2	20116	N	19	15	Sipho	0	0	0	NA	Uncultured organism clone 1041059765920 genomic sequence	NA	Uncultured organism clone 1041059765920 genomic sequence
7	P2	15451	N	8	17	Sipho	1	0	7	gi|374531191|ref|NC_016761.1| Salmonella phage SPN1S, complete genome	Enterobacter sp. 638, complete genome	gi|146309667|ref|NC_009436.1| Enterobacter sp. 638, complete genome	gi|374531191|ref|NC_016761.1| Salmonella phage SPN1S, complete genome
8	P2	13044	N	8	18	Sipho	0	0	0	NA	Uncultured organism clone 1041059767004 genomic sequence	NA	Uncultured organism clone 1041059767004 genomic sequence
9	P2	11590	N	4	8	Sipho	0	0	2	gi|356870600|ref|NC_016158.1| Escherichia phage HK639, complete genome	Klebsiella oxytoca E718, complete genome	gi|397655102|ref|NC_018106.1| Klebsiella oxytoca E718 chromosome, complete genome	gi|356870600|ref|NC_016158.1| Escherichia phage HK639, complete genome

<table>
<tr><td></td><td></td><td></td><td></td><td></td><td></td><td></td><td></td><td></td><td></td><td></td><td></td><td></td><td></td></tr>
<tr><td>10</td><td>P2</td><td>19022</td><td>N</td><td>18</td><td>14</td><td>Sipho</td><td>0</td><td>3</td><td>3</td><td>NA</td><td>Salmonella enterica subsp. enterica serovar Thompson str. RM6836, complete genome</td><td>gi|549478129|ref|NC_022525.1| Salmonella enterica subsp. enterica serovar Thompson str. RM6836, complete genome</td><td>gi|549478129|ref|NC_022525.1| Salmonella enterica subsp. enterica serovar Thompson str. RM6836, complete genome</td></tr>
<tr><td>11</td><td>P2</td><td>12385</td><td>N</td><td>7</td><td>4</td><td>Sipho</td><td>0</td><td>0</td><td>1</td><td>NA</td><td>Citrobacter koseri ATCC BAA-895, complete genome</td><td>gi|157144296|ref|NC_009792.1| Citrobacter koseri ATCC BAA-895 chromosome, complete genome</td><td>gi|157144296|ref|NC_009792.1| Citrobacter koseri ATCC BAA-895 chromosome, complete genome</td></tr>
<tr><td>12</td><td>P2</td><td>12307</td><td>N</td><td>9</td><td>1</td><td>Sipho</td><td>0</td><td>3</td><td>1</td><td>NA</td><td>Escherichia coli ABU 83972, complete genome</td><td>gi|386637352|ref|NC_017631.1| Escherichia coli ABU 83972 chromosome, complete genome</td><td>gi|386637352|ref|NC_017631.1| Escherichia coli ABU 83972 chromosome, complete genome</td></tr>
<tr><td>13</td><td>P2</td><td>19616</td><td>N</td><td>11</td><td>3</td><td>Micro</td><td>0</td><td>2</td><td>1</td><td>NA</td><td>Klebsiella pneumoniae subsp. pneumoniae HS11286, complete genome</td><td>gi|378976159|ref|NC_016845.1| Klebsiella pneumoniae subsp. pneumoniae HS11286 chromosome, complete genome</td><td>gi|378976159|ref|NC_016845.1| Klebsiella pneumoniae subsp. pneumoniae HS11286 chromosome, complete genome</td></tr>
<tr><td>14</td><td>P2</td><td>10493</td><td>N</td><td>4</td><td>11</td><td>Sipho</td><td>1</td><td>0</td><td>4</td><td>gi|45597386|ref|NC_005822.1| Lactococcus phage phiLC3, complete genome</td><td>Lactococcus lactis subsp. lactis KF147, complete genome</td><td>gi|281490498|ref|NC_013656.1| Lactococcus lactis subsp. lactis KF147 chromosome, complete genome</td><td>gi|45597386|ref|NC_005822.1| Lactococcus phage phiLC3, complete genome</td></tr>
</table>

15	P2	8016	N	3	8	Sipho	1	1	3	gi|155370093|ref|NC_007804.2| Escherichia phage phiV10, complete genome	Escherichia coli O7:K1 str. CE10, complete genome	gi|386622414|ref|NC_017646.1| Escherichia coli O7:K1 str. CE10 chromosome, complete genome	gi|155370093|ref|NC_007804.2| Escherichia phage phiV10, complete genome
16	P2	9492	N	5	7	Sipho	0	0	4	gi|356870600|ref|NC_016158.1| Escherichia phage HK639, complete genome	Klebsiella pneumoniae subsp. pneumoniae MGH 78578, complete sequence	gi|152968582|ref|NC_009648.1| Klebsiella pneumoniae subsp. pneumoniae MGH 78578 chromosome, complete genome	gi|356870600|ref|NC_016158.1| Escherichia phage HK639, complete genome
17	P2	11076	N	8	3	Podo	0	3	2	NA	Klebsiella pneumoniae subsp. pneumoniae HS11286, complete genome	gi|378976159|ref|NC_016845.1| Klebsiella pneumoniae subsp. pneumoniae HS11286 chromosome, complete genome	gi|378976159|ref|NC_016845.1| Klebsiella pneumoniae subsp. pneumoniae HS11286 chromosome, complete genome
18	P2	10752	N	14	11	Sipho	0	0	1	NA	Uncultured organism clone 1041059766118 genomic sequence	NA	Uncultured organism clone 1041059766118 genomic sequence
19	P2	7891	N	5	3	Myo	0	0	1	NA	Escherichia coli NA114, complete genome	gi|386617516|ref|NC_017644.1| Escherichia coli NA114 chromosome, complete genome	gi|386617516|ref|NC_017644.1| Escherichia coli NA114 chromosome, complete genome
20	P2	8815	N	7	5	Myo	0	1	2	NA	Escherichia coli O83:H1 str. NRG 857C, complete genome	gi|387615344|ref|NC_017634.1| Escherichia coli O83:H1 str. NRG 857C chromosome, complete genome	gi|387615344|ref|NC_017634.1| Escherichia coli O83:H1 str. NRG 857C chromosome, complete genome

21	P2	10316	N	9	3	Podo	0	0	0	NA	Uncultured organism clone 1041059764512 genomic sequence	NA	Uncultured organism clone 1041059764512 genomic sequence
22	P2	8327	N	7	4	Sipho	0	1	2	NA	Citrobacter koseri ATCC BAA-895, complete genome	gi|157144296|ref|NC_009792.1| Citrobacter koseri ATCC BAA-895 chromosome, complete genome	gi|157144296|ref|NC_009792.1| Citrobacter koseri ATCC BAA-895 chromosome, complete genome
23	P2	13269	N	13	10	Sipho	0	3	2	NA	Escherichia coli str. 'clone D i14', complete genome	gi|386632422|ref|NC_017652.1| Escherichia coli str. 'clone D i14' chromosome, complete genome	gi|386632422|ref|NC_017652.1| Escherichia coli str. 'clone D i14' chromosome, complete genome
24	P2	7147	N	3	6	Sipho	0	2	1	gi|41057278|ref|NC_005344.1| Enterobacteria phage Sf6, complete genome	Escherichia coli ABU 83972, complete genome	gi|386637352|ref|NC_017631.1| Escherichia coli ABU 83972 chromosome, complete genome	gi|41057278|ref|NC_005344.1| Enterobacteria phage Sf6, complete genome
25	P2	16252	N	8	11	Sipho	0	0	5	gi|155370093|ref|NC_007804.2| Escherichia phage phiV10, complete genome	Escherichia coli IHE3034, complete genome	gi|386597751|ref|NC_017628.1| Escherichia coli IHE3034 chromosome, complete genome	gi|155370093|ref|NC_007804.2| Escherichia phage phiV10, complete genome
26	P2	8737	N	7	5	Sipho	0	1	2	NA	Escherichia coli O83:H1 str. NRG 857C, complete genome	gi|387615344|ref|NC_017634.1| Escherichia coli O83:H1 str. NRG 857C chromosome, complete genome	gi|387615344|ref|NC_017634.1| Escherichia coli O83:H1 str. NRG 857C chromosome, complete genome

27	P2	7389	N	4	1	Herpes	0	1	0	NA	Escherichia coli str. 'clone D i14', complete genome	gi|386627502|ref|NC_017651.1| Escherichia coli str. 'clone D i2' chromosome, complete genome	gi|386627502|ref|NC_017651.1| Escherichia coli str. 'clone D i2' chromosome, complete genome
28	P2	7835	N	6	3	Sipho	0	1	2	NA	Citrobacter koseri ATCC BAA-895, complete genome	gi|157144296|ref|NC_009792.1| Citrobacter koseri ATCC BAA-895 chromosome, complete genome	gi|157144296|ref|NC_009792.1| Citrobacter koseri ATCC BAA-895 chromosome, complete genome
29	P2	10398	N	6	6	Sipho	1	0	5	NA	NA	NA	NA
30	P2	7044	N	5	1	Sipho	0	1	0	NA	Salmonella bongori N268-08, complete genome	gi|526225953|ref|NC_021870.1| Salmonella bongori N268-08, complete genome	gi|526225953|ref|NC_021870.1| Salmonella bongori N268-08, complete genome
31	P2	6003	N	4	8	Sipho	0	0	0	NA	NA	NA	NA
32	P2	7518	N	5	3	Sipho	0	0	0	NA	Citrobacter koseri ATCC BAA-895, complete genome	gi|157144296|ref|NC_009792.1| Citrobacter koseri ATCC BAA-895 chromosome, complete genome	gi|157144296|ref|NC_009792.1| Citrobacter koseri ATCC BAA-895 chromosome, complete genome
33	P2	5026	N	5	4	Sipho	0	0	2	NA	Klebsiella pneumoniae JM45, complete genome	gi|530627845|ref|NC_022082.1| Klebsiella pneumoniae JM45, complete genome	gi|530627845|ref|NC_022082.1| Klebsiella pneumoniae JM45, complete genome
34	P2	9480	N	4	5	Sipho	0	0	3	NA	Enterococcus faecium Aus0085, complete genome	gi|529232713|ref|NC_021994.1| Enterococcus faecium Aus0085, complete genome	gi|529232713|ref|NC_021994.1| Enterococcus faecium Aus0085, complete genome

35	P2	6175	N	3	2	Sipho	0	0	1	NA	Klebsiella pneumoniae 342, complete genome	gi|206575712|ref|NC_011283.1| Klebsiella pneumoniae 342 chromosome, complete genome	gi|206575712|ref|NC_011283.1| Klebsiella pneumoniae 342 chromosome, complete genome
36	P2	20743	N	13	8	Sipho	1	0	3	NA	NA	NA	NA
37	P2	21762	N	17	11	Sipho	0	2	4	NA	Klebsiella pneumoniae KCTC 2242, complete genome	gi|386032579|ref|NC_017540.1| Klebsiella pneumoniae KCTC 2242 chromosome, complete genome	gi|386032579|ref|NC_017540.1| Klebsiella pneumoniae KCTC 2242 chromosome, complete genome
38	P2	4842	N	4	3	Myo	0	0	0	NA	Salmonella enterica subsp. enterica serovar Schwarzengrund str. CVM19633, complete genome	gi|194733902|ref|NC_011094.1| Salmonella enterica subsp. enterica serovar Schwarzengrund str. CVM19633 chromosome, complete genome	gi|194733902|ref|NC_011094.1| Salmonella enterica subsp. enterica serovar Schwarzengrund str. CVM19633 chromosome, complete genome
39	P2	4775	N	6	2	Sipho	0	0	0	NA	Uncultured organism clone 1041059767192 genomic sequence	NA	Uncultured organism clone 1041059767192 genomic sequence
40	P2	5107	N	4	3	Sipho	0	0	1	NA	Enterococcus faecium Aus0085, complete genome	gi|529232713|ref|NC_021994.1| Enterococcus faecium Aus0085, complete genome	gi|529232713|ref|NC_021994.1| Enterococcus faecium Aus0085, complete genome
41	P2	7423	N	3	3	Sipho	2	0	3	gi|13786531|ref|NC_002747.1| Lactococcus phage TP901-1, complete genome	Lactococcus lactis subsp. cremoris A76, complete genome	gi|385836969|ref|NC_017492.1| Lactococcus lactis subsp. cremoris A76 chromosome, complete genome	gi|13786531|ref|NC_002747.1| Lactococcus phage TP901-1, complete genome

42	P2	3918	N	5	6	Sipho	0	0	1	NA	Escherichia coli str. 'clone D i14', complete genome	gi|386627502|ref|NC_017651.1| Escherichia coli str. 'clone D i2' chromosome, complete genome	gi|386627502|ref|NC_017651.1| Escherichia coli str. 'clone D i2' chromosome, complete genome
43	P2	5630	N	2	5	Sipho	0	0	1	gi|46402086|ref|NC_005857.1| Klebsiella phage phiKO2, complete genome	Klebsiella oxytoca E718, complete genome	gi|397655102|ref|NC_018106.1| Klebsiella oxytoca E718 chromosome, complete genome	gi|46402086|ref|NC_005857.1| Klebsiella phage phiKO2, complete genome
44	P2	4254	N	2	7	Sipho	0	0	1	NA	Uncultured bacterium clone HA0AAA2ZB09FM1 genomic sequence	NA	Uncultured bacterium clone HA0AAA2ZB09FM1 genomic sequence
45	P2	5268	N	3	2	Sipho	0	0	1	NA	Klebsiella pneumoniae JM45, complete genome	gi|530627845|ref|NC_022082.1| Klebsiella pneumoniae JM45, complete genome	gi|530627845|ref|NC_022082.1| Klebsiella pneumoniae JM45, complete genome
46	P2	6648	N	4	9	Sipho	0	1	2	NA	Klebsiella pneumoniae subsp. pneumoniae MGH 78578, complete sequence	gi|550443072|ref|NC_022566.1| Klebsiella pneumoniae CG43, complete genome	gi|550443072|ref|NC_022566.1| Klebsiella pneumoniae CG43, complete genome
47	P2	3692	N	1	3	Sipho	0	0	0	NA	Klebsiella pneumoniae JM45, complete genome	gi|530627845|ref|NC_022082.1| Klebsiella pneumoniae JM45, complete genome	gi|530627845|ref|NC_022082.1| Klebsiella pneumoniae JM45, complete genome
48	P2	8153	N	7	4	Myo	0	1	0	NA	Klebsiella pneumoniae KCTC 2242, complete genome	gi|386032579|ref|NC_017540.1| Klebsiella pneumoniae KCTC 2242 chromosome, complete genome	gi|386032579|ref|NC_017540.1| Klebsiella pneumoniae KCTC 2242 chromosome, complete genome

49	P2	6089	N	7	3	Sipho	0	0	1	NA	Klebsiella pneumoniae KCTC 2242, complete genome	gi|386032579|ref|NC_017540.1| Klebsiella pneumoniae KCTC 2242 chromosome, complete genome	gi|386032579|ref|NC_017540.1| Klebsiella pneumoniae KCTC 2242 chromosome, complete genome
50	P2	6593	N	2	0	NA	0	0	1	NA	Citrobacter rodentium ICC168, complete genome	gi|283783779|ref|NC_013716.1| Citrobacter rodentium ICC168 chromosome, complete genome	gi|283783779|ref|NC_013716.1| Citrobacter rodentium ICC168 chromosome, complete genome
51	P2	4503	N	2	5	Sipho	0	1	1	NA	Escherichia coli PMV-1 main chromosome, complete genome	gi|91209055|ref|NC_007946.1| Escherichia coli UTI89 chromosome, complete genome	gi|91209055|ref|NC_007946.1| Escherichia coli UTI89 chromosome, complete genome
52	P2	5443	N	1	1	NA	0	0	0	NA	Uncultured bacterium clone LM0ACA14ZD05RM1 genomic sequence	NA	Uncultured bacterium clone LM0ACA14ZD05RM1 genomic sequence
53	P2	4700	N	2	0	NA	0	2	0	NA	Citrobacter freundii fimbrial operon	gi|345297184|ref|NC_015968.1| Enterobacter asburiae LF7a chromosome, complete genome	gi|345297184|ref|NC_015968.1| Enterobacter asburiae LF7a chromosome, complete genome
54	P2	8179	N	6	2	Myo	0	1	2	NA	Citrobacter koseri ATCC BAA-895, complete genome	gi|157144296|ref|NC_009792.1| Citrobacter koseri ATCC BAA-895 chromosome, complete genome	gi|157144296|ref|NC_009792.1| Citrobacter koseri ATCC BAA-895 chromosome, complete genome

55	P2	4212	N	3	6	Sipho	0	0	3	gi|45597386|ref|NC_005822.1| Lactococcus phage phiLC3, complete genome	Bacteriophage phi LC3, complete genome	gi|281490498|ref|NC_013656.1| Lactococcus lactis subsp. lactis KF147 chromosome, complete genome	gi|45597386|ref|NC_005822.1| Lactococcus phage phiLC3, complete genome
56	P2	4950	N	9	1	Myo	0	0	0	NA	Uncultured organism clone 1041059766118 genomic sequence	NA	Uncultured organism clone 1041059766118 genomic sequence
57	P2	3115	N	2	4	Sipho	0	0	1	gi|169257267|ref|NC_010393.1| Phage Gifsy-2, complete genome	Klebsiella pneumoniae 342, complete genome	gi|206575712|ref|NC_011283.1| Klebsiella pneumoniae 342 chromosome, complete genome	gi|169257267|ref|NC_010393.1| Phage Gifsy-2, complete genome
59	P2	6535	N	4	3	Sipho	0	0	1	NA	NA	NA	NA
60	P2	3415	N	2	2	Sipho	0	0	0	NA	Escherichia coli ABU 83972, complete genome	gi|386637352|ref|NC_017631.1| Escherichia coli ABU 83972 chromosome, complete genome	gi|386637352|ref|NC_017631.1| Escherichia coli ABU 83972 chromosome, complete genome
61	P2	3688	N	2	4	Sipho	0	1	0	NA	Citrobacter koseri ATCC BAA-895, complete genome	gi|157144296|ref|NC_009792.1| Citrobacter koseri ATCC BAA-895 chromosome, complete genome	gi|157144296|ref|NC_009792.1| Citrobacter koseri ATCC BAA-895 chromosome, complete genome
62	P2	5839	N	4	6	Sipho	1	0	3	NA	Enterococcus faecium Aus0004, complete genome	gi|383327320|ref|NC_017022.1| Enterococcus faecium Aus0004 chromosome, complete genome	gi|383327320|ref|NC_017022.1| Enterococcus faecium Aus0004 chromosome, complete genome

63	P2	4025	N	3	0	NA	0	0	0	NA	Citrobacter koseri ATCC BAA-895, complete genome	gi|157144296|ref|NC_009792.1| Citrobacter koseri ATCC BAA-895 chromosome, complete genome	gi|157144296|ref|NC_009792.1| Citrobacter koseri ATCC BAA-895 chromosome, complete genome
64	P2	3595	N	2	2	Sipho	0	0	0	NA	Klebsiella pneumoniae subsp. rhinoscleromatis strain SB3432, complete genome	gi|529985600|ref|NC_021232.1| Klebsiella pneumoniae subsp. rhinoscleromatis strain SB3432, complete genome	gi|529985600|ref|NC_021232.1| Klebsiella pneumoniae subsp. rhinoscleromatis strain SB3432, complete genome
65	P2	3393	N	6	1	Sipho	0	0	0	NA	Uncultured organism clone 1041059764512 genomic sequence	NA	Uncultured organism clone 1041059764512 genomic sequence
68	P2	6780	N	4	1	Sipho	0	1	1	NA	Escherichia coli ABU 83972, complete genome	gi|386637352|ref|NC_017631.1| Escherichia coli ABU 83972 chromosome, complete genome	gi|386637352|ref|NC_017631.1| Escherichia coli ABU 83972 chromosome, complete genome
69	P2	5641	N	3	1	NA	0	0	1	NA	Klebsiella pneumoniae JM45, complete genome	gi|530627845|ref|NC_022082.1| Klebsiella pneumoniae JM45, complete genome	gi|530627845|ref|NC_022082.1| Klebsiella pneumoniae JM45, complete genome
70	P2	4939	N	2	2	Sipho	1	0	1	NA	NA	NA	NA
71	P2	4038	N	3	0	NA	0	0	0	gi|46402086|ref|NC_005857.1| Klebsiella phage phiKO2, complete genome	Klebsiella oxytoca KCTC 1686, complete genome	gi|375256816|ref|NC_016612.1| Klebsiella oxytoca KCTC 1686 chromosome, complete genome	gi|46402086|ref|NC_005857.1| Klebsiella phage phiKO2, complete genome

73	P2	4073	N	2	1	Sipho	1	0	1	gi|195661200|ref|NC_011104.1| Lactobacillus phage Lrm1, complete genome	Lactobacillus rhamnosus LOCK900, complete genome	gi|523515595|ref|NC_021723.1| Lactobacillus rhamnosus LOCK900, complete genome	gi|195661200|ref|NC_011104.1| Lactobacillus phage Lrm1, complete genome
74	P2	3490	N	2	0	NA	0	0	0	NA	Klebsiella pneumoniae 342, complete genome	gi|206575712|ref|NC_011283.1| Klebsiella pneumoniae 342 chromosome, complete genome	gi|206575712|ref|NC_011283.1| Klebsiella pneumoniae 342 chromosome, complete genome
76	P2	5997	N	5	2	Myo	0	1	1	NA	Klebsiella pneumoniae subsp. pneumoniae NTUH-K2044 DNA, complete genome	gi|238892256|ref|NC_012731.1| Klebsiella pneumoniae NTUH-K2044 chromosome, complete genome	gi|238892256|ref|NC_012731.1| Klebsiella pneumoniae NTUH-K2044 chromosome, complete genome
77	P2	5088	N	3	0	NA	0	0	0	NA	Klebsiella pneumoniae subsp. pneumoniae MGH 78578, complete sequence	gi|152968582|ref|NC_009648.1| Klebsiella pneumoniae subsp. pneumoniae MGH 78578 chromosome, complete genome	gi|152968582|ref|NC_009648.1| Klebsiella pneumoniae subsp. pneumoniae MGH 78578 chromosome, complete genome
78	P2	3443	N	3	2	Sipho	1	0	1	NA	Enterobacter cloacae subsp. cloacae NCTC 9394 draft genome	gi|479270911|ref|NC_021046.1| Enterobacter cloacae subsp. cloacae NCTC 9394 draft genome	gi|479270911|ref|NC_021046.1| Enterobacter cloacae subsp. cloacae NCTC 9394 draft genome
81	P2	4472	N	3	1	Sipho	0	0	0	NA	Salmonella enterica subsp. enterica serovar Agona str. SL483, complete genome	gi|197247352|ref|NC_011149.1| Salmonella enterica subsp. enterica serovar Agona str. SL483 chromosome, complete genome	gi|197247352|ref|NC_011149.1| Salmonella enterica subsp. enterica serovar Agona str. SL483 chromosome, complete genome

82	P2	3097	N	3	0	NA	0	0	0	NA	Bacteroides fragilis strain SWE94 carbapenem-resistance protein (cfiA) gene, partial cds; and insertion sequence IS614B Tp614 gene, complete cds	gi|302344773|ref|NC_014370.1| Prevotella melaninogenica ATCC 25845 chromosome I, complete sequence	gi|302344773|ref|NC_014370.1| Prevotella melaninogenica ATCC 25845 chromosome I, complete sequence
86	P2	3202	N	3	1	Sipho	0	0	1	NA	Klebsiella pneumoniae KCTC 2242, complete genome	gi|386032579|ref|NC_017540.1| Klebsiella pneumoniae KCTC 2242 chromosome, complete genome	gi|386032579|ref|NC_017540.1| Klebsiella pneumoniae KCTC 2242 chromosome, complete genome
91	P2	3531	N	2	1	Sipho	0	0	0	NA	Escherichia coli O83:H1 str. NRG 857C, complete genome	gi|387615344|ref|NC_017634.1| Escherichia coli O83:H1 str. NRG 857C chromosome, complete genome	gi|387615344|ref|NC_017634.1| Escherichia coli O83:H1 str. NRG 857C chromosome, complete genome
93	P2	3885	N	4	2	Myo	0	0	0	NA	Klebsiella pneumoniae subsp. pneumoniae HS11286, complete genome	gi|378976159|ref|NC_016845.1| Klebsiella pneumoniae subsp. pneumoniae HS11286 chromosome, complete genome	gi|378976159|ref|NC_016845.1| Klebsiella pneumoniae subsp. pneumoniae HS11286 chromosome, complete genome
95	P2	3675	N	3	1	NA	0	1	1	NA	Escherichia coli NA114, complete genome	gi|386617516|ref|NC_017644.1| Escherichia coli NA114 chromosome, complete genome	gi|386617516|ref|NC_017644.1| Escherichia coli NA114 chromosome, complete genome

contig-100_0	P2	41942	N	26	3	Myo	0	2	8	NA	Citrobacter koseri ATCC BAA-895, complete genome	gi|157144296|ref|NC_009792.1| Citrobacter koseri ATCC BAA-895 chromosome, complete genome	gi|157144296|ref|NC_009792.1| Citrobacter koseri ATCC BAA-895 chromosome, complete genome
contig-100_10	P2	15042	N	10	6	Sipho	1	0	6	gi|23505445|ref|NC_004313.1| Salmonella phage ST64B, complete genome	pneumoniae replication protein P (kc01) gene, partial cds; putative DNA binding protein (kc02), hypothetical protein (kc03), hypothetical protein (kc04), hypothetical protein (kc05), hypothetical protein (kc06), hypothetical protein (kc07), hypothetical protein (kc08), hypothetical protein (kc09), klebicin C phage associated protein (kcp), klebicin C activity (kca), and klebicin C immunity (kci) genes, complete cds; and antiterminator	gi|550443072|ref|NC_022566.1| Klebsiella pneumoniae CG43, complete genome	gi|23505445|ref|NC_004313.1| Salmonella phage ST64B, complete genome
contig-100_102	P2	3571	N	3	2	NA	0	2	3	NA	Escherichia coli ED1a chromosome, complete genome	gi|218687878|ref|NC_011745.1| Escherichia coli ED1a chromosome, complete genome	gi|218687878|ref|NC_011745.1| Escherichia coli ED1a chromosome, complete genome

contig-100_106	P2	3455	N	4	0	NA	0	1	2	NA	Citrobacter koseri ATCC BAA-895, complete genome	gi|157144296|ref|NC_009792.1| Citrobacter koseri ATCC BAA-895 chromosome, complete genome	gi|157144296|ref|NC_009792.1| Citrobacter koseri ATCC BAA-895 chromosome, complete genome
contig-100_107	P2	3455	N	3	0	NA	0	0	0	NA	Escherichia coli ED1a chromosome, complete genome	gi|218687878|ref|NC_011745.1| Escherichia coli ED1a chromosome, complete genome	gi|218687878|ref|NC_011745.1| Escherichia coli ED1a chromosome, complete genome
contig-100_108	P2	3398	N	1	0	NA	0	0	0	NA	Klebsiella pneumoniae JM45, complete genome	gi|530627845|ref|NC_022082.1| Klebsiella pneumoniae JM45, complete genome	gi|530627845|ref|NC_022082.1| Klebsiella pneumoniae JM45, complete genome
contig-100_11	P2	14747	N	9	9	Myo	1	0	7	gi|23505445|ref|NC_004313.1| Salmonella phage ST64B, complete genome	Citrobacter rodentium ICC168, complete genome	gi|283783779|ref|NC_013716.1| Citrobacter rodentium ICC168 chromosome, complete genome	gi|23505445|ref|NC_004313.1| Salmonella phage ST64B, complete genome
contig-100_110	P2	3315	N	4	2	Sipho	0	0	2	gi|155370093|ref|NC_007804.2| Escherichia phage phiV10, complete genome	Enterobacteria phage phiV10, complete genome	gi|386612163|ref|NC_017641.1| Escherichia coli UMNK88 chromosome, complete genome	gi|155370093|ref|NC_007804.2| Escherichia phage phiV10, complete genome
contig-100_111	P2	3307	N	2	3	Micro	0	0	1	NA	Klebsiella pneumoniae KCTC 2242, complete genome	gi|386032579|ref|NC_017540.1| Klebsiella pneumoniae KCTC 2242 chromosome, complete genome	gi|386032579|ref|NC_017540.1| Klebsiella pneumoniae KCTC 2242 chromosome, complete genome

contig-100_112	P2	3265	N	1	1	Sipho	0	0	1	NA	Klebsiella pneumoniae JM45, complete genome	gi|530627845|ref|NC_022082.1| Klebsiella pneumoniae JM45, complete genome	gi|530627845|ref|NC_022082.1| Klebsiella pneumoniae JM45, complete genome
contig-100_114	P2	3214	N	3	1	Sipho	0	0	1	NA	Citrobacter rodentium ICC168, complete genome	gi|283783779|ref|NC_013716.1| Citrobacter rodentium ICC168 chromosome, complete genome	gi|283783779|ref|NC_013716.1| Citrobacter rodentium ICC168 chromosome, complete genome
contig-100_115	P2	3176	N	2	0	NA	0	0	0	NA	Citrobacter koseri ATCC BAA-895, complete genome	gi|157144296|ref|NC_009792.1| Citrobacter koseri ATCC BAA-895 chromosome, complete genome	gi|157144296|ref|NC_009792.1| Citrobacter koseri ATCC BAA-895 chromosome, complete genome
contig-100_117	P2	3171	N	2	1	Sipho	0	0	0	NA	Escherichia coli IHE3034, complete genome	gi|91209055|ref|NC_007946.1| Escherichia coli UTI89 chromosome, complete genome	gi|91209055|ref|NC_007946.1| Escherichia coli UTI89 chromosome, complete genome
contig-100_118	P2	3170	N	1	0	NA	0	0	0	NA	Klebsiella pneumoniae subsp. pneumoniae 1084, complete genome	gi|238892256|ref|NC_012731.1| Klebsiella pneumoniae NTUH-K2044 chromosome, complete genome	gi|238892256|ref|NC_012731.1| Klebsiella pneumoniae NTUH-K2044 chromosome, complete genome
contig-100_12	P2	14582	N	10	2	Asfar	0	2	1	NA	Klebsiella pneumoniae subsp. pneumoniae MGH 78578, complete sequence	gi|152968582|ref|NC_009648.1| Klebsiella pneumoniae subsp. pneumoniae MGH 78578 chromosome, complete genome	gi|152968582|ref|NC_009648.1| Klebsiella pneumoniae subsp. pneumoniae MGH 78578 chromosome, complete genome

contig-100_120	P2	3126	N	1	0	NA	0	1	1	NA	Escherichia coli UM146, complete genome	gi|91209055|ref|NC_007946.1| Escherichia coli UTI89 chromosome, complete genome	gi|91209055|ref|NC_007946.1| Escherichia coli UTI89 chromosome, complete genome
contig-100_121	P2	3093	N	0	0	NA	0	0	0	NA	Klebsiella pneumoniae subsp. pneumoniae NTUH-K2044 DNA, complete genome	gi|238892256|ref|NC_012731.1| Klebsiella pneumoniae NTUH-K2044 chromosome, complete genome	gi|238892256|ref|NC_012731.1| Klebsiella pneumoniae NTUH-K2044 chromosome, complete genome
contig-100_13	P2	14189	N	5	0	NA	0	0	0	NA	Escherichia coli O83:H1 str. NRG 857C, complete genome	gi|387615344|ref|NC_017634.1| Escherichia coli O83:H1 str. NRG 857C chromosome, complete genome	gi|387615344|ref|NC_017634.1| Escherichia coli O83:H1 str. NRG 857C chromosome, complete genome
contig-100_14	P2	13702	N	11	18	Podo	0	0	10	gi|151266287|ref|NC_009643.1| Actinomyces phage Av-1, complete genome	Actinomyces phage Av-1, complete genome	NA	gi|151266287|ref|NC_009643.1| Actinomyces phage Av-1, complete genome
contig-100_15	P2	12919	N	10	7	Sipho	1	1	4	NA	Escherichia coli HS, complete genome	gi|157159467|ref|NC_009800.1| Escherichia coli HS, complete genome	gi|157159467|ref|NC_009800.1| Escherichia coli HS, complete genome
contig-100_17	P2	12184	N	8	7	Sipho	1	0	8	gi|356870600|ref|NC_016158.1| Escherichia phage HK639, complete genome	Klebsiella pneumoniae subsp. rhinoscleromatis strain SB3432, complete genome	gi|529985600|ref|NC_021232.1| Klebsiella pneumoniae subsp. rhinoscleromatis strain SB3432, complete genome	gi|356870600|ref|NC_016158.1| Escherichia phage HK639, complete genome

contig-100_22	P2	9257	N	2	2	Sipho	0	0	2	NA	Enterobacter aerogenes KCTC 2190, complete genome	gi|336246508|ref|NC_015663.1| Enterobacter aerogenes KCTC 2190 chromosome, complete genome	gi|336246508|ref|NC_015663.1| Enterobacter aerogenes KCTC 2190 chromosome, complete genome
contig-100_27	P2	8954	N	19	1	Myo	0	0	1	NA	Uncultured organism clone 1041059767192 genomic sequence	NA	Uncultured organism clone 1041059767192 genomic sequence
contig-100_32	P2	7143	N	2	1	Podo	0	0	1	gi|169936017|ref|NC_010463.1| Enterobacteria phage Fels-2, complete genome	Klebsiella pneumoniae JM45, complete genome	gi|530627845|ref|NC_022082.1| Klebsiella pneumoniae JM45, complete genome	gi|169936017|ref|NC_010463.1| Enterobacteria phage Fels-2, complete genome
contig-100_33	P2	6737	N	4	4	Sipho	0	0	4	gi|155370093|ref|NC_007804.2| Escherichia phage phiV10, complete genome	Escherichia coli APEC O1, complete genome	gi|117622295|ref|NC_008563.1| Escherichia coli APEC O1 chromosome, complete genome	gi|155370093|ref|NC_007804.2| Escherichia phage phiV10, complete genome
contig-100_37	P2	6436	N	3	1	NA	1	2	2	NA	Escherichia coli SE15 DNA, complete genome	gi|387828053|ref|NC_013654.1| Escherichia coli SE15, complete genome	gi|387828053|ref|NC_013654.1| Escherichia coli SE15, complete genome
contig-100_41	P2	6190	N	4	0	NA	0	1	1	NA	Citrobacter koseri ATCC BAA-895, complete genome	gi|157144296|ref|NC_009792.1| Citrobacter koseri ATCC BAA-895 chromosome, complete genome	gi|157144296|ref|NC_009792.1| Citrobacter koseri ATCC BAA-895 chromosome, complete genome
contig-100_42	P2	6137	N	4	1	Sipho	0	0	1	NA	Uncultured bacterium clone HA0AAA21ZA07RM1 genomic sequence	NA	Uncultured bacterium clone HA0AAA21ZA07RM1 genomic sequence

contig-100_44	P2	5940	N	4	1	Sipho	0	1	1	NA	Citrobacter koseri ATCC BAA-895, complete genome	gi|157144296|ref|NC_009792.1| Citrobacter koseri ATCC BAA-895 chromosome, complete genome	gi|157144296|ref|NC_009792.1| Citrobacter koseri ATCC BAA-895 chromosome, complete genome
contig-100_45	P2	5899	Y	8	2	Micro	0	0	2	NA	NA	NA	NA
contig-100_46	P2	5854	N	4	3	Sipho	0	0	1	NA	Salmonella enterica subsp. enterica serovar Typhimurium str. DT2, complete genome	gi|525836786|ref|NC_021814.1| Salmonella enterica subsp. enterica serovar Typhimurium var. 5- str. CFSAN001921, complete genome	gi|525836786|ref|NC_021814.1| Salmonella enterica subsp. enterica serovar Typhimurium var. 5- str. CFSAN001921, complete genome
contig-100_5	P2	20880	N	11	10	Sipho	1	2	8	gi|356870600|ref|NC_016158.1| Escherichia phage HK639, complete genome	Enterobacteria phage phi80, complete genome	gi|170079663|ref|NC_010473.1| Escherichia coli str. K-12 substr. DH10B chromosome, complete genome	gi|356870600|ref|NC_016158.1| Escherichia phage HK639, complete genome
contig-100_50	P2	5715	N	6	1	NA	0	1	2	NA	Escherichia coli 536, complete genome	gi|110640213|ref|NC_008253.1| Escherichia coli 536, complete genome	gi|110640213|ref|NC_008253.1| Escherichia coli 536, complete genome
contig-100_53	P2	5628	N	3	0	NA	0	1	1	NA	Klebsiella pneumoniae subsp. pneumoniae NTUH-K2044 DNA, complete genome	gi|238892256|ref|NC_012731.1| Klebsiella pneumoniae NTUH-K2044 chromosome, complete genome	gi|238892256|ref|NC_012731.1| Klebsiella pneumoniae NTUH-K2044 chromosome, complete genome

contig-100_57	P2	5371	N	2	4	Sipho	0	0	2	gi|77999988|ref|NC_007501.1| Lactobacillus phage Lc-Nu, complete genome	Lactobacillus paracasei subsp. paracasei 8700:2, complete genome	gi|532355525|ref|NC_022112.1| Lactobacillus paracasei subsp. paracasei 8700:2, complete genome	gi|77999988|ref|NC_007501.1| Lactobacillus phage Lc-Nu, complete genome
contig-100_58	P2	5268	N	3	2	Sipho	1	1	2	NA	Enterobacter asburiae LF7a, complete genome	gi|345297184|ref|NC_015968.1| Enterobacter asburiae LF7a chromosome, complete genome	gi|345297184|ref|NC_015968.1| Enterobacter asburiae LF7a chromosome, complete genome
contig-100_62	P2	4833	N	2	0	NA	0	1	0	NA	Escherichia coli ED1a chromosome, complete genome	gi|218687878|ref|NC_011745.1| Escherichia coli ED1a chromosome, complete genome	gi|218687878|ref|NC_011745.1| Escherichia coli ED1a chromosome, complete genome
contig-100_66	P2	4512	N	3	0	NA	0	0	1	NA	Escherichia coli ED1a chromosome, complete genome	gi|218687878|ref|NC_011745.1| Escherichia coli ED1a chromosome, complete genome	gi|218687878|ref|NC_011745.1| Escherichia coli ED1a chromosome, complete genome
contig-100_67	P2	4508	N	3	0	NA	0	0	0	NA	Citrobacter koseri ATCC BAA-895, complete genome	gi|157144296|ref|NC_009792.1| Citrobacter koseri ATCC BAA-895 chromosome, complete genome	gi|157144296|ref|NC_009792.1| Citrobacter koseri ATCC BAA-895 chromosome, complete genome
contig-100_68	P2	4464	N	1	1	Sipho	0	0	1	NA	NA	NA	NA
contig-100_7	P2	20128	N	12	10	Myo	2	0	10	gi|19548987|ref|NC_003444.1| Enterobacteria phage SfV, complete genome	Salmonella enterica subsp. enterica Serovar Cubana str. CFSAN002050, complete genome	gi|525855729|ref|NC_021818.1| Salmonella enterica subsp. enterica Serovar Cubana str. CFSAN002050, complete genome	gi|19548987|ref|NC_003444.1| Enterobacteria phage SfV, complete genome

contig-100_70	P2	4408	N	2	2	Myo	0	0	2	NA	Klebsiella variicola At-22, complete genome	gi|288932888|ref|NC_013850.1| Klebsiella variicola At-22 chromosome, complete genome	gi|288932888|ref|NC_013850.1| Klebsiella variicola At-22 chromosome, complete genome
contig-100_72	P2	4327	N	3	2	Sipho	0	0	1	NA	Klebsiella pneumoniae subsp. pneumoniae 1084, complete genome	gi|238892256|ref|NC_012731.1| Klebsiella pneumoniae NTUH-K2044 chromosome, complete genome	gi|238892256|ref|NC_012731.1| Klebsiella pneumoniae NTUH-K2044 chromosome, complete genome
contig-100_74	P2	4199	N	4	0	NA	2	0	1	NA	Uncultured organism clone VC1BS83TF genomic sequence	NA	Uncultured organism clone VC1BS83TF genomic sequence
contig-100_76	P2	4072	N	4	1	Micro	0	0	0	NA	NA	NA	NA
contig-100_77	P2	4030	N	2	1	Myo	0	0	0	NA	NA	NA	NA
contig-100_78	P2	4014	N	3	1	Phycodna	0	0	1	NA	Escherichia coli UMN026 chromosome, complete genome	gi|218703261|ref|NC_011751.1| Escherichia coli UMN026 chromosome, complete genome	gi|218703261|ref|NC_011751.1| Escherichia coli UMN026 chromosome, complete genome
contig-100_8	P2	16971	N	12	7	Sipho	0	1	3	NA	Escherichia coli 536, complete genome	gi|110640213|ref|NC_008253.1| Escherichia coli 536, complete genome	gi|110640213|ref|NC_008253.1| Escherichia coli 536, complete genome
contig-100_80	P2	3966	N	4	5	Podo	0	0	1	NA	Uncultured bacterium clone LM0ABA5ZD09RM1 genomic sequence	NA	Uncultured bacterium clone LM0ABA5ZD09RM1 genomic sequence

contig-100_82	P2	3882	N	2	2	Sipho	0	0	2	gi|23505445|ref|NC_004313.1| Salmonella phage ST64B, complete genome	Salmonella enterica subsp. enterica serovar Paratyphi B str. SPB7, complete genome	gi|161612313|ref|NC_010102.1| Salmonella enterica subsp. enterica serovar Paratyphi B str. SPB7 chromosome, complete genome	gi|23505445|ref|NC_004313.1| Salmonella phage ST64B, complete genome
contig-100_83	P2	3868	N	2	4	Myo	0	0	2	NA	Enterobacteria phage HK542, complete genome	gi|386612163|ref|NC_017641.1| Escherichia coli UMNK88 chromosome, complete genome	gi|386612163|ref|NC_017641.1| Escherichia coli UMNK88 chromosome, complete genome
contig-100_85	P2	3858	N	3	2	Sipho	0	0	2	NA	NA	NA	NA
contig-100_89	P2	3739	N	2	0	NA	0	0	0	NA	NA	NA	NA
contig-100_90	P2	3725	N	3	2	Micro	0	0	2	NA	NA	NA	NA
contig-100_91	P2	3702	Y	3	0	NA	0	2	0	NA	Klebsiella pneumoniae subsp. pneumoniae NTUH-K2044 DNA, complete genome	gi|238892256|ref|NC_012731.1| Klebsiella pneumoniae NTUH-K2044 chromosome, complete genome	gi|238892256|ref|NC_012731.1| Klebsiella pneumoniae NTUH-K2044 chromosome, complete genome
contig-100_96	P2	3645	N	4	1	Myo	0	0	1	NA	NA	NA	NA
contig-100_97	P2	3639	N	3	1	Pox	0	1	1	NA	Salmonella enterica subsp. arizonae serovar 62:z4,z23:--, complete genome	gi|161501984|ref|NC_010067.1| Salmonella enterica subsp. arizonae serovar 62:z4,z23:- str. RSK2980 chromosome, complete genome	gi|161501984|ref|NC_010067.1| Salmonella enterica subsp. arizonae serovar 62:z4,z23:- str. RSK2980 chromosome, complete genome
contig-100_98	P2	3638	N	3	2	Sipho	0	0	2	NA	NA	NA	NA

contig-100_99	P2	3595	N	4	4	Sipho	0	0	4	NA	Salmonella enterica subsp. enterica Serovar Cubana str. CFSAN002050, complete genome	gi|525855729|ref|NC_021818.1| Salmonella enterica subsp. enterica Serovar Cubana str. CFSAN002050, complete genome	gi|525855729|ref|NC_021818.1| Salmonella enterica subsp. enterica Serovar Cubana str. CFSAN002050, complete genome
1	P3 pre FMT	114441	N	60	46	Sipho	2	2	25	NA	Uncultured organism clone Turk_fec101 genomic sequence	gi|347524522|ref|NC_015975.1| Lactobacillus ruminis ATCC 27782 chromosome, complete genome	gi|347524522|ref|NC_015975.1| Lactobacillus ruminis ATCC 27782 chromosome, complete genome
2	P3 pre FMT	46143	Y	30	36	Sipho	5	0	13	NA	Clostridiales sp. SSC/2 draft genome	gi|479158859|ref|NC_021016.1| Butyrate-producing bacterium SSC/2, complete genome	gi|479158859|ref|NC_021016.1| Butyrate-producing bacterium SSC/2, complete genome
3	P3 pre FMT	65627	Y	27	24	Sipho	2	0	12	NA	Bacteroides vulgatus ATCC 8482, complete genome	gi|150002608|ref|NC_009614.1| Bacteroides vulgatus ATCC 8482 chromosome, complete genome	gi|150002608|ref|NC_009614.1| Bacteroides vulgatus ATCC 8482 chromosome, complete genome
4	P3 pre FMT	36021	N	19	17	Sipho	2	0	4	NA	Clostridiales sp. SSC/2 draft genome	gi|479158859|ref|NC_021016.1| Butyrate-producing bacterium SSC/2, complete genome	gi|479158859|ref|NC_021016.1| Butyrate-producing bacterium SSC/2, complete genome
5	P3 pre FMT	26614	N	22	18	Sipho	0	0	8	NA	NA	NA	NA
6	P3 pre FMT	39237	N	15	18	Sipho	0	0	4	NA	Eubacterium rectale DSM 17629 draft genome	gi|479140210|ref|NC_021010.1| Eubacterium rectale DSM 17629 draft genome	gi|479140210|ref|NC_021010.1| Eubacterium rectale DSM 17629 draft genome

7	P3 pre FMT	28555	N	22	26	Sipho	2	1	13	NA	Clostridium botulinum F str. 230613, complete genome	gi|153937894|ref|NC_009699.1| Clostridium botulinum F str. Langeland chromosome, complete genome	gi|153937894|ref|NC_009699.1| Clostridium botulinum F str. Langeland chromosome, complete genome
8	P3 pre FMT	22210	N	14	21	Sipho	5	0	7	NA	NA	NA	NA
9	P3 pre FMT	52097	N	36	22	Sipho	0	1	8	NA	NA	NA	NA
10	P3 pre FMT	16262	N	8	19	Sipho	0	0	8	gi|374531191|ref|NC_016761.1| Salmonella phage SPN1S, complete genome	Escherichia coli 0127:H6 E2348/69 complete genome, strain E2348/69	gi|215485161|ref|NC_011601.1| Escherichia coli O127:H6 str. E2348/69 chromosome, complete genome	gi|374531191|ref|NC_016761.1| Salmonella phage SPN1S, complete genome
11	P3 pre FMT	23694	N	12	11	Sipho	0	0	9	NA	NA	NA	NA
12	P3 pre FMT	15333	N	24	4	Sipho	0	0	4	NA	Bifidobacterium phage Bbif-1, complete sequence	gi|311063459|ref|NC_014638.1| Bifidobacterium bifidum PRL2010 chromosome, complete genome	gi|311063459|ref|NC_014638.1| Bifidobacterium bifidum PRL2010 chromosome, complete genome
13	P3 pre FMT	7799	N	4	5	Myo	0	0	2	NA	Uncultured organism clone 1041059767192 genomic sequence	NA	Uncultured organism clone 1041059767192 genomic sequence
14	P3 pre FMT	7306	N	4	12	Sipho	0	0	3	NA	NA	NA	NA
15	P3 pre FMT	7956	N	6	8	Sipho	0	0	2	NA	NA	NA	NA
16	P3 pre FMT	6584	N	6	7	Sipho	0	0	0	NA	Bacteroides fragilis conjugal transfer (of antibiotic-resistance factors) protein (btgA and btgB) genes, complete cds	gi|325278757|ref|NC_015160.1| Odoribacter splanchnicus DSM 20712 chromosome, complete genome	gi|325278757|ref|NC_015160.1| Odoribacter splanchnicus DSM 20712 chromosome, complete genome
17	P3 pre FMT	8661	N	5	5	Myo	0	1	3	NA	NA	NA	NA
18	P3 pre FMT	16391	N	6	12	Sipho	2	1	3	NA	NA	NA	NA
19	P3 pre FMT	6916	N	5	5	Sipho	0	0	2	NA	NA	NA	NA
20	P3 pre FMT	6348	N	7	7	Myo	0	0	4	NA	NA	NA	NA

21	P3 pre FMT	14581	N	13	7	Podo	1	1	5	NA	Ruminococcus torques L2-14 draft genome	gi|479155735|ref|NC_021015.1| Ruminococcus torques L2-14 draft genome	gi|479155735|ref|NC_021015.1| Ruminococcus torques L2-14 draft genome
22	P3 pre FMT	7182	N	1	4	Sipho	0	0	0	NA	NA	NA	NA
23	P3 pre FMT	11950	N	7	10	Sipho	0	0	0	NA	NA	NA	NA
24	P3 pre FMT	18330	N	11	9	Sipho	0	0	0	NA	NA	NA	NA
25	P3 pre FMT	8864	N	7	12	Sipho	1	0	1	NA	NA	NA	NA
26	P3 pre FMT	5646	N	3	7	Micro	0	0	2	NA	Bacteroides vulgatus ATCC 8482, complete genome	gi|150002608|ref|NC_009614.1| Bacteroides vulgatus ATCC 8482 chromosome, complete genome	gi|150002608|ref|NC_009614.1| Bacteroides vulgatus ATCC 8482 chromosome, complete genome
27	P3 pre FMT	7667	N	7	6	Sipho	0	0	5	NA	NA	NA	NA
28	P3 pre FMT	6000	N	5	4	Sipho	0	0	3	NA	NA	NA	NA
29	P3 pre FMT	12125	N	10	10	Sipho	3	0	8	NA	NA	NA	NA
30	P3 pre FMT	5888	N	4	3	Micro	0	0	2	NA	NA	NA	NA
31	P3 pre FMT	4294	N	2	8	Sipho	0	0	1	NA	Ruminococcus torques L2-14 draft genome	gi|479155735|ref|NC_021015.1| Ruminococcus torques L2-14 draft genome	gi|479155735|ref|NC_021015.1| Ruminococcus torques L2-14 draft genome
32	P3 pre FMT	7544	N	5	4	Sipho	1	0	2	NA	Faecalibacterium prausnitzii L2/6 draft genome	gi|479208076|ref|NC_021042.1| Faecalibacterium prausnitzii L2-6, complete genome	gi|479208076|ref|NC_021042.1| Faecalibacterium prausnitzii L2-6, complete genome
33	P3 pre FMT	4969	N	4	4	Sipho	0	0	0	NA	Uncultured bacterium clone LM0ABA40ZG10FM1 genomic sequence	NA	Uncultured bacterium clone LM0ABA40ZG10FM1 genomic sequence

34	P3 pre FMT	4984	N	4	5	Sipho	0	0	1	NA	Uncultured organism clone 1041059765628 genomic sequence	gi|150006674|ref|NC_009615.1| Parabacteroides distasonis ATCC 8503 chromosome, complete genome	gi|150006674|ref|NC_009615.1| Parabacteroides distasonis ATCC 8503 chromosome, complete genome
35	P3 pre FMT	5100	N	5	5	Sipho	0	0	2	NA	NA	NA	NA
36	P3 pre FMT	3940	N	2	8	Sipho	0	0	0	NA	NA	NA	NA
37	P3 pre FMT	4880	N	3	11	Sipho	0	0	0	NA	NA	NA	NA
38	P3 pre FMT	11091	N	15	6	Sipho	0	0	3	NA	Bifidobacterium phage Bbif-1, complete sequence	gi|311063459|ref|NC_014638.1| Bifidobacterium bifidum PRL2010 chromosome, complete genome	gi|311063459|ref|NC_014638.1| Bifidobacterium bifidum PRL2010 chromosome, complete genome
39	P3 pre FMT	7373	Y	3	5	Sipho	1	0	3	NA	NA	NA	NA
40	P3 pre FMT	10690	N	8	9	Sipho	1	0	6	NA	Eubacterium siraeum 70/3 draft genome	gi|479143419|ref|NC_021011.1| Eubacterium siraeum 70/3 draft genome	gi|479143419|ref|NC_021011.1| Eubacterium siraeum 70/3 draft genome
41	P3 pre FMT	4181	N	1	3	Sipho	0	0	1	NA	NA	NA	NA
42	P3 pre FMT	5867	N	3	6	Sipho	0	0	2	NA	NA	NA	NA
43	P3 pre FMT	4929	N	3	5	Sipho	0	0	0	NA	NA	NA	NA
44	P3 pre FMT	4815	N	5	7	Sipho	0	0	1	NA	Unidentified phage clone 1013_scaffold47 genomic sequence	NA	Unidentified phage clone 1013_scaffold47 genomic sequence
45	P3 pre FMT	4547	N	5	2	Sipho	0	1	1	NA	NA	NA	NA
46	P3 pre FMT	5756	N	1	9	Sipho	0	0	0	NA	NA	NA	NA
47	P3 pre FMT	3150	N	2	3	Sipho	0	0	0	NA	Uncultured organism clone 1041059767004 genomic sequence	NA	Uncultured organism clone 1041059767004 genomic sequence
48	P3 pre FMT	4817	N	6	4	Myo	0	0	1	NA	Faecalibacterium prausnitzii L2/6 draft genome	gi|479208076|ref|NC_021042.1| Faecalibacterium prausnitzii L2-6, complete genome	gi|479208076|ref|NC_021042.1| Faecalibacterium prausnitzii L2-6, complete genome
49	P3 pre FMT	4683	N	2	3	Sipho	0	0	0	NA	NA	NA	NA

50	P3 pre FMT	4248	Y	5	0	NA	0	0	0	NA	Bacteroides fragilis plasmid pBFUK1 DNA, complete genome, strain: GAI92082	NA	Bacteroides fragilis plasmid pBFUK1 DNA, complete genome, strain: GAI92082
51	P3 pre FMT	4711	N	3	6	Sipho	0	0	2	NA	NA	NA	NA
52	P3 pre FMT	4463	N	2	2	Myo	1	1	2	NA	Bacteroides helcogenes P 36-108, complete genome	gi|319899888|ref|NC_014933.1| Bacteroides helcogenes P 36-108 chromosome, complete genome	gi|319899888|ref|NC_014933.1| Bacteroides helcogenes P 36-108 chromosome, complete genome
53	P3 pre FMT	3058	N	4	1	Sipho	1	0	1	NA	NA	NA	NA
54	P3 pre FMT	14787	N	8	6	Sipho	1	0	3	NA	NA	NA	NA
55	P3 pre FMT	4786	N	3	7	Sipho	0	0	2	NA	Unidentified phage clone 1013_scaffold47 genomic sequence	gi|479140210|ref|NC_021010.1| Eubacterium rectale DSM 17629 draft genome	gi|479140210|ref|NC_021010.1| Eubacterium rectale DSM 17629 draft genome
56	P3 pre FMT	3812	N	4	1	Myo	0	0	0	NA	Odoribacter splanchnicus DSM 20712, complete genome	gi|325278757|ref|NC_015160.1| Odoribacter splanchnicus DSM 20712 chromosome, complete genome	gi|325278757|ref|NC_015160.1| Odoribacter splanchnicus DSM 20712 chromosome, complete genome
57	P3 pre FMT	3018	N	2	5	Sipho	0	0	2	NA	NA	NA	NA
58	P3 pre FMT	4182	N	2	2	Sipho	0	0	1	NA	NA	NA	NA
59	P3 pre FMT	3471	N	4	5	Sipho	0	0	3	NA	NA	NA	NA
63	P3 pre FMT	3199	N	1	0	NA	0	0	0	NA	Bacteroides vulgatus ATCC 8482, complete genome	gi|150002608|ref|NC_009614.1| Bacteroides vulgatus ATCC 8482 chromosome, complete genome	gi|150002608|ref|NC_009614.1| Bacteroides vulgatus ATCC 8482 chromosome, complete genome

64	P3 pre FMT	6543	N	9	6	Sipho	0	1	4	NA	Unidentified phage clone 2011_scaffold 152 genomic sequence	gi|479140210|ref|NC_021010.1| Eubacterium rectale DSM 17629 draft genome	gi|479140210|ref|NC_021010.1| Eubacterium rectale DSM 17629 draft genome
66	P3 pre FMT	3158	N	2	1	Sipho	0	0	1	NA	Uncultured organism clone 1041059765390 genomic sequence	NA	Uncultured organism clone 1041059765390 genomic sequence
67	P3 pre FMT	4394	N	5	3	Sipho	0	0	1	NA	NA	NA	NA
71	P3 pre FMT	4859	N	4	2	Sipho	0	0	1	NA	NA	NA	NA
72	P3 pre FMT	7788	N	6	1	Myo	0	0	2	NA	NA	NA	NA
75	P3 pre FMT	4341	N	2	3	Pox	0	0	1	NA	NA	NA	NA
78	P3 pre FMT	3763	N	3	4	Sipho	0	0	2	NA	NA	NA	NA
81	P3 pre FMT	3788	N	3	1	Sipho	0	0	0	NA	NA	NA	NA
85	P3 pre FMT	3007	N	3	0	NA	2	0	0	NA	Bacteroides fragilis strain B16078 carbapenem-resistance protein (cfiA) gene, partial cds; and insertion sequence IS614B Tp614 gene, complete cds	gi|302344773|ref|NC_014370.1| Prevotella melaninogenica ATCC 25845 chromosome I, complete sequence	gi|302344773|ref|NC_014370.1| Prevotella melaninogenica ATCC 25845 chromosome I, complete sequence
contig-100_1	P3 pre FMT	51800	N	35	22	Sipho	1	0	10	NA	NA	NA	NA
contig-100_101	P3 pre FMT	3426	N	2	2	Sipho	0	0	2	NA	NA	NA	NA
contig-100_102	P3 pre FMT	3424	N	2	2	Sipho	1	0	1	NA	NA	NA	NA
contig-100_105	P3 pre FMT	3352	N	2	0	NA	0	0	0	NA	Atopobium parvulum DSM 20469, complete genome	gi|257783814|ref|NC_013203.1| Atopobium parvulum DSM 20469 chromosome, complete genome	gi|257783814|ref|NC_013203.1| Atopobium parvulum DSM 20469 chromosome, complete genome
contig-100_107	P3 pre FMT	3323	N	2	1	Sipho	1	0	2	NA	NA	NA	NA
contig-100_110	P3 pre FMT	3268	N	1	2	Sipho	0	0	0	NA	NA	NA	NA
contig-100_113	P3 pre FMT	3236	N	0	1	Sipho	0	0	0	NA	NA	NA	NA
contig-100_114	P3 pre FMT	3233	N	0	1	Sipho	0	0	0	NA	NA	NA	NA

contig-100_116	P3 pre FMT	3161	N	4	0	NA	0	0	0	NA	Parabacteroides distasonis ATCC 8503, complete genome	gi|150006674|ref|NC_009615.1| Parabacteroides distasonis ATCC 8503 chromosome, complete genome	gi|150006674|ref|NC_009615.1| Parabacteroides distasonis ATCC 8503 chromosome, complete genome
contig-100_118	P3 pre FMT	3141	N	2	0	NA	0	0	0	gi|30387379|ref|NC_004775.1| Enterobacteria phage epsilon15, complete genome	Escherichia coli 0127:H6 E2348/69 complete genome, strain E2348/69	gi|215485161|ref|NC_011601.1| Escherichia coli O127:H6 str. E2348/69 chromosome, complete genome	gi|30387379|ref|NC_004775.1| Enterobacteria phage epsilon15, complete genome
contig-100_123	P3 pre FMT	3079	N	3	1	Sipho	1	0	2	NA	Clostridiales sp. SS3/4 draft genome	gi|479192860|ref|NC_021035.1| Butyrate-producing bacterium SS3/4, complete genome	gi|479192860|ref|NC_021035.1| Butyrate-producing bacterium SS3/4, complete genome
contig-100_124	P3 pre FMT	3073	N	2	0	NA	0	0	1	NA	NA	NA	NA
contig-100_125	P3 pre FMT	3064	N	1	0	NA	0	0	0	NA	NA	NA	NA
contig-100_126	P3 pre FMT	3058	N	2	1	Sipho	0	0	1	NA	NA	NA	NA
contig-100_17	P3 pre FMT	12681	N	9	8	Sipho	0	0	2	NA	Unidentified phage clone 1013_scaffold1563 genomic sequence	NA	Unidentified phage clone 1013_scaffold1563 genomic sequence
contig-100_18	P3 pre FMT	12185	N	8	9	Sipho	1	0	0	NA	NA	NA	NA
contig-100_19	P3 pre FMT	11949	N	8	1	Sipho	0	0	1	NA	Uncultured bacterium clone LM0ABA44ZD03FM1 genomic sequence	NA	Uncultured bacterium clone LM0ABA44ZD03FM1 genomic sequence
contig-100_2	P3 pre FMT	46062	Y	46	16	Sipho	1	1	17	NA	NA	NA	NA
contig-100_20	P3 pre FMT	10950	N	9	7	Sipho	0	0	2	NA	Uncultured organism clone VC1CH09TR genomic sequence	gi|325278757|ref|NC_015160.1| Odoribacter splanchnicus DSM 20712 chromosome, complete genome	gi|325278757|ref|NC_015160.1| Odoribacter splanchnicus DSM 20712 chromosome, complete genome
contig-100_21	P3 pre FMT	10224	N	4	7	Podo	1	0	1	NA	NA	NA	NA

contig-100_23	P3 pre FMT	8101	N	10	2	Sipho	0	0	2	NA	Bacteroides helcogenes P 36-108, complete genome	gi|319899888|ref|NC_014933.1| Bacteroides helcogenes P 36-108 chromosome, complete genome	gi|319899888|ref|NC_014933.1| Bacteroides helcogenes P 36-108 chromosome, complete genome
contig-100_26	P3 pre FMT	7260	N	5	1	Sipho	0	0	0	NA	NA	NA	NA
contig-100_28	P3 pre FMT	6896	N	3	5	Myo	1	0	3	gi|228861315|ref|NC_012638.1| Enterobacteria phage RB14, complete genome	Enterobacteria phage vB_EcoM_ACG-C40, complete genome	gi|257091663|ref|NC_013194.1| Candidatus Accumulibacter phosphatis clade IIA str. UW-1 chromosome, complete genome	gi|228861315|ref|NC_012638.1| Enterobacteria phage RB14, complete genome
contig-100_29	P3 pre FMT	6811	Y	5	0	NA	0	0	0	NA	NA	NA	NA
contig-100_34	P3 pre FMT	6549	N	4	0	NA	0	0	0	NA	NA	NA	NA
contig-100_37	P3 pre FMT	6045	Y	6	4	Micro	0	0	3	NA	NA	NA	NA
contig-100_4	P3 pre FMT	40628	N	40	15	Sipho	4	1	11	NA	NA	NA	NA
contig-100_40	P3 pre FMT	5978	N	4	0	NA	1	0	0	NA	Clostridiales sp. SSC/2 draft genome	gi|479158859|ref|NC_021016.1| Butyrate-producing bacterium SSC/2, complete genome	gi|479158859|ref|NC_021016.1| Butyrate-producing bacterium SSC/2, complete genome
contig-100_43	P3 pre FMT	5934	N	7	3	Adeno	0	0	2	NA	NA	NA	NA
contig-100_44	P3 pre FMT	5828	N	0	1	Sipho	0	0	0	NA	NA	NA	NA
contig-100_47	P3 pre FMT	5728	N	7	6	Myo	0	0	6	NA	NA	NA	NA
contig-100_49	P3 pre FMT	5664	N	3	1	Phycodna	0	2	2	NA	Uncultured organism clone 1041059765324 genomic sequence	gi|319899888|ref|NC_014933.1| Bacteroides helcogenes P 36-108 chromosome, complete genome	gi|319899888|ref|NC_014933.1| Bacteroides helcogenes P 36-108 chromosome, complete genome
contig-100_5	P3 pre FMT	30422	N	11	14	Sipho	1	1	3	NA	Unidentified phage clone 1013_scaffold1563 genomic sequence	NA	Unidentified phage clone 1013_scaffold1563 genomic sequence
contig-100_52	P3 pre FMT	5431	N	2	1	Sipho	0	0	0	NA	NA	NA	NA
contig-100_53	P3 pre FMT	5420	N	5	2	Myo	0	0	2	NA	NA	NA	NA

contig-100_54	P3 pre FMT	5353	N	3	3	Sipho	1	0	3	NA	NA	NA	NA
contig-100_56	P3 pre FMT	5302	N	4	3	Sipho	0	0	0	NA	Bacteroides vulgatus ATCC 8482, complete genome	gi|150002608|ref|NC_009614.1| Bacteroides vulgatus ATCC 8482 chromosome, complete genome	gi|150002608|ref|NC_009614.1| Bacteroides vulgatus ATCC 8482 chromosome, complete genome
contig-100_57	P3 pre FMT	5295	N	3	5	Sipho	0	0	0	NA	Clostridiales sp. SSC/2 draft genome	gi|479158859|ref|NC_021016.1| Butyrate-producing bacterium SSC/2, complete genome	gi|479158859|ref|NC_021016.1| Butyrate-producing bacterium SSC/2, complete genome
contig-100_59	P3 pre FMT	5078	N	3	3	Sipho	1	1	3	gi|374531191|ref|NC_016761.1| Salmonella phage SPN1S, complete genome	Escherichia coli 0127:H6 E2348/69 complete genome, strain E2348/69	gi|215485161|ref|NC_011601.1| Escherichia coli O127:H6 str. E2348/69 chromosome, complete genome	gi|374531191|ref|NC_016761.1| Salmonella phage SPN1S, complete genome
contig-100_61	P3 pre FMT	4928	N	3	1	Sipho	1	0	1	NA	NA	NA	NA
contig-100_64	P3 pre FMT	4714	N	2	1	Podo	0	0	1	NA	NA	NA	NA
contig-100_65	P3 pre FMT	4679	N	1	0	NA	0	0	0	NA	Bacteroides helcogenes P 36-108, complete genome	gi|319899888|ref|NC_014933.1| Bacteroides helcogenes P 36-108 chromosome, complete genome	gi|319899888|ref|NC_014933.1| Bacteroides helcogenes P 36-108 chromosome, complete genome
contig-100_67	P3 pre FMT	4348	N	4	3	Sipho	1	0	3	NA	Clostridiales sp. SSC/2 draft genome	gi|479158859|ref|NC_021016.1| Butyrate-producing bacterium SSC/2, complete genome	gi|479158859|ref|NC_021016.1| Butyrate-producing bacterium SSC/2, complete genome
contig-100_69	P3 pre FMT	4271	N	2	3	Sipho	1	0	0	NA	NA	NA	NA
contig-100_71	P3 pre FMT	4248	N	3	3	Sipho	0	0	2	NA	NA	NA	NA
contig-100_72	P3 pre FMT	4206	N	3	2	Sipho	0	0	0	NA	NA	NA	NA
contig-100_75	P3 pre FMT	4155	N	3	0	NA	0	0	0	NA	NA	NA	NA
contig-100_76	P3 pre FMT	4154	N	5	1	Micro	0	0	1	NA	NA	NA	NA
contig-100_77	P3 pre FMT	4135	N	2	2	Sipho	1	0	1	NA	NA	NA	NA
contig-100_78	P3 pre FMT	4131	N	3	1	Phycodna	0	0	0	NA	NA	NA	NA

contig-100_81	P3 pre FMT	4081	N	1	0	NA	0	0	0	NA	NA	NA	NA
contig-100_84	P3 pre FMT	3853	N	1	0	NA	0	0	0	NA	NA	NA	NA
contig-100_91	P3 pre FMT	3628	N	2	0	NA	0	0	0	NA	NA	NA	NA
contig-100_94	P3 pre FMT	3506	N	3	5	Sipho	1	1	2	NA	NA	NA	NA
contig-100_95	P3 pre FMT	3505	N	2	0	NA	1	0	0	NA	Uncultured bacterium clone HA0AAA10ZG05FM1 genomic sequence	NA	Uncultured bacterium clone HA0AAA10ZG05FM1 genomic sequence
contig-100_96	P3 pre FMT	3482	N	1	1	Myo	0	0	0	NA	NA	NA	NA
contig-100_98	P3 pre FMT	3474	N	4	3	Sipho	0	0	3	NA	NA	NA	NA
contig-100_99	P3 pre FMT	3451	N	3	4	Sipho	0	0	1	NA	NA	NA	NA
