# Supplementary material for: Transfer of Viral Communities between Human Individuals during Fecal Microbiota Transplantation
Source: mBio. 2016 Mar 29;7(2):e00322-16. doi: 10.1128/mBio.00322-16 (PMC4817255; doi:10.1128/mBio.00322-16)
Supplement: Table S5 — Primers used for qPCR analysis. Data represent the oligonucleotides used for 16S and VLP contig verification qPCR. [file mbo002162747st5.pdf]

Supplementary Table 5. Oligonucleotides used.

Name	Sequence	Description
16S rRNA	AGAGTTTGATCCTGGCTCAG	Forward primer for qPCR
	CTGCTGCCTYCCGTA	Reverse primer for qPCR
	5' - /56-FAM/TAA +CA+C ATG +CA+A GT+C GA/3BHQ_1/ - 3'	Probe<br>*

*   + symbol precedes the position of LNA base.

Contig-17	ACTGACACATCTCTTCCTTGCAAAATAAC GCTGCCAATGCTAGTTGCTTCCTGCTTCT GTCCGAATGTGGCGGAGTACCCATCCTG AGACCCGGCACCACGAATAAACGCCGCC TGCAAGTTTGGAAGGGCAAAGG	gBlock
	ACTGACACATCTCTTCCTTGC	Forward primer for qPCR
	CCTTTGCCCTTCCAAACTTG	Reverse primer for qPCR
Contig-95-22	TGTTTGTGGATAGTACGGCAGAAGTTGC ACTCCCGGAAACACTTACGTTGCACTGG GATGCAAAAGACGAAAC	gBlock
	TGTTTGTGGATAGTACGGCAG	Forward primer for qPCR
	GTTTCGTCTTTTGCATCCCAG	Reverse primer for qPCR
Contig-95-100	ATCGTACCAACTTCCCAACCAGTCGAGGC CGGGGAGGTAATCTGAGGCATGGCGTAC TCCGTCTCAGGAATCCAGGCACTTTCCGT ATTCTCACCACAGAACTCTTTCCAGTGCT GCCACACAAGGCGATTAGGAA CGAAGA	gBlock
	ATCGTACCAACTTCCCAACC	Forward primer for qPCR
	TCAACGTTCCTAATCGCCTTG	Reverse primer for qPCR
Contig-95-203	CTCAATCTCCTTCCCACTCAGACGAAACG CACAGAGGAAATGGAGAGCTATCAGCAG TTTTCCACCCCGCCAAATATCGCCTATAT	gBlock
	CTCAATCTCCTTCCCACTCAG	Forward primer for qPCR
	ATATAGGCGATATTTGGCGGG	Reverse primer for qPCR
Contig-66-Siphoviridae	CGCCGGGAATCTCTCTTATTTTTGTGATG TCATAAAACACTCTGCCATTGGGTGAGTT GGCTATATTGATGGTTCCCTCGAAGGTTT TGCCCCCCAATTGAAACCGGGTGGTATA ATAATCCCAGCCCAAGCTC	gBlock
	CGCCGGGAATCTCTCTTATTT	Forward primer for qPCR
	GAGCTTGGGCTGGGATTATT	Reverse primer for qPCR
Contig-100-176 Siphoviridae	GACAACTCGTTTCTGGGATGTAAATGCGA ATGCTACATCCATTTTTGCGGTAGACGGG AGATGGTTTAACAGGGGTGGCATAAAGA	gBlock

	GGTGAGTGCTTGTGG	
	GACAACTCGTTTCTGGGATGTA	Forward primer for qPCR
	CCACAAGCACTCACCTCTTTA	Reverse primer for qPCR
Contig-100-230 Siphoviridae	ATACTGGAGACCTGGCTATGAAGTTCAAAGAAGCTTATATTAAGCAATTCAACGCTATGGAAGCCGCCCTGCAAGGAAAGCGAATTGAGCGTGAGAAGGGA	gBlock
	ATACTGGAGACCTGGCTATGA	Forward primer for qPCR
	TCCCTTCTCACGCTCAATTC	Reverse primer for qPCR
Contig-100-72 Siphoviridae	CCTGATTCCTCCGATTCATTCTCTTGCAGGGCGAAGGGCAGCGCTGTCTTTTCCTTCCTCGGCCTGGCCGCAGGCCTGGGCCTCCCGGCTACAAAATACCGGATGGCGTCAG	gBlock
	CCTGATTCCTCCGATTCATTCT	Forward primer for qPCR
	CTGACGCCATCCGGTATTT	Reverse primer for qPCR
