# Supplementary material for: Transfer of Viral Communities between Human Individuals during Fecal Microbiota Transplantation
Source: mBio. 2016 Mar 29;7(2):e00322-16. doi: 10.1128/mBio.00322-16 (PMC4817255; doi:10.1128/mBio.00322-16)
Supplement: Text S1 — Supplemental methods. Download [file mbo002162747s1.docx]

**Supplemental Methods**

Classifying Viral Read

All MiSeq reads were BLAST aligned to a custom database ([Norman et al., 2015](#_ENREF_9)) using an e-value 10^-3^ and max_target_seqs 100. All default settings were used unless otherwise stated. The BLAST outputs (compressed BLAST XML format) were analyzed using MEGAN ([Huson et al., 2011](#_ENREF_3)) with the following parameters: minsupport=1, minscore=30.0, toppercent=10.0, and mincomplexity=0.44. All classifications that did not include a viral family (e.g. “root;Viruses;dsDNA viruses, no RNA stage; unclassified dsDNA phages”, “root;Viruses;unclassified phages;Enterococcus phage EF62phi”) were considered Unclassified. Proportions of the classified viral families were calculated. Samples with less than 50,000 reads (i.e. two negative control SM buffer samples) were excluded.

Bacterial Data

Previously published bacterial 16S data ([Kellermayer et al., 2015](#_ENREF_4)) was reanalyzed using R packages qiimer and vegan. Shannon diversity was calculated for each sample. A principal coordinate ordination plot, based on the Weighted Unifrac distances, was built. The Weighted Unifrac distance between each patient sample and the centroid of the donor samples was calculated.

Assembling, Annotating, and Visualizing Viral Contigs

Sequence reads were first assembled into contigs using IDBA-UD ([Peng et al., 2012](#_ENREF_10)) with pre-correction and minimum and maximum k-mer lengths set to default 20 and 100 bp respectively. Minimo ([Treangen et al., 2011](#_ENREF_15)), a program which utilizes a conservative overlap-layout-consensus algorithm, was used to further combine the contigs. Contigs were built from 1) all 4 donor samples, including two crude stool samples and two processed samples which were blended and filtered to generate the liquid preparation used for the FMTs, 2) patient 1 pre-FMT’s samples, 3), patient 2 pre-FMT samples, and 4) patient 3 pre-FMT samples. Each of the three patients’ pre-FMT sample (after Genomiphi amplification) was sequenced thrice. The sequencing data from each patient’s pre-FMT samples was combined.

After the contigs were built, reads from each donor and patient sample were aligned using Bowtie2 ([Langmead and Salzberg, 2012](#_ENREF_5)) to each of the four sets of contigs. Bowtie’s very-sensitive parameters (i.e. -D 20 -R 3 -N 0 -L 20 -i S,1,0.50) were used. Samtools and bedtools ([Li et al., 2009](#_ENREF_7); [Quinlan and Hall, 2010](#_ENREF_11)) were used to parse the alignment results and assess genome coverage.

In addition to the number of reads matching each of the four sets of viral contigs, length of the viral contig was determined. The circularity of each contig was also assessed by looking for at least 10 and at most 1000 bp of overlap between the end and beginning of the contig sequences that were greater than 3500 bp in length.

Entire contig sequences were then compared using BLAST with an e-value threshold 10^-5^ to NCBI reference viral database, nucleotide (NT) database, and bacterial genomes database (downloaded January 7, 2015 from NCBI Refseq). Top matches were recorded. Additional “host” information was given to each contig using the following rules: 1) if a match to a reference viral database was found, the top match was used, 2) if no match to the viral database was found but a match to a bacterial genome was found, the match to the bacterial genome was used, and 3) if no match to the viral or bacterial reference genomes was found but a match was found to the NT database, that match was used.

Contigs were then annotated with open reading frames (ORFs) using Glimmer ([Salzberg et al., 1998](#_ENREF_13)), a program that utilizes the Interpolated Context Model to predict genes. Minimum gene length was set to 100 bp. The number of predicted ORFs was recorded. Predicted ORFs, in protein form, were BLAST aligned to ORFs of known viral families. The number of ORFs matching predicted ORFs from reference viral family sequences was recorded. A putative viral family was given to each contig based on the viral family with the most ORF matches. For example, if a contig had 4 predicted ORFs, 3 of which matched ORFs in known Siphoviridae viruses and 1 of which matched an ORF in a known Podoviridae virus, the contig would be putatively annotated as belonging to the Siphoviridae family. If only 1 of the 4 predicted ORFs matched an ORF in a known Siphoviridae virus and the remaining 3 ORFs did not have any matches, the contig would be assigned to the Siphoviridae family. In the rare instance were an equal number of ORFs matched two families (e.g. 2 ORFs matched one viral family and another 2 ORFs matched another family), a viral family was selected at random.

Seven contigs contained ORFs that showed a top blast hit to an animal cell virus ORF. In all cases, the matched ORFs were a minority of all ORFs on the contig. In 5 out of 7 cases, the contig as a whole found a better alignment to a phage or bacterial sequence. In the remaining 2 cases, no better matches were found but because the animal cell viruses matched 1 out of 3 or 1 out of 6 contig ORFs, we concluded that these contigs were likely derived from phage or bacteria as well.

In addition to using ORFs for providing putative viral family names, ORFs were aligned to conserved domains of integrase proteins with Pfam ([Sonnhammer et al., 1998](#_ENREF_14)), the Virulence Factor Database ([Chen et al., 2012](#_ENREF_2)), and Aclame database of mobile genetic elements ([Leplae et al., 2010](#_ENREF_6)). The number of matches to each of these databases was recorded.

Predicted ORFs, in protein form, were also compared to the Pfam ([Sonnhammer et al., 1998](#_ENREF_14)) database of conserved domains with Reversed Position Specific BLAST (RPS-BLAST). Low compositional complexity regions were not masked and default e-value of 0.01 was used for the search. Pfam families were grouped using custom database with Pfam domain identifiers linked to phage regulation function ([Minot et al., 2011](#_ENREF_8)). Contigs from 1) 4 donor samples, 2) patient 1 pre-FMT, 3) patient 2 pre-FMT, and 4) patient 3 pre-FMT were annotated with phage gene types.

Last, the number of reads from each sample mapping to each contigs were plotted. The size of the circle was set to represent percent of the contig covered by reads from that sample. The colors of each sample were used to distinguish the donor, each patient, and the control samples. Contig names were annotated with length and putative viral family (if any).

Donor contigs in patients during and post-FMT

Contig transfer was assessed separately for each patient and and then for all patients combined. Transfer meant 1) an increase in the number of reads matching a contig in a patient’s during FMT and/or post-FMT sample(s) from the patient’s pre-FMT sample, 2) at least 5 paired reads matching the contig during and/or post-FMT and 3) at least 50% coverage of the contig during and/or post-FMT. The number of viral contigs that transferred (as well as the contigs’ putative viral family name) was recorded for each patient. Viral contigs, built from the donor samples, could have been seen in a patient’s during FMT sample, patient’s post-FMT sample or both. The number of transfers as well as putative viral family classification was represented using Sankey plots (using R riverplot package). Contigs that were seen to be transferred in at least 2 of the 3 patients were also recorded. 2x2 contingency tables were built for each viral family and transfer, as well as pfams and transfer. Fisher’s exact test, and odds ratio, was used to calculate significance.

Verification of donor contigs

To verify our sequence results, four contigs were selected for verification with qPCR. Prior to qPCR, the sequence data from all of the samples was re-aligned to each contig and visualized using IGV ([Robinson et al., 2011](#_ENREF_12)). Manual visual inspection confirmed the contig transfer plots, and qPCR assays were developed. After seeing an overrepresentation of Siphoviridae being transferred, 4 contigs specifically annotating as Siphoviridae were assessed for qPCR. Alignment of all sample reads to these contigs was again visualized in IGV and manually confirmed to match the contig transfer plots.

Caporaso, J.G., Kuczynski, J., Stombaugh, J., Bittinger, K., Bushman, F.D., Costello, E.K., Fierer, N., Pena, A.G., Goodrich, J.K., Gordon, J.I.*, et al.* (2010). QIIME allows analysis of high-throughput community sequencing data. Nature methods *7*, 335-336.

Chen, L., Xiong, Z., Sun, L., Yang, J., and Jin, Q. (2012). VFDB 2012 update: toward the genetic diversity and molecular evolution of bacterial virulence factors. Nucleic acids research *40*, D641-645.

Huson, D.H., Mitra, S., Ruscheweyh, H.J., Weber, N., and Schuster, S.C. (2011). Integrative analysis of environmental sequences using MEGAN4. Genome research *21*, 1552-1560.

Kellermayer, R., Nagy-Szakal, D., Harris, R.A., Luna, R.A., Pitashny, M., Schady, D., Mir, S.A., Lopez, M.E., Gilger, M.A., Belmont, J.*, et al.* (2015). Serial fecal microbiota transplantation alters mucosal gene expression in pediatric ulcerative colitis. The American journal of gastroenterology *110*, 604-606.

Langmead, B., and Salzberg, S.L. (2012). Fast gapped-read alignment with Bowtie 2. Nature methods *9*, 357-359.

Leplae, R., Lima-Mendez, G., and Toussaint, A. (2010). ACLAME: a CLAssification of Mobile genetic Elements, update 2010. Nucleic acids research *38*, D57-61.

Li, H., Handsaker, B., Wysoker, A., Fennell, T., Ruan, J., Homer, N., Marth, G., Abecasis, G., Durbin, R., and Genome Project Data Processing, S. (2009). The Sequence Alignment/Map format and SAMtools. Bioinformatics *25*, 2078-2079.

Minot, S., Sinha, R., Chen, J., Li, H., Keilbaugh, S.A., Wu, G.D., Lewis, J.D., and Bushman, F.D. (2011). The human gut virome: inter-individual variation and dynamic response to diet. Genome research *21*, 1616-1625.

Norman, J.M., Handley, S.A., Baldridge, M.T., Droit, L., Liu, C.Y., Keller, B.C., Kambal, A., Monaco, C.L., Zhao, G., Fleshner, P.*, et al.* (2015). Disease-specific alterations in the enteric virome in inflammatory bowel disease. Cell *160*, 447-460.

Peng, Y., Leung, H.C., Yiu, S.M., and Chin, F.Y. (2012). IDBA-UD: a de novo assembler for single-cell and metagenomic sequencing data with highly uneven depth. Bioinformatics *28*, 1420-1428.

Quinlan, A.R., and Hall, I.M. (2010). BEDTools: a flexible suite of utilities for comparing genomic features. Bioinformatics *26*, 841-842.

Robinson, J.T., Thorvaldsdottir, H., Winckler, W., Guttman, M., Lander, E.S., Getz, G., and Mesirov, J.P. (2011). Integrative genomics viewer. Nature biotechnology *29*, 24-26.

Salzberg, S.L., Delcher, A.L., Kasif, S., and White, O. (1998). Microbial gene identification using interpolated Markov models. Nucleic acids research *26*, 544-548.

Sonnhammer, E.L., Eddy, S.R., Birney, E., Bateman, A., and Durbin, R. (1998). Pfam: multiple sequence alignments and HMM-profiles of protein domains. Nucleic acids research *26*, 320-322.

Treangen, T.J., Sommer, D.D., Angly, F.E., Koren, S., and Pop, M. (2011). Next generation sequence assembly with AMOS. Current protocols in bioinformatics / editoral board, Andreas D Baxevanis [et al] *Chapter 11*, Unit 11 18.
